# RNA interference of an orthologue of Dicer of *Meloidogyne incognita* alludes to the gene’s importance in nematode development

**DOI:** 10.1038/s41598-021-90363-8

**Published:** 2021-05-27

**Authors:** Sadia Iqbal, Michael G. K. Jones, John Fosu-Nyarko

**Affiliations:** Crop Biotechnology Research Group, College of Science, Health, Engineering and Education, WA State Agricultural Biotechnology Centre, Murdoch University, Perth, Australia

**Keywords:** Biotechnology, Functional genomics, RNAi

## Abstract

Dicers and dicer-like enzymes play an essential role in small RNA processing in eukaryotes. Nematodes are thought to encode one dicer, DCR-1; only that for *Caenorhabditis* spp. is well-characterised. Using genomic sequences of eight root-knot nematodes (*Meloidogyne* spp.), we identified putative coding sequences typical of eukaryotic DICERS. We noted that the primary and secondary structures of DICERS they encode were different for different *Meloidogyne* species and even for isolates of the same species, suggesting paralogy for the gene. One of the genes for *M. incognita* (*Midcr-1.1*) expressed in eggs, juvenile stage 2 and adults, with the highest expression in the adult females. All the *Meloidogyne* DICERS had seven major domains typical of those for *Caenorhabditis* spp. and humans with very similar protein folding. RNAi of *Midcr-1.1* in J2s using seven dsRNAs, each based on sequences encoding the domains, induced mild paralysis but measurable knockdown was detected in J2s treated with five of the dsRNAs. For four of the dsRNAs, the RNAi effect lasted and reduced the nematode’s infectivity. Also, host plant delivery of dsRNAs complementary to coding sequences of the Dicer Dimerisation domain impaired development, reducing nematode infection by 71%. These results confirm the importance of the gene to nematode health.

## Introduction

Dicers (DCRs) or dicer-like proteins (DCLs) of eukaryotes are members of a highly conserved family of class III RNases that recognise and cleave double-stranded RNA (dsRNA) substrates to various lengths^1, 2^. They play essential roles in molecular processes such as nucleic acid processing necessary for antiviral defence mechanisms, genome rearrangement, developmental timing, chromatin remodelling, regulation of metabolic processes and stem cell maintenance^3–6^. The involvement of DCRs and DCLs in these biological mechanisms are primarily related to the ribonuclease III activity, which is central to the RNA silencing pathways of eukaryotes^3, 4, 7^. However, the DCR of the free-living nematode, *Caenorhabditis elegans*, CeDCR-1, is also known to have a deoxyribonuclease I activity exhibited during the apoptotic DNA degradation process following cleavage by the cell death protease, CED-3; this activity is independent of the dicer's role in RNA processing and gene silencing^7^.

The role of DCRs and DCLs in RNA silencing is mostly related to the processing of both exogenous and endogenous dsRNA into primary and secondary small interfering RNA (siRNA) duplexes, usually with 5' phosphate and 3' hydroxyl termini^1, 8^ and the biogenesis of microRNA (miRNA) duplexes^9, 10^. These duplexes are the primers or guides for the RNA silencing pathways; when helicases unwind them, they become incorporated into an RNA-induced silencing complex (RISC) which upon activation directs sequence-specific cleavage of complementary messenger RNA (mRNA) leading to post-transcriptional gene silencing (siRNAs) or translational repression (miRNAs)^reviewed^ ^by^^11^. The main functional domains and motifs that determine the activity of DCRs and DCLs are Helicases, Dicer Dimerisation, PAZ (Piwi Argonaut and Zwille), and RNase III domains, and a Double-stranded RNA binding motif^reviewed^ ^by^^12^. The numbers, types and arrangement of the major functional domains and the secondary and tertiary structures may be different for DCRs or their paralogues encoded by the same or different organisms. A typical example is how the two RNase III domains dimerise to generate the dicing activity and, the interaction between the PAZ and RNase III domains which determine the length of small RNA fragments they process^13, 14^.

The structure and function of DCRs and DCLs of many eukaryotes, from humans, plants, insects, fungi and *Caenorhabditis* species, have been studied in some detail. On current evidence, it appears orthology or paralogy of DCRs or DCLs in these eukaryotes has not been driven by the complexity of organisms or the need for processing different lengths or types of small RNAs. For example, the model plants *Arabidopsis thaliana* and *Nicotiana benthamiana* encode four paralogues of DCLs (DCL-1, DCL-2, DCL-3 and DCL-4) which process dsRNAs into functionally diverse small RNAs of distinct lengths (21—24 nucleotides long)^15, 16^. However, insects (e.g. *Drosophila melanogaster*) and fungi (e.g. *Neurospora crassa*) are known to encode two paralogues of DCLs (DCL-1 and DCL-2) both of which process all dicer-generated small RNAs involved in cellular regulation and development^17–19^. For other complex organisms like humans and nematodes, only one major DCR has so far been characterised^20, 21^. The evidence for the existence of a single DCR for nematodes has been based on detailed characterisation of the DCR of *C. elegans,* CeDCR-1 (http://www.wormbase.org).

The CeDCR-1 has all the functional domains typical of ribonuclease III enzymes and processes diverse small RNAs and miRNAs involved in multiple small RNA-mediated pathways^22^. Its role is therefore critical for biological processes that directly dictate successful development through all stages of the nematode, feeding, growth, reproduction and life span^3, 4, 20, 23, 24^. The existence of a DCR in genomes of plant-parasitic nematodes (PPNs) was first demonstrated indirectly when exogenous dsRNAs corresponding to a C-type lectin, a cysteine proteinase and a major sperm protein was shown to have affected the normal biology and behaviour of *Heterodera glycines* and *Globodera pallida*^25^*.* Since then, putative coding sequences of DCRs of many PPNs have been identified from transcriptome and genome sequence data generated through Next Generation Sequencing platforms^26–33^. Despite the recent increase and availability of genomic data, the DCR of any PPN has not been studied to near the same detail as for CeDCR-1.

The analyses in this study used available genomic sequences of eight root-knot nematode species (*Meloidogyne* spp.) to identify putative coding sequences of DCRs encoded by the nematodes and, to predict their functions based on similarity to the architecture of characterised DCRs of other eukaryotes. The only reported attempt to study the role of a DCR in the development of a *Meloidogyne* spp. employed three siRNAs to downregulate the gene’s expression in *M. incognita,* but no significant change in expression was observed in eggs and juvenile stage 2 (J2s) of the nematode^34^. Whether the gene is susceptible to exogenous RNA interference (RNAi) is not known. If this nematode indeed encodes one DCR, then its role may be so crucial that down-regulation of expression may affect several processes, or the organism may have means of resisting the gene's silencing. The importance of the proper functioning of the gene in J2s of *M. incognita* was also investigated using dsRNAs corresponding to coding sequences of the protein domains and one motif typical of eukaryotic DCRs.

## Results

### Putative DCR genes of *Meloidogyne *spp

The amino acid sequence of CeDCR-1 was used to identify putative coding sequences of DCRs from genomic sequences of the eight species of root-knot nematodes (*Meloidogyne* spp.) for which data is currently available at the National Center for Biotechnology Information (NCBI) databases. Twenty-seven genomic contigs and scaffolds with query coverages of 50% or more were identified using TBLASTN; there were six for *M. arenaria* (three isolates), five each for *M. enterolobii* (two isolates)*, M. javanica* (two strains) and *M. incognita* (three isolates)*,* three for *M. luci* strain SI and one contig each for *M. hapla* strain VW9*, M. graminicola* strain IARI and *M. floridensis* isolate SJF1 (Supplementary Table S1). Twenty-one of the contigs with matches across the whole of the CeDCR-1 coding sequences were selected for further study. These included all five contigs of *M. incognita,* five for *M. arenaria,* three for *M. javanica,* all three for *M. luci,* two for *M. enterolobii* and each of the contigs for *M. hapla, M. graminicola* and *M. floridensis*.

The coding sequences of DCRs for *M. incognita* were first delineated within the contigs using FGENESH and FGENESH-C programmes with CeDCR-1 as the template. The putative consensus coding sequence generated was then used to delineate similar coding sequences for the DCRs of the other *Meloidogyne* spp. This approach reduced redundancy in the prediction of the gene sequences for the other *Meloidogyne* spp. The length of predicted gene sequences (from the start codon-exons-introns-stop codon) from the contigs varied among contigs of the same species and between contigs of different species (Supplementary Table S2). Notably, the analyses indicated that different mRNAs may be processed from splicing of 35 or 36 exons into mature mRNAs of varying lengths (Supplementary Table S2). The shortest (5313 nt) and longest (5457 nt) mRNAs were predicted respectively for *M. hapla* and *M. arenaria* (Supplementary Table S2). The overall mean nucleotide diversity in the predicted mRNA sequences was 0.054 with the sequence for *M. graminicola,* the most distantly related without which the overall mean nucleotide diversity was only 0.035 among the twenty sequences. In contrast, the diversity between predicted mRNA sequences for four *Caenorhabditis* spp. (*C. elegans*, *C. brenneri*, *C. remanei* and *C. briggsae*) was ten times higher, at 0.34.

### DCRs of *Meloidogyne* spp

The predicted amino acid sequences of the DCRs for six of the 21 contigs obtained after manual translation were much different from the others, and those obtained from the delineated exonic sequences predicted by FGENESH and FGENESH-C. The differences in the peptides ranged between a loss or addition of five and 52 amino acids (Supplementary Table S3) in the following sequences: four for *M. arenaria* (QEUI01000193, QEUI01000369, RCFJ01030829 and CEWM01002306), one for *M. enterolobii* (RCFM01005457) and one for *M. luci* (CACSLI010000297). The differences in the translated sequences were caused by insertion and/or deletion (INDELS) of bases. When the INDELS were in exons, they caused frameshifting leading to different treatments by FGENESH-C and FGENESH. Manual curation of the sequences was needed to eliminate the frameshifting, and to obtain identical peptides based on consensus comparison of all the sequences and between sequences of the same species. The curation involved the addition of a single base in either an exon or intron except for the coding sequence for contigs RCFM01005457 where “CCTA” was added and QEUI01000369 where in addition to inserting a “G” to an exon, an “A” and “G” were deleted from an intron. Supplementary Data S1 and Table S3 contain all the predicted gene sequences of the DCRs of the eight *Meloidogyne* spp. and the changes made to six of the 21 sequences. In all cases, the missing bases were in regions where there was a high likelihood of base miscalling because of a run of the same nucleotides in the sequence. No such correction could be made for the *M. hapla* and *M. graminicola* sequences because only one putative contig for a DCR was found in the respective genomes.

**Figure 1:**
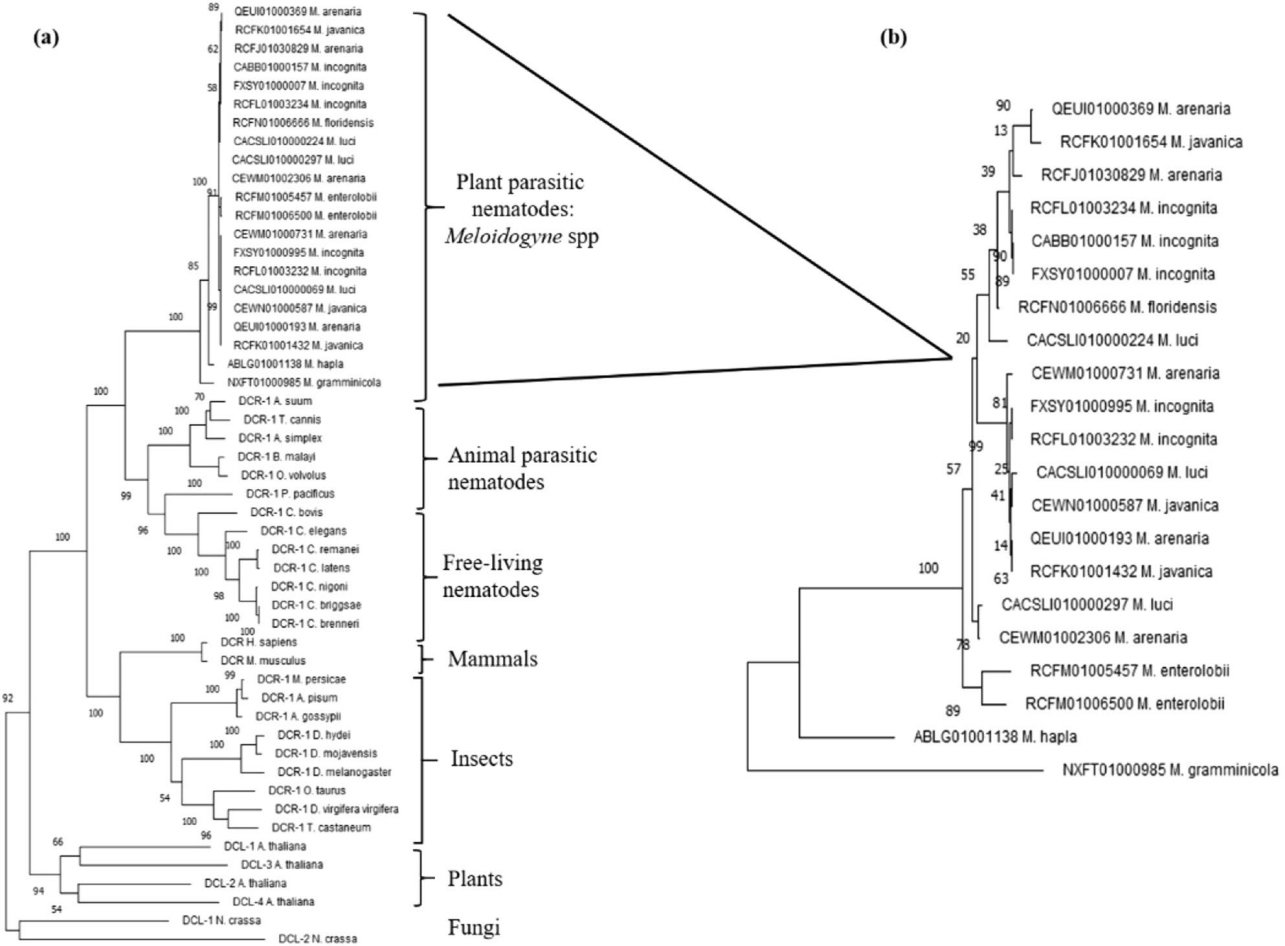
Phylogenetic relationships between DCRs and DCLs of selected eukaryotes. **(a)** Relationships between predicted DCRs of *Meloidogyne* spp. and DCRs and DCLs of plants, fungi, insects, mammals and free-living and animal-parasitic nematodes. **(b)** Relationships between the predicted DCRs of eight *Meloidogyne* spp.

**Figure 2:**
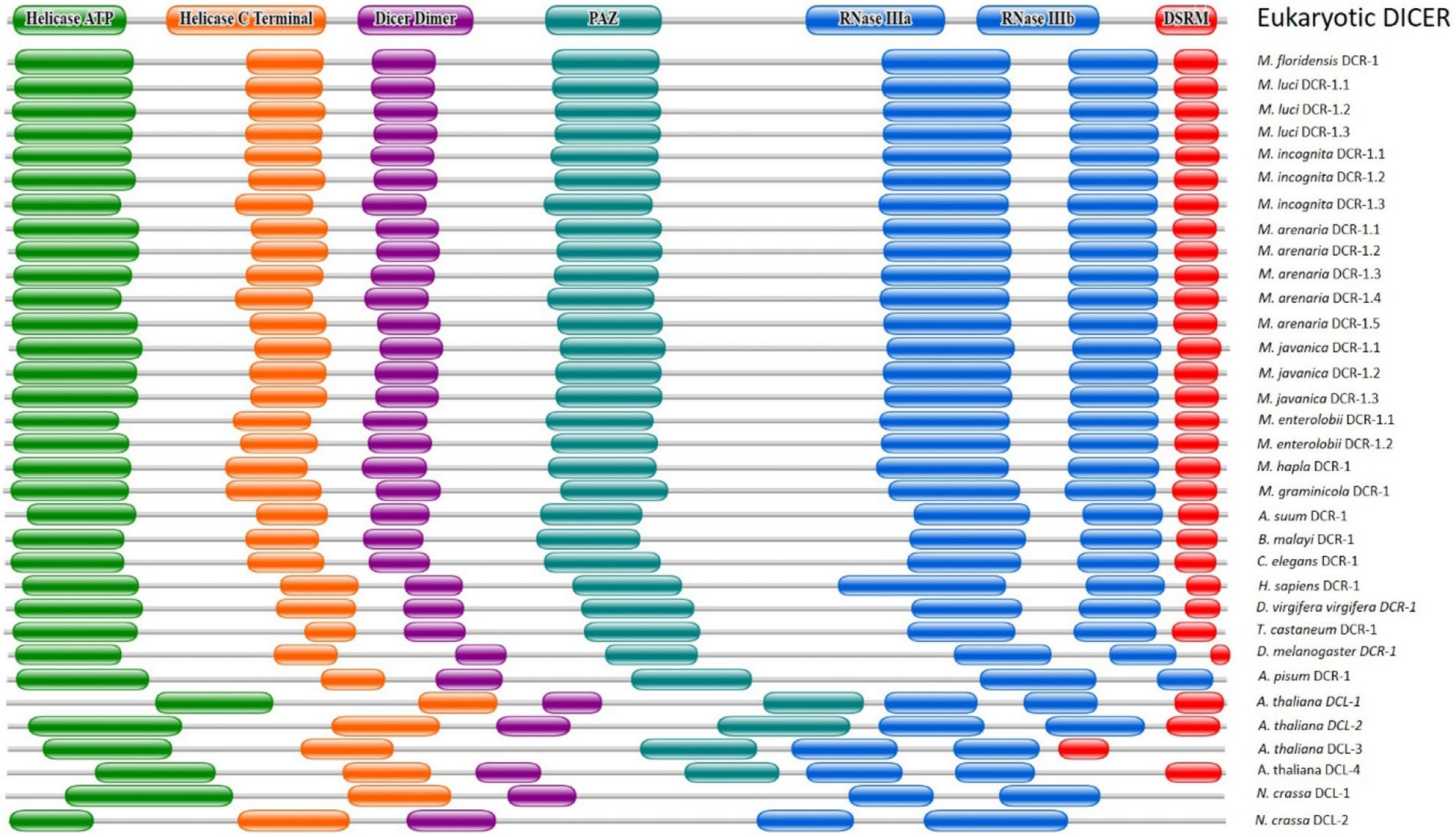
Predicted structures and arrangement of six major domains and a motif of DCRs putatively encoded by genomic contigs of eight *Meloidogyne* species compared to those characterised for a typical eukaryote and other nematodes, insects, plants and fungi.

**Figure 3:**
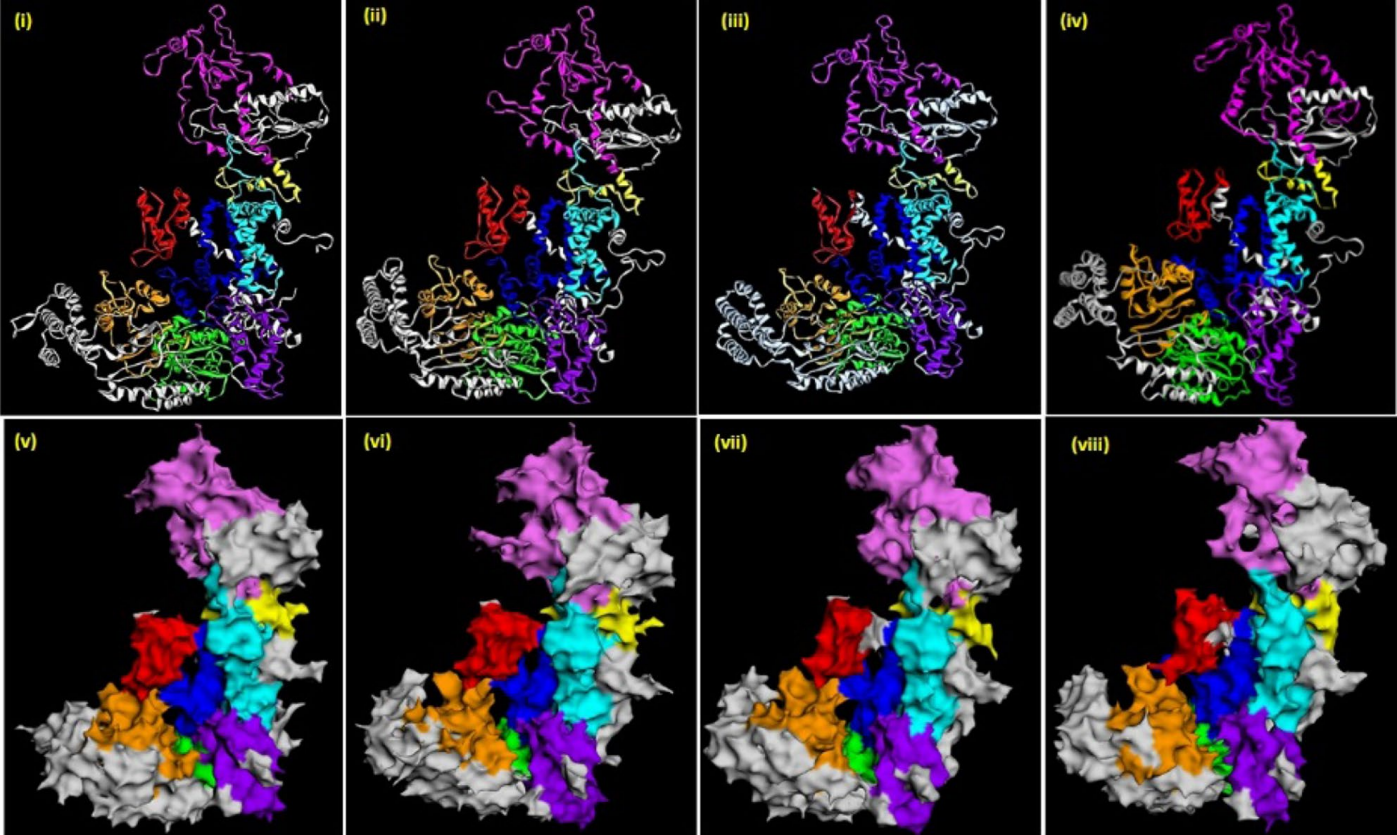
Predicted tertiary structure of MiDCR-1.1 of *M. incognita* (i, v), representative of the eight *Meloidogyne* spp., and those of *C. elegans,* (ii, vi), *H. sapiens* (iii, vii) and *A. thaliana* (iv, viii). The structures were predicted from translated nucleotide sequences of DCRs in the respective genomes using Phyre2 and re-created by EzMol to show the backbone structures and globular protein structures. Designation of domains/motif: *Helicase* *ATP* - helicase ATP binding domain, *Helicase C terminal* - helicase C terminal domain, *Dicer Dimer* - dicer dimerisation domain, *PAZ* PAZ domain, *RNase IIIa* - RNase IIIa domain, *RNase IIIb* -  RNase IIIb domain, *DSRM* - double-stranded RNA binding motif.

The curated nucleotide sequences derived from the 21 contigs putatively encode DCRs of varying sizes, between 1,770 and 1,818 amino acids long (Supplementary Table S2, Data S2). Differences in the translated sequences were not only limited to the types of amino acids, but there were also insertions or deletions of up to 28 amino acids (Supplementary Data S2, Supplementary Fig. S1). Notably, the predicted peptides of DCRs of the same species were not identical (Supplementary Data S2, Fig. S1). However, they were more closely related to each other than representative DCRs of free-living and animal parasitic nematodes, mammals, insects, fungi and plants (Fig. 1). Prediction of functional domains and motifs using databases and tools at Pfam, Conserved Domain Database (NCBI) and ScanProsite indicated all the *Meloidogyne* DCRs had six major functional domains and a motif typical of most characterised eukaryotic DCRs. These were, from the N-terminus, a Helicase ATP binding, a Helicase C terminal, a Dicer Dimerisation, a PAZ, and two Ribonuclease III domains, and a Double-stranded RNA binding motif (Fig. 2). The predicted amino acid sequences of the Ribonuclease IIIb domain and those of the Double-stranded RNA binding motif of the DCRs of the different species were more identical than the other domains; those of the Helicase ATP binding domain were the most diverse (Supplementary Fig. S1). The architecture of the domains confirms the closer relations of the *Meloidogyne* DCRs to eukaryotic DCRs in general (Fig. 2). However, the differences in the primary (amino acid differences) and secondary structures (arrangement of the domains) of the DCRs encoded by different contigs from different, or the same isolates allowed classification of the DCRs as potentially different proteins. These are indicated in Fig. 2, and the Supplementary Table S2 with sub-numerals following the DCRs encoded by the species. The tertiary structure of the *Meloidogyne* DCRs had the L shape characteristic of DCRs of humans, *C. elegans* and *A. thaliana* (Fig. 3) with subtle differences in the folding of the different domains which include the folding of the PAZ and the RNase IIIa domain that eventually determines the sizes of siRNAs cut by the DCRs (Fig. 3).

### RNAi of *Midcr-1.1*

**Figure 4:**
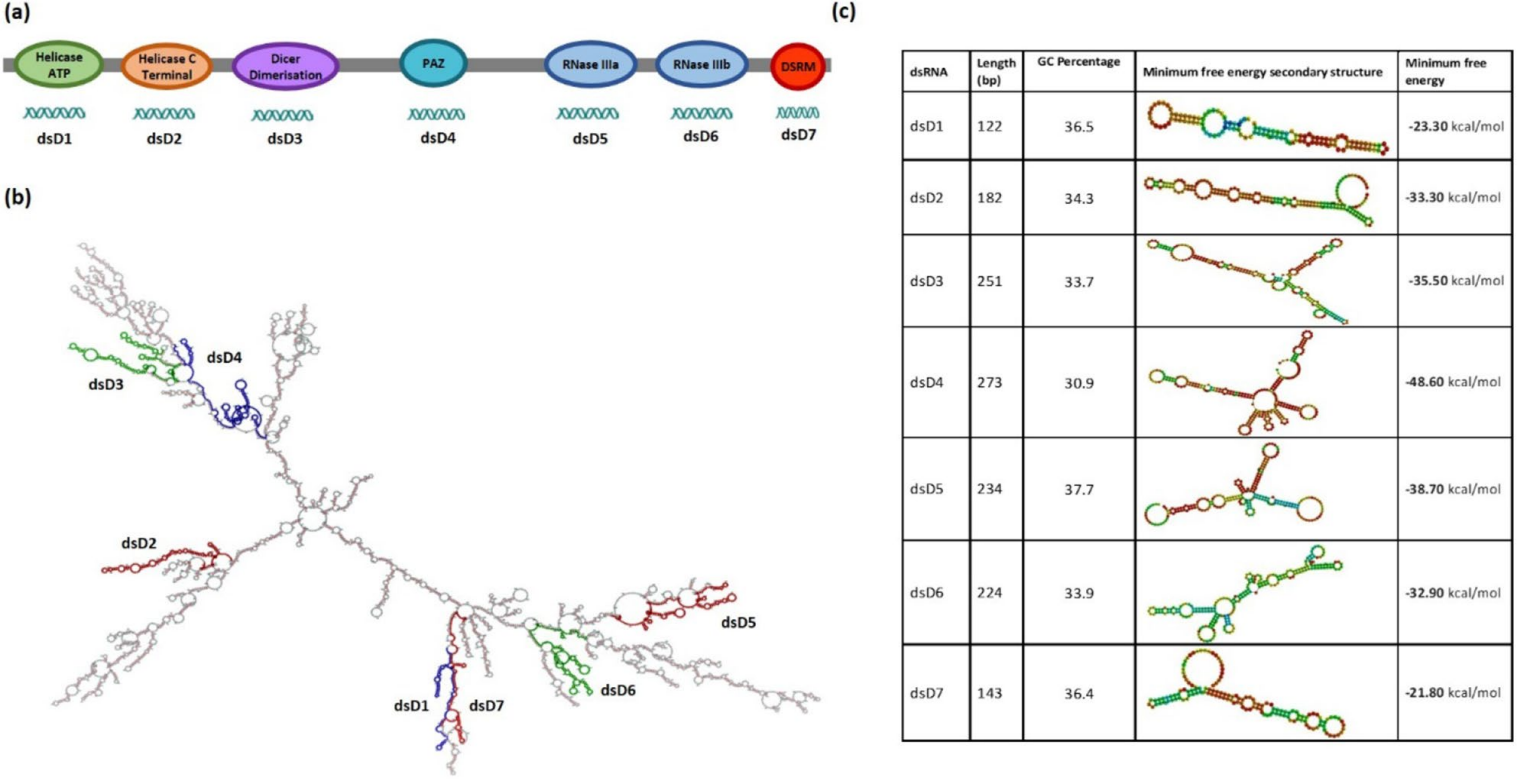
Secondary structures and features of *Midcr-1.1* mRNA and the dsRNA triggers. **(a)** Representation of the six protein domains and motif and the parts used for dsRNA (dsD1 to dsD7) synthesis. **(b)** Secondary structures of *Midcr-1.1* mRNA and the location of the dsRNA triggers. **(c)** Physical and thermodynamic features of the seven dsRNA triggers used to silence the *Midcr-1.1.*

**Figure 5:**
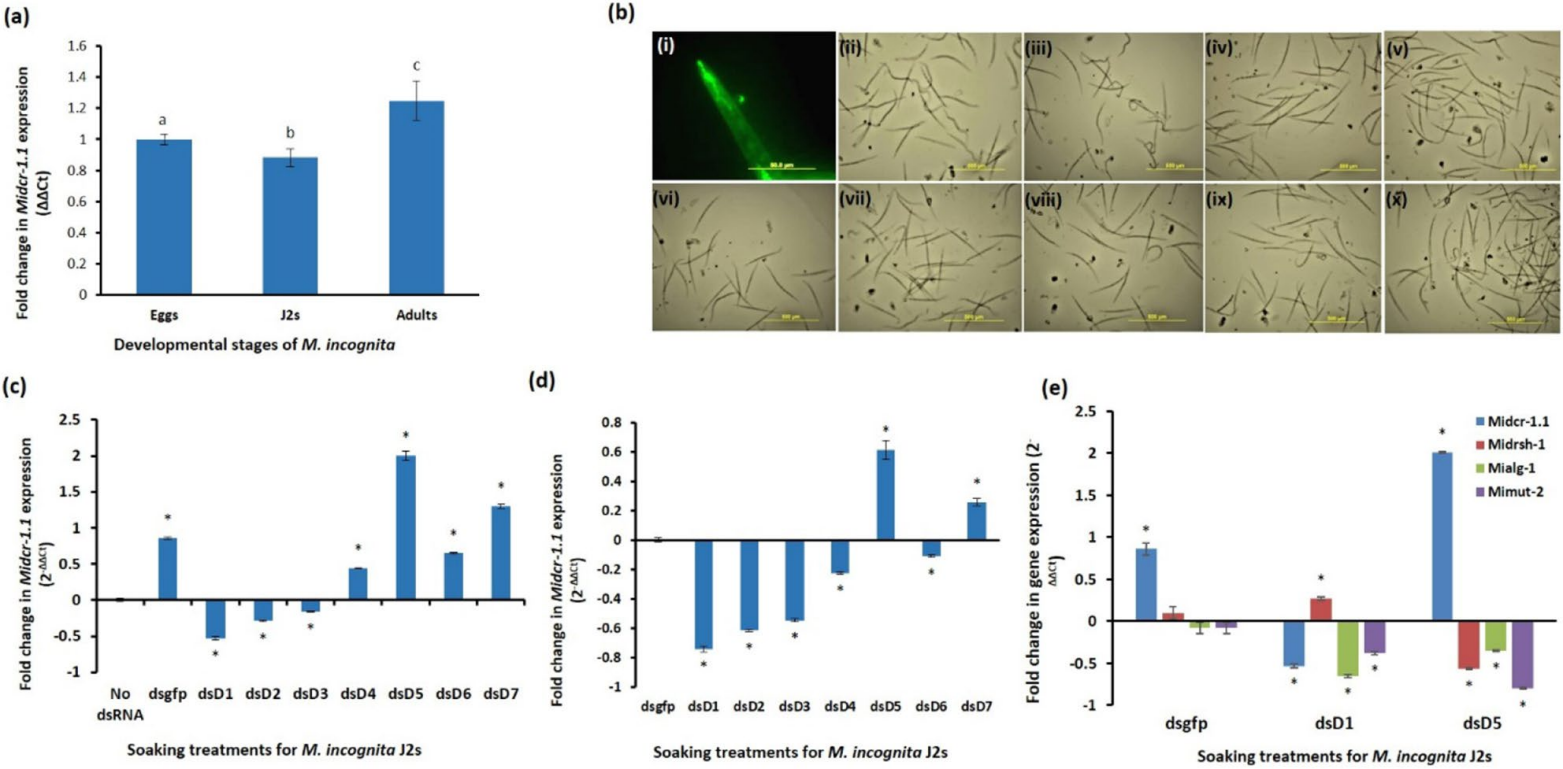
Expression and RNAi of *Midcr-1.1* in *M. incognita.* **(a)** Fold changes in expression of *Midcr-1.1* in eggs, J2s and adult females. **(b)** Phenotypes of J2s 16 h after soaking in buffers with or without dsRNA (i) Representative J2 with the gut, stylet and granules showing uptake of FITC (ii) J2s soaked without dsRNA (iii) J2s treated with dsgfp (iv–ix), J2s treated with seven dsRNAs namely dsD1 (iv)**,** dsD2 (v), dsD3 (vi), dsD4 (vii), dsD5 (viii), dsD6 (ix), and dsD7 (x). **(c,d)** Fold change in *Midcr-1.1* expression in dsRNA-treated J2s relative to expression in J2s treated without dsRNA **(c)** and relative to expression in dsgfp-treated J2s **(d). (e)** Expression of *Midcr-1.1, Midrsh-1, Mialg-1* and *Mimut-2* in J2s treated with dsgfp, dsD1 and dsD5. Means significantly different from the controls are indicated by different letters or “*”; they were determined at *p* < *0.05* using T-test (two-tailed) with three replicates. Error bars represent standard error.

Seven dsRNAs each correspond﻿ing to parts of the coding sequences of the six protein domains and a motif of MiDCR-1.1 were used to assess the importance of the protein in the development and infectivity of J2s of *M. incognita*. These were dsD1 for the Helicase ATP binding domain, dsD2 for the Helicase C terminal domain, dsD3 for the Dicer Dimerisation domain, dsD4 for the PAZ domain, dsD5 and dsD6 for the two Ribonuclease III domains and dsD7 for the Double-stranded RNA binding motif (Fig. 4a). The locations of the dsRNAs on the tertiary structure of the mRNA of *Midcr-1.1* are shown in Fig. 4b; they were 122 to 273 nt long, with GC percentages between 30.9% and 37.7%. Subsequently, their tertiary structures and minimum free energies were different (Fig. 4c). The expression of *Midcr-1.1* was compared in three developmental stages of *M. incognita*: eggs, J2s and adult females; it was highest in the adult white females (*p* < *0.05*, Fig. 5a) than in the egg and J2 stages. The results gave us the confidence to study the possible effect of downregulation of the gene on the J2s of the nematode. The J2 stage is a developmental stage that permitted relatively easier assessment of RNAi.

The dsRNAs (1 mg/mL) were each added to a soaking buffer with 7,000 active J2s, and the effect of ingesting the triggers on the nematodes assessed 16 h later. In a separate set up where nematodes were soaked with 1 mg/mL fluorescein isothiocyanate (FITC) for the same period, the fluorescence in the stylet and body of the J2s indicated the nematodes had ingested the external solution (Fig. 5b (i)). This observation suggested that when J2s were soaked with dsRNAs in similar buffers without FITC, the nematodes would have ingested some dsRNA together with the solutions. The J2s soaked in the buffer without dsRNA or with dsgfp (a 500 nt dsRNA corresponding to the sequence of the Green Fluorescent Protein gene of *Aequorea victoria*) were as active and mobile as healthy untreated nematodes. The nematodes soaked with the seven dsRNAs (dsD1–dsD7) targeting the *Midcr-1.1* were mostly inactive; they had a straight body posture with slight movement only in the head region, with the lower body paralysed (Fig. 5b (iv–x)).

The higher expression of the *Midcr-1.1* in dsgfp-treated J2s 16 h after treatment indicated the gene and or the protein it encodes was activated when the nematodes were soaked in the dsgfp (Fig. 5c). The gene’s expression in J2s treated with dsD1 to dsD7 was therefore calibrated against expression in J2s treated with dsgfp to accurately assess the effect of the different *Midcr-1.1* dsRNAs on gene expression. The expression of *Midcr-1.1* in J2s treated with dsD1, dsD2, dsD3, dsD4 and dsD6 was reduced by up to 74% (Fig. 5d). *Midcr-1.1* expression in J2s treated with dsD5 and dsD7, on the other hand, increased by 61% and 26% respectively (Fig. 5d). The expression of three *C. elegans* orthologous genes, the drosha ribonuclease III (*drsh-1*), the argonaute (*alg-1)* and the nucleotidyl transferase (*mut-*2), respectively labelled as *Midrsh-1, Mialg-1* and *Mimut-2,* were also quantified in J2s treated with dsgfp, dsD1 and dsD5. Fold changes in the expression were calibrated with expressions in J2s treated without dsRNA. The aim was to assess how downregulation and upregulation of the *Midcr-1.1* affected the expression of some genes, especially those also involved in the RNAi pathway. The nematodes treated with dsD1 and dsD5 were selected because expression in them represented the two extremes for *Midcr-1.1* expression after RNAi treatment (Fig. 5e). Unlike the *Midcr-1.1*, the expression of *Midrsh-1*, *Mialg-1* and *Mimut-2* were not significantly affected in nematodes treated with dsgfp (*p* < *0.05*, Fig. 5e). The expression of the three genes were, however, affected to different degrees in dsD1- and dsD5-treated J2s. Except for the expression of *Midrsh-1* in dsD1-treated J2s where there was a 27% increase, there was generally a significant (*p* < *0.05*) downregulation. The most reduction, 80%, was observed for *Mimut-2,* in the dsD5-treated J2s (*p* < *0.05*, Fig. 5e).

### RNAi of *Midcr-1.1* affects parasitism and development of treated J2s

**Figure 6:**
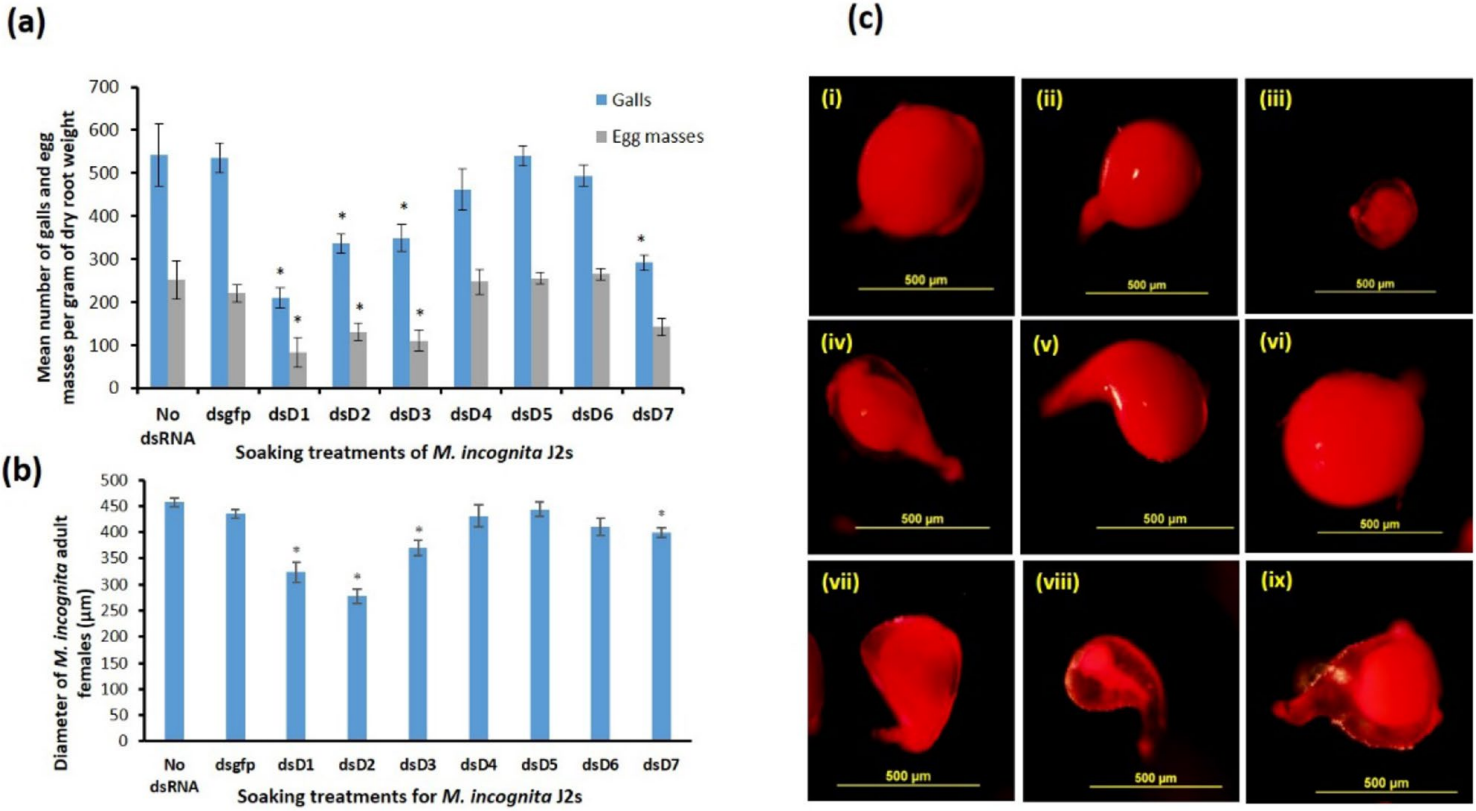
Infectivity and development of dsRNA-treated J2s on tomato seedlings. **(a)** Bars are means (n = 7) with standard errors of the number of galls and egg masses per gram of dry root weight of infected plants. **(b)** Mean diameter with standard errors (n = 14 to 25) of adult females dissected out of tomato roots seven weeks after inoculation with treated J2s. Note: One outlier was removed from the dsgfp data before statistical analysis; it was 395 µm in diameter. **(c)** Representative acid fuchsin-stained females isolated from tomato roots seven weeks after inoculation with J2s previously treated with (i) buffer without dsRNA (ii) dsgfp (iii) dsD1 (iv) dsD2 (v) dsD3 (vi) dsD4 (vii) dsD5 (viii) dsD6 (ix) dsD7. Means significantly different from the means of control J2s (no dsRNA and dsgfp) at *p* < *0.05* were ascertained using T-test (two-tailed) and are represented by “*”.

Infectivity of the treated J2s was investigated by comparing the signs of infection on tomato seedlings seven weeks after inoculation with 400 J2s per plant. There were no significant differences in the mean numbers of galls or egg masses per gram of dry root weight (galls/gdrw) for the plants inoculated with the control J2s (treated with dsgfp and without dsRNA) and those previously treated with dsD4, dsD5 and dsD6 (*p* < *0.05,* Fig. 6a). Treatment of J2s with the dsD1, which induced the maximum knockdown of *Midcr-1.1* in J2s, resulted in the highest reduction in nematode infection. For these J2s, there were 61% and 67% reductions in the numbers of galls and egg masses compared to those induced by the control nematodes (*p* < *0.05,* Fig. 6a). Infectivity of the dsD2- and the dsD3-treated J2s was also significantly reduced: there was 38% and 35% fewer galls/gdrw and resulted in 48% and 56% fewer egg masses, respectively in the plants. The infection of the dsD7-treated J2s resulted in 46% reduction in galls/gdrw, but there was no significant change in the mean number of egg masses compared to those on plants infected with the control J2s (Fig. 6a).

The development of nematodes in the infected roots was also investigated using their sizes, shape and consistency seven weeks after inoculation. Developing and maturing white females were dissected from galls, stained with acid fuchsin and observed under a stereomicroscope. The females that developed from the J2s previously treated with dsD1, dsD2, dsD3 and dsD7 were significantly smaller compared to the controls, with the most affected being those treated with dsD1 and dsD2 (*p* < *0.05*; Fig. 6b,c). Females that developed from J2s treated with dsD2 and dsD1 were mostly elongated compared to the normal saccate shape of those that developed from control J2s. The transparency in the females that developed from J2s treated with dsD1, dsD6 and dsD7 indicated they were malformed (Fig. 6c iii, viii, ix). The mean size of the females of the dsD4-treated J2 lineage was not different from those that developed from the control J2s. Those of the dsD3-treated J2 lineage were not necessarily malformed, but the relatively smaller sizes were consistent with delayed development (Fig. 6c v).

### Host induced gene silencing (HIGS) of *Midcr-1.1* reduces *M. incognita* infectivity

**Figure 7:**
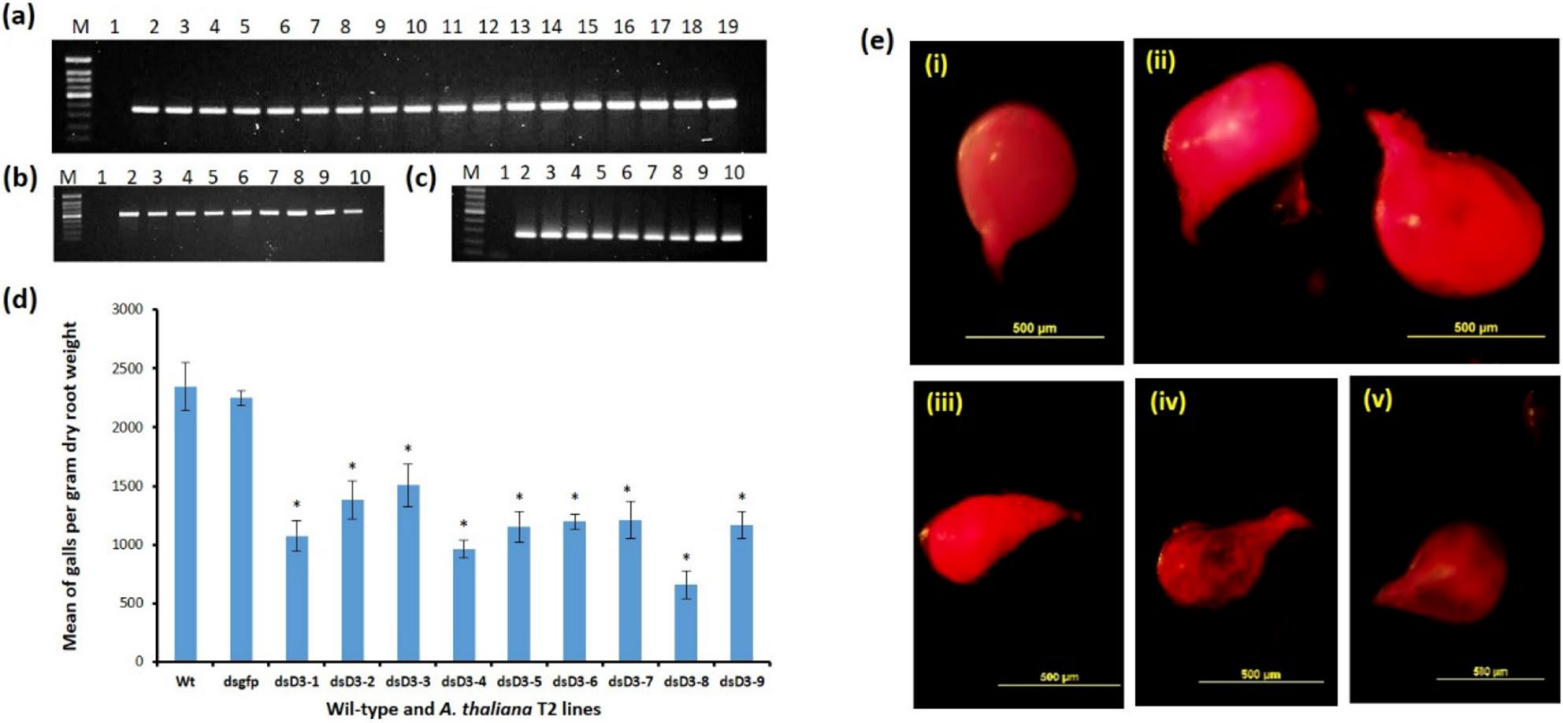
Characterisation of T2 transgenic *A. thaliana* lines. **(a)** PCR bands of the *nptII* gene (364 bp) from genomic DNA of transgenic lines. Lane M = 100 bp DNA marker, Lane 1 = No DNA control, Lane 2 – 10 = Bands from nine lines of dsgfp T2 plants, Lane 11–19 = Bands from nine lines of dsD3 T2 plants **(b)** RT-PCR of dsgfp hairpin from RNA of the nine T2 dsgfp lines. Lane M = 100 bp DNA marker, Lane 1 = No cDNA control, Lane 2–10 = Bands (524 bp) from cDNA of dsgfp from nine dsgfp lines. **(c)** RT-PCR of dsD3 hairpin from RNA of the nine T2 dsD3 lines. Lane M = 100 bp DNA marker, Lane 1 = no cDNA control, Lane 2–10 = Bands (251 bp) from cDNA of dsD3 from nine dsD3 lines. **(d)** Mean plus standard errors of the number of galls per gram of dry root weight on T2 Arabidopsis lines carrying hairpins of dsgfp, and nine lines of dsD3 (Dcr1-1 to Dcr1-9). Means significantly different from that of controls at *p* < *0.05* ascertained by using T-test (two-tailed) based on 14 replicates for each transgenic line and 20 replicates for wild-type plants. **(e)** Acid-fuchsin-stained representative adult *M. incognita* females dissected from infected wild-type and T2 transgenic lines carrying hairpins of dsgfp and dsD3. Females dissected out of (i) wild-type plants (ii) dsgfp plants (iii,v) dsD3 plants.

The sequence of the dsD3 (Dicer Dimerisation domain) was used to create transgenic plants to study HIGS and the effectiveness of the plants compared to nematode infection on transgenic lines harbouring hairpins of the dsgfp. Transgene integration and transcription of the corresponding hairpins and the selectable *nptII* gene in nine T2 Arabidopsis lines for each dsRNA were confirmed with PCR and RT-PCR (Fig. 7a–c). The susceptibility of the T2 lines (14 replicates per line) and 20 wild-type Arabidopsis Col 0 plants was assessed four weeks after inoculation with 200 freshly hatched J2s of *M. incognita*. There was no significant difference in the mean number of galls/gdrw on the T2 dsgfp lines and the wild-type plants (*p* < *0.05*). The mean number of galls/gdrw on replicates of the nine T2 dsgfp lines were very similar and were therefore represented as a single mean in the bar graphs to make comparison with those of the dsD3 lines easier. The mean numbers of galls/gdrw on each of the nine T2 dsD3 lines were significantly less than that of the wild-type and dsgfp lines (*p* < *0.05*). For five of the nine dsD3 lines, nematode infection was reduced by 50% or more, with the most significant reduction of 71% for the dsD3-8 line (Fig. 7d). As with the females that developed from J2s treated with dsRNAs of the *Midcr-1.1 *in vitro*,* the adult females dissected from roots of the T2 dsD3 lines were malformed, transparent and smaller than those on roots of the dsgfp lines and wild-type plants (Fig. 7e).

## Discussion

The existence of DCRs in the genomes of PPNs has been predicted in many transcriptomes and genomic sequences^27–29, 31^. Their functions in the small RNA processing pathway has been presumed in many reported gene silencing experiments; so far detailed characterisation of the gene(s) or the proteins they encode is lacking. Using available genomic sequences, we showed that eight *Meloidogyne* spp. potentially encode DCRs of different primary, secondary and tertiary structures, although they are similar to those characterised for the free-living nematode *C. elegans*, humans and Arabidopsis. Notably, the genomes of the three species for which sequences of only one isolate/strain was available, *M*. *hapla, M. graminicola,* and *M. floridensis*, appear to encode one DCR. The primary sequences and arrangement of major domains of the multiple DCRs encoded by the same strain of *M. luci*, and those of different strains and isolates of *M. javanica, M. incognita* and *M. arenaria* suggest these nematode species potentially encode DCRs with structural or functional variance. Do these observed differences represent paralogy of the DCR in root-knot nematodes? Because variations in copy number of genes resulting from adaptation to different hosts or locations has previously been observed in *M. incognita*^35, 36^, there may be paralogues of DCRs in these nematodes. Whether these differences we report in this study represent true paralogy or arise from artefacts from Next Generation Sequencing and analyses may be resolved using transcriptional and functional characterisation of the full-length mRNA transcripts of the various DCRs.

The functional relevance of *Midcr-1.1* was deduced from its expression in eggs, J2s and adult females, and the effect downregulation had on the biology of J2s. The different effects of *Midcr-1.1* knockdown on the J2s induced by the seven dsRNAs used in the study, however, suggest the nature of the dsRNAs and perhaps the region of the gene targeted may have contributed to the differences in efficiencies of the dsRNAs in inducing knockdown. The dsRNAs which were complementary to and targeted degradation of the mRNA of *Midcr-1.1* at the 5’ end induced more significant knockdown than those targeting the 3’ end of the gene (*p* < *0.05*). The only other study which attempted to silence a DCR gene in eggs and J2s of *M. incognita* suggested the gene was recalcitrant to exogenous RNAi^34^. They observed significant upregulation of the gene in eggs soaked in three different siRNAs without any observed impact on egg differentiation or hatching. For the J2s, all three siRNAs failed to reduce the dicer transcript significantly^34^. Interestingly, two of the three Dicer siRNAs they used (siRNA 1 and 2) were located within the coding sequences of the Ribonuclease IIIa domain, which was the target region for dsD5, the ingestion of which did not result in downregulation of *Midcr-1.1* in the J2s in our study. Their Dicer siRNA 3 was located 33 bp upstream of the PAZ domain, and this region was not included as a target in our study. The results of Dalzell et al., (2010) did not indicate which contig of *M. incognita* was used in their research. However, the timeline for publication of the *M. incognita* genome suggests they would have used sequences of *Midcr*-1.1. Their results, therefore, confirm our suspicion that the sequence complementary to the dsD5 was not the best region to target for downregulation of the *Midcr-1.1*.

This study also indicated that *Midcr-1.1* is susceptible to exogenous RNAi, unlike some RNAi-recalcitrant genes^37–39^, but the efficiency may depend on the dsRNA used. Such differential knockdown triggered by long and short dsRNAs of different structures complementary to different regions of the same mRNA of several genes has been reported in diverse eukaryotes^40–44^. Properties of the dsRNAs that may have influenced the extent of the *Midcr-1.1* knockdown included secondary structures, length, and GC content, which eventually determined the formation of secondary structures and the associated differences in minimum free energies (MFE). However, there was no strong correlation between the level of gene knockdown induced by the dsRNAs and two properties of the dsRNAs: GC content (*r* = 0.4) and MFEs (*r* = − 0.13). It is, therefore, plausible other mechanistic properties such as accessibility of target mRNA by an activated RISC, which has been reported to contribute to RNAi efficiency by up to 40% may be involved^45^. Also, *si-Fi* analyses of the effectiveness of processed siRNAs indicated that all seven dsRNAs used in the study could be processed into effective siRNAs.

Generally, it was expected that expression of the *Midcr-1.1* would increase following increased activity of the MiDCR-1.1 when exogenous dsRNA is introduced into cells or when the small RNA pathways are activated. The two-fold increase in expression of *Midcr-1.1* in J2s soaked in dsRNA of GFP followed this pattern. Treatment of J2s with the non-nematode sequence (dsgfp), or the dsD1 or dsD5, which induced up- and downregulation of the *Midcr-1.1* respectively, all resulted in downregulation of the argonaute gene *Mialg-1* and the *Mimut-2* gene. However, transcript changes in both genes were generally less pronounced in J2s treated with dsgfp than in those treated with dsRNA of the target *Midcr-1.1*. Whether this has implications for a recognition system in the nematode for specific processing and differential engagement of the RNAi components (proteins and complexes) with target and non-target dsRNAs and siRNAs is not clear. It is now evident that some concentrations of control, non-nematode dsRNA targets including GFP can have some phenotypic effects on nematodes (*M. incognita* and *Globodera pallida*)^46^ and the honey bee, *Apis mellifera*^47^. No such phenotypic effects were observed in our experiments. This observation was supported by our *si-Fi* analyses of potential siRNAs that could have been generated from the 524 bp fragment of the GFP we used. No predicted siRNA of 18–25 bp from the dsgfp was homologous to any mRNA predicted from the genome of *M. incognita*. Of the 12 siRNAs (three 18 bp and nine 17 bp siRNAs) predicted as potentially homologous to nine predicted mRNAs of *M. incognita*, none was an effective siRNA as determined by the parameters of the *si-Fi* software^48^. While it is clear from our results that upregulation of the *Midcr-1.1* in J2s following exposure to dsgfp possibly indicated the activation of the small RNA pathway when the dsRNA entered the cells of the nematodes, the activity of other proteins of the RNAi machinery perhaps treats genome-specific and non-specific siRNAs differently as indicated by the differential expression of the *Mialg-1* and *Mimut-2* in J2s treated with the dsRNAs of the *Midcr-1.1* and the non-nematode dsgfp.

Our results also provide crucial evidence that because the MiDCR-1.1 of *M. incognita* is involved in the RNAi mechanism and is relevant for normal development, its knockdown can be exploited for control of the nematode and other plant-parasitic nematodes using HIGS. Proof of this concept was demonstrated with nine *A. thaliana* transgenic lines harbouring hairpins corresponding to sequences of the Dicer Dimerisation domain of the MiDCR-1.1 where there was significantly reduced infectivity of J2s, followed by impaired development of female adult nematodes. For practical applications of HIGS to control plant nematode pests, specific dsRNAs without sequence similarity to dicers of crop plants and other beneficial organisms will need to be designed and employed. The availability of genomic sequences for crop plants and pests can facilitate the process. Also, the use of root-specific promoters particularly, those induced by nematode infection to drive expression of dsRNAs can restrict expression to roots further reducing unintended effects on beneficial insects which may feed on aerial parts of transgenic plants.

## Materials and methods

### Sequence analyses of contigs of *Meloidogyne* spp. coding for DCRs

The amino acid sequence of the *C. elegans* dicer-1, CeDCR-1 (Wormbase Protein ID K12H4.8) was used to identify putative coding sequences of DCRs of root-knot nematodes. TBLASTN searches with a threshold expected value of 1E−10 was used to search the whole genome sequence databases of eight *Meloidogyne* species. Genomic contigs with at least 50% query coverage for the *Meloidogyne* species, *M. incognita, M. hapla, M. javanica, M. graminicola, M. arenaria, M. enterolobii, M. floridensis* and *M. luci* were downloaded from the NCBI database in May 2020. Putative gene sequences of DCRs from the *Meloidogyne* contigs, that is the mRNA, exons, introns and the putative protein sequences were predicted using FGENESH and FGENESH-C with the CeDCR-1 as the query^49, 50^. The MultAlin program was used to compare the sequences^51^. MEGA7 was used to generate phylogenetic trees and distances between the predicted mRNA and translated amino acid sequences of the DCRs and those of other eukaryotes. Phylogenetic trees were constructed using Maximum Likelihood, Neighbour-joinging and Minimun-evolution based approaches and the most consistent tree (the Maximum Likelihood method based on the JTT matrix-based model) with 1000 bootstrap replicates selected for presentation^52, 53^. Domains in the putative DCRs were identified using Pfam (threshold e-value 1.0)^54^, the NCBI Conserved Domain Database (threshold e-value 0.01)^55^ and the ScanProsite tool^56^. The identified domain structures were re-drawn with the Generate domain images tool (Pfam)^54^. The secondary structure of the putative mRNA of MiDCR-1.1 and the minimum free energies of the dsRNAs were predicted using the RNAfold Web server (http://rna.tbi.univie.ac.at/cgi-bin/RNAfold.cgi). The tertiary structures of DCRs of *H. sapiens* (Accession No. NP_001258211), *C. elegans* (Wormbase ID: K12H4.8), *A. thaliana* (Accession No. NP_001184881) and MiDCR1.1 (*M. incognita* from the genomic contig CABB01000157) were predicted with Phyre2 (Protein Homology/analogY Recognition Engine V 2.0, http://www.sbg.bio.ic.ac.uk/~phyre2) using the normal Modelling mode settings. The results were downloaded as a .pdb file which was then uploaded into EzMol (http://www.sbg.bio.ic.ac.uk/ezmol) to create the backbone and globular models of various domains and motifs. Potential off-target effects of the dsgfp and effectiveness of knockdown by the dsRNAs corresponding to *Midcr-1.1* were assessed using si-Fi software—this included identifying effective siRNAs or in the case of dsgfp, identifying potential siRNAs corresponding to available sequences of *Meloidogyne* spp.^48^ using *M. incognita* genome (NCBI-BioProject PRJEB8714) and the predicted mRNA database (mRNA_MincV3) downloaded from INRAe-Meloidogyne genomic resources (https://www6.inrae.fr/meloidogyne_incognita). The default settings of all web-based tools were used for all analyses.

### Reverse transcription, PCRs and quantitative PCRs

Total RNA from nematodes (30 white females, 2000 J2s from RNAi experiments, 10,000 untreated J2s and 50 egg masses) and plants (200 mg) was extracted using the Trizol method followed by DNase I treatment as described by Tan *et al*.^57^ Complementary DNA was generated from 500 ng of RNA from the mixed stage nematodes or 100 ng of RNA each from the egg, J2s or adult stages or 200 ng of RNA from leaves using the High-Capacity cDNA Reverse Transcription kit (Applied Biosystems, USA). Genomic DNA from 100 mg of leaves of *A. thaliana* plants was isolated using the CTAB method^58^. PCRs were done using the 2 × GoTaq^®^ Green Master Mix (Promega Corporation, Australia) using the conditions described by Iqbal *et al*.^38^ Standard molecular techniques were used to clone and sequence amplicons and DNA vectors^38^. All primers used for PCRs are provided in Supplementary Table S4.

Quantitative PCRs were done in triplicates using the GoTaq® qPCR 2 × Master Mix (Promega Corporation, Australia) with 10 µM of primers in a Corbett RotorGene Quantitative Thermal Cycler (Qiagen Pty Ltd., Australia). Primers used to amplify genes after dsRNA treatment were designed outside of the exon region used to synthesise dsRNAs (Supplementary Table S4). Expression of the actin gene (Accession no. BE225475) of *M. incognita* was used to normalise the expression of other genes using the ∆∆C_T_ method^59^. Melting curves were used to ensure all primers were specific and had > 95% efficiencies using the same temperature profiles for all qPCRs described by Iqbal *et al*.^38^

### Synthesis of dsRNA and RNAi induction via soaking

The DNA templates used to synthesise dsRNAs corresponding to the mRNA of the six protein domains and one motif of the *Midcr-1.1* were generated using the transcription vector pDoubler as described by Fosu-Nyarko *et al*.^60^ DsRNAs were prepared using the HiScribe T7 In vitro Transcription Kit (New England BioLabs, Australia) following the procedures described by Iqbal *et al*.^38^ For each RNAi soaking treatment, 7000 freshly hatched *M. incognita* J2s were used. The soaking experiments with 1 mg/mL dsRNAs corresponding to the mRNA of the domains and motif of MiDCR-1.1 (dsD1–dsD7), the buffers used, the control dsRNA of the GFP gene (dsgfp) and incubation time and temperature of the set-ups were as described previously^38^. The effect of soaking nematodes in dsRNA was assessed using an Olympus BX-51 microscope with the FITC filter and by comparing expression of the target *Midcr-1.1* in nematodes treated with any of the dsD1–dsD7 and those treated with dsgfp.

### Generation of T2 transgenic lines for HIGS

The sense and the antisense sequence used for dsD3 dsRNA were ligated sequentially to the pCleaver vector, and the hairpin cassette, driven by the 35S promoter was transferred to the binary vector, pART27^61^ as described by Iqbal *et al*.^38^ A similar binary vector was created with a hairpin cassette containing 500 bp of the GFP gene^38^. These were used to generate transgenic *A. thaliana* lines using *Agrobacterium*-mediated floral dip transformation^62^. For both dsD3 and dsgfp hairpins, nine T2 transgenic lines were used for HIGS; they were generated after antibiotic selection followed by PCR of the *nptII* gene and RT-PCRs of the dsgfp and dsD3 hairpins used to confirm their transgenic status using the methods previously described.^29,38^

### *M. incognita* cultures, inoculation and assessment of nematode infection

Freshly hatched *M. incognita* J2s, maintained on the susceptible tomato cv. Grosse Lisse, were used for RNAi experiments. Four hundred dsRNA-treated nematodes and controls treated with dsgfp and without dsRNA were each used to inoculate ten tomato seedlings (cv. Grosse Lisse) each growing in a 120 cm^3^ pot. The nematodes were pipetted 2–3 cm deep into the soil at four points, 1–2 cm away from the main stem of the plants. For HIGS, 200 active J2s were used to inoculate 14 replicates of three-week-old transgenic plants and 20 wild-type *A. thaliana* plants. Inoculation involved pipetting J2s suspended in water 2 cm deep into the soil at four sides of each test plant. Seven infected tomato plants, 16 wild-type and ten transgenic *A. thaliana* plants from each line were assessed four weeks after inoculation using the numbers of galls and egg masses per gram of dry root weight. Adult female nematodes were dissected out of the roots of the remaining tomato, and *A. thaliana* plants seven and five weeks after inoculation, respectively, and stained with acid fuchsin to assess their consistency. Egg masses stained with 0.05% phloxine B were counted under a dissecting microscope. Infectivity was expressed as the number of galls or egg masses per gram of dry root weight. Roots were dried at 55° C for a day. Images of stained adult females were captured using an Olympus BX-51 microscope. Their diameter was measured from the captured images using Mountains® 8 software (https://www.digitalsurf.com).

### Statistical analysis

Correlation analyses, as well as means, standard errors and bar charts for all parameters, were generated using the Microsoft Excel Analysis ToolPak. Analysis of variance followed by T-test (two-tailed) was used at *p* < *0.05* to determine significant differences between all means.

## Supplementary Information

Supplementary Information.
