# Supplementary material for: RNA interference of an orthologue of Dicer of Meloidogyne incognita alludes to the gene’s importance in nematode development
Source: Sci Rep. 2021 May 27;11:11156. doi: 10.1038/s41598-021-90363-8 (PMC8160347; doi:10.1038/s41598-021-90363-8)

**Supplementary Information**

.

RNA interference of an orthologue of Dicer of *Meloidogyne incognita* alludes to the gene’s importance

in nematode development.

Sadia Iqbal, Michael G.K. Jones and John Fosu-Nyarko

Crop Biotechnology Research Group, College of Science, Health, Engineering and Education, WA State Agricultural Biotechnology Centre, Murdoch University, Perth, Australia

**Supplementary Table S1:** TBLASTN (Threshold e-value 1E-10, May 2020) results for eight *Meloidogyne* species using *C. elegans* DCR-1 amino acid sequence (1910 aa) as query and *Meloidogyne* spp. whole genome shotgun sequences as the target database. Genomic contig Accession numbers, description, query coverage and percent identities are indicated.

| **Accession No.** | **Description** | **Max Score** | **Total Score** | **Query Cover** | **E value** | **Per. Ident** |
| --- | --- | --- | --- | --- | --- | --- |
| CEWM01002306 | *Meloidogyne arenaria* genome assembly, contig: Meloidogyne_arenaria_scaffold_2306, whole genome shotgun sequence | 152 | 1221 | 69% | 1.00E-105 | 53.79% |
| CEWM01006559 | *Meloidogyne arenaria* genome assembly, contig: Meloidogyne_arenaria_scaffold_6559, whole genome shotgun sequence | 254 | 1186 | 64% | 2.00E-71 | 29.60% |
| CEWM01000731 | *Meloidogyne arenaria* genome assembly, contig: Meloidogyne_arenaria_scaffold_731, whole genome shotgun sequence | 154 | 1240 | 76% | 2.00E-64 | 43.98% |
| QEUI01000193 | *Meloidogyne arenaria* isolate A2-O Okinawa_01 tig00001575, whole genome shotgun sequence | 189 | 1097 | 74% | 9.00E-48 | 33.75% |
| QEUI01000369 | *Meloidogyne arenaria* isolate A2-O Okinawa_01 tig00001649, whole genome shotgun sequence | 160 | 1137 | 62% | 7.00E-37 | 39.34% |
| RCFJ01030829 | *Meloidogyne arenaria* isolate HarA scaffold36919_cov73, whole genome shotgun sequence | 168 | 1104 | 68% | 1.00E-107 | 43.28% |
| CAIGJL010000001 | *Meloidogyne enterolobii* genome assembly, contig: Ment3s00001, whole genome shotgun sequence | 186 | 1129 | 76% | 7.00E-74 | 41.30% |
| CAIGJL010000034 | *Meloidogyne enterolobii* genome assembly, contig: Ment3s00034, whole genome shotgun sequence | 157 | 1125 | 60% | 4.00E-36 | 39.02% |
| CAIGJL010002411 | *Meloidogyne enterolobii* genome assembly, contig: Ment3s02411, whole genome shotgun sequence | 196 | 1139 | 75% | 2.00E-64 | 44.00% |
| RCFM01005457 | *Meloidogyne enterolobii* isolate L30 scaffold6107_cov164, whole genome shotgun sequence | 200 | 1113 | 62% | 2.00E-104 | 43.62% |
| RCFM01006500 | *Meloidogyne enterolobii* isolate L30 scaffold7310_cov168, whole genome shotgun sequence | 218 | 1087 | 71% | 4.00E-87 | 36.50% |
| RCFN01006666 | *Meloidogyne floridensis* isolate SJF1 scf7180000422736, whole genome shotgun sequence | 152 | 1128 | 58% | 2.00E-121 | 53.79% |
| NXFT01000985 | *Meloidogyne graminicola* strain IARI scaffold985, whole genome shotgun sequence | 154 | 1276 | 71% | 2.00E-40 | 52.59% |
| ABLG01001138 | *Meloidogyne hapla* strain VW9 Mh10g200708_Contig1137, whole genome shotgun sequence | 186 | 998 | 58% | 1.00E-60 | 41.60% |
| FXSY01000007 | *Meloidogyne incognita* genome assembly, contig: Meloidogyne_incognita_scaffold_7, whole genome shotgun sequence | 152 | 1121 | 58% | 2.00E-72 | 53.79% |
| FXSY01000995 | *Meloidogyne incognita* genome assembly, contig: Meloidogyne_incognita_scaffold_995, whole genome shotgun sequence | 128 | 1216 | 76% | 9.00E-76 | 41.88% |
| RCFL01003232 | *Meloidogyne incognita* isolate W1 scaffold9115_cov70, whole genome shotgun sequence | 128 | 1216 | 76% | 9.00E-76 | 41.88% |
| RCFL01003234 | *Meloidogyne incognita* isolate W1 scaffold9117_cov151, whole genome shotgun sequence | 152 | 1121 | 58% | 2.00E-72 | 53.79% |
| CABB01000157 | *Meloidogyne incognita*, whole genome shotgun sequence assembly, contig_157, strain Morelos | 152 | 1121 | 58% | 2.00E-72 | 53.79% |
| CEWN01002511 | *Meloidogyne javanica* genome assembly, contig: Meloidogyne_javanica_scaffold_2511, whole genome shotgun sequence | 168 | 1330 | 69% | 1.00E-107 | 43.28% |
| CEWN01004422 | *Meloidogyne javanica* genome assembly, contig: Meloidogyne_javanica_scaffold_4422, whole genome shotgun sequence | 121 | 1102 | 69% | 1.00E-32 | 37.09% |
| CEWN01000587 | *Meloidogyne javanica* genome assembly, contig: Meloidogyne_javanica_scaffold_587, whole genome shotgun sequence | 182 | 1142 | 75% | 2.00E-107 | 39.53% |
| RCFK01001432 | *Meloidogyne javanica* isolate VW4 scaffold1561_cov271, whole genome shotgun sequence | 235 | 1225 | 78% | 3.00E-123 | 45.30% |
| RCFK01001654 | *Meloidogyne javanica* isolate VW4 scaffold1811_cov234, whole genome shotgun sequence | 150 | 1170 | 70% | 3.00E-39 | 30.89% |
| CACSLI010000224 | *Meloidogyne luci* strain SI-Smartno genome assembly, contig: 000025F, whole genome shotgun sequence | 284 | 1024 | 73% | 6.00E-101 | 38.36% |
| CACSLI010000297 | *Meloidogyne luci* strain SI-Smartno genome assembly, contig: 000026F, whole genome shotgun sequence | 214 | 1108 | 69% | 3.00E-116 | 42.90% |
| CACSLI010000069 | *Meloidogyne luci* strain SI-Smartno genome assembly, contig: 000033F, whole genome shotgun sequence | 177 | 1209 | 75% | 3.00E-71 | 46.08% |

**Supplementary Table S2:** Characterisation of genomic contigs of *Meloidogyne* species identifying location of the coding sequence of DCRs in contigs, length of mRNA, number of exons, protein length, number of domains and putative designation of the variants of the encoded DCRs. ***** Ruler length refers to the number of amino acids located between the PAZ and the RNase IIIa domains.

| ***Meloidogyne* species** | **NCBI Accession number of contig containing putative coding sequence of DCRs** | **Coordinates of putative coding sequence of dicer in contig**  **[F=5’-3’ and R=3’-5’]** | **Length of mRNA of DCR (bases)** | **Number of exons in gene sequence** | **Number of amino acids of putative dicer** | **The “Ruler length”* (amino acids)** | **No. of protein domains** | **Designation of putative dicer protein based on identity and domain arrangement** |
| --- | --- | --- | --- | --- | --- | --- | --- | --- |
| *M. incognita* | CABB01000157 | 11869-20033 (R) | 5412 | 36 | 1803 | 331 | 7 | MiDCR-1.1 |
| *M. incognita* | FXSY01000007 | 74358-82522 (R) | 5412 | 36 | 1803 | 331 | 7 | MiDCR-1.1 |
| *M. incognita* | FXSY01000995 | 22567-30719 (F) | 5433 | 35 | 1810 | 330 | 7 | MiDCR-1.2 |
| *M. incognita* | RCFL01003234 | 2692-10856 (F) | 5364 | 35 | 1787 | 331 | 7 | MiDCR-1.3 |
| *M. incognita* | RCFL01003232 | 2681-10833 (F) | 5433 | 35 | 1810 | 330 | 7 | MiDCR-1.2 |
| *M. arenaria* | CEWM01000731 | 21522-29702 (F) | 5433 | 35 | 1810 | 330 | 7 | MaDCR-1.2 |
| *M. arenaria* | RCFJ01030829 | 14233-22389 (F) | 5376 | 35 | 1791 | 331 | 7 | MaDCR-1.4 |
| *M. arenaria* | QEUI01000369 | 13164-21301 (F) | 5448 | 35 | 1815 | 331 | 7 | MaDCR-1.1 |
| *M. arenaria* | CEWM01002306 | 15782-23972 (F) | 5457 | 35 | 1818 | 330 | 7 | MaDCR-1.5 |
| *M. arenaria* | QEUI01000193 | 109561-117673 (F) | 5397 | 35 | 1798 | 330 | 7 | MaDCR-1.3 |
| *M. javanica* | RCFK01001654 | 3656-11860 (R) | 5448 | 35 | 1815 | 331 | 7 | MjDCR-1.3 |
| *M. javanica* | CEWN01000587 | 14126-22295 (F) | 5445 | 35 | 1814 | 330 | 7 | MjDCR-1.1 |
| *M. javanica* | RCFK01001432 | 7622-15758 (R) | 5433 | 35 | 1810 | 330 | 7 | MjDCR-1.2 |
| *M. luci* | CACSLI010000297 | 1603444-1611631 (R) | 5421 | 36 | 1806 | 330 | 7 | MlDCR-1.3 |
| *M. luci* | CACSLI010000224 | 1290745-1298783 (R) | 5415 | 36 | 1804 | 332 | 7 | MlDCR-1.1 |
| *M. luci* | CACSLI010000069 | 1204356-1212503 (F) | 5433 | 35 | 1810 | 330 | 7 | MlDCR-1.2 |
| *M. enterolobii* | RCFM01005457 | 13623-21641 (F) | 5364 | 35 | 1787 | 331 | 7 | MeDCR-1.1 |
| *M. enterolobii* | RCFM01006500 | 15354-23452 (F) | 5403 | 36 | 1800 | 331 | 7 | MeDCR-1.2 |
| *M. graminicola* | NXFT01000985 | 15973-23643 (F) | 5328 | 35 | 1775 | 320 | 7 | MgDCR-1.1 |
| *M. hapla* | ABLG01001138 | 11738-19965 (R) | 5313 | 36 | 1770 | 320 | 7 | MhDCR-1.1 |
| *M. floridensis* | RCFN01006666 | 12550-20739 (R) | 5409 | 36 | 1802 | 330 | 7 | MfDCR-1.1 |

**Supplementary Table S3:** Summary of manual curation done to correct expected translation in six of the 21 genomic contigs of the eight *Meloidogyne spp.* before protein translation.

| **Contig ID** | **Problem - position on protein sequence** | **Reason** | **Changes made** |
| --- | --- | --- | --- |
| RCFM01005457-Me | 10 extra amino acids - (19-28) | Alternate splicing including 41 bp in the exon - “CCTA” missing in intron | Added “CCTA” in intron |
| RCFM01005457-Me | 9 AAs missing - 1241 | Alternate splicing dropping 25bp which also includes an “A” missing | Added “A” in exon |
| QEUI01000193-Ma | 37 amino acids additional (1800-1836) | “A” missing in a stretch of five “A”s (position 5394 on mRNA) | Added “A” in exon |
| QEUI01000193-Ma | 12 amino acids missing - 656 | “G” missing after 3 “G”s in a row resulting in splicing out 36 bp | “G” Added in exon |
| QEUI01000193-Ma | 17 amino acids missing - 577 | “A” missing in the exon resulting in splicing out 51 bp | “A” added in exon |
| QEUI01000193-Ma | 33 additional amino acids - 215 | Alternative splicing (two “A”s deleted, 1 each in exon and intron) | As added 1 each in exon and intron |
| QEUI01000369-Ma | 52 amino acids missing - 418 | Alternative splicing-156 bp spliced out (“A” missing in exon and “TA” missing in intron) | “A” added in exon and “TA” added in intron |
| QEUI01000369-Ma | 15 amino acids missing - 1041 | Alternate splicing -44 bp spliced out- “T” missing in exon | “T” added in exon |
| QEUI01000369-Ma | 11 amino acids missing - 1120 | Alternate splicing-42 bp spliced out- “C” missing in exon and “A” missing in intron | “C” added in exon and “A” added in intron |
| QEUI01000369-Ma | 5 amino acids missing, 18 translated differently -1295 | Alternate splicing on both ends-14 bp spliced out- “A” and “G” additional in intron, “G” missing in the following exon. | “A” and “G” deleted in the intron and “G” added in the exon |
| RCFJ01030829-Ma | 14 amino acids additional -278 | Alternate splicing- “A” missing in intron | “A” added in intron |
| CEWM01002306-Ma | 14 amino acids additional -257 | Alternate splicing- “A” missing in intron | “A” added in intron |
| CACSLI010000297-Ml | 14 amino acids additional -245 | Alternate splicing- “T” missing in intron | “T” added in intron (reverse strand) |

**Supplementary Data S1:** Twenty one mRNA sequences of DCR-1 from eight root-knot nematodes species (*M. arenaria, M. luci, M, incognita, M. enterolobii, M. javanica, M. hapla, M.* *floridensis* and *M. graminicola*) indicating corrections made after manual curation of genomic sequences. Contig IDs are followed by species name and number of exons in the mRNA in parenthesis. Red alphabets indicate addition of the bases and green alphabets indicate the bases that were deleted from the sequence before translating into proteins, grey text represents introns).

>CACSLI010000297-*M. luci* (36 exons)

ATGTCTCCACCGAAGGATTTTAGTGGTAAATGTATTCCTCCAAGAGATTATCAGTTTAAATTCAAGGTTGAATTACTTGATCGTGCAAAGATTCAAAACACGATTATTTCGCTCGGTACTGGGAGTGGAAAAACATTTGTGGCGGTTCTTTTAATAAAAGAATATTCTCAGAGATTGTTACATCAAAATGAAAAAGCTGTGTTTTTGGTTAATACTGTTGAACTTGTTGCCCAGCAAGCCGAGCATATTGAATTTCATTCAAGTTTGTCTGTCGCCCGTATTTCTGGCTCTACAATTAAAAGAAAATATGAGAGAAAAGAAGTTGAGAAGATTACTAACACCAATCAGGCTAGTCATGTGATTGTAATTACAGCTCAAGTGTTTCTTGATTTAATCAATCACGGCTTATTCGATTTCTCTTCTTTGGCCTTGTTGATTGTTGATGAATGCCATCATTGCTTGGGAGAACTGCATCCATATAGGCTGATTATGAATCATTACAAGAAACTGCAAGGTCAGCGACCACGCGTTTTGGGCCTTACAGCTTCAATTCTGAATAAAAAAGTGCCATCCTCTCGTATTGAGTGCACCGCTCAGCTGCTAGAACAAATAATGGATTCACAGATAGAAACTTCCAGCAATTATACACAGGAGCGCCTTCGTTCATTCATTGAAAAGAATGAAGATTTTCATTCAGAACTTGAAGTTGATCCGCGTCGCAAAATTTTTGAG/gtttttaccataaaattaaaatttatttatttttat**a**attaag/ACAATTTCCCGTTCATTTTCAACTCTTCAACAAGTTGGTAGCTGGGCAGCTCTAAAAGGTTTTATCCTTTGGCAAAAGAATTTACTTAAACATGTTGATGACCCAATAATTGGCAATAAACAAAAATGTATTTTAAGAATGGCTGAAACTGCTTTTAGAACTTGTTCAAAAGTTTTAAGTCACAAAATAAATCCGTTAAACAGCTATGACAAATTGGGTAACGTAAATAAATCTTATATCAGTGACAGAGTACGCAAATTGTTGGAAATATTAAAAAGTTATTCGCCTTCAAAACGTGAATTGTCTGGAATTAAAGATACACTTTTTGGATTAGTTTTTGTGAAAGAACGATTTATTGCCTTTATGATTAACAATTTGCTTCGTTTTCTTGTAAAACAAAATCCTGAAGAGTTTGGACATTTAAAAGTGGATTTTATTGTTGGACATACTGGAAATTCTGAAACAGGAGATGAAGATAGGCGTTTAGTAAATCGAAAGCAAGAGCAGACGCTTACACGATTCCGGAATGGGCAACTTAATTTATTAATAACAACAAATGTTTTGGAAGAAGGAATTGATCTGAGAAATTGTAATTTGGTTATTCGATTTGATCCACCAATGGATTTTCGGTCATTTATTCAATCTAGCGGTCGTGCTCGAAGAGAAAATAGCGCTTTTTATATACTTATTGAAGAGAAAAATTATTTGGATTTTATGATGGACTTAACAGGATATGCACAAGCAGAAGAGTTTGTGCTGCGACGTTATCGTTCTGGCAATGATTTTACTTTGGAAGGAAATGATGAAACAAAAATATTACATCCACATCTGGATGATGCTGTTGCTCCTTATGTTGTTACAACTGAAAAAGGGACAGCTAAAGTCTCGTTAAGTAATGCTATACAATTGGTGAACAGATACTGTGCAAAATTACCATCTGATATTTTTACTCGATTAGTCCCTCGTTATACGATACAAACTTTATCAGAAAATGGACAAACTTTGTATATTGCTGAATTATATCTTCCAATCAATTCACCAATAAAGGAGCCAATAAAATCTAAGCCGATGACTTCTAAAAGATTATCTTTAATGGCAGTCGCTTTGGAGGCTTGCAAACGATTACATCAAAGAAAAGAATTAAATGACAATCTTTTGCCTGCAGGAAAAGATATCTTGGATGATCTTTTAGGCGAAGTTGATGATGATGAATATCTGCCTCATTTACCAAGCAGAATGGGATCCTCAAAGAAAAAACGTCTCTATGATAGAAAGATGTCGAAAACTCTTAATTCTACATTACCTTCGCAAGACAGTGAATGCATTCTTTACGTTATGGAAATGAAATTGATTAAGCCTGTTACTGATGAAGGGAATCCAAAAAGGAGAAAAATTATTGATCCGTTTGAATCAAATTCGGCATTTGGATTTTTGTCTTCGAAGGAGCTCCCGAAGATTCCGGGATTTCCAGTTTTTCAACGAAATGGTGAAATGAATGTGCAAATTCGAAAAGTAAAAAATCAACAGTTTCGTCCCACTTTTGAACTTTTACAACTTATTTACCTCTTCCATCAACACATCTTTGAAGATATTTTGCGTGTTGCTCGTGGTGGTGTTGTTTTTGCACCTGGGCATTCTCCAATACCACTTTTAATTGTTCCTTTGAAAAAATTAAGCACTGCCGATTTGGACTATGAAATTGACAGAGATTACCTAAATTGGGATATCCGTGAATCTCCAACTACACCTTCAGATGAAATTAGAAAACAATTCTCTTTTGAAGAATCAAACTACTTAAATGCTGTTGTTTCACCTTGGTATCGTTCTGAAGATCAGTCTGCTTTTTATTACGTAGCTGAGATAATGACTGATCAATTTCCGTCTTCTTCATTTCCTGATGAAAAATTTACTTGTTTTAATCAATATTTTATGAGTAAATATCAATTGGAGATCTACAATCAAAAACAAAATTTACTTGATGTTGACCATACATCTGCTAGAATGAATCTTTTATTACCACGAGCAATTACTGGAAAATCAGCTACTCGTTCATTAGACCCGTCTCAACGTCAAATTCTTGTTCCAGAATTGGTACATATACATCCTCTTTCTGCAACTCTTTGGTCAATTATTGTTACTCTTCCTACAATTCTTTATAGACTTAATTCTTTACTGCTTGCTGATGAATTTCGTAGCAAAGTTCTTGAAGATGCTTTGAAAATTAATTCACAAACACCCGACGATTTTGAATGGAGTCCTCTTCAATATGTCACTCTAAATGATGATTTGACACAAAAAAGTATAAGAAATTTAGATCAGTTGAGAAAAATGAACAAACAAGAGAAGGAAAATGAAGTACCAATGGAGTGTGACATTATTGAGGAGAATGTGAATGAGACAGCTGCAAGTGGAATTGTGGGTAAATTAAAATATTTGAAATCAAATGATTTTGAAATCGGCGTTTGGGACCCTTCAGATGGCGCACAAATGTCTAGCAATGATAATACTCTGAATGAACCTTCCCCAGTCATTCATAATGCACCTGTAAATGGAATAATCCCTGGGCGGCGGAATGGTCTTCGAGGAGTTATAGCTGCTAGAGACGAGGAACTCTCTGAAATTATTGCTGTTGGTAATGATACAACACTTCATAATTATGGAGATATTTCTGATGATGATGATGTTGCAGCTGAATATGATAAATTTAAATTTTTGATGGACAATAAAATGACTACAAGTGATATTGGTGATCTAGGTGAAATGGATGTTCGACCTGCCGGGTGGAATGACGACAGCAATGCCAATGTTATCCAAATTGAAAACGAAAGTCTTCCATTGACAATATCGACAAACAATCCTCATATTAATATTGCAAGTTTAATGAATGATTTGGAGAAAAATTATGCTGCTTTTGGAACTTCAAACCATATTTCAAATAATAAAACGAATACACCGATCATTTCGAGTGATACTGCTGTTGACAAAACGGTCTTACAAAGAAAGGAGGTCCTCTTAACATATCTTATTAACAATTCGGTAAAAAGACAAAATGAAGATATTTTACCCGAGGAAGTTTTTTGGGGAATGGATGAAGCTGAGATAGGAGAGAATATTAGCAGTGAAGCATTGACAACTTCAAGTGCTGCGGATGGAATAAATTTGGAAAGGTTAGAAACAATAGGAGATTCCTTTCTCAAAATGGCTGTCACCAACTATTTCTACTATAAACACACTGAACAACATGAAGGAAAGTTAAGCTATGCTCGTTCAAAAGAAGTTTCGAACTCCCATCTTTTTTATCTTGGACGACAACGAGGCATCCCTCTTCTAATAGAAACACTGAAATTCGATCCACATGTTAACTGGCTTCCTCCTTGTTATGCTTCTACTTCAGAATTTCACGCTGTCAATCCTTTTGATTATACAGATTTAGATGAAGATCAATGTCAAGTGCCAATGGAAGGTGTTGATACTACTGAAACTGTAGATCAACAGCAAAAAATTAATAAAGAAACAATAGCAACTGGATGGGGAACTCTTGACGATGATAAACAAAATTATAAACGTGAAAATGGAGTAGAAACTTTAACTTTTCCACAACCAACAAAATCAGAAATTCCGGACTTACCTCCAATGCCATACAATATGCTTACTCAACAATGGATAAGTGACAAATCAATTGCTGATGCAGTAGAAGCATTAATTGGAGCTCATTTAATTCAACTTGGACAATCAGCTACTCTCAAATTTATGAATTGGTTGGGAATTAAGGTTCTAACTGATATTTCTTCATTGCCCTCACCACTTCTTCGTTTCATTGACACACCTGAAGATCCTAATCTTTCACTTAAACATCTTGCCCTTCTTTATGAGAAATTTGATTTTGCTACTGTTGAGAACAATATTGGCTATAAATTTGCAAATAAGGCATATTTAGTCCAAGCATTTACTCACGCATCATATTATAACAATCGAGTAACTGGTTGTTACCAACGTTTGGAATTCCTTGGTGATGCTGTTTTAGATTATATGATAACTCGTTTTCTTTATGAACACAAGAAGCAATATAGTCCGGGTGTTCTTACTGATCTTCGCTCAGCTCTTGTCAATAATACAATTTTTGCTTCGCTTGCAGTTAAATATTCTTTTCACAAACATTTTGTTATGATTTGTCCTCCACTGTATCAAATGGTCGAAAAGTTTGTTAATTTCTGTAAACAAAAAGATTTTCTTCATTGCGCGAATTTTGACGATGAAATATTTATGTTAACTGAAGATGAAATTGATGAGGAAGACCTTTTAAGTGAAGAAGATGTGGAAGTACCTAAAGCGATGGGTGATATTTTTGAATCAATTGCTGGTGCTGcAGATTGTGGAATGGATTTAGATATTGTTTGGCGTGTATTTTATAATTTAATGAGAGATGTTATTCAAAAATGTTGTGAAAATCCGCCACAATCGCCAGTTAGAGAATTATTTGAGAGAAAGAATTGTCGAGCAAAATTCTCGAAATTGGAACGAAAATTAGAAACAGGCAAAGTTAGAGTTACTGTAACGGTTAATGATAATCTCCAATTTACTGGTATGGGAAGGAGTTATCGAATTGCTAAATGTACAGCTGCTAAACGAGCACTTCAACATTTAAGAAAATTAGATGCCACAAAAACAGCAAAGAATAAATAA

>CEWM01002306-*M. arenaria* (35 exons)

ATGTCTCCACCGAAGGATTTTAGTGGTAAATGTATTCCTCCAAGAGATTATCAGTTTAAATTCAAGGTTGAATTACTTGATCGTGCAAAGATTCAAAACACGATTATTTCGCTCGGTACTGGGAGTGGAAAAACATTTGTGGCGGTTCTTTTAATAAAAGAATATTCTCAGAGATTGTTACATCAAAATGAAAAAGCTGTGTTTTTAGTTAATACTGTTGAACTTGTTGCCCAGCAAGCCGAGCACATTGAATTTCATTCAAGTCTGTCTGTCGCCCGTATTTCTGGCTCTACAATTAAAAGAAAATATGAGAGAAAAGAAGTTGAGAAAATTACTAGCAGCAATCAGGCAAGTCATGTATTATTAAAGTTTAGTTTATTTTTTGTTTTAAAGGTGATTGTAATTACAGCTCAAGTGTTTCTTGATTTAATCAATCACGGCTTATTCGATTTCTCTTCTTTGGCCTTGTTGATTGTTGATGAATGCCATCATTGCTTGGGAGAACTGCATCCATATAGGCTGATTATGAATCATTACAAGAAACTGCAAGGTCAACGACCACGCGTTTTGGGCCTTACAGCTTCAATTCTGAATAAAAAAGTGCCATCCTCTCGTATTGAGTGCACCGCTCAGCTGCTAGAACAAATAATGGATTCACAGATAGAAACTTCCAGCAATTATACACAGGAGCGCCTTCGTTCATTTATTGAGAAGAATGAAGATTTTCATTCAGAACTTGAAGTTGATCCGCGTCGCAAAATTTTTGAG/gtttttaccataaaattaaaatttatttatttttat**a**attaag/ACAATTTCCCGTTCATTTTCAACTCTTCAACAAGTTGGTAGCTGGGCAGCTCTAAAAGGTTTTATCCTTTGGCAAAAGAATTTACTTAAACATGTTGATGACCCAATAATTGGCAATAAACAAAAATGTATTTTAAGAATGGCTGAAACTGCTTTTAGAACTTGTTCAAAAGTTTTAAGTCACAAAATAAATCCGTTAAACAGCTATGACAAATTGGGTAACGTAAATAAATCTTATATCAGTGACAGAGTGCGCAAATTGTTGGAAATATTAAAAAGTTATTCGCCTTCAAAACGTGAATTCTCTGGAATTAAAGATACACTTTTTGGATTAGTTTTTGTGAAAGAACGATTTATTGCCTTTATGATTAACAATTTGCTTCGTTTTCTTGTAAAACAAAATCCTGAAGAGTTTGGACATTTAAAAGTGGATTTTATTGTTGGACATACTGGAAATTCTGAAACAGGAGATGAAGATAGGCGTTTAGTAAATCGAAAGCAAGAGCAGACGCTTACACGATTCCGGAATGGGCAACTTAATTTATTAATAACAACAAATGTTTTGGAAGAAGGAATTGATCTGAGAAATTGTAATTTGGTTATTCGATTTGATCCACCAATGGATTTTCGGTCATTTATTCAATCTAGCGGTCGTGCTCGAAGAGAAAATAGCGCTTTTTATATACTTATTGAAGAGAAAAATTATTTGGATTTTATGATGGACTTAACAGGATATGCACAAGCAGAAGAGTTTGTGCTGCGACGTTATCGTTCTGGCAATGATTTTACTTTGGAAGGAAATGATGAAACAAAAATATTACATCCACATCTGGATGATGCTGTTGCTCCTTATGTTGTTACAACTGAAAAAGGGACAGCTAAAGTCTCGTTAAGTAATGCTATACAATTGGTGAACAGATACTGTGCAAAATTACCATCTGATATTTTTACTCGATTAGTCCCTCGTTATACGATACAAACTTTATCAGAAAATGGACAAACTTTGTATATTGCTGAATTATATCTTCCAATCAATTCACCAATAAAGGAGCCAATAAAATCTAAGCCGATGACTTCTAAAAGATTATCTTTAATGGCAGTCGCTTTGGAGGCTTGCAAACGATTACATCAAAGAAAAGAATTAAATGACAATCTTTTGCCTGCAGGAAAAGATATCTTGGATGATCTTTTAGGCGAAGTTGATGATGATGAATATCTGCCTCATTTACCAAGCAGAATGGGATCCTCAAAGAAAAAACGTCTCTATGATAGAAAGATGTCGAAAACTCTTAATTCTACATTACCTTCGCAAGACAGTGAATGCATTCTTTACGTTATGGAAATGAAATTGATTAAGCCTGTTACTGATGAAGGGAATCCAAAAAGGAGAAAAATTATTGATCCGTTTGAATCAAATTCGGCATTTGGATTTTTGTCTTCGAAGGAGCTCCCGAAGATTCCGGGATTTCCAGTTTTTCAACGAAATGGTGAAATGAATGTGCAAATTCGAAAAGTAAAAAATCAACAGTTTCGTCCCACTTTTGAACTTTTACAACTTATTTACCTCTTCCATCAACACATCTTTGAAGATATTTTGCGTGTTGCTCGTGGTGGTGTTGTTTTTGCACCTGGGCATTCTCCAATACCACTTTTAATTGTTCCTTTGAAAAAATTAAGCACTGCCGATTTGGACTATGAAATTGACAGAGATTACCTAAATTGGGATATCCGTGAATCTCCAACTACACCTTCAGATGAAATTAGAAAACAATTTTCTTTTGAAGAATCAAACTACTTAAATGCTGTTGTTTCACCTTGGTATCGTTCTGAAGATCAGTCTGCTTTTTATTACGTAGCTGAGATAATGACTGATCAATTTCCGTCTTCTTCATTTCCTGATGAAAAATTTACTTGTTTTAATCAATATTTTATGAGTAAATATCAATTGGAGATCTACAATCAAAAACAAAATTTACTTGATGTTGACCATACATCTGCTAGAATGAATCTTTTATTACCACGAGCAATTACTGGAAAATCAGCTACTCGTTCATTAGACCCGTCTCAACGTCAAATTCTTGTTCCAGAATTGGTACATATACATCCTCTTTCTGCAACTCTTTGGTCAATTATTGTTACTCTTCCTACAATTCTTTATAGACTTAATTCTTTACTGCTTGCTGATGAATTTCGTAGCAAAGTTCTTGAAGATGCTTTAAAAATTAATTCACAAACACCCGACGATTTTGAATGGAGTCCTCTTCAATATGTCACTCTAAATGATGATTTGACACAAAAAAGTATAAGAAATTTAGATCAGTTGAGAAAAATGAACAAACAAGAGAAGGAAAATGAAGTACCAATGGAGTGTGACATTATTGAGGAGAATGTGAATGAGACAGCTGCAAGTGGAATTGTGGGTAAATTAAAATATTTGAAATCAAATGATTTTGAAATCGGCGTTTGGGACCCTTCAGATGGCGCACAAATGTCTAACAATGATAATACTCTGAATGAACCTTCCCCAGTCATTCATAATGCACCTGTAAATGGAATAATCCCTGGGCGGCGGAATGGTCTTCGAGGAGTTATAGCTGCTAGAGACGAGGAACTCTCTGAAATTATTGCTGTTGGTAATGATACAACACTTCATAATTATGGAGATATTTCTGATGATGATGATGTTGCAGCTGAATATGATAAATTTAAATTTTTGATGGACAATAAAATGACTACAAGTGATATTGGTGATCTAGGTGAAATGGATGTTCGACCTGCCGGGTGGAATGACGACAGCAATGCCAATGTTATCCAAATTGAAAACGAAAGTCTTCCATTGACAATATCGACAAACAATCCTCATATTAATATTGCAAGTTTAATGAATGATTTGGAGAAAAATTATGCTGCTTTTGGAACTTCAAACCATATTTCAAATAATAAAACGAATACACCGATCATTTCGAGTGATACTGCTGTTGACAAAACGGTCTTACAAAGAAAGGAGGTCCTCTTAACATATCTTATTAACAATTCGGTAAAAAGACAAAATGAAGATATTTTACCCGAGGAAGTTTTTTGGGGAATGGATGAAGCTGAGATAGGAGAAAATATTAGCAGTGAAGCATTGACAACTTCAAGTGCTGCGGATGGAATAAATTTGGAAAGGTTAGAAACAATAGGAGATTCCTTTCTCAAAATGGCTGTCACCAACTATTTCTACTATAAACACACTGAACAACATGAAGGAAAGTTAAGCTATGCTCGTTCAAAAGAAGTTTCGAATTCTCATCTTTTTTATCTTGGACGACAACGAGGCATCCCTCTTCTAATAGAAACACTGAAATTCGATCCACATGTTAACTGGCTTCCTCCTTGTTATGCTTCTACTTCAGAATTTCACGCTGTCAATCCTTTTGATTATACAGATTTAGATGAAGATCAATGTCAAGTGCCAATGGAAGGTGTTGATACTACTGAAACTGTAGATCAACAGCAAAAAATTAATAAAGAAACAATAGCAACTGGATGGGGAACTCTTGACGATGATAAACAAAATTATAAACGTGAAAATGGAGTAGAAACTTTAACTTTTCCACAACCAACAAAATCAGAAATTCCAGACTTACCTCCAATGCCATACAATATGCTTACTCAACAATGGATAAGTGACAAATCAATTGCTGATGCAGTAGAAGCATTAATTGGAGCTCATTTAATTCAACTTGGACAATCAGCTACTCTCAAATTTATGAATTGGTTGGGAATTAAGGTTCTAACTGACATTTCTTCATTGCCCTCACCACTTCTTCGTTTCATTGACACACCTGAAGATCCTAATCTTTCACTCAAACATCTTGCCCTTCTTTATGAGAAATTTGATTTTGCTACTGTTGAGAACAATATTGGCTATAAATTTGCAAATAAGGCATACTTAGTCCAAGCATTTACTCACGCTTCATATTATAATAATCGAGTAACCGGTTGTTACCAGCGTTTGGAATTCCTTGGCGATGCTGTTTTAGATTATATGATAACTCGTTTTCTTTATGAACACAAGAAGCAATATAGTCCGGGTGTTCTTACTGATCTTCGCTCCGCTCTTGTCAATAATACAATTTTTGCTTCGCTTGCAGTTAAATATTCTTTTCACAAACATTTTGTTATGATTTGTCCTCCACTTTATCAAATGGTCGAAAAGTTTGTTAATTTCTGTAAGCAAAAAGATTTTCTTCATTGCGCGAATTTTGACGATGAAATATTTATGTTAACTGAAGATGAAATCGATGAGGAAGACCTTTTAAGTGAAGAAGATGTCGAAGTACCTAAAGCAATGGGTGATATTTTTGAATCAATTGCTGGCGCTGTTTATTTAGATTGTGGAATGGATTTAGATATTGTTTGGCGTGTATTTTATAATTTAATGAGAGATGTTATACAAAAATGTTGTGAAAATCCGCCACAATCGCCAGTTAGAGAATTATTTGAGAGAAAGAATTGTCGAGCAAAATTCTCAAAATTGGAACGAAAATTAGAAACAGGCAAAGTTAGAGTTACTGTAACGGTTAATGATAATCTCCAATTTACTGGTATGGGAAGGAGTTATCGAATTGCTAAATGTACAGCTGCTAAACGAGCACTTCAACATTTAAGAAAATTAGATGCCACGAAAACAGCAAAGAATAAATAA

>RCFJ01030829- *M. arenaria* (35 exons) ATGTCTCCACCGAAGGATTTTAGTGGTAAATGTATTCCTCCAAGAGATTATCAGTTTAAATTCAAGGTTGAATTACTTGATCGTGCAAAGATTCAAAACACGATTATTTCGCTCGGTACTGGGAGTGGAAAAACATTTGTGGCGGTTCTTTTAATAAAAGAATATTCTCAGAGATTGTTACATCAAAATGAAAAAGCTGTGTTTTTGGTTAATACTGTTGAACTTGTTGCCCAGCAAGCCGAGCATATTGAATTTCATTCAAGTTTGTCTGTCGCCCGTATTTCTGGCTCTACAATTAAAAGAAAATATGAGAGAAAAGAAGTTGAGAAGATTACTAACACCAATCAGGCTAGTCATTTATTTGATTTCTCTTCTTTGGCCTTGTTGATTGTTGATGAATGCCATCATTGTTTGGGAGAACTGCATCCATATAGGCTGATTATGAATCATTACAAGAAACTTCAAGGTCAACGACCACGCGTTTTGGGCCTTACAGCTTCAATTCTGAATAAAAAAGTGCCATCCTCTCGTATTGAGTGCACCGCTCAGCTGCTAGAACAAATAATGGATTCACAGATAGAAACTTCCAGCAATTATACACAGGAGCGCCTTCGTTCATTTATTGAGAAGAATGAAGATTTTCATTCAGAACTTGAAGTTGATCCGCGTCGCAAAATTTTTGAG/gtttttaccataaaattaaaatttatttatttttat**a**attaag/ACAATTTCCCGTTCATTTTCAACTCTTCAACAAGTTGGTAGCTGGGCAGCTCTAAAAGGTTTTATCCTTTGGCAAAAGAATTTACTTAAACATGTTGATGACCCAATAATTGGCAATAAACAAAAATGTATTTTAAGAATGGCTGAAACTGCTTTTAGAACTTGTTCAAAAGTTTTAAGTCACAAAATAAATCCGTTAAACAGCTATGACAAATTGGGTAACGTAAATAAATCTTATATCAGTGACAGAGTGCGCAAATTGTTGGAAATATTAAAAAGTTATTCGCCTTCAAAACGTGAATTGTCTGGAATTAAAGATACACTTTTTGGATTAGTTTTTGTGAAAGAACGATTTATTGCCTTTATGATTAACAATTTGCTTCGTTTTCTTGTAAAACAAAATCCTGAAGAGTTTGGACATTTAAAAGTGGATTTTATTGTTGGACATACTGGAAATTCTGAAACAGGAGATGAAGATAGGCGTTTAGTAAATCGAAAGCAAGAGCAGACGCTTACACGATTCCGGAATGGGCAACTTAATTTATTAATAACAACAAATGTTTTGGAAGAAGGAATTGATCTGAGAAATTGTAATTTGGTTATTCGATTTGATCCACCAATGGATTTTCGGTCATTTATTCAATCTAGCGGTCGTGCTCGAAGAGAAAATAGCGCTTTTTATATACTTATTGAAGAGAAAAATTATTTGGATTTTATGATGGACTTAACAGGATATGCACAAGCAGAAGAGTTTGTGCTGCGACGTTATCGTTCTGGCAATGATTTTACTTTGGAAGGAAATGATGAAACAAAAATATTACATCCACATCTGGATGATGCTGTTGCTCCTTATGTTGTTACAACTGAAAAAGGGACAGCTAAAGTCTCGTTAAGTAATGCTATACAATTGGTGAACAGATACTGTGCAAAATTACCATCTGATATTTTTACTCGATTAGTCCCTCGTTATACGATACAAACTTTATCAGAAAATGGACAAACTTTGTATATTGCTGAATTATATCTTCCAATCAATTCACCAATAAAGGAGCCAATAAAATCTAAGCCGATGACTTCTAAAAGATTATCTTTAATGGCAGTCGCTTTGGAGGCTTGCAAACGATTACATCAAAGAAAAGAATTAAATGACAATCTTTTGCCTGCAGGAAAAGATATCTTGGATGATCTTTTAGGCGAAGTTGATGATGATGAATATCTGCCTCATTTACCAAGCAGAATGGGATCCTCAAAGAAAAAACGTCTCTATGATAGAAAGATGTCGAAAACTCTTAATTCTACATTACCTTCGCAAGACAGTGAATGCATTCTTTACGTTATGGAAATGAAATTGATTAAGCCTGTTACTGATGAAGGGAATCCAAAAAGGAGAAAAATTATTGATCCGTTTGAATCAAATTCGGCATTTGGATTTTTGTCTTCGAAGGAGCTCCCGAAGATTCCGGGATTTCCAGTTTTTCAACGAAATGGTGAAATGAATGTGCAAATTCGAAAAGTAAAAAATCAACAGTTTCGTCCCACTTTTGAACTTTTACAACTTATTTACCTCTTCCATCAACACATCTTTGAAGATATTTTGCGTGTTGCTCGTGGTGGTGTTGTTTTTGCACCTGGGCATTCTCCAATACCACTTTTAATTGTTCCTTTGAAAAAATTAAGCACTGCCGATTTGGACTATGAAATTGACAGAGATTACCTAAATTGGGATATCCGTGAATCTCCAACTACACCTTCAGATGAAATTAGAAAACAATTTTCTTTTGAAGAATCAAACTACTTAAATGCTGTTGTTTCACCTTGGTATCGTTCTGAAGATCAGTCTGCTTTTTATTACGTAGCTGAGATAATGACTGATCAATTTCCGTCTTCTTCATTTCCTGATGAAAAATTTACTTGTTTTAATCAATATTTTATGAGTAAATATCAATTGGAGATCTACAATCAAAAACAAAATTTACTTGATGTTGACCATACATCTGCTAGAATGAATCTTTTATTACCACGAGCAATTACTGGAAAATCAGCTACTCGTTCATTAGACCCATCTCAACGTCAAATTCTTGTTCCAGAATTGGTACATATACATCCTCTTTCTGCAACTCTTTGGTCAATTATTGTTACTCTTCCTACAATTCTTTATAGACTTAATTCTTTACTGCTTGCTGATGAATTTCGTAGCAAAGTTCTTGAAGATGCTTTAAAAATTAATTCACAAACACCCGACGATTTTGAATGGAGTCCTCTTCAATATGTCACTCTAAATGATGATCTGACACAAAAAAGTATAAGAAATTTAGATCAGTTGAGAAAAATGAACAAACAAGAGAAGGAAAATGAAGTACCAATGGAGTGTGAGAATATTGAGGAGAATGTGAATGAGACAGCTGCAAGTGGAATAGTGGGTGAATTAAAAAATTTTAGATCAAATGATTTTGAAATCGGCGTTTGGGACCCTTCAGATGGCGCACAAATGTCTAACAATGATAATACTCTGAATGAACCTTCCCCAGTCATTCATAATGCACCTGTAAATGGATTAATCCCTGGTCGGAGGAACGGTCTTCGAGGAGTTATAGCTGCTAGAGACGAGGAACTCTCTGAAATTATTGCTGTCGGGAATGATACAACACTTCATAATTATGGAGATATTTCTGATGATGATGATGTTGCAGCTGAATATGATAAATTTAAATTTTTGATGGACAATAAAATGACTACAAGTGATATTGGTGATCTCGGTGAAATGGATGTTCGACCTGCTGGGTGGAATGACGACAGTAATGCCAATGTTATCCAAATTGAAAACGAAAGTCTTCCCTTGACAATATCGACAAACAATCCTCATATTAATATTGCAAGTTTAATGAATGATTTGGAGAAAAATTATGCTGCTTTTGGTACTTCAAGCCATATTTCGAATAATACTAGAACGAATACACCGATCATTTCGAGTGATACTGCTGTTGACAAAACGGCCTTGCAAAGAAAGGAGGTCCTCTTAACATATCTTACTAACAATTCGGTAAAAAGGCAAAATGAAGATATTTTACCTGAGGAAGTTTTTTGGGGAATGGATGAAGCTGAGATAGGAGAAAATATTAGCAGTGAAGCATTGACAACTTCGAGTGCTGCGGATGGAATAAATTTGGAAAGATTAGAAACAATTGGAGATTCCTTTCTTAAAATGGCTGTCACCAACTATTTCTACTATAAACACACTGAACAACATGAAGGAAAGTTAAGCTATGCTCGTTCAAAAGAAGTTTCGAATTCTCATCTTTTTTATCTTGGACGGCAACGAGGCATCCCTCTTCTAATAGAAACACTTAAATTCGATCCACATGTTAACTGGCTTCCTCCTTGTTATGCTTCTACTTCAGAATTCCATGCTGTCAATCCTTTTGATTATACAGATTTAGATGAAGATCAATGTCAAGTGCCAATGGAAGGTGTTGATACTACTGAAACTTTAGATCAACAGCAAAAGACTAATAAAGAAACAATAGCAACTGGATGGGGTACTCTTGACGATGATAAACAAAATTATAAACGTGAAAATGGAGTAGAAACTTTAACTTTTCCACAACCAACAAAATCAGAAATTCCAGACTTACCTCCAATGCCATACAATATGCTTACTCAACAATGGATAAGTGACAAATCAATTGCTGATGCAGTAGAGGCATTAATTGGAGCACATTTAATTCAACTTGGACAATCAGCTACTCTCAAATTTATGAATTGGTTGGGAATTAAGGTTCTAACTGACATTTCTTCATTGCCCTCACCACTTCTACGTTTCATTGACACACCTGAAGATCCTAATCTTTCACTTAAACATCTTGCCCTTCTTTATGAGAAATTTGATTTTGCTACTGTTGAGAACAATATTGGCTATAAATTTGCAAATAAGGCATACTTAGTCCAAGCATTTACTCACGCTTCATATTATAATAATCGAGTAACCGGTTGTTACCAGCGTTTGGAATTCCTTGGCGATGCTGTTTTAGATTATATGATAACTCGTTTTCTTTATGAACACAAGAAGCAATATAGTCCGGGTGTTCTTACTGATCTTCGCTCCGCTCTTGTCAATAATACAATTTTTGCTTCGCTTGCAGTTAAATATTCTTTTCACAAACATTTTGTTATGATTTGTCCTCCACTTTATCAAATGGTCGAAAAGTTTGTTAATTTCTGTAAACAAAAAGATTTTCTTCATTGCGCGAATTTTGACGATGAAATATTTATGTTAACTGAAGATGAAATTGATGAGGAAGACCTTTTAAGTGAAGAAGATGTGGAAGTACCTAAAGCGATGGGTGATATTTTCGAATCAATTGCTGGTGCTGTTTATTTAGATTGTGGAATGGATTTAGATATTGTTTGGCGTGTATTCTATAATTTAATGAGAGATGTTATACAAAAATGTTGTGAAAATCCGCCACAATCGCCAGTTAGAGAATTATTTGAGAGAAAGAATTGTCGAGCAAAATTCTCAAAATTGGAACGAAAATTAGAAACAGGCAAAGTTAGAGTTACTGTAACGGTTAATGATAATCTCCAATTTACTGGTATGGGAAGGAGTTATCGAATTGCTAAATGTACAGCTGCTAAACGAGCACTTCAACATTTAAGAAAATTAGATGCCACGAAAACAGCAAAGAATAAATAA

>RCFM01005457-*M. enterolobii* (35 exons)

ATGTCTCCACCGAAGGATTTTAGTGCTAAATGTATTCCTCCAAGAGATTATCAGGT/attta**ccta**ttctacttttttgtctaatttactttaaatttaaggt/TGAATTGCTTGATCGTGCAAAAATTCAAAACACGATTATTTCGCTCGGTACTGGTAGTGGAAAAACATTTGTGGCGGTTCTTTTAATAAAGGAATATTCTCAGAGGTTGTTACATCAAAATGAAAAAGCTGTGTTTTTGGTTAATACTGTTGAACTTGTTGCCCAGCAAGCCGAGCACATTGAATTTCATTCAAGTTTGTCTGTCGCCCGTATTTCTGGTTCTACAATTAAAAGAAAATATGAGAGAAGAGAAGTTGAGAAAATTACTAGCAACAATCAGGCAAGTCATTTATTCGATTTCTCTTCTTTGGCTTTGTTGATTGTTGATGAATGCCATCATTGTTTGGGGGAACTGCATCCATATAGGCTGATTATGAATCATTACAAGAAACTGCAAGGTCAACGACCACGCGTTTTGGGCCTTACAGCTTCAATTCTGAATAAAAAAGTGCCGTCCTCTCGCATTGAATGCACCGCTCAGCTGCTAGAACAAATAATGGATTCGCAGATAGAAACCTCCAGCAATTATACACAGGAGCGCCTTCGTTCATTTATTGAAAAGAATGAAGATTTTCATTCAGAATTTGAAGTTGATCCGCGTCGCAAAATTTTTGAAACAATTTCCCGTTCATTTTCAACTCTTCAACAAGTTGGTAGCTGGGCAGCTCTAAAAGGGTTTATCCTTTGGCAAAAGAATTTACTTAAACATGTTGATGACCCGATAATTGGCAATAAACAAAAATGCATTTTAAGAATGGCTGAGACTGCTTTTAGAACTTGTTCAAAAGTTTTAAGTCACAAAATAAATCCATTAAACAGCTATGACAAATTGGGTAACGTAAATAAATCATATATCAGTGACAGAGTGCGCAAATTGCTGGAAATATTAAAAAGTTATTCGCCTTCAAAACGTGAATTGTCTGGAATTAAAGATACGCTTTTTGGATTAGTTTTTGTGAAAGAACGATTTATTGCCTTTATGATTAATAATTTACTTCGTTTTCTTGTAAAACAAAATCCTGAAGAGTTTGGACATTTAAAAGTGGACTTTATTGTTGGACACACGGGAAATTCTGAGACAGGAGATGAAGATAGGCGTCTGGTAAATCGAAAACAGGAACGAACTCTTACACAATTCCGGAATGGGCAACTGAATTTATTGATAACAACGAATGTTTTAGAAGAAGGCATTGATCTTAGAAACTGCAATTTAGTCATTCGATTTGATCCACCAATGGATTTTCGGTCATTTATTCAGTCTAGCGGTCGTGCTCGAAGAGAAAATAGTGCTTTCTATATACTTATTGAAGAGAAAAATTATTTGGATTTTATGATGGACTTAACTGGATATGCACAAGCAGAAGAGTTTGTTCTGCGACGTTATCGTTCTGGCAATGATTTCACTTTGGAAGGAAATGATGAAACAAAAATGATACAACCACATCTGGATGATGCTGTTGCTCCTTATGTTGTTACAACTGAAAAAGGGACAGCTAAAGTCTCATTAAGTAATGCTATACAATTGGTGAACAGATACTGTGCAAAATTACCATCTGATATTTTTACTCGATTAGTGCCACGTTATACAATACAAACTTTATCAGAAAATGGGCAAACTTTGTATATTGCCGAATTATATCTCCCAATCAATTCACCAATAAAGGAACCGATAAAATCTAAACCGATGAATTCTAAAAGATTATCTTTAATGGCAGTTGCTTTGGAGGCTTGCAAACGATTACATCAAAGAAAAGAATTAAATGACAATCTTTTGCCTGCAGGAAAAGATATCTTGGATGATCTTTTAGGTGAAGTAGATGATGACGAGTATCTACCTCATTTACCTAGCAGAATGGGATCCTCAAAGAAAAAACGTCTCTACGATAGAAAAATGTCGAAAACTCTTAATTCTACATTACCTTCGCAAGACAGTCAATGCATTCTTTACGTTATGGAAATGAAACTGGTTAAGCCCGTTACTGATGAAGGAAATCCAAAGAGGAGAAAAATTATTGATCCGTTCGAATCAAATTCGGCATTTGGATTTTTGTCTTCCAAGGAGCTCCCGAAGGTTCCGGGATTTCCAGTTTTTCAACGGAATGGTGAAATGCTTGTGCAAATTCGTAAAGTAAAAAATCAACAATTTCGTCCCACTTTCGAACTTTTACAACTCATTTATCTCTTTCATCAACACATTTTTGAAGATATTTTGCGTGTTGCTCGTGGTGGTGTTGTTTTTGCACCTGGTCATTCTCCAATACCACTTTTAATCGTTCCTTTAAAAAAATTAAGCACTGCCGATTTGGACTATGAAATTGACAAAGATTACCTAAATTGGAATATCCGTGAATCTCCAACTACACCTTCAGATGAAATTAGAAGACAATTTTCTTTTGAAGAATCAAACTACTTAAATGCTGTTGTTTCGCCTTGGTATCGTTCTGAAGATCAGTCTGCTTTTTATTACGTAGCTGAGATAATGACTGATCAATTTCCGTCTTCTTCATTTCCTGATGAAAAATTTACTTGTTTCAATCAATACTTTATGAGTAAATATCAATTGGAGATCTACAATCAAAAACAAAATTTACTTGATGTTGACCATACATCTGCTAGAATGAATCTTTTATTACCACGAGCAATTACTGGAAAATCAGCTACTCGTTCACTAGACCCATCTCAACGTCAAATTCTTGTTCCAGAATTGGTACATATACATCCTCTTTCTGCAACTCTTTGGTCAATTATTGTTACTCTCCCTACAATTCTTTATAGACTTAATTCTTTACTGCTTGCTGATGAATTTCGTAGCAAAGTTCTTGAAGATGCTTTGAAAGTTAATTCACAAACACCCGACGATTTTGAATGGAGTCCTCTTCAATATGTCACTTTAAATGATGATCTGACACAAAAAAGTATAAAAAATTTAGATCAGTTGAGAAAAATGAACAAACAAGAGAAGGAAAATGAAGTACCAATGGAGCTTGATATTATTGATGAGAATGTGAATGAGACAGCAGCTACTGGAATGGTGGGTGAATTAAAAATTTTGAGATCAAATGATTTCGAAATCGGTGTTTGGGACCCCTCAGATGGCACACAAATGTCTAACAATGATAATACTTTGAATGAACCTTCCCCAGTAATTCACAATGCGCCTGTAAATGGATTAATCCCTGGGCGGAGGAATGGTCTTCGAGGAGTTATAGCTGCTAGAGACGAGGAGCTCTCTGAAATCATTGCTGTCGGGAATGATACAACACTTCATAATTATGGAGATATTTCTGATGATGATGATGTTGCAGCTGAATATGATAAATTTAAATTTTTGATGGACAATAAAATGACTACAAGTGATATTGGTGATCTAGGTGAAATGGATGTTCGACCTGCTGGGTGGAATGACGATAGCAATGCCAATGTTATCCAAATTGAGAATGAAAGTCTTCCTTTGACAATATCGACAAATAATCCTCATATTAATATTGCAAGTTTAATGAATGATTTGGAGAAAAATTATGCTGCTTTTGGAACTTCAAGCCATATTTCCAATAATAATAAAACGAATACCTCGATCATTCCGAGTGCTACAGCTGTTGACAAAACGGTCTTGCAAAGAAAGGAGGTCCTCTTAACAT**A**TCTTATTAACAATTCAGTAAAAAGGCAAAATGAAGATATTTTACCTGAGGAAGTTTTTTGGGGAATGGATGAAGCTGAGATAGGAGAGAATATTAGCAGTGAAGCATTGACAACTTCAAGTGCTGCGGATGGAATAAATTTGGAAAGGTTAGAAACAATTGGAGATTCCTTTCTCAAAATGGCTGTCACCAACTATTTCTACTATAAACACACTGAACAACATGAAGGAAAGTTGAGCTATGCACGTTCTAAAGAAGTTTCGAATTCCCATCTTTTTTATCTTGGACGGCAACGAGGCATCCCTCTCCTAATAGAAACGCTGAAATTTGATCCACATGTTAACTGGCTTCCTCCTTGTTATGCTTCTACTTCAGAATTTCATGCTGTGAATCCTTTTGATTATACAGATTTAGATGAAGATCAATGTCAAGTGCCAATGGAGGGTGTTGGTACTACTGAAACTGTAGATCAACAGCAAAAGATTAATAAAGAAACAATAGCAACTGGATGGGGTACTCTTGACGATGATAAACAGAATTACAAACGTGAAAATGGAGTAGAAACTTTAACTTTTCCACAACCAACAAAATCAGAAATTCCGGACTTACCTCCAATGCCATACAATATGCTTACTCAACAATGGATAAGTGACAAATCAATTGCTGATGCGGTAGAGGCATTAATTGGAGCACATTTAATTCAACTTGGACAATCAGCTACTCTCAAATTTATGAATTGGTTGGGAATTAAGGTTTTAACTGATATTTCTTCATTGCCCTCACCACTTTTACGTTTCATTGACACACCTGAAGATCCTAATCTTTCACTTAAACATCTTGCCCTTCTTTATGAGAAATTTGATTTTGCCACTGTTGAGAACAACATTGGTTATAAATTTGCAAATAAGGCATATTTAGTCCAAGCATTTACTCACGCTTCATATTACAACAATCGAGTAACCGGTTGTTACCAACGTTTGGAATTCCTTGGCGATGCTGTTTTGGATTATATGATAACTCGTTTTCTTTATGAACACAAGAAGCAATATAGTCCGGGTGTTCTTACTGATCTTCGCTCCGCTCTTGTCAATAATACAATTTTTGCTTCGCTCGCAGTTAAATATTCTTTTCACAAACATTTTGTTATGATTTGTCCTCCACTCTATCAAATGGTCGAAAAGTTTGTTAATTTCTGTAAACAAAAAGATTTTCTTCATTGCGCGAATTTTGACGATGAAATATTTATGTTAACTGAAGATGAAATTGATGAGGAAGACCTTTTAAGTGAAGAGGATGTAGAGGTACCTAAAGCGATGGGTGATATTTTCGAATCAATTGCTGGTGCTGTTTATTTGGATTGTGGAATGAATTTAGATATTGTTTGGCGTGTATTTTATAATTTAATGAGAGATGTTATACAAAAATGTTGTGAAAATCCGCCACAATCGCCAGTTCGAGAATTATTTGAGAGAAAGAATTGTCGAGCTAAATTCTCAAAATTGGAACGAAAATTAGAAACAGGCAAAGTTAGAGTTACTGTAACGGTTAACGATAATCTCCAATTTACTGGGATGGGAAGGAGTTATCGAATTGCTAAATGTACAGCTGCTAAACGAGCTCTTCAACATTTAAGAAAATTAGATGCTGCAAAAACAGCAAAGAATAAATAA

>QEUI01000193- *M. arenaria* (35 exons) ATGTCTCCACCGAAGGATTTTAGTGGTAAATGTATTCCTCCAAGGGATTATCAGGTTGAATTGCTTGATCGTGCAAAAATTCAAAACACGATTATTTCGCTCGGTACTGGTAGTGGAAAAACGTTTGTGGCGGTTCTTTTAATAAAGGAATATTCTCAGAGATTGTTACATCAAAATGAAAAAGCTGTGTTTTTGGTTAATACTGTTGAACTTGTTGCCCAGCAAGCCGAGCACATTGAATTTCATTCAAGTTTGTCTGTCGCTCGTATTTCTGGCTCTACAATTAAAAGAAAATATGAGAGAAAAGAAGTTGAGAAAATTACTAGCAGCAATCAGGCAAGTCATGTATTATTAAAGTCTAGTTTATTTTTTGTTTTAAAGGTGATTGTAATTACAGCTCAATTATTCGATTTCTCTTCTTTGGCCTTGTTGATTGTTGATGAATGCCATCATTGTTTGGGAGAACTGCATCCATATAGGCTTATTATGAATCATTACAAGAAACTGCAAGGTCAACGACCACGCGTTTTGGGCCTTACAGCTTCAATTCTGAATAAAAAAGTGCCATCCTCTCGTATTGAGTGCACCGCTCAGCTGCTAGAACAAATAATGGATTCACAGATAGAGACCTCCAGCAATTATAC**A**CAGG/tagaatttttaaattgtatttt**a**tttaaaaattaattttatttaaagatatgcaaatatgttacaaaaccaaagcaatttattgtttgtacgaaggatgattgtacaaatgaaaaatttgtggttgatttattggtacgctcatgttatttagaatattaaattaagataattacacttaaattccaaaaattatgttgttcactttaatttttatagattcgattaataaataaaatcaattcattcttaatttaagg/AGCGCCTTCGTTCATTTATTGAAAAGAATGAAGATTTTCATTCAGAACTTGAAGTTGATCCGCGTCGCAAAATTTTTGAGACAATTTCCCGTTCATTTTCAACTCTTCAACAAGTTGGTAGCTGGGCAGCTCTAAAAGGTTTTATCCTTTGGCAAAAGAATTTACTTAAACATGTTGATGACCCAATAATTGGCAATAAACAAAAATGTATTTTAAGAATGGCTGAGACTGCTTTTAGAACTTGTTCAAAAGTTTTAAGTCACAAAATAAATCCGTTAAACAGCTATGACAAATTGGTTAACGTAAATAAATCATTTATCAGTGATAGAGTGCGCAAATTGTTGGAAATATTAAAAAGTTATTCACCCTCAAAACGTGAATTGTCTGGAATTAAAGATACACTTTTTGGATTAGTTTTTGTGAAAGAACGATTTATTGCCTTTATGATTAATAATTTGCTTCGGTTTCTTGTAAAACAAAATCCTGAAGAGTTTGGACATTTAAAAGTGGACTTTATTGTTGGACACACTGGAAATTCTGAGACAGGAGATGAAGATAGGCGTTTGGTGAATCGAAAACAAGAACAAACTCTTACAAAATTCCGAAATGGTCAATTAAATTTATTAATTACGACAAATGTTTTGGAAGAAGGAATTGATTTAAGAAATTGTAATTTAGTCGTTCGATTTGATCCGCCGATGGATTTTCGGTCATTTATTCAATCTAGCGGTCGTGCTCGAAGAGAAAATAGCGCTTTTTATATACTTATTGAAGAGAAAAATTATTTGGAGTTTATGATGGACTTAACAGGATATGCACAAGCAGAAGAGTTTGTTTTGCGGCGTTATCGTTCTGGCAATGATTTTACTCTGGAAGGAAATGATGAAACAAAATTATTACATCCACATCTGGATGATGCTGTTGCTCCTTATGTTGTTACAACTGAAAAAGGGACAGCTAAAGTCTCATTAAGTAATGCTATACAATATTGTGCAAAATTACCATCTGATATTTTTACTCGATTAGTGCCTCGTTATACA**A**TACAAACTTTATCAGAAAATGGACAAACTTTGTATATTGCTGAATTATACCTTCCAATCAATTCACCAATAAAGGAGCCAATAAAATCTAAGCCGATGACTTCTAAAAGATTATCTTTAATGGCAGTCGCTTTGGAGGCTTGCAAACGATTACATCAAAGAAAAGAATTAAATGACAATCTTTTGCCTGCAGGAAAAGATATCTTGGATGATCTTTTAGGG**G**AAGTTGATGATGATGAATATCTGCCTCATTTACCAAGCAGAATGGGATCCTCAAAGAAAAAACGTCTCTACGATAGAAAAATGTCGAAAACTCTTAATTCTACATTACCTTCGCAAGACAGTGAATGCGTTCTTTACGTTATGGAAATGAAACTGATTAAGCCCGTTACTGATGAAGGGAATCCGAAAAGGAGAAAAATTATTGATCCGTTCGAGTCAAATTCGGCGTTTGGCTTTTTGTCTTCGAAGGAGCTCCCGAAGGTTCCGGGATTTCCAGTTTTTCAACGGAATGGTGAAATGAATGTGCAAATTCGTAAAGTAAAGAATCAGCAATTTCGTCCCACTTTTGAACTTTTACAACTTATTTACCTCTTTCATCAACACATCTTTGAAGATATTTTGCGTGTTGCCCGTGGTGGTGTTGTTTTTGCACCTGGTCATTCTCCAATACCACTTTTAATTGTTCCCTTGAAAAAATTAAGCACTGCCGATTTGGATTATGAAATTGACAGAGATTACCTAAATTGGAATATCCGAGAATCTCCAACTACACCTTCAGATGAAATTAGAAAACAATTTTCTTTTGAAGAATCAAACTACTTAAATGCTGTTGTTTCACCTTGGTATCGTTCTGAAGATCAGTCTGCTTTTTATTACATAATGACTGATCAATTTCCGTCTTCTTCATTTCCTGATGAAAAATTTGCTTGTTTTAATCAATACTTTATGAGTAAATATCAATTGGAGATCTACAATCAAAAACAAAATTTACTTGATGTTGACCATACATCTGCTAGAATGAATCTTTTATTACCACGAGCAATTTCTGGAAAATCAGCTACTCGATCATTGGACCCATCTCAACGTCAAATTCTTGTCCCAGAATTGGTACATATACATCCTCTTTCTGCAACTCTTTGGTCAATTATTGTTACTCTTCCTACAATTCTTTATAGACTTAATTCTTTACTGCTTGCTGATGAATTTCGTAGCAAAGTTCTTGAAGACGCTTTGAAAATTAATTCACAAACACCCGACGATTTTGAATGGAGTCCTCTTCAATATGTCACTCTAAATGATGATCTGACACAAAAAAGTATAAGAAATTTAGATCAGTTGAGAAAAATGAACAAACAAGAGAAGGAAAATGAAGTACCAATGGAGTGTGACATTATTGAAGAGAATGTGAATGAGACAGCAGCAAGTGGAATAGTGGGTAGATTAAAATTTTTGAAATCAAATGATTTTGAAATCGGCGTTTGGGACCCTTCAGATGGCGCACAAATGTCTAACAATGATAATACTCCGAATGAACCTTCTCCAGTCATCCATAATGCACCTGTAAATGGATTAATCCCTGGGCGGAGGAATGGTCTTCGAGGAGTTATAGCTGCTAGAGACGAGGAACTCTCAGAAATTATTGCTGTTGGTAATGATACAACGCTTCATAATTATGGAGATATTTCTGATGATGATGATGTTGCAGCTGAATACGATAAATTTAAATTTTTGATGGACAATAAAATGACTACAAGTGATATTGGTGATCTAGGTGAAATGGATGTTCGACCTGCTGGTTGGAATGACGATAGCAATGCCAATGTTATCCAAATTGAAAATGAAAGTCTTCCCTTGACAATATCGACAAACAATCCTCATATTAATATTGCAAGTTTAATGAATGATTTGGAGAAAAATTATGCTGCTTTTGGTACTTCAAACCATATTTCCAATAATAATAAAACGAATACACCGAACATTCCGAATGCTACTGCTGACAAAACGGACTTGCAAAAAAAGGAGGTCCTCTTAACATATCTTATTAACAATTCGGTAAAAAGGCAAAATGAAGATATTTTACCTGAGGAAGTTTTTTGGGGAATGGATGAAGCTGAGATAGGAGAGAATATTAGCAGTGAAGCATTGACAACTTCAAGTGCTGCGGATGGAATAAATTTGGAAAGGTTGGAAACAATTGGAGATTCTTTCCTCAAGATGGCTGTCACCAACTATTTCTACTATAAACACACGGAACAACATGAAGGAAAGTTAAGCTATGCACGTTCAAAAGAAGTTTCGAATTCCCATCTTTTTTATCTTGGACGGCAACGAGGAATCCCTCTTTTAATAGAAACACTGAAATTCGATCCACATGTTAACTGGCTTCCTCCTTGTTATGCTTCTACTTCAGAATTCCATGCTGTCAATCCTTTTGATTATACAGATTTAGATGAAGATCAATGTGCAGTGCCAATGGAGGGTGTTGATACTACTGAAACTGTAGATCAACAGCAAAAGATTAATAAAGAAACAATAGCAACTGGATGGGGAACTATTGACGATGATAAACAAAATTATAAACGTGAAAATGGAGTAGAAACTTTAACTTTTCCACAACCAACAAAATCAGAAATTCCGGACTTACCTCCAATGCCATACAATATGCTTACTCAACAATGGATAAGTGACAAATCGATTGCTGATGCAGTAGAGGCATTAATTGGAGCACATTTAATTCAACTTGGACAATCAGCTACTCTGAAATTTATGAATTGGTTGGGAATTAAGGTTCTGACTGATATTTCTTCATTGCCCTCACCACTTCTTCGTTTCATTGACACACCTGAAGATCCTAATCTTTCACTTAAACATCTTGCCCTTCTTTATGAGAAATTTGATTTTGCCACTGTTGAGAACAATATTGGTTATAAATTTGCAAATAAGGCATATTTAGTCCAAGCATTTACTCACGCTTCATATTATAACAATCGAGTAACCGGTTGTTACCAACGTTTGGAATTCCTTGGCGATGCTGTTTTAGATTATATGATAACTCGTTTTCTTTATGAACATAAGAAGCAATATAGTCCTGGTGTTCTTACTGATCTTCGCTCCGCTCTTGTCAATAATACAATTTTTGCTTCGCTTGCAGTTAAATATTCTTTTCACAAACATTTTGTTATGATTTGTCCTCCACTTTATCAAATGGTCGAAAAGTTTGTTAATTTCTGTAAGCAAAAAGATTTTCTTCATTGCGCGAATTTTGACGATGAAATATTTATGTTAACTGAAGATGAAATCGATGAGGAAGACCTTTTAAGTGAAGAAGATGTAGAAGTACCTAAAGCGATGGGTGATATTTTCGAATCAATTGCTGGTGCTGTTTATTTAGATTGTGGAATGGATTTAGATATTGTTTGGCGTGTATTTTATAATTTAATGAGAGATGTTATACAAAAATGTTGTGAAAATCCACCACAATCGCCAGTTAGAGAATTATTTGAGAGAAAGAATTGTCGAGCAAAATTTTCAAAATTGGAACGAAAATTAGAAACAGGCAAAGTTAGAGTTACTGTAACGGTTAATGATAATCTCCAATTTACTGGTATGGGAAGGAGTTATCGAATTGCTAAATGTACAGCTGCTAAACGAGCACTACAACATTTAAGAAAATTAGATGCCAC**A**AAAACAGCAAAGAATAAATAA

>QEUI01000369- *M. arenaria* (35 exons)

ATGTCTCCACCGAAGGATTTTAGTGGTAAATGTATTCCTCCAAGAGATTATCAGTTTAAATTCAAGGTTGAATTACTTGATCGTGCAAAGATTCAAAACACGATTATTTCGCTCGGTACTGGGAGTGGAAAAACATTTGTGGCGGTTCTTTTAATAAAAGAATATTCTCAGAGATTGTTACATCAAAATGAAAAAGCTGTGTTTTTAGTTAATACTGTTGAACTTGTTGCCCAGCAAGCCGAGCACATTGAATTTCATTCAAGTCTGTCTGTCGCCCGTATTTCTGGCTCTACAATTAAAAGAAAATATGAGAGAAAAGAAGTTGAGAAAATTACTAGCAGCAATCAGGCAAGTCATGTATTATTAAAGTTTAGTTTATTTTTTGTTTTAAAGGTGATTGTAATTACAGCTCAAGTGTTTCTTGATTTAATCAATCACGGCTTATTCGATTTCTCTTCTTTGGCCTTGTTGATTGTTGATGAATGCCATCATTGCTTGGGAGAACTGCATCCATATAGGCTGATTATGAATCATTACAAGAAACTGCAAGGTCAACGACCACGCGTTTTGGGCCTTACAGCTTCAATTCTGAATAAAAAAGTGCCATCCTCTCGTATTGAGTGCACCGCTCAGCTGCTAGAACAAATAATGGATTCACAGATAGAAACTTCCAGCAATTATACACAGGAGCGCCTTCGTTCATTTATTGAAAAGAATGAAGATTTTCATTCAGAACTTGAAGTTGATCCGCGTCGCAAAATTTTTGAGACAATTTCCCGTTCATTTTCAACTCTTCAACAAGTTGGTAGCTGGGCAGCTCTAAAAGGTTTTATCCTTTGGCAAAAGAATTTACTTAAACATGTTGATGATCCAATAATTGGCAATAAACAAAAATGTATTTTAAGAATGGCTGAAACTGCTTTTAGAACTTGCTCAAAAGTTTTAAGTCACAAAATAAATCCGTTAAACAGCTATGACAAATTGAGTAACGTAAATAAATCATTTATCAGTGACAGAGTGCGCAAATTGTTGGAAATATTAAAAAGTTATTCGCCCTCGAAACGTGAATTGTCTGGAATTAAAGATACACTTTTTGGATTAGTTTTTGTGAAAGAACGATTTATTGCCTTTATGATTAATAATTTGCTTCGGTTTCTTGTAAAACAAAATCCTGAAGAGTTTGGACATTTAAAAGTGGACTTTATTGTTGGACATACTGGTAATTCTGAGACAGGAGATGAAGATAGGCGTTTGGTGAATCGAAAACAAGAACAAACTCTTACAAAATTCCGAAATGGCCAATTAA**A**TTTATTAATTACGACAAATGTTTTGGAAGAAGGAATTGATTTAAGAAATTGTAATTTAGTCGTTCGATTTGATCCGCCGATGGACTTTCGTTCATTTATTCAA/gtaaattcttaaatcattaaactaaataaaataatgttta**ta**g/TCTAGCGGTCGTGCTCGAAGAGAAAATAGCGCTTTTTATATACTTATTGAAGAGAAAAATTATTTGGATTTTATGATGGACTTAACAGGATATGCACAAGCAGAAGAGTTTGTTTTGCGGCGTTATCGTTCTGGCAATGATTTTACTTTGGAAGGAACTGATGAAACAAAGATAATACATCCACATCTGGATGATGCTGTTGCTCCTTATGTTGTTACAACTGAAAAAGGGACAGCTAAAGTCTCATTAAGTAATGCTATACAATACTGTGCAAAATTACCATCTGATATTTTTACTCGACTAGTGCCACGTTATACAATACAAACTTTATCAGAAAATGGACAAACTTTGTATATTGCCGAATTATATCTTCCAATCAATTCACCAATAAAGGAGCCAATAAAATCTAAACCGATGAATTCTAAAAGATTATCTTTAATGGCAGTCGCTTTGGAGGCTTGCAAACGATTACATCAAAGAAAAGAATTAAATGACAATCTTTTGCCTGCAGGAAAAGATATCTTGGATGATCTTTTAGGTGAAGTAGATGATGACGAATATCTGCCACATTTACCAAGCAGAATGGGATCCTCAAAGAAAAAACGTCTCTATGATAGAAAAATGTCGAAAACTCTTAATTCTACATTACCTTCGCAAGACAGTGCATGCTTTCTTTACGTTATGGAAATGAAACTGATTAAGCCCGTTACAGATGAAGGGAATCCGAAAAGGAGAAAAATTATTGATCCGTTCGAGTCAAATTCGGCGTTTGGCTTTTTGTCTTCGAAGGAGCTCCCGAAGGTTCAGGGATTTCCAGTTTTTCAACGGAATGGTGAAATGAATGTGCAAATTCGGAAAGTAAAGAATCAACAATTCCGTCCCAATTTTGAACTTTTACAACTTATTTACCTCTTTCATCAACACATCTTTGAAGATATTTTGCGTGTTGCTCGTGGTGGTGTTGTTTTTGCACCTGGTCATTCTCCAATACCACTTTTAATTGTTCCTTTGAAAAAATTAAGCACTGCCGATTTGGATTATGAAATTGACAAAGATTACCTAAATTGGGATATTCGTGAATCTCCAACTACACCTTCAGATGAAATTAGAAAACAATTTTCTTTTGAAGAATCAAACTACTTAAATGCTGTTGTTTCACCTTGGTATCGTTCTGAAGATCAGTCTGCTTTTTATTACGTAGCTGAGATAATGACTGATCAATTTCCGTCTTCTTCATTTCCTGATGAAAAATTTACTTGTTTTAATCAATATTTTATGAGTAAATATCAATTGGAGATCTACAATCAAAAACAAAATTTACTTGATGTTGACCATACATCTGCTAGAATGAATCTTTTATTACCACGAGCAATTACTGGAAAATCAGCTACTCGTTCATTAGACCCATCTCAACGTCAAATTCTTGTTCCAGAATTGGTACATATACATCCTCTTTCTGCAACTCTTTGGTCAATTATTGTTACTCTTCCTACAATTCTTTATAGACTTAATTCTTTACTGCTTGCTGATGAATTTCGTAGCAAAGTTCTTGAAGATGCTTTAAAAATTAATTCACAAACACCCGACGATTTTGAATGGAGTCCTCTTCGATATGTCACTCTAAATGATGATCTGACACAAAAAAGTATAAGAAATTTAGATCAGTTGAGAAAAATGAACAAACAAGAGAAGGAAAATGAAGTACCAATGGAGTGTGAGAATATTGAGGAGAATGTGAATGAGACAGCTGCAAGTGGAATAGTGGGTGAATTAAAAAATTTTAGATCAAATGATTTTGAAATCGGCGTTTGGGACCCTTCAGATGGCGCACAAATGTCTAACAATGATAATACTCTGAATGAACCTTC**T**CCAGTCATTCATAATGCACCTGTAAATGGATTAATCCCTGGTCGGAGGAACGGTCTTCGAGGAGTTATAGCTGCTAGAGACGAGGAACTCTCTGAAATTATTGCTGTCGGGAATGATACAACACTTCATAATTATGGAGATATTTCTGATGATGATGATGTTGCAGCTGAATATGATAAATTTAAATTTTTGATGGACAATAAAATGACTACAAGTGATATTGGTGATCTCGGTGAAATGGATGTTCGACCTGCTGGGTGGAATGACGACAGTAATGCCAATGTTATCCAAATTGAAAACGAAAGTCTTCC**C**TTG/gtaatataaacttaacgat**a**taaaaatttcttttacag/ACAATATCGACAAACAATCCTCATATTAATATTGCAAGTTTAATGAATGATTTGGAGAAAAATTATGCTGCTTTTGGTACTTCAAGCCATATTTCGAATAATACTAGAACGAATACACCGATCATTTCGAGTGATACTGCTGTTGACAAAACGGCCTTGCAAAGAAAGGAGGTCCTCTTAACATATCTTACTAACAATTCGGTAAAAAGGCAAAATGAAGATATTTTACCTGAGGAAGTTTTTTGGGGAATGGATGAAGCTGAGATAGGAGAAAATATTAGCAGTGAAG/gtgttaatattctcttctgacaaattctgattatttt**a**tatttaagaaac**g**aataaaatcatccaaacaaaaagtgccgattctattagccctacacgtgatttgagagatcttgaaaaacaagtgataaaatataatttaaatattaaaataattattttataattctaaactatttttatatttcttaaattttattttagaactttaaaataatggcacctgaacttaaatggatgcctttttcttttatgtggaacttacttgatcagaatccccatggtgtttcaccagctctactgcttcaaggtatattgttgaactgttactaaaattattttttattttag/CATTGACAACTTCGAGTGCTGCGGATGGAATAAATTTGGAAAGATTAGAAACAATTGGAGATTCCTTTCTAAA**G**ATGGCTGTCACCAACTATTTCTACTATAAACACACTGAACAACATGAAGGAAAGTTAAGCTATGCTCGTTCAAAAGAAGTTTCGAATTCTCATCTTTTTTATCTTGGACGGCAACGAGGCATCCCTCTTCTAATAGAAACACTTAAATTCGATCCACATGTTAACTGGCTTCCTCCTTGTTATGCTTCTACTTCAGAATTCCATGCTGTCAATCCTTTTGATTATACAGATTTAGATGAAGATCAATGTCAAGTGCCAATGGAAGGTGTTGATACTACTGAAACTTTAGATCAACAGCAAAAGACTAATAAAGAAACAATAGCAACTGGATGGGGTACTCTTGACGATGATAAACAAAATTATAAACGTGAAAATGGAGTAGAAACTTTAACTTTTCCACAACCAACAAAATCAGAAATTCCAGACTTACCTCCAATGCCATACAATATGCTTACTCAACAATGGATAAGTGACAAATCAATTGCTGATGCAGTAGAGGCATTAATTGGAGCACATTTAATTCAACTTGGACAATCAGCTACTCTCAAATTTATGAATTGGTTGGGAATTAAGGTTCTAACTGACATTTCTTCATTGCCCTCACCACTTCTACGTTTCATTGACACACCTGAAGATCCTAATCTTTCACTTAAACATCTTGCCCTTCTTTATGAGAAATTTGATTTTGCTACTGTTGAGAACAATATTGGCTATAAATTTGCAAATAAGGCATACTTAGTCCAAGCATTTACTCACGCTTCATATTATAATAATCGAGTAACCGGTTGTTACCAGCGTTTGGAATTCCTTGGCGATGCTGTTTTAGATTATATGATAACTCGTTTTCTTTATGAACACAAGAAGCAATATAGTCCGGGTGTTCTTACTGATCTTCGCTCCGCTCTTGTCAATAATACAATTTTTGCTTCGCTTGCAGTTAAATATTCTTTTCACAAACATTTTGTTATGATTTGTCCTCCACTTTATCAAATGGTCGAAAAGTTTGTTAATTTCTGTAAGCAAAAAGATTTTCTTCATTGCGCGAATTTTGACGATGAAATATTTATGTTAACTGAAGATGAAATCGATGAGGAAGACCTTTTAAGTGAAGAAGATGTCGAAGTACCTAAAGCAATGGGTGATATTTTTGAATCAATTGCTGGCGCTGTTTATTTAGATTGTGGAATGGATTTAGATATTGTTTGGCGTGTATTTTATAATTTAATGAGAGATGTTATACAAAAATGTTGTGAAAATCCACCACAATCGCCAGTTAGAGAATTATTTGAAAGGAAGAATTGTCGAGCAAAATTCTCAAAATTGGAACGAAAATTAGAAACAGGCAAAGTTAGAGTTACTGTAACGGTTAATGATAATCTCCAATTTACTGGTATGGGACGTAGTTATCGAATTGCTAAATGTACAGCTGCTAAACGAGCACTTCAACATTTAAGAAAATTAGATGCCACAAAAACAGCAAGGAATAAATAA

>ABLG01001138-*M. hapla* (36 exons)

ATGTCTCCACCGAAAGATTTTAGTGGTAAATGTATTCCTCCAAGAGATTATCAGGTTGAATTGCTCGATCGTGCCAAAATTCAAAATACAATTATTTCACTCGGCACTGGTGCAGGAAAAACATTTGTGGCTGTACTTTTAATTAAAGAATATGCTCAACGTTTGCTTCATCGAAATGAAAAAGCTGCGTTTTTGGTTAATACTGTTGAACTTGTTGCTCAACAAGCTGAACACATTGAATTTCATTCAAGTTTGTCTGTCGCTCGTATTTCCGGTTCTACAATTAAAAGAAAACACGAGAGAGGGGAAGTTGAGAAAATCACTCGTAACAATCAGGCAAGTATTGTGATAGTAATTACTGCACAAGTGTTTCTTGACTTCATAAATCATGGATTATTCGACTTCTCTTCTTTGGCTGTGTTGATTGTTGATGAATGCCATCATTGCTTGGGAGAACTTCATCCATACAGGCTGATTATGAATCATTACAGGAAATTACCGGGGCAACGTCCACGCGTTTTGGGCCTTACAGCCTCAATTCTGAATAAGAAAGTGCCATTCTCTCGCATTGAGTGTACTGCGCAACTATTAGAACAAATAATGGATTCGCATATAGAGACTACCAGTAATTATACCCAGGAGCGTCTTCGTTCATTTATTGAAAAGAATGAGGATTTTCATTCGGAACTCGAAGTTGATCCACGTCGCCCAATTTTTGAGAAGAAATTACTTAAACTTGTTGATGACCCAATACTTGGCACTAAACAGAAATGTATTTTAAGAATGGCTGAGACTGCTTTTAGAACTTGTTCAAAAGTTTTAAGTCACAAAATAAATCCATTAAACACTTATGATAAATTGGCAAACCCTAAAAAATCATACCTCAGCGATAGAATGCGCAAATTAGTGGAAATATTAAAAAGTTATGCTCCATCAAAACGTGAAAAGTCTGGAATAAAAGATACTCTTTTTGGATTAATTTTTGTTAAAGAAAGATTTATTGCTTTTATGATTAATAATTTACTTCGTTTTCTTGTAAAGCAAAATCCTGAAGACTTTGGCCATCTGAAAGTGGACTTTCTTGTTGGACAAACTGGAAATTCTGAAACTGGAGATGAAGATAGACGTTTAGCTGCTCGCAAACAAGAGCAAACTCTCTGTCGTTTTCGAAATGGTCAATTAAATTTGTTGGTTACTACAAATGTTTTAGAAGAAGGAATTGACTTGAGAAATTGTAATTTAGTTATTCGATTTGATCCACCGATGGATTTTCGTTCTTTTGTTCAGTCTAGTGGTCGTGCTCGAAAAGAAAATAGTGCTTTTTATATGCTTATCGAAGAGAAAAATTATTTGGAATTTATGATGGACTTAACTGGATATGCACAAGCAGAAGAGCTTGTTCTTCGACGTTATCGTTCGGGCAATGATTTTACTTTGAATGGTAAAGAGGAAGGGAATAATGAAACAAAAATTATACAACCACATCTCGATGATGTTGTTGCTCCTTATGTTGTTATAACTCCAAATGGAACAGCTAAGGTTTCGTTAAGTGGTGCTATACATTTGGTGAATAGATATTGTTCGAAATTACCATCTGATATTTTTACTCGATTGGTGCCACGTTATACAATAAAAACAATATCAGAAAATGGACAAACTTTGTATATTGCTGAATTATATCTCCCAATCAATTCTCCAATAAAAGAGCCAATAACATCTAAACCAATGGCTTCAAAAAGATTATCTTTAATGACAGCCGCATTGGAGGCTTGTAAACGATTACATCAAAGAAAAGAATTAAATGACCAGCTTTTGCCGGCAGGAAAGGAAATTGTTTTGGATCTTCTAGGTGAAGTAGACGATGATGAATATTTACCTTATTTACCCAGCAAGATGGGATCATCAAAGAAAAAACGACTTTACGATAGAAAAATGTCTAAAACCCTCTCTTCAACATTACCTGCACAAGAAAGTGAATGTATTCTTTATGTTATGGAAATGAAACTGATCAAGCCCGTTAGCGAGGAACGAAATCCGAAAAGAAGAAAAATTATTGATCCATTCGAATCAAATTCTGCATTTGGATTTTTATCTTCAAAGGAGCTTCCAAAAGTTCCCGGATTTCCAGTCTTTCAACGAAATGGTGAAATGATTGTACAAATTCGTAAAGCTAAAAATCAACCTGTTCGTCTCACATTTGAACTTTTCCAACTCATTTGTCTCTTCCATCAGCATATATTTGAAGATATTTTACGCATTGCTCGTGGTGGTGTTGTTTTTGCGCCTGGTCATTCTCCAATACCATTATTAATTGTTCCATTGAAAAAACTTGGAACTGTCGATCTGGACTATGAAATTGACAGAGATTACCTCAATTGGGATATCCGTGAACCTCCAACTACACCTTCTGATGAAATTAGAAAACAATACGTCTTTGAAGAGTCGAGCTATTTAAATGCTGTTGTTTCACCATGGTATCGTTCTGAGGATCAATCTGCTTTTTATTACGTAGCAGAGATTATGACAGATCAGTTTCCGTCGTCTTCATTTCCTGATGAGAAATTTACTTGTTTCAATCAATACTTTATGAGTAAATATCAGTTGGAAATTTACAATCAAAAGCAAAATTTACTTGATGTTGACCATACATCAGCTAGAATGAATCTTTTATTACCAAGAGCAATAACAGGAAAATCAGCACTTCGTTCATTAGACCCATCTCAACGCCAAATTCTTGTTCCAGAGCTGCTACATATTCATCCTCTTTCAGCAACTCTTTGGTCAATTATTGTTACACTTCCTACAATTCTTTATAGACTTAATTCTTTACTGCTTGCTGATGAATTTCGCAGCAAAGTTCTTGAGGATGCATTGAAACTTGGTTCACAAACGCCAAGTGATTTTGAATGGACTCCTCTTCAATATGTCACTCCAAATGATGATCAAAATCAAAAAAGTATCAGAAATTTGGATCAGTTAAGAAAAATAAACCAACAGGAAAAAGAAAATGAAGTAGCAATGGAATGTGATACTGTTGAAGAAAAAGGAAATGAAACGGCAGCTAGTGGAATAAATGATTTCGAAATCGGCGTTTGGGATCCAACACCTAACATTGACAATATCCTGGATGAGCCTCCTCCAGTTATTCATAATGCTCCTGTAAATGGATTAATCCCTGGACGAAGGAATGGACTTCGAGGAGTTATAGCTGCTAGGGACGAAGAACTCTCTGAAATTATTGCTGTTGGTAATGATACAACGATTCATAATTATGGAGATATTTCTGATGATGATGATGTTGCAGCTGAATATGATAAATTTAAATTTTTGATGCACAATAAAATGACTACAAGTGATATTGGTGAACTCGGTGAAATGGATGTTCGACCTGCAGGATGGAATGACGATAGCAATGTCAATGTTATACAAATTGAAAACGAAAGTCTTCCATTGACAATATCTACAAACAATCCTCATATCAATATTGCAAGTTTAATGAATGACTTGGAGAAAAATTATGCTGCTTTTGGTACATCAACAAGCACTGCAAACAATAATAAAGTTAATACTCCCGTCACTCCAAGTGCTACTACCACTGATAAAACGGTCATCCAAAGGAAAGAGCTTAATCTCGATTCATTAAATGTCATTGACCAGAATGATCCATCAATAAAAAGGCAAAATGAAGATATTTTACCTGAGGAAGTTTTTTGGGGAATGGATGAAATGGAAGAGAATATTAACAATGAAGCATTAACAACTTCAAGTGCTGCTGACGGAATAAACTTGGAAAGGCTAGAGACAATTGGCGATTCTTTCCTCAAAATGGCTGTCACCAACTATTTCTACCATAAACACACTGAGCAACACGAAGGAAAGCTAAGCTTTGCTCGTTCAAAAGAAGTTTCAAACTCTCATTTATTTTATCTTGGTCGGCAACGAGGCATTCCTCTTCTAATAGAAACATTAAAATTTGATCCACATGTTAATTGGCTTCCTCCTTGCTATGCTTCTACTTCAGAATTTCATGCTGTCAATCCATTTGATTACACAGATTTAGATGAAGAACAAGACCAACGTGAAGTGCCAATGGAGGGTGTTGAAACTAATAAAACTGTAGATCAACAACAAAAGAATAATAAAGAAACAATAGCAACAGGTTGGGGTACTCTTGATGATGACAGACAGAATTATAAATGTGAAAATGGTGTGGAGACATTAACTTTTCCACAACAAACAAAATCTGAAATCCCAGACTTACCCCCAATGCCATACAATATGCTTACTCAACAATGGATAAGTGACAAATCAATTGCTGATGCAGTAGAAGCATTAATTGGAGCGCATTTAATTCAACTTGGGCAATCATCTACGCTCAAATTTATGAATTGGTTGGGAATTAAGGTTTTAACTGACATTTCTTCATTGCCTTCACCACTTCTTCGTTTCATTGACACACCTGAAGATCCTAATCTTTCGCTCAAACATTTGGCCCTTTTTTATGAGAAATTTGACTTTGCTACGGTTGAGAACAATATTGGTTATAAATTTGCAAATAAGGCATATCTGGTTCAAGCATTTACACACGCTTCTTATTATAACAATCGAGTAACTGGTTGCTACCAGCGTTTGGAATTCCTTGGTGATGCAGTTTTGGATTATATGATAACTCGTTTCCTTTACGAACACAAAAGACAATACAGCCCTGGTGTACTTACTGATCTTCGCTCCGCTCTTGTCAATAATACAATTTTTGCTTCTTTAGCAGTCAAATATTCTTTTCATAAGCATTTTGTTATGATTTGTCCTCCACTTTATCAAATGGTTGAGAAATTTGTTAATTTCTGTAAACAAAAAGATTTTCTACATTGCGCGAATTTTGACGATGAAATATTTATGTTAACTGAAGAAGAAATAGATGAAGAAGACCTTGTAAGTGAAGAAGATGTAGAAGTGCCAAAAGCGATGGGTGATATTTTCGAATCAATAGCTGGTGCTGTTTATTTAGATTGTGGAATGGATTTAGATATTGTTTGGCGTGTATTTTATAATTTAATGAGAGATGTTATACAAAAATGCTGTGAAAATCCACCACAATCGCCAGTTAGGGAATTGTTTGAAAGAAAAAATTGTCGAGCAAAATTCTCAAAATTGGAACGAAAATTAGAAACTGGCAAAGTTAGAGTAACTGTAACAGTTAATGATAATCTCCAATTTACTGGTATGGGACGTAGTTATCGAATTGCTAAATGTACAGCTGCTAAACGAGCACTTCAACATTTAAGAAAATTAGATGCTGCAAAGAATAAATAA

>CABB01000157-*M. incognita* (36 exons)

ATGTCTCCACCGAGGGATTTTAGTGGTAAATGTATTCCTCCAAGAGATTATCAGTTTAAATTCAAGGTTGAATTACTTGATCGTGCAAAAATTCAAAACACGATTATTTCGCTCGGTACTGGTAGTGGAAAAACGTTTGTGGCGGTTCTTTTAATAAAGGAATATTCCCAGAGGTTGTTACATCAAAATGAAAAAGCTGTGTTTTTGGTTAATACTGTTGAACTTGTTGCCCAGCAAGCCGAGCATATTGAATTTCATTCAAGTTTGTCTGTCGCCCGTATTTCTGGCTCTACAATTAAAAGAAAATATGAGAGAAAAGAAGTTGAGAAGATTACTAACACCAATCAGGCTAGTCATGTGATTGTAATTACAGCTCAAGTGTTTCTTGATTTAATCAATCACGGCTTATTTGATTTCTCTTCTTTGGCCTTGTTGATTGTTGATGAATGCCATCATTGTTTGGGAGAACTGCATCCATATAGGCTGATTATGAATCATTACAAGAAACTTCAAGGTCAGCGACCACGCGTTTTGGGCCTTACAGCTTCAATTCTGAATAAAAAAGTGCCATCCTCTCGTATTGAGTGCACCGCTCAGCTGCTAGAACAAATAATGGATTCACAGATAGAAACTTCCAGCAATTATACACAGGAGCGCCTTCGTTCATTCATTGAAAAGAATGAAGATTTTCATTCAGAACTTGAAGTTGATCCGCGTCGCAAAATTTTTGAGACAATTTCCCGTTCATTTTCAACTCTTCAACAAGTTGGTAGCTGGGCAGCTCTAAAAGGTTTTATCCTTTGGCAAAAGAATTTACTTAAACATGTTGATGACCCAATAATTGGCAATAAACAAAAATGTATTTTAAGAATGGCTGAAACTGCTTTTAGAACTTGTTCAAAAGTTTTAAGTCACAAAATAAATCCGTTAAACAGCTATGACAAATTGAGTAACGTAAATAAATCATTTATCAGTGACAGAGTGCGCAAATTGTTGGAAATATTAAAAAGTTATTCGCCCTCGAAACGTGAATTGTCTGGAATTAAAGATACACTTTTTGGATTAGTTTTTGTGAAAGAACGATTTATTGCCTTTATGATTAATAATTTGCTTCGGTTTCTTGTAAAACAAAATCCTGAAGAGTTTGGACATTTAAAAGTGGACTTTATTGTTGGACATACTGGAAATTCTGAGACAGGCGATGAAGATAGGCGTTTGGTGAATCGAAAACAAGAACAAACTCTTACAAAATTCCGAAATGGCCAATTAAATTTATTAATTACGACAAATGTTTTGGAAGAAGGAATTGATTTAAGAAATTGTAATTTAGTCGTTCGATTTGATCCGCCGATGGACTTTCGTTCATTTATTCAATCTAGCGGTCGTGCTCGAAGAGAAAATAGCGCTTTTTATATACTTATTGAAGAGAAAAATTATTTGGATTTTATGATGGACTTAACAGGGTATGCACAAGCAGAAGAGTTTGTTCTGAGACGTTATCGTTCTGGCAATGATTTTACTTTGGAAGGAAATGATGAAACAAAAATATTACATCCACATCTGGATGATGCTGTTGCTCCTTATGTTGTTACAACTGAAAAAGGGACAGCTAAAGTCTCGTTAAGTAATGCTATACAGTACTGTGCAAAATTACCATCTGATATTTTTACTCGATTAGTGCCACGTTATACAATACAAACTTTATCAGAAAATGGACAAACTTTGTATATTGCCGAATTATATCTCCCAATCAATTCACCAATAAAGGAGCCAATAAAATCTAAACCGATGAATTCTAAAAGATTATCTTTAATGGCAGTCGCATTGGAGGCTTGCAAACGATTACATCAAAGAAAAGAATTAAATGACAATCTTTTGCCTGCAGGAAAGGATATCTTGGATGATCTTTTAGGTGAAGTTGATGATGATGAATATCTGCCTCATCTACCAAGCAGAATGGGATCCTCAAAGAAAAAACGTCTCTATGATAGAAAAATGTCGAAAACTCTTAATTCTACATTACCTTCGCAAGACAGTGAATGCATTCTTTACGTTATGGAAATGAAACTGGTTAAGCCCGTTACTGATGAAGGGAATCCGAAAAGGAGAAAAATTATTGATCCGTTCGAATCAAATTCGGCATTTGGATTTTTGTCTTCGAAGGAGCTCCCGAAGATTCCGGGATTTCCAGTTTTTCAACGGAATGGTGAAATGAATGTGCAAATTCGAAAAGTAAAGAATCAACAATTTCGTCCCACTTTTGAACTTTTACAACTTATTTACCTCTTTCATCAACACATCTTTGAAGATATTTTGCGTGTTGCTCGTGGTGGTGTTGTTTTTGCACCTGGTCATTCTCCAATACCACTTTTAATTGTTCCTTTGAAAAAATTAAGCAATGCCGATTTGGACTATGAAATTGACAAAGATTACCTAAATTGGGATATTCGTGAATCTCCAACTACACCTTCAGATGAAATTAGAAAACAATTCTCTTTTGAAGAATCAAACTACTTAAATGCTGTTGTTTCACCTTGGTATCGTTCTGAAGATCAGTCTGCTTTTTATTACGTAGCTGAGATAATGACTGATCAATTTCCGTCTTCTTCATTTCCTGATGAAAAATTTACTTGTTTTAATCAATATTTTATGAGTAAATATCAATTGGAGATCTACAATCAAAAACAAAATTTACTTGATGTTGACCATACATCTGCTAGAATGAATCTTTTATTACCACGAGCAATTACTGGAAAATCAGCTACTCGTTCATTAGACCCATCTCAACGTCAAATTCTTGTTCCAGAATTAGTACATATACATCCTCTTTCTGCAACTCTTTGGTCAATTATTGTTACTCTTCCTACAATTCTTTATAGACTTAATTCTTTACTGCTTGCTGATGAATTTCGTAGCAAAGTTCTTGAAGATGCTTTAAAAATTAATTCACAAACACCCGACGATTTTGAATGGAGTCCTCTTCAATATGTCACTCTAAATGATGATCTGACACAAAAAAGTATAAGAAATTTAGATCAGTTGAGAAAAATGAACAAACAAGAGAAGGAAAATGAAGTACCAATGGAGTGTGAGAATATTGAGGAGAATGTGAATGAGACAGCTGCAAGTGGAATAGTGGGTGAATTAAAAAATTTTAGATCAAATGATTTTGAAATCGGCGTTTGGGACCCTTCAGATGGCGCACAAATGTCTAACAATGATAATACTCTGAATGAACCTTCCCCAGTCATTCATAATGCACCTGTAAATGGATTAATCCCTGGTCGGAGGAACGGTCTTCGAGGAGTTATAGCTGCTAGAGACGAGGAACTCTCTGAAATTATTGCTGTCGGGAATGATACAACACTTCATAATTATGGAGATATTTCTGATGATGATGATGTTGCAGCTGAATATGATAAATTTAAATTTTTGATGGACAATAAAATGACTACAAGTGATATTGGTGATCTCGGTGAAATGGATGTTCGACCTGCTGGGTGGAATGACGACAGTAATGCCAATGTTATCCAAATTGAAAACGAAAGTCTTCCCTTGACAATATCGACAAACAATCCTCATATTAATATTGCAAGTTTAATGAATGATTTGGAGAAAAATTATGCTGCTTTTGGTACTTCAAGCCATATTTCGAATAATACTAGAACGAATACACCGATCATTTCGAGTGATACTGCTGTTGACAAAACGGCCTTGCAAAGAAAGGAGGTCCTCTTAACATATCTTACTAACAATTCGGTAAAAAGGCAAAATGAAGATATTTTACCTGAGGAAGTTTTTTGGGGAATGGATGAAGCTGAGATAGGAGAAAATATTAGCAGTGAAGCATTGACAACTTCGAGTGCTGCGGATGGAATAAATTTGGAAAGATTAGAAACAATTGGAGATTCCTTTCTTAAAATGGCTGTCACCAACTATTTCTACTATAAACACACTGAACAACATGAAGGAAAGTTAAGCTATGCTCGTTCAAAAGAAGTTTCGAATTCTCATCTTTTTTATCTTGGACGGCAACGAGGCATCCCTCTTCTAATAGAAACACTTAAATTCGATCCACATGTTAACTGGCTTCCTCCTTGTTATGCTTCTACTTCAGAATTCCATGCTGTCAATCCTTTTGATTATACAGATTTAGATGAAGATCAATGTCAAGTGCCAATGGAGGGTGTCGATACTACTGAAACTTTAGATCAACAGCAAAAGACTAATAAAGAAACAATAGCAACTGGATGGGGTACTCTTGACGATGATAAACAAAATTATAAACGTGAAAATGGAGTAGAAACTTTAACTTTTCCACAACCAACAAAATCAGAAATTCCAGACTTACCTCCAATGCCATACAATATGCTTACTCAACAATGGATAAGTGACAAATCAATTGCTGATGCAGTAGAGGCATTAATTGGAGCACATTTAATTCAACTTGGACAATCAGCTACTCTCAAATTTATGAATTGGTTGGGAATTAAGGTTCTAACTGACATTTCTTCATTGCCCTCACCACTTCTACGTTTCATTGACACACCTGAAGATCCTAATCTTTCACTTAAACATCTTGCCCTTCTTTATGAGAAATTTGATTTTGCTACTGTTGAGAACAATATTGGCTATAAATTTGCAAATAAGGCATACTTAGTCCAAGCATTTACTCACGCTTCATATTATAATAATCGAGTAACCGGTTGTTACCAGCGTTTGGAATTCCTTGGCGATGCTGTTTTAGATTATATGATAACTCGTTTTCTTTATGAACACAAGAAGCAATATAGTCCGGGTGTTCTTACTGATCTTCGCTCCGCTCTTGTCAATAATACAATTTTTGCTTCGCTTGCAGTTAAATATTCTTTTCACAAACATTTTGTTATGATTTGTCCTCCACTTTATCAAATGGTCGAAAAGTTTGTTAATTTCTGTAAGCAAAAAGATTTTCTTCATTGCGCGAATTTTGACGATGAAATATTTATGTTAACTGAAGATGAAATCGATGAGGAAGACCTTTTAAGTGAAGAAGATGTCGAAGTACCTAAAGCAATGGGTGATATTTTTGAATCAATTGCTGGCGCTGTTTATTTAGATTGTGGAATGGATTTAGATATTGTTTGGCGTGTATTTTATAATTTAATGAGAGATGTTATACAAAAATGTTGTGAAAATCCACCACAATCGCCAGTTAGAGAATTATTTGAAAGGAAGAATTGTCGAGCAAAATTCTCAAAATTGGAACGAAAATTAGAAACAGGCAAAGTTAGAGTTACTGTAACGGTTAATGATAATCTCCAATTTACTGGTATGGGAAGGAGTTATCGAATTGCTAAATGTACAGCTGCTAAACGAGCACTTCAACATTTAAGAAAATTGGATGCCACGAAAACAGCAAAGAATAAATAA

>CACSLI010000069-*M. luci* (35 exons)

ATGTCTCCACCGAAGGATTTTAGTGGTAAATGTATTCCTCCAAGGGATTATCAGGTTGAATTGCTTGATCGTGCAAAAATTCAAAACACGATTATTTCGCTCGGTACTGGTAGTGGAAAAACGTTTGTGGCGGTTCTTTTAATAAAGGAATATTCTCAGAGATTGTTACATCAAAATGAAAAAGCTGTGTTTTTGGTTAATACTGTTGAACTTGTTGCCCAGCAAGCCGAGCACATTGAATTTCATTCAAGTTTGTCTGTCGCTCGTATTTCTGGCTCTACAATTAAAAGAAAATATGAGAGAAAAGAAGTTGAGAAAATTACTAGCAGCAATCAGGCAAGTCATGTATTATTAAAGTCTAGTTTATTTTTTGTTTTAAAGGTGATTGTAATTACAGCTCAAGTGTTTCTTGATTTAATTAATCACGGGTTATTCGATTTCTCTTCTTTGGCCTTGTTGATTGTTGATGAATGCCATCATTGTTTGGGAGAACTGCATCCATATAGGCTTATTATGAATCATTACAAGAAACTGCAAGGTCAACGACCACGCGTTTTGGGCCTTACAGCTTCAATTCTGAATAAAAAAGTGCCATCCTCTCGTATTGAGTGCACCGCTCAGCTGCTAGAACAAATAATGGATTCACGGATAGAGACCTCCAGCAATTATACACAGGAGCGCCTTCGTTCATTTATTGAAAAGAATGAAGATTTTCATTCAGAACTTGAAGTTGATCCGCGTCGCAAAATTTTTGAGACAATTTCCCGTTCATTTTCAACTCTTCAACAAGTCGGTAGCTGGGCAGCTCTAAAAGGTTTTATCCTTTGGCAAAAGAATTTACTTAAACATGTTGATGACCCAATAATTGGCAATAAACAAAAATGTATTTTAAGAATGGCTGAGACTGCTTTTAGAACTTGTTCAAAAGTTTTAAGTCACAAAATAAATCCGTTAAACAGCTATGACAAATTGGTTAACGTAAATAAATCATTTATCAGTGATAGAGTGCGCAAATTGTTGGAAATATTAAAAAGTTATTCACCCTCAAAACGTGAATTGTCTGGAATTAAAGATACACTTTTTGGATTAGTTTTTGTGAAAGAACGATTTATTGCCTTTATGATTAATAATTTGCTTCGGTTTCTTGTAAAACAAAATCCTGAAGAGTTTGGACATTTAAAAGTGGACTTTATTGTTGGACACACTGGAAATTCTGAGACAGGAGATGAAGATAGGCGTTTGGTGAATCGAAAACAAGAACAAACTCTTACAAAATTCCGAAATGGTCAATTAAATTTATTAATTACGACAAATGTTTTGGAAGAAGGAATTGATTTAAGAAATTGTAATTTAGTCGTTCGATTTGATCCGCCGATGGATTTTCGGTCATTTATTCAATCTAGCGGTCGTGCTCGAAGAGAAAATAGCGCTTTTTATATACTTATTGAAGAGAAAAATTATTTGGAGTTTATGATGGACTTAACAGGATATGCACAAGCAGAAGAGTTTGTTTTGCGGCGTTATCGTTCTGGCAATGATTTTACTCTGGAAGGAAATGATGAAACAAAATTATTACATCCACATCTGGATGATGCTGTTGCTCCTTATGTTGTTACAACTGAAAAAGGGGCAGCTAAAGTCTCATTAAGTAATGCTATACAATATTGTGCAAAATTACCATCTGATATTTTTACTCGATTAGTGCCTCGTTATACAATACAAACTTTATCAGAAAATGGACAAACTTTGTATATTGCTGAATTATACCTTCCAATCAATTCACCAATAAAGGAGCCAATAAAATCTAAGCCGATGACTTCTAAAAGATTATCTTTAATGGCAGTCGCTTTGGAGGCTTGCAAACGATTACATCAAAGAAAAGAATTAAATGACAATCTTTTGCCTGCAGGAAAAGATATCTTGGATGATCTTTTAGGGGAAGTTGATGATGATGAATATCTGCCTCATTTACCAAGCAGAATGGGATCCTCAAAGAAAAAACGTCTCTACGATAGAAAAATGTCGAAAACTCTTAATTCTACATTACCTTCGCAAGACAGTGAATGCGTTCTTTACGTTATGGAAATGAAACTGATTAAGCCCGTTACTGATGAAGGGAATCCGAAAAGGAGAAAAATTATTGATCCGTTCGAGTCAAATTCGGCGTTTGGCTTTTTGTCTTCGAAGGAGCTCCCGAAGGTTCCGGGATTTCCAGTTTTTCAACGGAATGGTGAAATGAATGTGCAAATTCGTAAAGCAAAGAATCAGCAATTTCGTCCCACTTTTGAACTTTTACAACTTATTTACCTCTTTCATCAACACATCTTTGAAGATATTTTGCGTGTTGCCCGTGGTGGTGTTGTTTTTGCACCTGGTCATTCTCCAATACCACTTTTAATTGTTCCCTTGAAAAAATTAAGCACTGCCGATTTGGATTATGAAATTGACAGAGATTACCTAAATTGGAATATCCGAGAATCTCCAACTACACCTTCAGATGAAATTAGAAAACAATTTTCTTTTGAAGAATCAAACTACTTAAATGCTGTTGTTTCACCTTGGTATCGTTCTGAAGATCAGTCTGCTTTTTATTACGTAGCTGAGATAATGACTGATCAATTTCCGTCTTCTTCATTTCCTGATGAAAAATTTGCTTGTTTTAATCAATACTTTATGAGTAAATATCAATTGGAGATCTACAATCAAAAACAAAATTTACTTGATGTTGACCATACATCTGCTAGAATGAATCTTTTATTACCACGAGCAATTTCTGGAAAATCAGCTACTCGATCATTGGACCCATCTCAACGTCAAATTCTTGTCCCAGAATTGGTACATATACATCCTCTTTCTGCAACTCTTTGGTCAATTATTGTTACTCTTCCTACAATTCTTTATAGACTTAATTCTTTACTGCTTGCTGATGAATTTCGTAGCAAAGTTCTTGAAGACGCTTTGAAAATTAATTCACAAACACCCGACGATTTTGAATGGAGTCCTCTTCAATATGTCACTCTAAATGATGATCTGACACAAAAAAGTATAAGAAATTTAGATCAGTTGAGAAAAATGAACAAACAAGAGAAGGAAAATGAAGTACCAATGGAGTGTGACATTATTGAAGAGAATGTGAATGAGACAGCAGCAAGTGGAATAGTGGGTAGATTAAAATTTTTGAAATCAAATGATTTTGAAATCGGCGTTTGGGACCCTTCAGATGGCGCACAAATGTCTAACAATGATAATACTCCGAATGAACCTTCTCCAGTCATCCATAATGCACCTGTAAATGGATTAATCCCTGGGCGGAGGAATGGTCTTCGAGGAGTTATAGCTGCTAGAGACGAGGAACTCTCAGAAATTATTGCTGTTGGTAATGATACAACGCTTCATAATTATGGAGATATTTCTGATGATGATGATGTTGCAGCTGAATACGATAAATTTAAATTTTTGATGGACAATAAAATGACTACAAGTGATATTGGTGATCTAGGTGAAATGGATGTTCGACCTGCTGGTTGGAATGACGATAGCAATGCCAATGTTATCCAAATTGAAAATGAAAGTCTTCCCTTGACAATATCGACAAACAATCCTCATATTAATATTGCAAGTTTAATGAATGATTTGGAGAAAAATTATGCTGCTTTTGGTACTTCAAACCATATTTCCAATAATAATAAAACGAATACACCGAACATTCCGAATGCTACTGCTGACAAAACGGTCTTGCAAAAAAAGGAGGTCCTCTTAACATATCTTATTAACAATTCGGTAAAAAGGCAAAATGAAGATATTTTACCTGAGGAAGTTTTTTGGGGAATGGATGAAGCTGAGATAGGAGAGAATATTAGCAGTGAAGCATTGACAACTTCAAGTGCTGCGGATGGAATAAATTTGGAAAGGTTGGAAACAATTGGAGATTCTTTCCTCAAGATGGCTGTCACCAACTATTTCTACTATAAACACACGGAACAACATGAAGGAAAGTTAAGCTATGCACGTTCAAAAGAAGTTTCGAATTCCCATCTTTTTTATCTTGGACGGCAACGAGGAATCCCTCTTTTAATAGAAACACTGAAATTCGATCCACATGTTAACTGGCTTCCTCCTTGTTATGCTTCTACTTCAGAATTCCATGCTGTCAATCCTTTTGATTATACAGATTTAGATGAAGATCAATGTGCAGTGCCAATGGAGGGTGTTGATACTACTGAAACTGTAGATCAACAGCAAAAGATTAATAAAGAAACAATAGCAACTGGATGGGGAACTATTGACGATGATAAACAAAATTATAAACGTGAAAATGGAGTAGAAACTTTAACTTTTCCACAACCAACAAAATCAGAAATTCCGGACTTACCTCCAATGCCATACAATATGCTTACTCAACAATGGATAAGTGACAAATCGATTGCTGATGCAGTAGAGGCATTAATTGGAGCACATTTAATTCAACTTGGACAATCAGCTACTCTGAAATTTATGAATTGGTTGGGAATTAAGGTTCTGACTGATATTTCTTCATTGCCCTCACCACTTCTTCGTTTCATTGACACACCTGAAGATCCTAATCTTTCACTTAAACATCTTGCCCTTCTTTATGAGAAATTTGATTTTGCCACTGTTGAGAACAATATTGGTTATAAATTTGCAAATAAGGCATATTTAGTCCAAGCATTTACTCACGCTTCATATTATAACAATCGAGTAACCGGTTGTTACCAACGTTTGGAATTCCTTGGCGATGCTGTTTTAGATTATATGATAACTCGTTTTCTTTATGAACATAAGAAGCAATATAGTCCTGGTGTTCTTACTGATCTTCGCTCCGCTCTTGTCAATAATACAATTTTTGCTTCGCTTGCAGTTAAATATTCTTTTCACAAACATTTTGTTATGATTTGTCCTCCACTTTATCAAATGGTCGAAAAGTTTGTTAATTTCTGTAAGCAAAAAGATTTTCTTCATTGCGCGAATTTTGACGATGAAATATTTATGTTAACTGAAGATGAAATCGATGAGGAAGACCTTTTAAGTGAAGAAGATGTAGAAGTACCTAAAGCGATGGGTGATATTTTCGAATCAATTGCTGGTGCTGTTTATTTAGATTGTGGAATGGATTTAGATATTGTTTGGCGTGTATTTTATAATTTAATGAGAGATGTTATACAAAAATGTTGTGAAAATCTACCACAATCGCCAGTTAGAGAATTATTTGAGAGAAAGAATTGTCGAGCAAAATTTTCAAAATTGGAACGAAAATTAGAAACAGGCAAAGTTAGAGTTACTGTAACGGTTAATGATAATCTCCAATTTACTGGTATGGGAAGGAGTTATCGAATTGCTAAATGTACAGCTGCTAAACGAGCACTACAACATTTAAGAAAATTAGATGCCACAAAAACAGCAAAGAATAAATAA

>CACSLI010000224-*M. luci* (36 exons)

ATGTCTCCACCGAAGGATTTTAGTGGTAAATGTATTCCTCCAAGAGATTATCAGTTTAAATTCAAGGTTGAATTACTTGATCGTGCAAAAATTCAAAACACGATTATTTCGCTCGGTACTGGCAGTGGAAAAACATTTGTGGCTGTTCTTTTAATAAAAGAATACTCTCAGAGGTTGTTGCATCAAAATGAAAAAGCTGTGTTTCTGGTTAATACTGTTGAACTTGTTGCCCAGCAAGCCGAGCACATTGAATTTCATTCAAGTCTGTCTGTCGCCCGTATTTCTGGTTCTACAATTAAAAGAAAATATGAGAAAAAAGAAGTTGAGAAAATTACTAGCAGCAATCAGGCTAGTCATGTGATTGTAATTACAGCTCAAGTGTTTCTTGATTTAATCAATCACGGGTTATTCGATTTCTCTTCTTTGGCCTTGTTGATTGTTGATGAATGCCATCATTGCTTGGGAGAACTGCATCCATATAGGCTGGTTATGAATCATTACAAAAAACTGCAAGGTCAACGACCACGCGTTTTGGGCCTTACAGCTTCAATTCTGAATAAAAAAGTGCCATCCTCTCGCATTGAGTGCACCGCTCAGCTGCTAGAACAAATAATGGATTCACAGATAGAAACTTCCAGCAATTATACACAGGAGCGCCTTCGTTCATTCATTGAAAAGAATGAAGATTTTCATTCAGAACTTGAAGTTGATCCGCGTCGCAAAATTTTTGAGACAATTTCCCGTTCATTTTCAACTCTTCAACAAGTTGGTAGCTGGGCAGCTCTAAAAGGTTTTATCCTTTGGCAAAAGAATTTACTTAAACATGTTGATGATCCAATAATTGGCAATAAACAAAAATGTATTTTAAGAATGGCTGAAACTGCTTTTAGAACTTGCTCAAAAGTTTTAAGTCACAAAATAAATCCGTTAAACAGCTATGACAAATTGAGTAACGTAAATAAATCATTTATCAGTGACAGAGTGCGCAAATTGTTGGAAATATTAAAAAGTTATTCGCCCTCGAAACGTGAATTGTCTGGAATTAAAGATACACTTTTTGGATTAGTTTTTGTGAAAGAACGATTTATTGCCTTTATGATTAATAATTTGCTTCGGTTTCTTGTAAAACAAAATCCTGAAGAGTTTGGACATTTAAAAGTGGACTTTATTGTTGGACATACTGGTAATTCTGAGACAGGAGATGAAGATAGGCGTTTGGTGAATCGAAAACAAGAACAAACTCTTACAAAATTCCGAAATGGCCAATTAAATTTATTAATTACGACAAATGTTTTGGAAGAAGGAATTGATTTAAGAAATTGTAATTTAGTCGTTCGATTTGATCCGCCGATGGACTTTCGTTCATTTATTCAATCTAGCGGTCGTGCTCGAAGAGAAAATAGCGCTTTTTATATACTTATTGAAGAGAAAAATTATTTGGATTTTATGATGGACTTAACAGGGTATGCACAAGCAGAAGAGTTTGTTCTGAGACGTTATCGTTCTGGCAATGATTTTACTTTGGAAGGAAATGATGAAACAAAAATATTACATCCACATCTGGATGATGCTGTTGCTCCTTATGTTGTTACAACTGAAAAAGGGACAGCTAAAGTCTCGTTAAGTAATGCTATACAGTACTGTGCAAAATTACCATCTGATATTTTTACTCGATTAGTGCCACGTTATACAATACAAACTTTATCAGAAAATGGACAAACTTTGTATATTGCTGAATTATATCTCCCAATCAATTCACCAATAAAGGAGCCAATAAAATCTAAACCGATGAATTCTAAAAGATTATCTTTAATGGCAGTCGCATTGGAGGCTTGCAAACGATTACATCAAAGAAAAGAATTAAATGACAATCTTTTGCCTGCAGGAAAAGATATCTTGGATGATCTTTTAGGCGAAGTTGATGATGATGAATATCTGCCTCATTTACCAAGCAGAATGGGATCCTCAAAGAAAAAACGTCTCTATGATAGAAAGATGTCGAAAACTCTTAATTCTACATTACCTTCGCAAGACAGTGAATGCATTCTTTACGTTATGGAAATGAAATTGATTAAGCCTGTTACTGATGAAGGGAATCCAAAAAGGAGAAAAATTATTGATCCGTTTGAATCAAATTCGGCATTTGGATTTTTGTCTTCGAAGGAGCTCCCGAAGATTCCGGGATTTCCAGTTTTTCAACGAAATGGTGAAATGAATGTGCAAATTCGGAAAGTAAAGAATCAACAATTCCGTCCCAATTTTGAACTTTTACAACTTATTTACCTCTTTCATCAACACATCTTTGAAGATATTTTGCGTGTTGCCCGTGGTGGTGTTGTTTTTGCACCTGGTCATTCTCCAATTCCACTTTTAATTGTTCCTTTGAAAAAATTAAGCACTGCCGATTTGGATTATGAAATTGACAAAGATTACCTAAATTGGAATATTCGTGAATCTCCAACTACACCTTCAGATGAAATTAGAAAACAATTTTCTTTTGAAGAATCAAACTACTTAAATGCTGTTGTTTCACCTTGGTATCGTTCTGAAGATCAGTCTGCTTTTTATTACGTAGCTGAGATAATGACTGATCAATTTCCGTCTTCTTCATTTCCCGATGAAAAATTTACTTGTTTCAATCAATACTTTATGAGTAAATATCAATTGGAGATCTACAATCAAAAACAAAATTTACTTGATGTTGACCATACATCTGCTAGAATGAATCTTTTATTACCACGAGCAATTACTGGAAAATCAGCTACTCGATCATTGGACCCTTCTCAACGTCAAATTCTTGTTCCAGAATTAGTACATATACATCCTCTTTCTGCAACTCTTTGGTCAATTATTGTTACTCTTCCTACAATTCTTTATAGACTTAATTCTTTACTGCTTGCTGATGAATTTCGTAGCAAAGTTCTTGAAGATGCTTTGAAAATTAATTCACGAACGCCGGACGATTTTGAATGGAGTCCTCTTCAATATGTCACTTTAAATGATGATCTGACACAAAAAAGTATAAGAAATTTAGATCAGTTGAGAAAAATGAACAAACAAGAGAAGGAAAATGAAGTACCAATGGAGTGTGAGAATATTGAGGAGAATGTGAATGAGACAGCTGCAAATGGAATTGTGGGTAATTTTAAAATTTTGAGATCAATAATTAATGATTTCGAAATCGGCGTTTGGGACCCTTCAGATGGAGCTCAAATGTCTAACAATGATAATACTCCGAATGAACCTTCTCCAGTCATTCACAATGCACCTGTAAATGGATTAATCCCTGGGCGGAGGAATGGTCTTCGTGGAGTGATAGCTGCTAGAGACGAGGAGCTCTCTGAAATTATTGCTGTCGGGAATGATACAACACTTCATAATTATGGAGATATTTCTGATGATGATGATGTTGCAGCTGAATATGATAAATTTAAATTTTTGATGGACAATAAAATGACTACAAGTGATATTGGTGATCTAGGTGAAATGGATGTTCGACCTGCCGGGTGGAATGACGACAGCAATGCCAATGTTATCCAAATTGAAAACGAAAGTCTTCCATTGACAATATCGACAAACAATCCTCATATTAATATTGCAAGTTTAATGAATGATTTGGAGAAAAATTATGCTGCTTTTGGAACTTCAAACCATATTATAAATAATAAAACGAATACACCGATCATTTCGAGTGATACTGCTGTTGACAAAACGGTCTTACAAAGAAAGGAGGTCCTCTTAACATATCTTATTAACAATTCGGTAAAAAGGCAAAATGAAGATATTTTACCCGAGGAAGTTTTTTGGGGAATGGATGAAGCTGAGATAGGAGAGAATATTAGCAGTGAAGCATTGACAACTTCAAGTGCTGCGGATGGAATAAATTTGGAAAGGTTAGAAACAATTGGAGATTCTTTTCTCAAAATGGCTGTCACCAACTATTTCTACTATAAACACACTGAACAACATGAAGGAAAGTTAAGCTATGCTCGGTCAAAAGAAGTTTCGAACTCCCATCTTTTTTATCTTGGACGGCAACGAGGCATCCCTCTTCTAATAGAAACACTGAAATTCGATCCTCATGTTAACTGGCTTCCTCCTTGTTATGCTTCTACTTCAGAATTTCATGCTGTCAATCCTTTTGACTATACAGATTTAGATGAAGATCAATGTGCAGTGCCAATGGAGGGTGTTGATACTACTGAAACTGTAGATCAACAGCAAAAGATTAATAAAGAAACAATAGCAACTGGATGGGGAACTCTTGACGATGATAAACAGAATTATAAACGTGAAAATGGAGTAGAAACTTTAACTTTTCCACAACCAACAAAATCAGAAATTCCGGACTTACCTCCAATGCCATACAATATGCTTACTCAACAATGGATAAGTGACAAATCAATTGCTGATGCAGTAGAGGCGTTAATTGGAGCACATTTAATTCAACTTGGACAATCAGCTACTCTGAAATTTATGAATTGGTTGGGAATTAAGGTTCTAACTGATATTTCTTCATTGCCCTCACCACTTCTTCGTTTCATTGACACACCTGAAGATCCTAATCTTTCACTTAAACATCTTGCCCTTCTTTATGAGAAATTTGATTTTGCCACTGTTGAGAACAATATTGGTTATAAATTTGCAAATAAGGCATATTTAGTCCAAGCATTTACTCACGCTTCATATTATAACAATCGAGTAACCGGTTGTTACCAGCGTTTGGAATTCCTTGGCGATGCTGTTTTAGATTATATGATAACTCGTTTTCTTTATGAACACAAGAAGCAATATAGTCCGGGTGTTCTTACTGATCTTCGCTCCGCTCTTGTCAATAATACAATTTTTGCTTCGCTTGCAGTTAAATATTCTTTTCACAAACATTTTGTTATGATTTGTCCTCCACTTTATCAAATGGTCGAAAAGTTTGTTAATTTCTGTAAGCAAAAAGATTTTCTTCATTGCGCGAATTTTGACGATGAAATATTTATGTTAACTGAAGATGAAATTGATGAGGAAGACCTTTTAAGTGAAGAAGATGTCGAAGTACCTAAAGCAATGGGTGATATTTTTGAATCAATTGCTGGTGCTGTTTATTTAGATTGTGGAATGGATTTAGATATTGTTTGGCGTGTATTCTATAATTTAATGAGAGATGTTATACAAAAATGTTGTGAAAATCCACCACAATCGCCAGTTAGAGAATTATTTGAGAGAAAGAATTGTCGAGCAAAATTCTCAAAATTGGAACGAAAATTAGAAACAGGCAAAGTTAGAGTTACTGTAACGGTTAATGATAATCTCCAATTTACTGGTATGGGAAGGAGTTATCGAATTGCTAAATGTACAGCTGCTAAACGAGCACTTCAACATTTAAGAAAATTAGATGCCACAAAAACAGCAAAGAATAAATAA

>CEWM01000731- *M. arenaria* (35 exons)

ATGTCTCCACCGAAGGATTTTAGTGGTAAATGTATTCCTCCAAGGGATTATCAGGTTGAATTGCTTGATCGTGCAAAAATTCAAAACACGATTATTTCGCTCGGTACTGGTAGTGGAAAAACGTTTGTGGCGGTTCTTTTAATAAAGGAATATTCTCAGAGATTGTTACATCAAAATGAAAAAGCTGTGTTTTTGGTTAATACTGTTGAACTTGTTGCCCAGCAAGCCGAGCACATTGAATTTCATTCAAGTTTGTCTGTCGCTCGTATTTCTGGCTCTACAATTAAAAGAAAATATGAGAGAAAAGAAGTTGAGAAAATTACTAGCAGCAATCAGGCAAGTCATGTATTATTAAAGTCTAGTTTATTTTTTGTTTTAAAGGTGATTGTAATTACAGCTCAAGTGTTTCTCGATTTAATTAATCACGGGTTATTCGATTTCTCTTCTTTGGCCTTGTTGATTGTTGATGAATGCCATCATTGTTTGGGAGAACTGCATCCATATAGGCTTATTATGAATCATTACAAGAAACTGCAAGGTCAACGACCACGCGTTTTGGGCCTTACAGCTTCAATTCTGAATAAAAAAGTGCCATCCTCTCGCATTGAGTGCACCGCTCAGCTGCTAGAACAAATAATGGATTCACAGATAGAGACCTCCAGCAATTATACACAGGAGCGCCTTCGTTCATTTATTGAAAGGAATGAAGATTTTCATTCAGAACTTGAAGTTGATCCGCGTCGCAAAATTTTTGAGACAATTTCCCGTTCATTTTCAACTCTTCAACAAGTTGGTAGCTGGGCAGCTCTAAAAGGTTTTATCCTTTGGCAAAAGAATTTACTTAAACATGTTGATGACCCAATAATTGGCAATAAACAAAAATGTATTTTAAGAATGGCTGAGACTGCTTTTAGAACTTGTTCAAAAGTTTTAAGTCACAAAATAAATCCGTTAAACAGCTATGACAAATTGGTTAACGTAAATAAATCATTTATCAGTGATAGAGTGCGCAAATTGTTGGAAATATTAAAAAGTTATTCACCCTCAAAACGTGAATTGTCTGGAATTAAAGATACACTTTTTGGATTAGTTTTTGTGAAAGAACGATTTATTGCCTTTATGATTAATAATTTGCTTCGGTTTCTTGTAAAACAAAATCCTGAAGAGTTTGGACATTTAAAAGTGGACTTTATTGTTGGACACACTGGAAATTCTGAGACAGGAGATGAAGATAGGCGTTTGGTGAATCGAAAACAAGAACAAACTCTTACAAAATTCCGAAATGGTCAATTAAATTTATTAATTACGACAAATGTTTTGGAAGAAGGAATTGATTTAAGAAATTGTAATTTAGTCGTTCGATTTGATCCGCCGATGGATTTTCGGTCATTTATTCAATCTAGCGGTCGTGCTCGAAGAGAAAATAGCGCTTTTTATATACTTATTGAAGAGAAAAATTATTTGGAGTTTATGATGGACTTAACAGGATATGCACAAGCAGAAGAGTTTGTTTTGCGGCGTTATCGTTCTGGCAATGATTTTACTCTGGAAGGAAATGATGAAACAAAATTATTACATCCACATCTGGATGATGCTGTTGCTCCTTATGTTGTTACAACTGAAAAAGGGACAGCTAAAGTCTCATTAAGTAATGCTATACAATATTGTGCAAAATTACCATCTGATATTTTTACTCGATTAGTGCCTCGTTATACAGTACAAACTTTATCAGAAAATGGACAAACTTTGTATATTGCTGAATTATACCTTCCAATCAATTCACCAATAAAGGAGCCAATAAAATCTAAGCCGATGACTTCTAAAAGATTATCTTTAATGGCAGTCGCTTTGGAGGCTTGCAAACGATTACATCAAAGAAAAGAATTAAATGACAATCTTTTGCCTGCAGGAAAAGATATCTTGGATGATCTTTTAGGGGAAGTTGATGATGATGAATATCTGCCTCATTTACCAAGCAGAATGGGATCCTCAAAGAAAAAACGTCTCTACGATAGAAAAATGTCGAAAACTCTTAATTCTACATTACCTTCGCAAGACAGTGAATGCGTTCTTTACGTTATGGAAATGAAACTGATTAAGCCCGTTACTGATGAAGGGAATCCGAAAAGGAGAAAAATTATTGATCCGTTCGAGTCAAATTCGGCGTTTGGCTTTTTGTCTTCGAAGGAGCTCCCGAAGGTTCCGGGATTTCCAGTTTTTCAACGGAATGGTGAAATGAATGTGCAAATTCGTAAAGTAAAGAATCAGCAATTTCGTCCCACTTTTGAACTTTTACAACTTATTTACCTCTTTCATCAACACATCTTTGAAGATATTTTGCGTGTTGCCCGTGGTGGTGTTGTTTTTGCACCTGGTCATTCTCCAATACCACTTTTAATTGTTCCCTTGAAAAAATTAAGCACTGCCGATTTGGATTATGAAATTGACAGAGATTACCTAAATTGGAATATCCGAGAATCTCCAACTACACCTTCAGATGAAATTAGAAAACAATTTTCATTTGAAGAATCAAACTACTTAAATGCTGTTGTTTCACCTTGGTATCGTTCTGAAGATCAGTCTGCTTTTTATTACGTAGCCGAGATAATGACTGATCAATTTCCGTCTTCTTCATTTCCTGATGAAAAATTTGCTTGTTTTAATCAATACTTTATGAGTAAATATCAATTGGAGATCTACAATCAAAAACAAAATTTACTTGATGTTGACCATACATCTGCTAGAATGAATCTTTTATTACCACGAGCAATTTCTGGAAAATCAGCTACTCGATCATTGGACCCATCTCAACGTCAAATTCTTGTCCCAGAATTGGTACATATACATCCTCTTTCTGCAACTCTTTGGTCAATTATTGTTACTCTTCCTACAATTCTTTATAGACTTAATTCTTTACTGCTTGCTGATGAATTTCGTAGCAAAGTTCTTGAAGACGCTTTGAAAATTAATTCACAAACACCCGACGATTTTGAATGGAGTCCTCTTCAATATGTCACTCTAAATGATGATCTGACACAAAAAAGTATAAGAAATTTAGATCAGTTGAGAAAAATGAACAAACAAGAGAAGGAAAATGAAGTACCAATGGAGTGTGACATTATTGAAGAGAATGTGAATGAGACAGCAGCAAGTGGAATAGTGGGTAGATTAAAATTTTTGAAATCAAATGATTTTGAAATCGGCGTTTGGGACCCTTCAGATGGCGCACAAATGTCTAACAATGATAATACTCCGAATGAACCTTCTCCAGTCATCCATAATGCACCTGTAAATGGATTAATCCCTGGGCGGAGGAATGGTCTTCGAGGAGTTATAGCTGCTAGAGACGAGGAACTCTCAGAAATTATTGCTGTTGGTAATGATACAACGCTTCATAATTATGGAGATATTTCTGATGATGATGATGTTGCAGCTGAATACGATAAATTTAAATTTTTGATGGACAATAAAATGACTACAAGTGATATTGGTGATCTAGGTGAAATGGATGTTCGACCTGCTGGTTGGAATGACGATAGCAATGCCAATGTTATCCAAATTGAAAATGAAAGTCTTCCCTTGACAATATCGACAAACAATCCTCATATTAATATTGCAAGTTTAATGAATGATTTGGAGAAAAATTATGCTGCTTTTGGTACTTCAAACCATATTTCCAATAATAATAAAACGAATACACCGAACATTCCGAATGCTACTGCTGACAAAACGGACTTGCAAAAAAAGGAGGTCCTCTTAACATATCTTATTAACAATTCGGTAAAAAGGCAAAATGAAGATATTTTACCTGAGGAAGTTTTTTGGGGAATGGATGAAGCTGAGATAGGAGAGAATATTAGCAGTGAAGCATTGACAACTTCAAGTGCTGCGGATGGAATAAATTTGGAAAGGTTAGAAACAATTGGAGATTCTTTTCTCAAAATGGCTGTCACCAACTATTTCTACTATAAACACACTGAACAACATGAAGGAAAATTAAGCTATGCTCGGTCAAAAGAAGTCTCGAATTCTCATCTTTTTTATCTTGGACGGCAACGAGGCATCCCTCTTCTAATAGAAACACTTAAATTCGATCCACATGTTAACTGGCTTCCTCCTTGTTATGCTTCTACTTCAGAATTCCATGCTGTCAATCCTTTTGATTATACAGATTTAGATGAAGATCAATGTCAAGTGCCAATGGAAGGTGTTGATACTACTGAAACTTTAGATCAACAGCAAAAGACTAATAAAGAAACAATAGCAACTGGATGGGGTACTCTTGACGATGATAAACAAAATTATAAACGTGAAAATGGAGTAGAAACTTTAACTTTTCCACAACCAACAAAATCAGAAATTCCAGACTTACCTCCAATGCCATACAATATGCTTACTCAACAATGGATAAGTGACAAATCAATTGCTGATGCAGTAGAGGCATTAATTGGAGCACATTTAATTCAACTTGGACAATCAGCTACTCTCAAATTTATGAATTGGTTGGGAATTAAGGTTCTAACTGACATTTCTTCATTGCCCTCACCACTTCTACGTTTCATTGACACACCTGAAGATCCTAATCTTTCACTTAAACATCTTGCCCTTCTTTATGAGAAATTTGATTTTGCTACTGTTGAGAACAATATTGGCTATAAATTTGCAAATAAGGCATACTTAGTCCAAGCATTTACTCACGCTTCATATTATAATAATCGAGTAACCGGTTGTTACCAGCGTTTGGAATTCCTTGGCGATGCTGTTTTAGATTATATGATAACTCGTTTTCTTTATGAACACAAGAAGCAATATAGTCCGGGTGTTCTTACTGATCTTCGCTCCGCTCTTGTCAATAATACAATTTTTGCTTCGCTTGCAGTTAAATATTCTTTTCACAAACATTTTGTTATGATTTGTCCTCCACTTTATCAAATGGTCGAAAAGTTTGTTAATTTCTGTAAGCAAAAAGATTTTCTTCATTGCGCGAATTTTGACGATGAAATATTTATGTTAACTGAAGATGAAATCGATGAGGAAGACCTTTTAAGTGAAGAAGATGTCGAAGTACCTAAAGCAATGGGTGATATTTTTGAATCAATTGCTGGCGCTGTTTATTTAGATTGTGGAATGGATTTAGATATTGTTTGGCGTGTATTTTATAATTTAATGAGAGATGTTATACAAAAATGTTGTGAAAATCCACCACAATCGCCAGTTAGAGAATTATTTGAAAGGAAGAATTGTCGAGCAAAATTCTCAAAATTGGAACGAAAATTAGAAACAGGCAAAGTTAGAGTTACTGTAACGGTTAATGATAATCTCCAATTTACTGGTATGGGACGTAGTTATCGAATTGCTAAATGTACAGCTGCTAAACGAGCACTTCAACATTTAAGAAAATTAGATGCCACAAAAACAGCAAGGAATAAATAA

>CEWN01000587-*M. javanica* (35 exons)

ATGTCTCCACCGAAGGATTTTAGTGGTAAATGTATTCCTCCAAGAGATTATCAGTTTAAATTCAAGGTTGAATTACTTGATCGTGCAAAGATTCAAAACACGATTATTTCGCTCGGTACTGGGAGTGGAAAAACATTTGTGGCGGTTCTTTTAATAAAAGAATATTCTCAGAGATTGTTACATCAAAATGAAAAAGCTGTGTTTTTAGTTAATACTGTTGAACTTGTTGCCCAGCAAGCCGAGCACATTGAATTTCATTCAAGTCTGTCTGTCGCCCGTATTTCTGGCTCTACAATTAAAAGAAAATATGAGAGAAAAGAAGTTGAGAAAATTACTAGCAGCAATCAGGCAAGTCATGTATTATTAAAGTTTAGTTTATTTTTTGTTTTAAAGGTGATTGTAATTACAGCTCAAGTGTTTCTTGATTTAATCAATCACGGCTTATTCGATTTCTCTTCTTTGGCCTTGTTGATTGTTGATGAATGCCATCATTGCTTGGGAGAACTGCATCCATATAGGCTGATTATGAATCATTACAAGAAACTGCAAGGTCAACGACCACGCGTTTTGGGCCTTACAGCTTCAATTCTGAATAAAAAAGTGCCATCCTCTCGTATTGAGTGCACCGCTCAGCTGCTAGAACAAATAATGGATTCACAGATAGAAACTTCCAGCAATTATACACAGGAGCGCCTTCGTTCATTTATTGAAAAGAATGAAGATTTTCATTCAGAACTTGAAGTTGATCCGCGTCGCAAAATTTTTGAGACAATTTCCCGTTCATTTTCAACTCTTCAACAAGTTGGTAGCTGGGCAGCTCTAAAAGGTTTTATCCTTTGGCAAAAGAATTTACTTAAACATGTTGATGACCCAATAATTGGCAATAAACAAAAATGTATTTTAAGAATGGCCGAGACTGCTTTTAGAACTTGTTCAAAAGTTTTAAGTCACAAAATAAATCCGTTAAACAGCTATGACAAATTGGTTAACGTAAATAAATCATTTATCAGTGATAGAGTGCGCAAATTGTTGGAAATATTAAAAAGTTATTCACCCTCAAAACGTGAATTGTCTGGAATTAAAGATACACTTTTTGGATTAGTTTTTGTGAAAGAACGATTTATTGCCTTTATGATTAATAATTTGCTTCGGTTTCTTGTAAAACAAAATCCTGAAGAGTTTGGACATTTAAAAGTGGACTTTATTGTTGGACACACTGGAAATTCTGAGACAGGAGATGAAGATAGGCGTTTGGTGAATCGAAAACAAGAACAAACTCTTACAAAATTCCGAAATGGTCAATTAAATTTATTAATTACGACAAATGTTTTGGAAGAAGGAATTGATTTAAGAAATTGTAATTTAGTCGTTCGATTTGATCCGCCGATGGATTTTCGGTCATTTATTCAATCTAGCGGTCGTGCTCGAAGAGAAAATAGCGCTTTTTATATACTTATTGAAGAGAAAAATTATTTGGAGTTTATGATGGACTTAACAGGATATGCACAAGCAGAAGAGTTTGTTTTGCGGCGTTATCGTTCTGGCAATGATTTTACTCTGGAAGGAAATGATGAAACAAAATTATTACATCCACATCTGGATGATGCTGTTGCTCCTTATGTTGTTACAACTGAAAAAGGGACAGCTAAAGTCTCATTAAGTAATGCTATACAATATTGTGCAAAATTACCATCTGATATTTTTACTCGATTAGTGCCTCGTTATACAATACAAACTTTATCAGAAAATGGACAAACTTTGTATATTGCTGAATTATACCTTCCAATCAATTCACCAATAAAGGAGCCAATAAAATCTAAGCCGATGACTTCTAAAAGATTATCTTTAATGGCAGTCGCTTTGGAGGCTTGCAAACGATTACATCAAAGAAAAGAATTAAATGACAATCTTTTGCCTGCAGGAAAAGATATCTTGGATGATCTTTTAGGGGAAGTTGATGATGATGAATATCTGCCTCATTTACCAAGCAGAATGGGATCCTCAAAGAAAAAACGTCTCTACGATAGAAAAATGTCGAAAACTCTTAATTCTACATTACCTTCGCAAGACAGTGAATGCGTTCTTTACGTTATGGAAATGAAACTGATTAAGCCCGTTACTGATGAAGGGAATCCGAAAAGGAGAAAAATTATTGATCCGTTCGAGTCAAATTCGGCGTTTGGCTTTTTGTCTTCGAAGGAGCTCCCGAAGGTTCCGGGATTTCCAGTTTTTCAACGGAATGGTGAAATGAATGTGCAAATTCGTAAAGTAAAGAATCAGCAATTTCGTCCCACTTTTGAACTTTTACAACTTATTTACCTCTTTCATCAACACATCTTTGAAGATATTTTGCGTGTTGCCCGTGGTGGTGTTGTTTTTGCACCTGGTCATTCTCCAATACCACTTTTAATTGTTCCCTTGAAAAAATTAAGCACTGCCGATTTGGATTATGAAATTGACAGAGATTACCTAAATTGGAATATCCGAGAATCTCCAACTACACCTTCAGATGAAATTAGAAAACAATTTTCTTTTGAAGAATCAAACTACTTAAATGCTGTTGTTTCACCTTGGTATCGTTCTGAAGATCAGTCTGCTTTTTATTACGTAGCCGAGATAATGACTGATCAATTTCCGTCTTCTTCATTTCCTGATGAAAAATTTGCTTGTTTTAATCAATACTTTATGAGTAAATATCAATTGGAGATCTACAATCAAAAACAAAATTTACTTGATGTTGACCATACATCTGCTAGAATGAATCTTTTATTACCACGAGCAATTTCTGGAAAATCAGCTACTCGATCATTGGACCCATCTCAACGTCAAATTCTTGTCCCAGAATTGGTACATATACATCCTCTTTCTGCAACTCTTTGGTCAATTATTGTTACTCTTCCTACAATTCTTTATAGACTTAATTCTTTACTGCTTGCTGATGAATTTCGTAGCAAAGTTCTTGAAGACGCTTTGAAAATTAATTCACAAACACCCGACGATTTTGAATGGAGTCCTCTTCAATATGTCACTCTAAATGATGATCTGACACAAAAAAGTATAAGAAATTTAGATCAGTTGAGAAAAATGAACAAACAGGAGAAGGAAAATGAAGTACCAATGGAGTGTGACATTATTGAAGAGAATGTGAATGAGACAGCAGCAAGTGGAATAGTGGGTAGATTAAAATTTTTGAAATCAAATGATTTTGAAATCGGCGTTTGGGACCCTTCAGATGGCGCACAAATGTCTAACAATGATAATACTCCGAATGAACCTTCTCCAGTCATCCATAATGCACCTGTAAATGGATTAATCCCTGGGCGGAGGAATGGTCTTCGAGGAGTTATAGCTGCTAGAGACGAGGAACTCTCAGAAATTATTGCTGTTGGTAATGATACAACGCTTCATAATTATGGAGATATTTCTGATGATGATGATGTTGCAGCTGAATATGATAAATTTAAATTTTTGATGGACAATAAAATGACTACAAGTGATATTGGTGATCTAGGTGAAATGGATGTTCGACCTGCTGGTTGGAATGACGATAGCAATGCCAATGTTATCCAAATTGAAAATGAAAGTCTTCCCTTGACAATATCGACAAACAATCCTCATATTAATATTGCAAGTTTAATGAATGATTTGGAAAAAAATTATGCTGCTTTTGGTACTTCAAACCATATTTCCAATAATAATAAAACGAATACACCGAACATTCCGAATGCTACTGCTGACAAAACGGTCTTGCAAAAAAAGGAGGTCCTCTTAACATATCTTATTAACAATTCGGTAAAAAGGCAAAATGAAGATATTTTACCTGAGGAAGTTTTTTGGGGAATGGATGAAGCTGAGATAGGAGAGAATATTAGCAGTGAAGCATTGACAACTTCAAGTGCTGCGGATGGAATAAATTTGGAAAGGTTGGAAACAATTGGAGATTCTTTCCTCAAGATGGCTGTCACCAACTATTTCTACTATAAACACACGGAACAACATGAAGGAAAGTTAAGCTATGCACGTTCAAAAGAAGTTTCGAATTCCCATCTTTTTTATCTTGGACGGCAACGAGGAATCCCTCTTTTAATAGAAACACTGAAATTCGATCCACATGTTAACTGGCTTCCTCCTTGTTATGCTTCTACTTCAGAATTCCATGCTGTCAATCCTTTTGATTATACAGATTTAGATGAAGATCAATGTGCAGTGCCAATGGAGGGTGTTGATACTACAGAAACTGTAGATCAACAGCAAAAGATTAATAAAGAAACAATAGCAACTGGATGGGGAACTATTGACGATGATAAACAAAATTATAAACGTGAAAATGGAGTAGAAACTTTAACTTTTCCACAACCAACGAAATCAGAAATTCCGGACTTACCTCCAATGCCATACAATATGCTTACTCAACAATGGATAAGTGACAAATCGATTGCTGATGCAGTAGAGGCATTAATTGGAGCACATTTAATTCAACTTGGACAATCAGCTACTCTGAAATTTATGAATTGGTTGGGAATTAAGGTTCTGACTGATATTTCTTCATTGCCCTCACCACTTCTTCGTTTCATTGACACACCTGAAGATCCTAATCTTTCACTTAAACATCTTGCCCTTCTTTATGAGAAATTTGATTTTGCCACTGTTGAGAACAATATTGGTTATAAATTTGCAAATAAGGCATATTTAGTCCAAGCATTTACTCACGCTTCATATTATAACAATCGAGTAACCGGTTGTTACCAACGTTTGGAATTCCTTGGCGATGCTGTTTTAGATTATATGATAACTCGTTTTCTTTATGAACATAAGAAGCAATATAGTCCTGGTGTTCTTACTGATCTTCGCTCCGCTCTTGTCAATAATACAATTTTTGCTTCGCTTGCAGTTAAATATTCTTTTCACAAACATTTTGTTATGATTTGTCCTCCACTTTATCAAATGGTCGAAAAGTTTGTTAATTTCTGTAAGCAAAAAGATTTTCTTCACTGCGCGAATTTTGACGATGAAATATTTATGTTAACTGAAGATGAAATCGATGAGGAAGACCTTTTAAGTGAAGAAGATGTAGAAGTACCTAAAGCGATGGGTGATATTTTCGAATCAATTGCTGGTGCTGTTTATTTAGATTGTGGAATGGATTTAGATATTGTTTGGCGTGTATTTTATAATTTAATGAGAGATGTTATACAAAAATGTTGTGAAAATCCACCACAATCGCCAGTTAGAGAATTGTTTGAAAGGAAGAATTGTCGAGCAAAATTTTCAAAATTGGAACGAAAATTAGAAACAGGCAAAGTTAGAGTTACTGTAACGGTTAATGATAATCTCCAATTTACTGGTATGGGAAGGAGTTATCGAATTGCTAAATGTACAGCTGCTAAACGAGCACTACAACATTTAAGAAAATTAGATGCCACAAAAACAGCAAAGAATAAATAA

>FXSY01000007-*M. incognita* (36 exons)

ATGTCTCCACCGAGGGATTTTAGTGGTAAATGTATTCCTCCAAGAGATTATCAGTTTAAATTCAAGGTTGAATTACTTGATCGTGCAAAAATTCAAAACACGATTATTTCGCTCGGTACTGGTAGTGGAAAAACGTTTGTGGCGGTTCTTTTAATAAAGGAATATTCCCAGAGGTTGTTACATCAAAATGAAAAAGCTGTGTTTTTGGTTAATACTGTTGAACTTGTTGCCCAGCAAGCCGAGCATATTGAATTTCATTCAAGTTTGTCTGTCGCCCGTATTTCTGGCTCTACAATTAAAAGAAAATATGAGAGAAAAGAAGTTGAGAAGATTACTAACACCAATCAGGCTAGTCATGTGATTGTAATTACAGCTCAAGTGTTTCTTGATTTAATCAATCACGGCTTATTTGATTTCTCTTCTTTGGCCTTGTTGATTGTTGATGAATGCCATCATTGTTTGGGAGAACTGCATCCATATAGGCTGATTATGAATCATTACAAGAAACTTCAAGGTCAGCGACCACGCGTTTTGGGCCTTACAGCTTCAATTCTGAATAAAAAAGTGCCATCCTCTCGTATTGAGTGCACCGCTCAGCTGCTAGAACAAATAATGGATTCACAGATAGAAACTTCCAGCAATTATACACAGGAGCGCCTTCGTTCATTCATTGAAAAGAATGAAGATTTTCATTCAGAACTTGAAGTTGATCCGCGTCGCAAAATTTTTGAGACAATTTCCCGTTCATTTTCAACTCTTCAACAAGTTGGTAGCTGGGCAGCTCTAAAAGGTTTTATCCTTTGGCAAAAGAATTTACTTAAACATGTTGATGACCCAATAATTGGCAATAAACAAAAATGTATTTTAAGAATGGCTGAAACTGCTTTTAGAACTTGTTCAAAAGTTTTAAGTCACAAAATAAATCCGTTAAACAGCTATGACAAATTGAGTAACGTAAATAAATCATTTATCAGTGACAGAGTGCGCAAATTGTTGGAAATATTAAAAAGTTATTCGCCCTCGAAACGTGAATTGTCTGGAATTAAAGATACACTTTTTGGATTAGTTTTTGTGAAAGAACGATTTATTGCCTTTATGATTAATAATTTGCTTCGGTTTCTTGTAAAACAAAATCCTGAAGAGTTTGGACATTTAAAAGTGGACTTTATTGTTGGACATACTGGAAATTCTGAGACAGGCGATGAAGATAGGCGTTTGGTGAATCGAAAACAAGAACAAACTCTTACAAAATTCCGAAATGGCCAATTAAATTTATTAATTACGACAAATGTTTTGGAAGAAGGAATTGATTTAAGAAATTGTAATTTAGTCGTTCGATTTGATCCGCCGATGGACTTTCGTTCATTTATTCAATCTAGCGGTCGTGCTCGAAGAGAAAATAGCGCTTTTTATATACTTATTGAAGAGAAAAATTATTTGGATTTTATGATGGACTTAACAGGGTATGCACAAGCAGAAGAGTTTGTTCTGAGACGTTATCGTTCTGGCAATGATTTTACTTTGGAAGGAAATGATGAAACAAAAATATTACATCCACATCTGGATGATGCTGTTGCTCCTTATGTTGTTACAACTGAAAAAGGGACAGCTAAAGTCTCGTTAAGTAATGCTATACAGTACTGTGCAAAATTACCATCTGATATTTTTACTCGATTAGTGCCACGTTATACAATACAAACTTTATCAGAAAATGGACAAACTTTGTATATTGCCGAATTATATCTCCCAATCAATTCACCAATAAAGGAGCCAATAAAATCTAAACCGATGAATTCTAAAAGATTATCTTTAATGGCAGTCGCATTGGAGGCTTGCAAACGATTACATCAAAGAAAAGAATTAAATGACAATCTTTTGCCTGCAGGAAAGGATATCTTGGATGATCTTTTAGGTGAAGTTGATGATGATGAATATCTGCCTCATCTACCAAGCAGAATGGGATCCTCAAAGAAAAAACGTCTCTATGATAGAAAAATGTCGAAAACTCTTAATTCTACATTACCTTCGCAAGACAGTGAATGCATTCTTTACGTTATGGAAATGAAACTGGTTAAGCCCGTTACTGATGAAGGGAATCCGAAAAGGAGAAAAATTATTGATCCGTTCGAATCAAATTCGGCATTTGGATTTTTGTCTTCGAAGGAGCTCCCGAAGATTCCGGGATTTCCAGTTTTTCAACGGAATGGTGAAATGAATGTGCAAATTCGAAAAGTAAAGAATCAACAATTTCGTCCCACTTTTGAACTTTTACAACTTATTTACCTCTTTCATCAACACATCTTTGAAGATATTTTGCGTGTTGCTCGTGGTGGTGTTGTTTTTGCACCTGGTCATTCTCCAATACCACTTTTAATTGTTCCTTTGAAAAAATTAAGCAATGCCGATTTGGACTATGAAATTGACAAAGATTACCTAAATTGGGATATTCGTGAATCTCCAACTACACCTTCAGATGAAATTAGAAAACAATTCTCTTTTGAAGAATCAAACTACTTAAATGCTGTTGTTTCACCTTGGTATCGTTCTGAAGATCAGTCTGCTTTTTATTACGTAGCTGAGATAATGACTGATCAATTTCCGTCTTCTTCATTTCCTGATGAAAAATTTACTTGTTTTAATCAATATTTTATGAGTAAATATCAATTGGAGATCTACAATCAAAAACAAAATTTACTTGATGTTGACCATACATCTGCTAGAATGAATCTTTTATTACCACGAGCAATTACTGGAAAATCAGCTACTCGTTCATTAGACCCATCTCAACGTCAAATTCTTGTTCCAGAATTAGTACATATACATCCTCTTTCTGCAACTCTTTGGTCAATTATTGTTACTCTTCCTACAATTCTTTATAGACTTAATTCTTTACTGCTTGCTGATGAATTTCGTAGCAAAGTTCTTGAAGATGCTTTAAAAATTAATTCACAAACACCCGACGATTTTGAATGGAGTCCTCTTCAATATGTCACTCTAAATGATGATCTGACACAAAAAAGTATAAGAAATTTAGATCAGTTGAGAAAAATGAACAAACAAGAGAAGGAAAATGAAGTACCAATGGAGTGTGAGAATATTGAGGAGAATGTGAATGAGACAGCTGCAAGTGGAATAGTGGGTGAATTAAAAAATTTTAGATCAAATGATTTTGAAATCGGCGTTTGGGACCCTTCAGATGGCGCACAAATGTCTAACAATGATAATACTCTGAATGAACCTTCCCCAGTCATTCATAATGCACCTGTAAATGGATTAATCCCTGGTCGGAGGAACGGTCTTCGAGGAGTTATAGCTGCTAGAGACGAGGAACTCTCTGAAATTATTGCTGTCGGGAATGATACAACACTTCATAATTATGGAGATATTTCTGATGATGATGATGTTGCAGCTGAATATGATAAATTTAAATTTTTGATGGACAATAAAATGACTACAAGTGATATTGGTGATCTCGGTGAAATGGATGTTCGACCTGCTGGGTGGAATGACGACAGTAATGCCAATGTTATCCAAATTGAAAACGAAAGTCTTCCCTTGACAATATCGACAAACAATCCTCATATTAATATTGCAAGTTTAATGAATGATTTGGAGAAAAATTATGCTGCTTTTGGTACTTCAAGCCATATTTCGAATAATACTAGAACGAATACACCGATCATTTCGAGTGATACTGCTGTTGACAAAACGGCCTTGCAAAGAAAGGAGGTCCTCTTAACATATCTTACTAACAATTCGGTAAAAAGGCAAAATGAAGATATTTTACCTGAGGAAGTTTTTTGGGGAATGGATGAAGCTGAGATAGGAGAAAATATTAGCAGTGAAGCATTGACAACTTCGAGTGCTGCGGATGGAATAAATTTGGAAAGATTAGAAACAATTGGAGATTCCTTTCTTAAAATGGCTGTCACCAACTATTTCTACTATAAACACACTGAACAACATGAAGGAAAGTTAAGCTATGCTCGTTCAAAAGAAGTTTCGAATTCTCATCTTTTTTATCTTGGACGGCAACGAGGCATCCCTCTTCTAATAGAAACACTTAAATTCGATCCACATGTTAACTGGCTTCCTCCTTGTTATGCTTCTACTTCAGAATTCCATGCTGTCAATCCTTTTGATTATACAGATTTAGATGAAGATCAATGTCAAGTGCCAATGGAGGGTGTCGATACTACTGAAACTTTAGATCAACAGCAAAAGACTAATAAAGAAACAATAGCAACTGGATGGGGTACTCTTGACGATGATAAACAAAATTATAAACGTGAAAATGGAGTAGAAACTTTAACTTTTCCACAACCAACAAAATCAGAAATTCCAGACTTACCTCCAATGCCATACAATATGCTTACTCAACAATGGATAAGTGACAAATCAATTGCTGATGCAGTAGAGGCATTAATTGGAGCACATTTAATTCAACTTGGACAATCAGCTACTCTCAAATTTATGAATTGGTTGGGAATTAAGGTTCTAACTGACATTTCTTCATTGCCCTCACCACTTCTACGTTTCATTGACACACCTGAAGATCCTAATCTTTCACTTAAACATCTTGCCCTTCTTTATGAGAAATTTGATTTTGCTACTGTTGAGAACAATATTGGCTATAAATTTGCAAATAAGGCATACTTAGTCCAAGCATTTACTCACGCTTCATATTATAATAATCGAGTAACCGGTTGTTACCAGCGTTTGGAATTCCTTGGCGATGCTGTTTTAGATTATATGATAACTCGTTTTCTTTATGAACACAAGAAGCAATATAGTCCGGGTGTTCTTACTGATCTTCGCTCCGCTCTTGTCAATAATACAATTTTTGCTTCGCTTGCAGTTAAATATTCTTTTCACAAACATTTTGTTATGATTTGTCCTCCACTTTATCAAATGGTCGAAAAGTTTGTTAATTTCTGTAAGCAAAAAGATTTTCTTCATTGCGCGAATTTTGACGATGAAATATTTATGTTAACTGAAGATGAAATCGATGAGGAAGACCTTTTAAGTGAAGAAGATGTCGAAGTACCTAAAGCAATGGGTGATATTTTTGAATCAATTGCTGGCGCTGTTTATTTAGATTGTGGAATGGATTTAGATATTGTTTGGCGTGTATTTTATAATTTAATGAGAGATGTTATACAAAAATGTTGTGAAAATCCACCACAATCGCCAGTTAGAGAATTATTTGAAAGGAAGAATTGTCGAGCAAAATTCTCAAAATTGGAACGAAAATTAGAAACAGGCAAAGTTAGAGTTACTGTAACGGTTAATGATAATCTCCAATTTACTGGTATGGGAAGGAGTTATCGAATTGCTAAATGTACAGCTGCTAAACGAGCACTTCAACATTTAAGAAAATTGGATGCCACGAAAACAGCAAAGAATAAATAA

>FXSY01000995-*M. incognita* (35 exons)

ATGTCTCCACCGAAGGATTTTAGTGGTAAATGTATTTCTCCAAGGGATTATCAGGTTGAATTGCTTGATCGTGCAAAAATTCAAAACACGATTATTTCGCTCGGTACTGGTAGTGGAAAAACGTTTGTGGCGGTTCTTTTAATAAAGGAATATTCTCAGAGATTGTTACATCAAAATGAAAAAGCTGTGTTTTTGGTTAATACTGTTGAACTTGTTGCCCAGCAAGCCGAGCACATTGAATTTCATTCAAGTTTGTCTGTCGCTCGTATTTCTGGCTCTACAATTAAAAGAAAATATGAGAGAAAAGAAGTTGAGAAAATTACTAGCAGCAATCAGGCAAGTCATGTATTATTAAAGTCTAGTTTATTTTTTGTTTTAAAGGTGATTGTAATTACAGCTCAAGTGTTTCTCGATTTAATTAATCACGGGTTATTCGATTTCTCTTCTTTGGCCTTGTTGATTGTTGATGAATGCCATCATTGTTTGGGAGAACTGCATCCATATAGGCTTATTATGAATCATTACAAGAAACTGCAAGGTCAACGACCACGCGTTTTGGGCCTTACAGCTTCAATTCTGAATAAAAAAGTGCCATCCTCTCGTATTGAGTGCACCGCTCAGCTGCTAGAACAAATAATGGATTCGCAGATAGAGACCTCCAGCAATTATACGCAGGAGCGCCTTCGTTCATTTATTGAAAAGAATGAAGATTTTCATTCAGAACTTGAAGTTGATCCGCGTCGCAAAATTTTTGAGACAATTTCCCGTTCATTTTCAACTCTTCAACAAGTTGGTAGCTGGGCAGCTCTAAAAGGTTTTATCCTTTGGCAAAAGAATTTACTTAAACATGTTGATGACCCAATAATTGGCAATAAACAAAAATGTATTTTAAGAATGGCCGAGACTGCTTTTAGAACTTGTTCAAAAGTTTTAAGTCACAAAATAAATCCGTTAAACAGCTATGACAAATTGGTTAACGTAAATAAATCATTTATCAGTGATAGAGTGCGCAAATTGTTGGAAATATTAAAAAGTTATTCACCCTCAAAACGTGAATTGTCTGGAATTAAAGATACACTTTTTGGATTAGTTTTTGTGAAAGAACGATTTATTGCCTTTATGATTAATAATTTGCTTCGGTTTCTTGTAAAACAAAATCCTGAAGAGTTTGGACATTTAAAAGTGGACTTTATTGTTGGACACACTGGAAATTCTGAGACAGGAGATGAAGATAGGCGTTTGGTGAATCGAAAACAAGAACAAACTCTTACAAAATTCCGAAATGGTCAATTAAATTTATTAATTACGACAAATGTTTTGGAAGAAGGAATTGATTTAAGAAATTGTAATTTAGTCGTTCGATTTGATCCGCCGATGGATTTTCGGTCATTTATTCAATCTAGCGGTCGTGCTCGAAGAGAAAATAGCGCTTTTTATATACTTATTGAAGAGAAAAATTATTTGGAGTTTATGATGGACTTAACAGGATATGCACAAGCAGAAGAGTTTGTTTTGCGGCGTTATCGTTCTGGCAATGATTTTACTCTGGAAGGAAATGATGAAACAAAATTATTACATCCACATCTGGATGATGCTGTTGCTCCTTATGTTGTTACAACTGAAAAAGGGACAGCTAAAGTCTCATTAAGTAATGCTATACAATATTGTGCAAAATTACCATCTGATATTTTTACTCGATTAGTGCCTCGTTATACAGTACAAACTTTATCAGAAAATGGACAAACTTTGTATATTGCTGAATTATACCTTCCAATCAATTCACCAATAAAGGAGCCAATAAAATCTAAGCCGATGACTTCTAAAAGATTATCTTTAATGGCAGTCGCTTTGGAGGCTTGCAAACGATTACATCAAAGAAAAGAATTAAATGACAATCTTTTGCCTGCAGGAAAAGATATCTTGGATGATCTTTTAGGGGAAGTTGATGATGATGAATATCTGCCTCATTTACCAAGCAGAATGGGATCCTCAAAGAAAAAACGTCTCTACGATAGAAAAATGTCGAAAACTCTTAATTCTACATTACCTTCGCAAGACAGTGAATGCGTTCTTTACGTTATGGAAATGAAACTGATTAAGCCCGTTACTGATGAAGGGAATCCGAAAAGGAGAAAAATTATTGATCCGTTCGAGTCAAATTCGGCGTTTGGCTTTTTGTCTTCGAAGGAGCTCCCGAAGGTTCCGGGATTTCCAGTTTTTCAACGGAATGGTGAAATGAATGTGCAAATTCGTAAAGTAAAGAATCAGCAATTTCGTCCCACTTTTGAACTTTTACAACTTATTTACCTCTTTCATCAACACATCTTTGAAGATATTTTGCGTGTTGCCCGTGGTGGTGTTGTTTTTGCACCTGGTCATTCTCCAATACCACTTTTAATTGTTCCCTTGAAAAAATTAAGCACTGCCGATTTGGATTATGAAATTGACAGAGATTACCTAAATTGGAATATCCGAGAATCTCCAACTACACCTTCAGATGAAATTAGAAAACAATTTTCATTTGAAGAATCAAACTACTTAAATGCTGTTGTTTCACCTTGGTATCGTTCTGAAGATCAGTCTGCTTTTTATTACGTAGCCGAGATAATGACTGATCAATTTCCGTCTTCTTCATTTCCTGATGAAAAATTTGCTTGTTTTAATCAATACTTTATGAGTAAATATCAATTGGAGATCTACAATCAAAAACAAAATTTACTTGATGTTGACCATACATCTGCTAGAATGAATCTTTTATTACCACGAGCAATTTCTGGAAAATCAGCTACTCGATCATTGGACCCATCTCAACGTCAAATTCTTGTCCCAGAATTGGTACATATACATCCTCTTTCTGCAACTCTTTGGTCAATTATTGTTACTCTTCCTACAATTCTTTATAGACTTAATTCTTTACTGCTTGCTGATGAATTTCGTAGCAAAGTTCTTGAAGACGCTTTGAAAATTAATTCACAAACACCCGACGATTTTGAATGGAGTCCTCTTCAATATGTCACTCTAAATGATGATCTGACACAAAAAAGTATAAGAAATTTAGATCAGTTGAGAAAAATGAACAAACAAGAGAAGGAAAATGAAGTACCAATGGAGTGTGACATTATTGAAGAGAATGTGAATGAGACAGCAGCAAGTGGAATAGTGGGTAGATTAAAATTTTTGAAATCAAATGATTTTGAAATCGGCGTTTGGGACCCTTCAGATGGCGCACAAATGTCTAACAATGATAATACTCCGAATGAACCTTCTCCAGTCATCCATAATGCACCTGTAAATGGATTAATCCCTGGGCGGAGGAATGGTCTTCGAGGAGTTATAGCTGCTAGAGACGAGGAACTCTCAGAAATTATTGCTGTTGGTAATGATACAACGCTTCATAATTATGGAGATATTTCTGATGATGATGATGTTGCAGCTGAATACGATAAATTTAAATTTTTGATGGACAATAAAATGACTACAAGTGATATTGGTGATCTAGGTGAAATGGATGTTCGACCTGCTGGTTGGAATGACGATAGCAATGCCAATGTTATCCAAATTGAAAATGAAAGTCTTCCCTTGACAATATCGACAAACAATCCTCATATTAATATTGCAAGTTTAATGAATGATTTGGAGAAAAATTATGCTGCTTTTGGTACTTCAAACCATATTTCCAATAATAATAAAACGAATACACCGAACATTCCGAATGCTACTGCTGACAAAACGGACTTGCAAAAAAAGGAGGTCCTCTTAACATATCTTATTAACAATTCGGTAAAAAGGCAAAATGAAGATATTTTACCTGAGGAAGTTTTTTGGGGAATGGATGAAGCTGAGATAGGAGAGAATATTAGCAGTGAAGCATTGACAACTTCAAGTGCTGCGGATGGAATAAATTTGGAAAGGTTGGAAACAATTGGAGATTCTTTCCTCAAGATGGCTGTCACCAACTATTTCTACTATAAACACACGGAACAACATGAAGGAAAGTTAAGCTATGCACGTTCAAAAGAAGTTTCGAATTCCCATCTTTTTTATCTTGGACGGCAACGAGGAATCCCTCTTTTAATAGAAACACTGAAATTCGATCCACATGTTAACTGGCTTCCTCCTTGTTATGCTTCTACTTCAGAATTCCATGCTGTCAATCCTTTTGATTATACAGATTTAGATGAAGATCAATGTGCAGTGCCAATGGAGGGTGTTGATACTACAGAAACTGTAGATCAACAGCAAAAGATTAATAAAGAAACAATAGCAACTGGATGGGGAACTATTGACGATGATAAACAAAATTATAAACGTGAAAATGGAGTAGAAACTTTAACTTTTCCACAACCAACAAAATCAGAAATTCCTGACTTACCTCCAATGCCATACAATATGCTTACTCAACAATGGATAAGTGACAAATCGATTGCTGATGCAGTAGAGGCATTAATTGGAGCACATTTAATTCAACTTGGACAATCAGCTACTCTGAAATTTATGAATTGGTTGGGAATTAAGGTACTGACTGATATTTCTTCATTGCCCTCACCACTTCTTCGTTTCATTGACACACCTGAAGATCCTAATCTTTCACTTAAACATCTTGCCCTTCTTTATGAGAAATTTGATTTTGCCACTGTTGAGAACAATATTGGTTATAAATTTGCAAATAAGGCATATTTAGTCCAAGCATTTACTCACGCTTCATATTATAACAATCGAGTAACCGGTTGTTACCAACGTTTGGAATTCCTTGGCGATGCTGTTTTAGATTATATGATAACTCGTTTTCTTTATGAACATAAGAAGCAATATAGTCCTGGTGTTCTTACTGATCTTCGCTCCGCTCTTGTCAATAATACAATTTTTGCTTCGCTTGCAGTTAAATATTCTTTTCACAAACATTTTGTTATGATTTGTCCTCCACTTTATCAAATGGTCGAAAAGTTTGTTAATTTCTGTAAGCAAAAAGATTTTCTTCATTGCGCGAATTTTGACGATGAAATATTTATGTTAACTGAAGATGAAATCGATGAGGAAGACCTTTTAAGTGAAGAAGATGTAGAAGTACCTAAAGCGATGGGTGATATTTTCGAATCAATTGCTGGTGCTGTTTATTTAGATTGTGGAATGGATTTAGATATTGTTTGGCGTGTATTTTATAATTTAATGAGAGATGTTATACAAAAATGTTGTGAAAATCCACCACAATCGCCAGTTAGAGAATTGTTTGAAAGGAAGAATTGTCGAGCAAAATTTTCAAAATTGGAACGAAAATTAGAAACAGGCAAAGTTAGAGTTACTGTAACGGTTAATGACAATCTCCAATTTACTGGTATGGGAAGGAGTTATCGAATTGCTAAATGTACAGCTGCTAAACGAGCACTACAACATTTAAGAAAATTAGATGCCACAAAAACAGCAAAGAATAAATAA

>NXFT01000985-*M. graminicola* (35 exons)

ATGTCTCCGCCAAAAGATTTTATTGGTAAATGTATTCCTCCAAGAGATTATCAGGTTGAGTTGCTAGATCGTGCTAAAATCCAAAATACGATTATTTCGCTCGGCACAGGAGCAGGAAAAACATTTGTAGCTGTTCTTTTGATAAAAGAATATTCTCAAAGGTTGCTTCACCGTAATGAAAAAGCTGTGTTTTTGGTTAATACTGCTTTGCTTACCTGTCAACAGGCTGATCATATTGAATTGCACTCAAGTTTGTCTGTCGCCCGAGTTTCAGGTTCTACAATTAAAGGGAAATATGATAGGGAAAAAGTCGAAAAAATTACTAAAGAGAATCAGGTTATTGTAATTACGGCGCAGGTTTTCCTCGATTTTATCAATCATGGGCTTTTCGATTTAACATCTTTGGCTTTATTAATTGTTGATGAATGCCATCATTGTCTGGGCGAGACACATCCATACAGGCTTATTATGAATCATTACATGAAGTTACAAGGTTTTCAACAACGTCCCCGTGTTTTGGGATTGACAGCCTCAATACTAAATAAAAAAGTGCCATTCTCTCGTATTGAATTTACTGCTCAACTTTTGGAAAAAATAATGGATTCACGTTTAGAAACTGCTAGCAGTTATACCCAGGACCGTCTTCGTTCATTTGTCGAAAAAAATGAGGATTTTCATTCAGAATTAGAGGCTGATCCTCGTCGTCCAATTTTACAAGCAATTTCTCGCTCATTTTCTACACTTCAACAAGTTGGTGGTTGGGCAGCCTTAGAAGGCTTTGTTATATGGCAGAAAAACTTGCTTAAACTTGCCGATGATTCTAACATTGGAACGAAACAAAAATGTATTCTTCGAATGGCTGAAACAACTTTTAGAACGTGTTCAAAGGTTTTAAGTCACAAAATAAAGTCATTGGACAGTTATGATAAACTGGCTGATTCTAAAAAATCTTTTATCAGCGATAGAATACGTAAATTGGTCGAAATTTTAAAAATGTATTCACCATTAAAACGTGAGAAGCATGGAATTAAGGATGCGCTTGCCGGACTTGTTTTTGTCAAGGAACGCTTCATTGCTTCCATGATTAATAAGCTGCTTCGTTATCTTGCAAAGAAATATCATGAAGATTTTGGCTATTTAAAAGTAGATTTTATTGTTGGAAACACTGGGAGTTTGGAGACTGGAGATGAAGATCGTCGCTTAGCAAATCGCAAACAAGAACAAACTCTTCGTGATTTCAAGAATGGTCAATTAAACCTCTTGATTACAACAAGTGTTTTAGAAGAAGGTATTGACTTGAGGAATTGCAATTTGGTTGTTCGATTCGATCCCCCAATGGATTTTTGTTCTTATGTTCAGTCCAGTGGGCGTGCTAGAAAAGAGAATGGCGCATTTTACATGCTAATTGAAGAGAAAAATTATGTTGATTTTATGATGGATTTAACTAAATATGCGCAGGCTGAAGAGCTTGTTTTGCGACGTTATCGTTCTGGAAATGATTTTATTTTGAATGATAAAGAGGAAATGAATGATAAAATGATTTTACAACCACATATCGATGATGTAGTTTCTCCTTATGTTGTTACCTCTGCAAATGGAACTGCTAAAGTTTCTTTGAGTGGTGCCATACAGCTGGTGAACAGATATTGTTCAAAATTGCCATCAGATATTTTTACTCGATTGGTGCCACGTTATAGTATACAAACAATTTCTGAAGCTGGACAACAAATGTATATCGCGGAATTAATTTTACCAATTAATTCGCCTATAAAAGAGACAATAACCACTAAACCTATGTCTTCAAAAAAATTATCCTTGATGGCAGCTGCATTGGAGGCATGCAAACGTTTACATGAAAGGAAGGAATTGAATGATCAACTTTTACCCACTGGAAAAGAAATTGTTTTAGACCTTTTGGGTGAAGTAGATGATGATGAATATTTGCCTTATTTACCTAGTAAAATGGGCTCTTCAAAGAAAAAACGACTCTACGATAGAAAAATGTCCAAAACACTTGATTCTACTTTACCATCTCAAGAAACTGATTGTTTTCTTTATATTATGGAGATGAAGCTAGTTAAACCTGTTAGTGAAGAGCGGAATCCAAAAAGGCGGAAGATAATTGATCCATTCGAGTCAAATTCCGCCTTTGGATTTTTGTCTTCTAAAGAGCTGCCGAAAATTCCCGGATTTCCCGTTTTTCAACGGCATGGAGAAATGCTTGTACAATTTCGTAAAGCGAAAAAACCTGTTAACCTTACTTTTGAACTTTTTCAACTCATTTGTTTATTTCATCAGCATATATTTGAAGATATTCTGCGTGTTGCTCGTGGAGGTGTTGTTTTTTCGCCTGGACATTCTCCAATGCCATTATTGATTGTACCGTTGAAAAAAGTTGGGACTGTCGATATGGATTATGAGATCGATAGAGATTATCTCAACTGGGATATTCGCGAATCTTCAACCATTCCATCTGACGAAATTCGAAAACAGTTTGTTTTTGACGAGTCTCGCTATTTAAATGCAGTTGTTTCACCATGGTATAGATCTGAAGATCAATCAGCTTTTTATTACGTTGCCGAGATTATGTCTGATCAATTTCCATCTTCTTCATTCCCTGATGAAAAGTTCACTTGTTTCAATCAGTACTTTATGAGTAAATATCAGTTGGAGATTTACAATCAAAAACAAAATTTACTTGACGTTGACCATACATCAGCTAGAATGAATCTTTTATTACCACGATCATTAACTGGAAAGTCGACAACTCGTTCGTTAGATCCTTCTCAACGTCAAATTCTTATCCCAGAATTAGTACATATTCACCCTCTTTCGGCAACTCTTTGGTCATTTATAGTTACACTTCCCACAATTCTCTATAGGCTTAATTTTTTGTTGCTTGCTGATGAATTTCGCAGCAAGGTTCTCGAGGATGCGCTTAAACTTAGCTCTCAAACGCCTGATGATTTTGAATGGAATCCACTTCAATATGTTACTTCAATTGATGGTCAAGCTGAGAAAAGCATACGAAACTTAGATCAACTTAGAAAAATGAATCAATTGGAAAAAGAAAATGAATTAATTATGGAATGTGATAATAATGAAAGTAAAACTTCTGAAGCAATCCAGGATGAAAATGAAATGAATGATTTTGAAATTGGAGTTTGGGATCCATCAGATGCTGCTCAATTCTCAAATGATGCTAATACTCAAAATGAACCTACTCAAGTTATTCATAATGCACCTATAAACGGTTTAATTAATGGACGGAGGAATGGATTAAGAGGAATTCTAGCTGCTAGAGATGAGGAACTTTCAGAAATAATTGCTGTTGGTAATGATACTACAATATATAATTATGGTGATATTTCTGATGATGATGATGTTGCGGCAGAATATGATAAATTTAAATTTTCGATGCACAACAAAATGACGACTAGTGATATTGGTGAAATTGGAGAAATTTCTCATGTTCGTCCTGCTGGGTGGGACGACAACAATGCCAACGTAATTCAAATTGATAACGAAAACCTTCCATTGACTATCTCAACAAATAACCCTCATATTAACATTGCAAGTTTAATGAATGATTTGGAGAGAAATTATGCTGCTTTTGGTACATCAACTACCAACGTTGTGAATAATAAAGATGTCGCTATGCAAAGTACTCTTAAGAAGAAAGAGTATAATTTTGATTCATTTGATGTTATGGACCAAAATGATCCATCGATAAAAAAACAGAATGAGGAAATTTTGCCCGAGGAAGTTTTTTGGGGGATAGATGAGAATGAGATTGATGAGAATATTAATAATGAGGCATTAACAACCTCAAGTGCGGCTGGAGGGATTAATTTAGAAAGGTTGGAAACCATTGGAGATTCATTTCTTAAAATGGCTGTAACTAATTATTTTTATCATAAACATACTGAACAACATGAGGGCAAATTAAGCTTTGCTCGTTCCAAAGAGGTTTCCAATTCTCATCTCTTCTATCTTGGAAGACAACGTGGTATTCCTCTTTTAATGGAAACACTAAAGTTCGATCCGCATGTAAATTGGCTTCCTCCTTGCTATGCTTCTACTTCAGAATTTCATGCTGTAAATCCATTTGATTACACTGATTTGGACGAAGAACAAAATCAACGTGTATTGCCAATGGAAGGAATTGAAACAATTAAAAACGTTGACCAAAAACAAAAAAATAAAGAAACAGTTGCAACAGGGTGGGGTACTCTTGACGATGAAAAACAGAATTATAAATATGAAAATGGAATAGAAACATTAACTTTTCCGCAGCCAACCAAATCTGAAATTCCTGATTTACCTCCAATGCCTTACAATATGCTTACTCAGCAATGGATAAGTGATAAATCAATTGCTGATGCAGTAGAAGCTTTAATTGGAGCACATTTAATTCAACTTGGACAATCAGCTACACTTAAATTTATGAATTGGTTGGGAATTAAACCAAATCGTTCACTTAGACGTCTTGCTTTACTTTACGAGAAATTTGATTTTGCGACGGTTGAAAACAATATTGGTTATAAATTTGCAAATAAGGCATATTTAGTTCAGGCATTTACACACGCTTCGTATTATGATAATAGAGTAACTGGCTGTTACCAACGTTTGGAATTTCTTGGTGATGCAGTCCTTGATTATATGATTACTCGATTTCTTTATGAACACAAAAAACAGTATAGCCCTGGTGTACTTACTGATCTTCGTTCAGCTCTTGTTAACAACACAATTTTTGCTTCTTTAGCAGTCAAATATTCCTTCCATAAACATTTTATTATGATTTGCCCTCCGCTTTATCAAATGATTGAAAAATTTGTCAATTTCTGCAAACAAAAAGATTTTATGCATTGCGCAAATTTTGACGATGAAATCTTTATGTTAACTGAAGAAGAAATTGATGAGGAAGAGCTTTTAAGTGAGGAAGATGTAGAAGTTCCTAAAGCTATGGGTGATGTATTTGAATCAATGGCTGGTGCGGTTTATTTAGATTGTGGAATGAATTTGGATATTGTTTGGCGTGTTATGTATAACTTAATGAGGGATGTTATACAAAAATGTTGTGAAAATCCACCACAATCACCCGTTAGAGAATTATTTGAAAGGAAGAATTGTCGTGCAAAATTCTCTAAATTGGAACGTAAATTGGAAACTGGAAAAGTTCGAGTCACAGTAACAATTAACGAAAATCTTCAATTCACGGGTATGGGACGTAGTTATCGGATTGCTAAGTGTACAGCTGCTAAACGGGCACTGCAACATTTGCGCAAATTAGATTCTGCAAAAAATGTGTCGTCAACAAATAAATAA

>RCFK01001432-*M. javanica* (35 exons)

ATGTCTCCACCGAAGGATTTTAGTGGTAAATGTATTCCTCCAAGGGATTATCAGGTTGAATTGCTTGATCGTGCAAAAATTCAAAACACGATTATTTCGCTCGGTACTGGTAGTGGAAAAACGTTTGTGGCGGTTCTTTTAATAAAGGAATATTCTCAGAGATTGTTACATCAAAATGAAAAAGCTGTGTTTTTGGTTAATACTGTTGAACTTGTTGCCCAGCAAGCCGAGCACATTGAATTTCATTCAAGTTTGTCTGTCGCTCGTATTTCTGGCTCTACAATTAAAAGAAAATATGAGAGAAAAGAAGTTGAGAAAATTACTAGCAGCAATCAGGCAAGTCATGTATTATTAAAGTCTAGTTTATTTTTTGTTTTAAAGGTGATTGTAATTACAGCTCAAGTGTTTCTTGATTTAATTAATCACGGGTTATTCGATTTCTCTTCTTTGGCCTTGTTGATTGTTGATGAATGCCATCATTGTTTGGGAGAACTGCATCCATATAGGCTTATTATGAATCATTACAAGAAACTGCAAGGTCAACGACCACGCGTTTTGGGCCTTACAGCTTCAATTCTGAATAAAAAAGTGCCATCCTCTCGTATTGAGTGCACCGCTCAGCTGCTAGAACAAATAATGGATTCGCAGATAGAGACCTCCAGCAATTATACGCAGGAGCGCCTTCGTTCATTTATTGAAAAGAATGAAGATTTTCATTCAGAACTTGAAGTTGATCCGCGTCGCAAAATTTTTGAGACAATTTCCCGTTCATTTTCAACTCTTCAACAAGTTGGTAGCTGGGCAGCTCTAAAAGGTTTTATCCTTTGGCAAAAGAATTTACTTAAACATGTTGATGACCCAATAATTGGCAATAAACAAAAATGTATTTTAAGAATGGCCGAGACTGCTTTTAGAACTTGTTCAAAAGTTTTAAGTCACAAAATAAATCCGTTAAACAGCTATGACAAATTGGTTAACGTAAATAAATCATTTATCAGTGATAGAGTGCGCAAATTGTTGGAAATATTAAAAAGTTATTCACCCTCAAAACGTGAATTGTCTGGAATTAAAGATACACTTTTTGGATTAGTTTTTGTGAAAGAACGATTTATTGCCTTTATGATTAATAATTTGCTTCGGTTTCTTGTAAAACAAAATCCTGAAGAGTTTGGACATTTAAAAGTGGACTTTATTGTTGGACACACTGGAAATTCTGAGACAGGAGATGAAGATAGGCGTTTGGTGAATCGAAAACAAGAACAAACTCTTACAAAATTCCGAAATGGTCAATTAAATTTATTAATTACGACAAATGTTTTGGAAGAAGGAATTGATTTAAGAAATTGTAATTTAGTCGTTCGATTTGATCCGCCGATGGATTTTCGGTCATTTATTCAATCTAGCGGTCGTGCTCGAAGAGAAAATAGCGCTTTTTATATACTTATTGAAGAGAAAAATTATTTGGAGTTTATGATGGACTTAACAGGATATGCACAAGCAGAAGAGTTTGTTTTGCGGCGTTATCGTTCTGGCAATGATTTTACTCTGGAAGGAAATGATGAAACAAAATTATTACATCCACATCTGGATGATGCTGTTGCTCCTTATGTTGTTACAACTGAAAAAGGGACAGCTAAAGTCTCATTAAGTAATGCTATACAATATTGTGCAAAATTACCATCTGATATTTTTACTCGATTAGTGCCTCGTTATACAATACAAACTTTATCAGAAAATGGACAAACTTTGTATATTGCTGAATTATACCTTCCAATCAATTCACCAATAAAGGAGCCAATAAAATCTAAGCCGATGACTTCTAAAAGATTATCTTTAATGGCAGTCGCTTTGGAGGCTTGCAAACGATTACATCAAAGAAAAGAATTAAATGACAATCTTTTGCCTGCAGGAAAAGATATCTTGGATGATCTTTTAGGGGAAGTTGATGATGATGAATATCTGCCTCATTTACCAAGCAGAATGGGATCCTCAAAGAAAAAACGTCTCTACGATAGAAAAATGTCGAAAACTCTTAATTCTACATTACCTTCGCAAGACAGTGAATGCGTTCTTTACGTTATGGAAATGAAACTGATTAAGCCCGTTACTGATGAAGGGAATCCGAAAAGGAGAAAAATTATTGATCCGTTCGAGTCAAATTCGGCGTTTGGCTTTTTGTCTTCGAAGGAGCTCCCGAAGGTTCCGGGATTTCCAGTTTTTCAACGGAATGGTGAAATGAATGTGCAAATTCGTAAAGTAAAGAATCAGCAATTTCGTCCCACTTTTGAACTTTTACAACTTATTTACCTCTTTCATCAACACATCTTTGAAGATATTTTGCGTGTTGCCCGTGGTGGTGTTGTTTTTGCACCTGGTCATTCTCCAATACCACTTTTAATTGTTCCCTTGAAAAAATTAAGCACTGCCGATTTGGATTATGAAATTGACAGAGATTACCTAAATTGGAATATCCGAGAATCTCCAACTACACCTTCAGATGAAATTAGAAAACAATTTTCTTTTGAAGAATCAAACTACTTAAATGCTGTTGTTTCACCTTGGTATCGTTCTGAAGATCAGTCTGCTTTTTATTACGTAGCCGAGATAATGACTGATCAATTTCCGTCTTCTTCATTTCCTGATGAAAAATTTGCTTGTTTTAATCAATACTTTATGAGTAAATATCAATTGGAGATCTACAATCAAAAACAAAATTTACTTGATGTTGACCATACATCTGCTAGAATGAATCTTTTATTACCACGAGCAATTTCTGGAAAATCAGCTACTCGATCATTGGACCCATCTCAACGTCAAATTCTTGTCCCAGAATTGGTACATATACATCCTCTTTCTGCAACTCTTTGGTCAATTATTGTTACTCTTCCTACAATTCTTTATAGACTTAATTCTTTACTGCTTGCTGATGAATTTCGTAGCAAAGTTCTTGAAGACGCTTTGAAAATTAATTCACAAACACCCGACGATTTTGAATGGAGTCCTCTTCAATATGTCACTCTAAATGATGATCTGACACAAAAAAGTATAAGAAATTTAGATCAGTTGAGAAAAATGAACAAACAGGAGAAGGAAAATGAAGTACCAATGGAGTGTGACATTATTGAAGAGAATGTGAATGAGACAGCAGCAAGTGGAATAGTGGGTAGATTAAAATTTTTGAAATCAAATGATTTTGAAATCGGCGTTTGGGACCCTTCAGATGGCGCACAAATGTCTAACAATGATAATACTCCGAATGAACCTTCTCCAGTCATCCATAATGCACCTGTAAATGGATTAATCCCTGGGCGGAGGAATGGTCTTCGAGGAGTTATAGCTGCTAGAGACGAGGAACTCTCAGAAATTATTGCTGTTGGTAATGATACAACGCTTCATAATTATGGAGATATTTCTGATGATGATGATGTTGCAGCTGAATATGATAAATTTAAATTTTTGATGGACAATAAAATGACTACAAGTGATATTGGTGATCTAGGTGAAATGGATGTTCGACCTGCTGGTTGGAATGACGATAGCAATGCCAATGTTATCCAAATTGAAAATGAAAGTCTTCCCTTGACAATATCGACAAACAATCCTCATATTAATATTGCAAGTTTAATGAATGATTTGGAGAAAAATTATGCTGCTTTTGGTACTTCAAACCATATTTCCAATAATAATAAAACGAATACACCGAACATTCCGAATGCTACTGCTGACAAAACGGACTTGCAAAAAAAGGAGGTCCTCTTAACATATCTTATTAACAATTCGGTAAAAAGGCAAAATGAAGATATTTTACCTGAGGAAGTTTTTTGGGGAATGGATGAAGCTGAGATAGGAGAGAATATTAGCAGTGAAGCATTGACAACTTCAAGTGCTGCGGATGGAATAAATTTGGAAAGGTTGGAAACAATTGGAGATTCTTTCCTCAAGATGGCTGTCACCAACTATTTCTACTATAAACACACGGAACAACATGAAGGAAAGTTAAGCTATGCACGTTCAAAAGAAGTTTCGAATTCCCATCTTTTTTATCTTGGACGGCAACGAGGAATCCCTCTTTTAATAGAAACACTGAAATTCGATCCACATGTTAACTGGCTTCCTCCTTGTTATGCTTCTACTTCAGAATTCCATGCTGTCAATCCTTTTGATTATACAGATTTAGATGAAGATCAATGTGCAGTGCCAATGGAGGGTGTTGATACTACAGAAACTGTAGATCAACAGCAAAAGATTAATAAAGAAACAATAGCAACTGGATGGGGAACTATTGACGATGATAAACAAAATTATAAACGTGAAAATGGAGTAGAAACTTTAACTTTTCCACAACCAACGAAATCAGAAATTCCGGACTTACCTCCAATGCCATACAATATGCTTACTCAACAATGGATAAGTGACAAATCGATTGCTGATGCAGTAGAGGCATTAATTGGAGCACATTTAATTCAACTTGGACAATCAGCTACTCTGAAATTTATGAATTGGTTGGGAATTAAGGTTCTGACTGATATTTCTTCATTGCCCTCACCACTTCTTCGTTTCATTGACACACCTGAAGATCCTAATCTTTCACTTAAACATCTTGCCCTTCTTTATGAGAAATTTGATTTTGCCACTGTTGAGAACAATATTGGTTATAAATTTGCAAATAAGGCATATTTAGTCCAAGCATTTACTCACGCTTCATATTATAACAATCGAGTAACCGGTTGTTACCAACGTTTGGAATTCCTTGGCGATGCTGTTTTAGATTATATGATAACTCGTTTTCTTTATGAACATAAGAAGCAATATAGTCCTGGTGTTCTTACTGATCTTCGCTCCGCTCTTGTCAATAATACAATTTTTGCTTCGCTTGCAGTTAAATATTCTTTTCACAAACATTTTGTTATGATTTGTCCTCCACTTTATCAAATGGTCGAAAAGTTTGTTAATTTCTGTAAGCAAAAAGATTTTCTTCATTGCGCGAATTTTGACGATGAAATATTTATGTTAACTGAAGATGAAATCGATGAGGAAGACCTTTTAAGTGAAGAAGATGTAGAAGTACCTAAAGCGATGGGTGATATTTTCGAATCAATTGCTGGTGCTGTTTATTTAGATTGTGGAATGGATTTAGATATTGTTTGGCGTGTATTTTATAATTTAATGAGAGATGTTATACAAAAATGTTGTGAAAATCCACCACAATCGCCAGTTAGAGAATTGTTTGAAAGGAAGAATTGTCGAGCAAAATTTTCAAAATTGGAACGAAAATTAGAAACAGGCAAAGTTAGAGTTACTGTAACGGTTAATGATAATCTCCAATTTACTGGTATGGGAAGGAGTTATCGAATTGCTAAATGTACAGCTGCTAAACGAGCACTACAACATTTAAGAAAATTAGATGCCACAAAAACAGCAAAGAATAAATAA

>RCFK01001654-*M. javanica* (35 exons)

ATGTCTCCACCGAAGGATTTTAGTGGTAAATGTATTCCTCCAAGAGATTATCAGTTTAAATTCAAGGTTGAATTACTTGATCGTGCAAAGATTCAAAACACGATTATTTCGCTCGGTACTGGGAGTGGAAAAACATTTGTGGCGGTTCTTTTAATAAAAGAATATTCTCAGAGATTGTTACATCAAAATGAAAAAGCTGTGTTTTTAGTTAATACTGTTGAACTTGTTGCCCAGCAAGCCGAGCACATTGAATTTCATTCAAGTCTGTCTGTCGCCCGTATTTCTGGCTCTACAATTAAAAGAAAATATGAGAGAAAAGAAGTTGAGAAAATTACTAGCAGCAATCAGGCAAGTCATGTATTATTAAAGTTTAGTTTATTTTTTGTTTTAAAGGTGATTGTAATTACAGCTCAAGTGTTTCTTGATTTAATCAATCACGGCTTATTCGATTTCTCTTCTTTGGCCTTGTTGATTGTTGATGAATGCCATCATTGCTTGGGAGAACTGCATCCATATAGGCTGATTATGAATCATTACAAGAAACTGCAAGGTCAACGACCACGCGTTTTGGGCCTTACAGCTTCAATTCTGAATAAAAAAGTGCCATCCTCTCGTATTGAGTGCACCGCTCAGCTGCTAGAACAAATAATGGATTCACAGATAGAAACTTCCAGCAATTATACACAGGAGCGCCTTCGTTCATTTATTGAAAAGAATGAAGATTTTCATTCAGAACTTGAAGTTGATCCGCGTCGCAAAATTTTTGAGACAATTTCCCGTTCATTTTCAACTCTTCAACAAGTTGGTAGCTGGGCAGCTCTAAAAGGTTTTATCCTTTGGCAAAAGAATTTACTTAAACATGTTGATGATCCAATAATTGGCAATAAACAAAAATGTATTTTAAGAATGGCTGAAACTGCTTTTAGAACTTGCTCAAAAGTTTTAAGTCACAAAATAAATCCGTTAAACAGCTATGACAAATTGAGTAACGTAAATAAATCATTTATCAGTGACAGAGTGCGCAAATTGTTGGAAATATTAAAAAGTTATTCGCCCTCGAAACGTGAATTGTCTGGAATTAAAGATACACTTTTTGGATTAGTTTTTGTGAAAGAACGATTTATTGCCTTTATGATTAATAATTTGCTTCGGTTTCTTGTAAAACAAAATCCTGAAGAGTTTGGACATTTAAAAGTGGACTTTATTGTTGGACATACTGGTAATTCTGAGACAGGAGATGAAGATAGGCGTTTGGTGAATCGAAAACAAGAACAAACTCTTACAAAATTCCGAAATGGCCAATTAAATTTATTAATTACGACAAATGTTTTGGAAGAAGGAATTGATTTAAGAAATTGTAATTTAGTCGTTCGATTTGATCCGCCGATGGACTTTCGTTCATTTATTCAATCTAGCGGTCGTGCTCGAAGAGAAAATAGCGCTTTTTATATACTTATTGAAGAGAAAAATTATTTGGATTTTATGATGGACTTAACAGGATATGCACAAGCAGAAGAGTTTGTTTTGCGGCGTTATCGTTCTGGCAATGATTTTACTTTGGAAGGAACTGATGAAACAAAGATAATACATCCACATCTGGATGATGCTGTTGCTCCTTATGTTGTTACAACTGAAAAAGGGACAGCTAAAGTCTCATTAAGTAATGCTATACAATACTGTGCAAAATTACCATCTGATATTTTTACTCGACTAGTGCCACGTTATACAATACAAACTTTATCAGAAAATGGACAAACTTTGTATATTGCCGAATTATATCTTCCAATCAATTCACCAATAAAGGAGCCAATAAAATCTAAACCGATGAATTCTAAAAGATTATCTTTAATGGCAGTCGCTTTGGAGGCTTGCAAACGATTACATCAAAGAAAAGAATTAAATGACAATCTTTTGCCTGCAGGAAAAGATATCTTGGATGATCTTTTAGGTGAAGTAGATGATGACGAATATCTGCCACATTTACCAAGCAGAATGGGATCCTCAAAGAAAAAACGTCTCTATGATAGAAAAATGTCGAAAACTCTTAATTCTACATTACCTTCGCAAGACAGTGCATGCTTTCTTTACGTTATGGAAATGAAACTGATTAAGCCCGTTACAGATGAAGGGAATCCGAAAAGGAGAAAAATTATTGATCCGTTCGAGTCAAATTCGGCGTTTGGCTTTTTGTCTTCGAAGGAGCTCCCGAAGGTTCCGGGATTTCCAGTTTTTCAACGGAATGGTGAAATGAATGTGCAAATTCGGAAAGTAAAGAATCAACAATTCCGTCCCAATTTTGAACTTTTACAACTTATTTACCTCTTTCATCAACACATCTTTGAAGATATTTTGCGTGTTGCTCGTGGTGGTGTTGTTTTTGCACCTGGTCATTCTCCAATACCACTTTTAATTGTTCCTTTGAAAAAATTAAGCACTGCCGATTTGGATTATGAAATTGACAAAGATTACCTAAATTGGGATATTCGTGAATCTCCAACTACACCTTCAGATGAAATTAGAAAACAATTTTCTTTTGAAGAATCAAACTACTTAAATGCTGTTGTTTCACCTTGGTATCGTTCTGAAGATCAGTCTGCTTTTTATTACGTAGCTGAGATAATGACTGATCAATTTCCGTCTTCTTCATTTCCTGATGAAAAATTTACTTGTTTTAATCAATATTTTATGAGTAAATATCAATTGGAGATCTACAATCAAAAACAAAATTTACTTGATGTTGACCATACATCTGCTAGAATGAATCTTTTATTACCACGAGCAATTACTGGAAAATCAGCTACTCGTTCATTAGACCCATCTCAACGTCAAATTCTTGTTCCAGAATTGGTACATATACATCCTCTTTCTGCAACTCTTTGGTCAATTATTGTTACTCTTCCTACAATTCTTTATAGACTTAATTCTTTACTGCTTGCTGATGAATTTCGTAGCAAAGTTCTTGAAGATGCTTTAAAAATTAATTCACAAACACCCGACGATTTTGAATGGAGTCCTCTTCAATATGTCACTCTAAATGATGATTTGACACAAAAAAGTATAAGAAATTTAGATCAGTTGAGAAAAATGAACAAACAAGAGAAGGAAAATGAAGTACCAATGGAGTGTGACATTATTGAGGAGAATGTGAATGAGACAGCTGCAAGTGGAATTGTGGGTAAATTAAAATATTTGAAATCAAATGATTTTGAAATCGGCGTTTGGGACCCTTCAGATGGCGCACAAATGTCTAACAATGATAATACTCTGAATGAACCTTCCCCAGTCATTCATAATGCACCTGTAAATGGAATAATCCCTGGGCGGCGGAATGGTCTTCGAGGAGTTATAGCTGCTAGAGACGAGGAACTCTCTGAAATTATTGCTGTTGGTAATGATACAACACTTCATAATTATGGAGATATTTCTGATGATGATGATGTTGCAGCTGAATATGATAAATTTAAATTTTTGATGGACAATAAAATGACTACAAGTGATATTGGTGATCTCGGTGAAATGGATGTTCGACCTGCTGGGTGGAATGACGACAGTAATGCCAATGTTATCCAAATTGAAAACGAAAGTCTTCCCTTGACAATATCGACAAACAATCCTCATATTAATATTGCAAGTTTAATGAATGATTTGGAGAAAAATTATGCTGCTTTTGGTACTTCAAGCCATATTTCGAATAATACTAGAACGAATACACCGATCATTTCGAGTGATACTGCTGTTGACAAAACGGCCTTGCAAAGAAAGGAGGTCCTCTTAACATATCTTACTAACAATTCGGTAAAAAGGCAAAATGAAGATATTTTACCTGAGGAAGTTTTTTGGGGAATGGATGAAGCTGAGATAGGAGAAAATATTAGCAGTGAAGCATTGACAACTTCGAGTGCTGCGGATGGAATAAATTTGGAAAGATTAGAAACAATTGGAGATTCCTTTCTTAAAATGGCTGTCACCAACTATTTCTACTATAAACACACTGAACAACATGAAGGAAAGTTAAGCTATGCTCGTTCAAAAGAAGTTTCGAATTCTCATCTTTTTTATCTTGGACGGCAACGAGGCATCCCTCTTCTAATAGAAACACTTAAATTCGATCCACATGTTAACTGGCTTCCTCCTTGTTATGCTTCTACTTCAGAATTCCATGCTGTCAATCCTTTTGATTATACAGATTTAGATGAAGATCAATGTCAAGTGCCAATGGAAGGTGTTGATACTACTGAAACTTTAGATCAACAGCAAAAGACTAATAAAGAAACAATAGCAACTGGATGGGGTACTCTTGACGATGATAAACAAAATTATAAACGTGAAAATGGAGTAGAAACTTTAACTTTTCCACAACCAACAAAATCAGAAATTCCAGACTTACCTCCAATGCCATACAATATGCTTACTCAACAATGGATAAGTGACAAATCAATTGCTGATGCAGTAGAGGCATTAATTGGAGCACATTTAATTCAACTTGGACAATCAGCTACTCTCAAATTTATGAATTGGTTGGGAATTAAGGTTCTAACTGACATTTCTTCATTGCCCTCACCACTTCTACGTTTCATTGACACACCTGAAGATCCTAATCTTTCACTTAAACATCTTGCCCTTCTTTATGAGAAATTTGATTTTGCTACTGTTGAGAACAATATTGGCTATAAATTTGCAAATAAGGCATACTTAGTCCAAGCATTTACTCACGCTTCATATTATAATAATCGAGTAACCGGTTGTTACCAGCGTTTGGAATTCCTTGGCGATGCTGTTTTAGATTATATGATAACTCGTTTTCTTTATGAACACAAGAAGCAATATAGTCCGGGTGTTCTTACTGATCTTCGCTCCGCTCTTGTCAATAATACAATTTTTGCTTCGCTTGCAGTTAAATATTCTTTTCACAAACATTTTGTTATGATTTGTCCTCCACTTTATCAAATGGTCGAAAAGTTTGTTAATTTCTGTAAGCAAAAAGATTTTCTTCATTGCGCGAATTTTGACGATGAAATATTTATGTTAACTGAAGATGAAATCGATGAGGAAGACCTTTTAAGTGAAGAAGATGTCGAAGTACCTAAAGCAATGGGTGATATTTTTGAATCAATTGCTGGCGCTGTTTATTTAGATTGTGGAATGGATTTAGATATTGTTTGGCGTGTATTTTATAATTTAATGAGAGATGTTATACAAAAATGTTGTGAAAATCCACCACAATCGCCAGTTAGAGAATTATTTGAAAGGAAGAATTGTCGAGCAAAATTCTCAAAATTGGAACGAAAATTAGAAACAGGCAAAGTTAGAGTTACTGTAACGGTTAATGATAATCTCCAATTTACTGGTATGGGACGTAGTTATCGAATTGCTAAATGTACAGCTGCTAAACGAGCACTTCAACATTTAAGAAAATTAGATGCCACAAAAACAGCAAGGAATAAATAA

>RCFL01003232-*M. incognita* (35 exons)

ATGTCTCCACCGAAGGATTTTAGTGGTAAATGTATTTCTCCAAGGGATTATCAGGTTGAATTGCTTGATCGTGCAAAAATTCAAAACACGATTATTTCGCTCGGTACTGGTAGTGGAAAAACGTTTGTGGCGGTTCTTTTAATAAAGGAATATTCTCAGAGATTGTTACATCAAAATGAAAAAGCTGTGTTTTTGGTTAATACTGTTGAACTTGTTGCCCAGCAAGCCGAGCACATTGAATTTCATTCAAGTTTGTCTGTCGCTCGTATTTCTGGCTCTACAATTAAAAGAAAATATGAGAGAAAAGAAGTTGAGAAAATTACTAGCAGCAATCAGGCAAGTCATGTATTATTAAAGTCTAGTTTATTTTTTGTTTTAAAGGTGATTGTAATTACAGCTCAAGTGTTTCTCGATTTAATTAATCACGGGTTATTCGATTTCTCTTCTTTGGCCTTGTTGATTGTTGATGAATGCCATCATTGTTTGGGAGAACTGCATCCATATAGGCTTATTATGAATCATTACAAGAAACTGCAAGGTCAACGACCACGCGTTTTGGGCCTTACAGCTTCAATTCTGAATAAAAAAGTGCCATCCTCTCGTATTGAGTGCACCGCTCAGCTGCTAGAACAAATAATGGATTCGCAGATAGAGACCTCCAGCAATTATACGCAGGAGCGCCTTCGTTCATTTATTGAAAAGAATGAAGATTTTCATTCAGAACTTGAAGTTGATCCGCGTCGCAAAATTTTTGAGACAATTTCCCGTTCATTTTCAACTCTTCAACAAGTTGGTAGCTGGGCAGCTCTAAAAGGTTTTATCCTTTGGCAAAAGAATTTACTTAAACATGTTGATGACCCAATAATTGGCAATAAACAAAAATGTATTTTAAGAATGGCCGAGACTGCTTTTAGAACTTGTTCAAAAGTTTTAAGTCACAAAATAAATCCGTTAAACAGCTATGACAAATTGGTTAACGTAAATAAATCATTTATCAGTGATAGAGTGCGCAAATTGTTGGAAATATTAAAAAGTTATTCACCCTCAAAACGTGAATTGTCTGGAATTAAAGATACACTTTTTGGATTAGTTTTTGTGAAAGAACGATTTATTGCCTTTATGATTAATAATTTGCTTCGGTTTCTTGTAAAACAAAATCCTGAAGAGTTTGGACATTTAAAAGTGGACTTTATTGTTGGACACACTGGAAATTCTGAGACAGGAGATGAAGATAGGCGTTTGGTGAATCGAAAACAAGAACAAACTCTTACAAAATTCCGAAATGGTCAATTAAATTTATTAATTACGACAAATGTTTTGGAAGAAGGAATTGATTTAAGAAATTGTAATTTAGTCGTTCGATTTGATCCGCCGATGGATTTTCGGTCATTTATTCAATCTAGCGGTCGTGCTCGAAGAGAAAATAGCGCTTTTTATATACTTATTGAAGAGAAAAATTATTTGGAGTTTATGATGGACTTAACAGGATATGCACAAGCAGAAGAGTTTGTTTTGCGGCGTTATCGTTCTGGCAATGATTTTACTCTGGAAGGAAATGATGAAACAAAATTATTACATCCACATCTGGATGATGCTGTTGCTCCTTATGTTGTTACAACTGAAAAAGGGACAGCTAAAGTCTCATTAAGTAATGCTATACAATATTGTGCAAAATTACCATCTGATATTTTTACTCGATTAGTGCCTCGTTATACAGTACAAACTTTATCAGAAAATGGACAAACTTTGTATATTGCTGAATTATACCTTCCAATCAATTCACCAATAAAGGAGCCAATAAAATCTAAGCCGATGACTTCTAAAAGATTATCTTTAATGGCAGTCGCTTTGGAGGCTTGCAAACGATTACATCAAAGAAAAGAATTAAATGACAATCTTTTGCCTGCAGGAAAAGATATCTTGGATGATCTTTTAGGGGAAGTTGATGATGATGAATATCTGCCTCATTTACCAAGCAGAATGGGATCCTCAAAGAAAAAACGTCTCTACGATAGAAAAATGTCGAAAACTCTTAATTCTACATTACCTTCGCAAGACAGTGAATGCGTTCTTTACGTTATGGAAATGAAACTGATTAAGCCCGTTACTGATGAAGGGAATCCGAAAAGGAGAAAAATTATTGATCCGTTCGAGTCAAATTCGGCGTTTGGCTTTTTGTCTTCGAAGGAGCTCCCGAAGGTTCCGGGATTTCCAGTTTTTCAACGGAATGGTGAAATGAATGTGCAAATTCGTAAAGTAAAGAATCAGCAATTTCGTCCCACTTTTGAACTTTTACAACTTATTTACCTCTTTCATCAACACATCTTTGAAGATATTTTGCGTGTTGCCCGTGGTGGTGTTGTTTTTGCACCTGGTCATTCTCCAATACCACTTTTAATTGTTCCCTTGAAAAAATTAAGCACTGCCGATTTGGATTATGAAATTGACAGAGATTACCTAAATTGGAATATCCGAGAATCTCCAACTACACCTTCAGATGAAATTAGAAAACAATTTTCATTTGAAGAATCAAACTACTTAAATGCTGTTGTTTCACCTTGGTATCGTTCTGAAGATCAGTCTGCTTTTTATTACGTAGCCGAGATAATGACTGATCAATTTCCGTCTTCTTCATTTCCTGATGAAAAATTTGCTTGTTTTAATCAATACTTTATGAGTAAATATCAATTGGAGATCTACAATCAAAAACAAAATTTACTTGATGTTGACCATACATCTGCTAGAATGAATCTTTTATTACCACGAGCAATTTCTGGAAAATCAGCTACTCGATCATTGGACCCATCTCAACGTCAAATTCTTGTCCCAGAATTGGTACATATACATCCTCTTTCTGCAACTCTTTGGTCAATTATTGTTACTCTTCCTACAATTCTTTATAGACTTAATTCTTTACTGCTTGCTGATGAATTTCGTAGCAAAGTTCTTGAAGACGCTTTGAAAATTAATTCACAAACACCCGACGATTTTGAATGGAGTCCTCTTCAATATGTCACTCTAAATGATGATCTGACACAAAAAAGTATAAGAAATTTAGATCAGTTGAGAAAAATGAACAAACAAGAGAAGGAAAATGAAGTACCAATGGAGTGTGACATTATTGAAGAGAATGTGAATGAGACAGCAGCAAGTGGAATAGTGGGTAGATTAAAATTTTTGAAATCAAATGATTTTGAAATCGGCGTTTGGGACCCTTCAGATGGCGCACAAATGTCTAACAATGATAATACTCCGAATGAACCTTCTCCAGTCATCCATAATGCACCTGTAAATGGATTAATCCCTGGGCGGAGGAATGGTCTTCGAGGAGTTATAGCTGCTAGAGACGAGGAACTCTCAGAAATTATTGCTGTTGGTAATGATACAACGCTTCATAATTATGGAGATATTTCTGATGATGATGATGTTGCAGCTGAATACGATAAATTTAAATTTTTGATGGACAATAAAATGACTACAAGTGATATTGGTGATCTAGGTGAAATGGATGTTCGACCTGCTGGTTGGAATGACGATAGCAATGCCAATGTTATCCAAATTGAAAATGAAAGTCTTCCCTTGACAATATCGACAAACAATCCTCATATTAATATTGCAAGTTTAATGAATGATTTGGAGAAAAATTATGCTGCTTTTGGTACTTCAAACCATATTTCCAATAATAATAAAACGAATACACCGAACATTCCGAATGCTACTGCTGACAAAACGGACTTGCAAAAAAAGGAGGTCCTCTTAACATATCTTATTAACAATTCGGTAAAAAGGCAAAATGAAGATATTTTACCTGAGGAAGTTTTTTGGGGAATGGATGAAGCTGAGATAGGAGAGAATATTAGCAGTGAAGCATTGACAACTTCAAGTGCTGCGGATGGAATAAATTTGGAAAGGTTGGAAACAATTGGAGATTCTTTCCTCAAGATGGCTGTCACCAACTATTTCTACTATAAACACACGGAACAACATGAAGGAAAGTTAAGCTATGCACGTTCAAAAGAAGTTTCGAATTCCCATCTTTTTTATCTTGGACGGCAACGAGGAATCCCTCTTTTAATAGAAACACTGAAATTCGATCCACATGTTAACTGGCTTCCTCCTTGTTATGCTTCTACTTCAGAATTCCATGCTGTCAATCCTTTTGATTATACAGATTTAGATGAAGATCAATGTGCAGTGCCAATGGAGGGTGTTGATACTACAGAAACTGTAGATCAACAGCAAAAGATTAATAAAGAAACAATAGCAACTGGATGGGGAACTATTGACGATGATAAACAAAATTATAAACGTGAAAATGGAGTAGAAACTTTAACTTTTCCACAACCAACAAAATCAGAAATTCCTGACTTACCTCCAATGCCATACAATATGCTTACTCAACAATGGATAAGTGACAAATCGATTGCTGATGCAGTAGAGGCATTAATTGGAGCACATTTAATTCAACTTGGACAATCAGCTACTCTGAAATTTATGAATTGGTTGGGAATTAAGGTACTGACTGATATTTCTTCATTGCCCTCACCACTTCTTCGTTTCATTGACACACCTGAAGATCCTAATCTTTCACTTAAACATCTTGCCCTTCTTTATGAGAAATTTGATTTTGCCACTGTTGAGAACAATATTGGTTATAAATTTGCAAATAAGGCATATTTAGTCCAAGCATTTACTCACGCTTCATATTATAACAATCGAGTAACCGGTTGTTACCAACGTTTGGAATTCCTTGGCGATGCTGTTTTAGATTATATGATAACTCGTTTTCTTTATGAACATAAGAAGCAATATAGTCCTGGTGTTCTTACTGATCTTCGCTCCGCTCTTGTCAATAATACAATTTTTGCTTCGCTTGCAGTTAAATATTCTTTTCACAAACATTTTGTTATGATTTGTCCTCCACTTTATCAAATGGTCGAAAAGTTTGTTAATTTCTGTAAGCAAAAAGATTTTCTTCATTGCGCGAATTTTGACGATGAAATATTTATGTTAACTGAAGATGAAATCGATGAGGAAGACCTTTTAAGTGAAGAAGATGTAGAAGTACCTAAAGCGATGGGTGATATTTTCGAATCAATTGCTGGTGCTGTTTATTTAGATTGTGGAATGGATTTAGATATTGTTTGGCGTGTATTTTATAATTTAATGAGAGATGTTATACAAAAATGTTGTGAAAATCCACCACAATCGCCAGTTAGAGAATTGTTTGAAAGGAAGAATTGTCGAGCAAAATTTTCAAAATTGGAACGAAAATTAGAAACAGGCAAAGTTAGAGTTACTGTAACGGTTAATGACAATCTCCAATTTACTGGTATGGGAAGGAGTTATCGAATTGCTAAATGTACAGCTGCTAAACGAGCACTACAACATTTAAGAAAATTAGATGCCACAAAAACAGCAAAGAATAAATAA

>RCFL01003234-*M. incognita* (35 exons)

ATGTCTCCACCGAGGGATTTTAGTGGTAAATGTATTCCTCCAAGAGATTATCAGTTTAAATTCAAGGTTGAATTACTTGATCGTGCAAAAATTCAAAACACGATTATTTCGCTCGGTACTGGTAGTGGAAAAACGTTTGTGGCGGTTCTTTTAATAAAGGAATATTCCCAGAGGTTGTTACATCAAAATGAAAAAGCTGTGTTTTTGGTTAATACTGTTGAACTTGTTGCCCAGCAAGCCGAGCATATTGAATTTCATTCAAGTTTGTCTGTCGCCCGTATTTCTGGCTCTACAATTAAAAGAAAATATGAGAGAAAAGAAGTTGAGAAGATTACTAACACCAATCAGGCTAGTCATTTATTTGATTTCTCTTCTTTGGCCTTGTTGATTGTTGATGAATGCCATCATTGTTTGGGAGAACTGCATCCATATAGGCTGATTATGAATCATTACAAGAAACTTCAAGGTCAGCGACCACGCGTTTTGGGCCTTACAGCTTCAATTCTGAATAAAAAAGTGCCATCCTCTCGTATTGAGTGCACCGCTCAGCTGCTAGAACAAATAATGGATTCACAGATAGAAACTTCCAGCAATTATACACAGGAGCGCCTTCGTTCATTCATTGAAAAGAATGAAGATTTTCATTCAGAACTTGAAGTTGATCCGCGTCGCAAAATTTTTGAGACAATTTCCCGTTCATTTTCAACTCTTCAACAAGTTGGTAGCTGGGCAGCTCTAAAAGGTTTTATCCTTTGGCAAAAGAATTTACTTAAACATGTTGATGACCCAATAATTGGCAATAAACAAAAATGTATTTTAAGAATGGCTGAAACTGCTTTTAGAACTTGTTCAAAAGTTTTAAGTCACAAAATAAATCCGTTAAACAGCTATGACAAATTGAGTAACGTAAATAAATCATTTATCAGTGACAGAGTGCGCAAATTGTTGGAAATATTAAAAAGTTATTCGCCCTCGAAACGTGAATTGTCTGGAATTAAAGATACACTTTTTGGATTAGTTTTTGTGAAAGAACGATTTATTGCCTTTATGATTAATAATTTGCTTCGGTTTCTTGTAAAACAAAATCCTGAAGAGTTTGGACATTTAAAAGTGGACTTTATTGTTGGACATACTGGAAATTCTGAGACAGGCGATGAAGATAGGCGTTTGGTGAATCGAAAACAAGAACAAACTCTTACAAAATTCCGAAATGGCCAATTAAATTTATTAATTACGACAAATGTTTTGGAAGAAGGAATTGATTTAAGAAATTGTAATTTAGTCGTTCGATTTGATCCGCCGATGGACTTTCGTTCATTTATTCAATCTAGCGGTCGTGCTCGAAGAGAAAATAGCGCTTTTTATATACTTATTGAAGAGAAAAATTATTTGGATTTTATGATGGACTTAACAGGGTATGCACAAGCAGAAGAGTTTGTTCTGAGACGTTATCGTTCTGGCAATGATTTTACTTTGGAAGGAAATGATGAAACAAAAATATTACATCCACATCTGGATGATGCTGTTGCTCCTTATGTTGTTACAACTGAAAAAGGGACAGCTAAAGTCTCGTTAAGTAATGCTATACAGTACTGTGCAAAATTACCATCTGATATTTTTACTCGATTAGTGCCACGTTATACAATACAAACTTTATCAGAAAATGGACAAACTTTGTATATTGCCGAATTATATCTCCCAATCAATTCACCAATAAAGGAGCCAATAAAATCTAAACCGATGAATTCTAAAAGATTATCTTTAATGGCAGTCGCATTGGAGGCTTGCAAACGATTACATCAAAGAAAAGAATTAAATGACAATCTTTTGCCTGCAGGAAAGGATATCTTGGATGATCTTTTAGGTGAAGTTGATGATGATGAATATCTGCCTCATCTACCAAGCAGAATGGGATCCTCAAAGAAAAAACGTCTCTATGATAGAAAAATGTCGAAAACTCTTAATTCTACATTACCTTCGCAAGACAGTGAATGCATTCTTTACGTTATGGAAATGAAACTGGTTAAGCCCGTTACTGATGAAGGGAATCCGAAAAGGAGAAAAATTATTGATCCGTTCGAATCAAATTCGGCATTTGGATTTTTGTCTTCGAAGGAGCTCCCGAAGATTCCGGGATTTCCAGTTTTTCAACGGAATGGTGAAATGAATGTGCAAATTCGAAAAGTAAAGAATCAACAATTTCGTCCCACTTTTGAACTTTTACAACTTATTTACCTCTTTCATCAACACATCTTTGAAGATATTTTGCGTGTTGCTCGTGGTGGTGTTGTTTTTGCACCTGGTCATTCTCCAATACCACTTTTAATTGTTCCTTTGAAAAAATTAAGCAATGCCGATTTGGACTATGAAATTGACAAAGATTACCTAAATTGGGATATTCGTGAATCTCCAACTACACCTTCAGATGAAATTAGAAAACAATTCTCTTTTGAAGAATCAAACTACTTAAATGCTGTTGTTTCACCTTGGTATCGTTCTGAAGATCAGTCTGCTTTTTATTACGTAGCTGAGATAATGACTGATCAATTTCCGTCTTCTTCATTTCCTGATGAAAAATTTACTTGTTTTAATCAATATTTTATGAGTAAATATCAATTGGAGATCTACAATCAAAAACAAAATTTACTTGATGTTGACCATACATCTGCTAGAATGAATCTTTTATTACCACGAGCAATTACTGGAAAATCAGCTACTCGTTCATTAGACCCATCTCAACGTCAAATTCTTGTTCCAGAATTAGTACATATACATCCTCTTTCTGCAACTCTTTGGTCAATTATTGTTACTCTTCCTACAATTCTTTATAGACTTAATTCTTTACTGCTTGCTGATGAATTTCGTAGCAAAGTTCTTGAAGATGCTTTAAAAATTAATTCACAAACACCCGACGATTTTGAATGGAGTCCTCTTCAATATGTCACTCTAAATGATGATCTGACACAAAAAAGTATAAGAAATTTAGATCAGTTGAGAAAAATGAACAAACAAGAGAAGGAAAATGAAGTACCAATGGAGTGTGAGAATATTGAGGAGAATGTGAATGAGACAGCTGCAAGTGGAATAGTGGGTGAATTAAAAAATTTTAGATCAAATGATTTTGAAATCGGCGTTTGGGACCCTTCAGATGGCGCACAAATGTCTAACAATGATAATACTCTGAATGAACCTTCCCCAGTCATTCATAATGCACCTGTAAATGGATTAATCCCTGGTCGGAGGAACGGTCTTCGAGGAGTTATAGCTGCTAGAGACGAGGAACTCTCTGAAATTATTGCTGTCGGGAATGATACAACACTTCATAATTATGGAGATATTTCTGATGATGATGATGTTGCAGCTGAATATGATAAATTTAAATTTTTGATGGACAATAAAATGACTACAAGTGATATTGGTGATCTCGGTGAAATGGATGTTCGACCTGCTGGGTGGAATGACGACAGTAATGCCAATGTTATCCAAATTGAAAACGAAAGTCTTCCCTTGACAATATCGACAAACAATCCTCATATTAATATTGCAAGTTTAATGAATGATTTGGAGAAAAATTATGCTGCTTTTGGTACTTCAAGCCATATTTCGAATAATACTAGAACGAATACACCGATCATTTCGAGTGATACTGCTGTTGACAAAACGGCCTTGCAAAGAAAGGAGGTCCTCTTAACATATCTTACTAACAATTCGGTAAAAAGGCAAAATGAAGATATTTTACCTGAGGAAGTTTTTTGGGGAATGGATGAAGCTGAGATAGGAGAAAATATTAGCAGTGAAGCATTGACAACTTCGAGTGCTGCGGATGGAATAAATTTGGAAAGATTAGAAACAATTGGAGATTCCTTTCTTAAAATGGCTGTCACCAACTATTTCTACTATAAACACACTGAACAACATGAAGGAAAGTTAAGCTATGCTCGTTCAAAAGAAGTTTCGAATTCTCATCTTTTTTATCTTGGACGGCAACGAGGCATCCCTCTTCTAATAGAAACACTTAAATTCGATCCACATGTTAACTGGCTTCCTCCTTGTTATGCTTCTACTTCAGAATTCCATGCTGTCAATCCTTTTGATTATACAGATTTAGATGAAGATCAATGTCAAGTGCCAATGGAGGGTGTCGATACTACTGAAACTTTAGATCAACAGCAAAAGACTAATAAAGAAACAATAGCAACTGGATGGGGTACTCTTGACGATGATAAACAAAATTATAAACGTGAAAATGGAGTAGAAACTTTAACTTTTCCACAACCAACAAAATCAGAAATTCCAGACTTACCTCCAATGCCATACAATATGCTTACTCAACAATGGATAAGTGACAAATCAATTGCTGATGCAGTAGAGGCATTAATTGGAGCACATTTAATTCAACTTGGACAATCAGCTACTCTCAAATTTATGAATTGGTTGGGAATTAAGGTTCTAACTGACATTTCTTCATTGCCCTCACCACTTCTACGTTTCATTGACACACCTGAAGATCCTAATCTTTCACTTAAACATCTTGCCCTTCTTTATGAGAAATTTGATTTTGCTACTGTTGAGAACAATATTGGCTATAAATTTGCAAATAAGGCATACTTAGTCCAAGCATTTACTCACGCTTCATATTATAATAATCGAGTAACCGGTTGTTACCAGCGTTTGGAATTCCTTGGCGATGCTGTTTTAGATTATATGATAACTCGTTTTCTTTATGAACACAAGAAGCAATATAGTCCGGGTGTTCTTACTGATCTTCGCTCCGCTCTTGTCAATAATACAATTTTTGCTTCGCTTGCAGTTAAATATTCTTTTCACAAACATTTTGTTATGATTTGTCCTCCACTTTATCAAATGGTCGAAAAGTTTGTTAATTTCTGTAAGCAAAAAGATTTTCTTCATTGCGCGAATTTTGACGATGAAATATTTATGTTAACTGAAGATGAAATCGATGAGGAAGACCTTTTAAGTGAAGAAGATGTCGAAGTACCTAAAGCAATGGGTGATATTTTTGAATCAATTGCTGGCGCTGTTTATTTAGATTGTGGAATGGATTTAGATATTGTTTGGCGTGTATTTTATAATTTAATGAGAGATGTTATACAAAAATGTTGTGAAAATCCACCACAATCGCCAGTTAGAGAATTATTTGAAAGGAAGAATTGTCGAGCAAAATTCTCAAAATTGGAACGAAAATTAGAAACAGGCAAAGTTAGAGTTACTGTAACGGTTAATGATAATCTCCAATTTACTGGTATGGGAAGGAGTTATCGAATTGCTAAATGTACAGCTGCTAAACGAGCACTTCAACATTTAAGAAAATTGGATGCCACGAAAACAGCAAAGAATAAATAA

>RCFM01006500-*M. enterolobii* (36 exons)

ATGTCTCCACCGAAGGATTTTAGTGCTAAATGTATTCCTCCAAGAGATTATCAGGTTGAATTACTTGATCGTGCAAAAATTCAAAACACGATTATTTCGCTCGGTACTGGTAGTGGAAAAACGTTTGTGGCGGTTCTTTTAATAAAAGAATATTCTCAGAGGTTGTTACATCAAAATGAAAAAGCTGTGTTTTTGGTTAATACCGTTGAACTTGTTGCCCAGCAAGCTGAGCACATTGAATTTCATTCGAGTCTTTCTGTTGCCCGTATTTCTGGTTCTACAATTAAAAGAAAATATGAGAGAAAAGAAGTTGAGAAAATTACTAGCAGCAGTCAGGTGATTGTAATTACTGCTCAAGTGTTTCTTGATTTAATCAATCATGGGTTATTCGATTTCTCTTCTTTGGCCTTGTTGATTGTTGATGAATGCCATCATTGTTTGGGAGAACTGCATCCATATAGGCTGATTATGAATCATTACAAGAAATTGCAAGGTCAACGACCACGCGTTTTGGGCCTTACAGCTTCAATTCTGAATAAAAAAGTGCCATCCTCTCGCATTGAGTGCACCGCTCAACTGTTAGAACAAATAATGGATTCACAGATAGAGACCTCCAGTAATTATACACAGGAGCGCCTTCGTTCATTTATTGAAAAGAATGAAGATTTTCATTCAGAACTTGAAGTTGATCCGCGTCGCAAAATTTATGAAACAATTTCCCGTTCATTTTCAACTCTTCAACAAGTTGGTAGTTGGGCAGCTCTAAAAGGTTTTATCCTTTGGCAAAAGAATTTACTTAAACATGTTGATGACCCAATAATTGGCAATAAACAAAAATGTATTTTAAGAATGGCTGAGACTGCTTTTAGAACTTGTTCAAAAGTTTTAAGTCACAAAATAAATCCATTAAACAGCTATGACAAATTGGGTAACGTGAACAAATCATATATCAGTGACAGAGTGCGCAAATTGTTGGAAATATTAAAAAGTTATTCGCCCTCAAAACGTGAATTGTCTGGAATTAAAGATACACTTTTTGGATTAGTTTTTGTGAAAGAACGATTTATTGCCTTTATGATTAATAATTTACTTCGTTTTCTTGTAAAACAAAATCCTGAAGAGTTTGGACATTTAAAAGTGGACTTTATTGTTGGACACACTGGCAATTCTGAAACTGGCGATGAAGATAGGCGTCTGGTAAATCGAAAACAGGAACGAACTCTTACACAATTCCGGAATGGGCAACTAAATTTATTAATAACAACGAATGTTCTGGAAGAAGGAATTGATTTAAGAAATTGTAATTTGGTTATTCGATTTGATCCACCAATGGATTTTCGGTCATTTATCCAATCTAGCGGTCGTGCTCGAAGGGAAAATAGCGCTTTTTATATACTTATTGAGGAGAAAAATTATTTGGATTTTATGATGGACTTAACTGGATATGCACAAGCAGAAGAGTTTGTTTTGCGACGTTATCGTTCTGGCAATGATTTTACTTTGGAAGGAAATGATGAAACAAAAATGATACATCCCCATCTGGATGATGCTGTTGCTCCTTATGTTGTTACAACTGAAAAAGGGACAGCTAAAGTCTCATTAAGTAATGCTATACAATTGGTGAACAGATATTGTGCAAAATTACCATCAGATATTTTTACTCGATTAGTGCCACGTTATACAATACAAACTTTATCAGAAAATGGACAAACTTTGTATATTGCCGAATTATATCTTCCGATCAATTCACCAATAAAGGAGCCAATAAAATCTAAACCGATGACTTCTAAAAGATTAGCTTTAATGGCAGTTGCTTTGGAGGCTTGCAAACGATTACATCAAAGAAAAGAATTAAATGACAATCTTTTGCCTGCAGGAAAAGATATCTTGGATGATCTTCTAGGTGAAGTAGATGATGACGAATATCTGCCTCATTTACCAAGCAGAATGGGATCCTCAAAGAAAAAACGTCTCTACGATAGAAAAATGTCGAAAACTCTTAATTCTACATTACCTTCGCAAAACAGTGAATGCATTCTTTACGTTATGGAAATGAAACTAGTTAAGCCCGTTACTGATGAAGGAAATCCAAAGAGGAGAAAAATTATTGATCCTTTCGAATCAAATTCGGCATTTGGATTTTTGTCTTCTAAGGAGCTCCCGAAGGTTCCAGGATTTCCAGTTTTTCAACGGAATGGCGAAATGCTTGTGCAAATTCGAAAAGTAAAGAATCAACAATTTCGTCCCACTTTTGAACTTTTACAACTTATTTACCTCTTTCATCAACACATTTTTGAAGATATTTTGCGTGTTGCTCGTGGTGGTGTTGTTTTTGCACCTGGTCATTCTCCAATACCACTTTTAATTGTTCCTTTGAAAAAATTAAGCACTGCCGATTTGGATTATGAAATTGACAAAGATTACCTTAATTGGAATATCCGCGAATCTCCAACTACACCTTCAGATGAAATTAGGAAACAATTTTCTTTTGAAGAATCAAACTACTTAAATTCTGTTGTTTCACCTTGGTATCGTTCTGAAGATCAGTCTGCTTTTTATTACGTAGCTGAGATAATGACTGATCAATTTCCGTCTTCTTCATTTCCTGATGAAAAATTTACTTGTTTCAATCAATACTTTATGAGTAAATATCAATTGGAGATCTACAATCAAAAACAAAATTTACTTGATGTTGACCATACATCTGCTAGAATGAATCTTTTATTACCACGAGCAATTACTGGAAAATCGGCTACTCGCTCACTAGACCCATCTCAACGTCAAATTCTTGTTCCAGAATTGGTACATATACATCCTCTTTCTGCAACTCTTTGGTCAATTATTGTTACTCTTCCTACAATTCTTTATAGACTTAATTCTTTACTGCTTGCCGATGAATTTCGTAGCAAGGTTCTTGAAGATGCTTTGAAATTTAATTCACAAACACCCGACGATTTTGAATGGAGTCCTCTTCAATATGTCACTCTAAATGATGATCTGACACAAAAAAGTATAAAAAATTTAGATCAATTGAGAAAAATGAATAAACAAGAGAAGGAAAATGAAGTACCAATGGAGCTTGATATTATTGGAGAGAATGTGAATGAGACAGCAGCAAGTGGAATTGTGGGTGAATTAAAAATTTTGAAATCAAATGATTTTGAAATCGGTGTTTGGGACCCTTCAGATGGCGCACAAATGTCTAACAATGATAATACTTCGAATGAGCCTTCTCCAGTCATTCACAATGCACCTGTAAATGGATTAATCCCTGGGCGGAGGAATGGTCTTCGAGGAGTTATAGCTGCTAGAGACGAGGAGCTCTCAGAAATTATTGCTGTCGGGAATGATACAACACTTCATAATTATGGAGATATTTCTGATGATGATGATGTTGCAGCTGAATATGATAAATTTAAATTTTTGATGGACAATAAAATGACTACAAGTGATATTGGTGATCTAGGTGAAATGGATGTTCGACCTGCGGGGTGGAATGACGATAGCAATGCCAATGTTATCCAAATTGAAAACGAAAGCCTTCCATTGACAATATCGACAAACAATCCTCATATTAATATTGCAAGTTTAATGAATGATTTGGAGAAAAATTATGCTGCTTTTGGTACTTCAAGCCATATTTCCAATAATAATAAGGCGAATACCCCTATCATTCCGAGTGCTACTGCTGTTGACAAAACGGTCTTGCAAAGAAAGGAGGTCCTCTTAACATATCTTATTAACAATTCGGTAAAAAGGCAAAATGAAGATATTTTACCAGAGGAAGTTTTTTGGGGAATGGATGAAGCTCAGATAGGAGAGAATATTAGCAGTGAAGCATTGACAACTTCAAGTGCTGCGGATGGAATAAATTTGGAAAGGTTAGAAACAATTGGAGATTCATTTCTCAAAATGGCTGTTACCAACTATTTCTACTATAAACACACTGAACAACATGAAGGAAAATTAAGCTATGCTCGTTCAAAAGAAGTTTCGAATTCCCATCTTTTTTATCTTGGACGGCAACGAGGCATCCCTCTTCTAATAGAAACACTAAAATTCGATCCACATGTTAACTGGCTTCCTCCTTGTTATGCTTCTACTTCAGAATTTCATGCTGTCAATCCTTTTGATTATACAGATTTAGATGAAGATCAACGTGAAGTGCCAATGGAGGGTGTTGATACTAATGAAACTGTAGATCAACAGCAAAAGATTAATAAAGAAGCAATAACAACTGGGTGGGGTACTCTGGACGATGATAAACAGAATTATAAACGTGAAAATGGAGTAGAAACTTTAACTTTTCCTCAACCAACAAAATCAGAAATTCCAGACTTACCTCCAATGCCATATAATATGCTAACTCAACAATGGATAAGTGACAAATCAATTGCTGATGCGGTAGAGGCATTAATTGGAGCACATTTAATTCAACTTGGACAATCAGCTACTCTCAAATTTATGAATTGGTTGGGAATTAAGGTTCTAACTGATATTTCTTCATTGCCCTCACCACTTCTACGTTTTATTGACACACCTGAAGATCCTAATCTTTCACTTAAACACCTTGCCCTTCTTTATGAGAAATTTGATTTTGCTACTGTTGAGAACAACATTGGTTATAAATTTGCAAATAAGGCATATTTAGTCCAAGCATTTACACACGCTTCATATTACAACAATCGAGTAACCGGTTGTTACCAACGTTTGGAATTCCTTGGCGATGCTGTTTTAGATTATATGATAACTCGTTTTCTTTACGAACACAAAAAGCAATATAGTCCTGGTGTTCTTACTGATCTTCGCTCCGCTCTTGTCAATAACACAATTTTTGCTTCTCTTGCAGTTAAATATTCTTTTCACAAACATTTTGTTATGATTTGTCCTCCACTCTATCAAATGGTCGAAAAGTTTGTTAATTTTTGTAAGCAAAAAGATTTTCTTCACTGCGCGAATTTTGACGATGAAATATTTATGTTAACTGAAGATGAAATTGATGAGGAAGATCTTTTAAGTGAAGAGGATGTAGAAGTACCTAAAGCGATGGGTGATATTTTCGAATCAATTGCTGGTGCTGTTTATTTGGATTGTGGAATGGATTTAGATATTGTTTGGCGTGTATTTTATAATTTAATGAGAGATGTTATACAAAAATGTTGTGAAAATCCACCACAATCGCCAGTTAGGGAATTATTTGAGAGAAAGAATTGTCGAGCAAAATTCTCAAAATTGGAACGAAAATTAGAAACAGGCAAAGTTAGAGTTACTGTAACGGTTAATGATAATCTCCAATTTACTGGGATGGGGAGGAGTTATCGAATTGCTAAATGTACAGCTGCTAAACGAGCACTTCAACATTTAAGAAAGTTAGATGCCACAAAATCAGCAAAGAATAAATAA

>RCFN01006666-*M. floridensis* (36 exons)

ATGTCTCCACCGAAGGATTTTAGTGGTAAATGTATTCCTCCAAGAGATTATCAGTTTAAATTCAAGGTTGAATTACTTGATCGTGCAAAAATTCAAAACACGATTATTTCGCTCGGTACTGGGAGTGGAAAAACATTTGTGGCGGTTCTTTTAATAAAAGAATATTCTCAGAGGTTGTTACATCAAAATGAAAAAGCTGTGTTTTTGGTTAATACTGTTGAACTTGTTGCCCAGCAAGCCGAGCATATTGAATTTCATTCAAGTTTGTCTGTCGCCCGTATTTCTGGCTCTACAATTAAAAGAAAATATGAGAGAAAAGAAGTTGAGAAGATTACTAACACCAATCAGGCTAGTCATGTGATTGTAATTACAGCTCAAGTGTTTCTTGATTTAATCAATCACGGCTTATTCGATTTCTCTTCTTTGGCCTTGTTGATTGTTGATGAATGCCATCATTGCTTGGGAGAACTGCATCCATATAGGCTGATTATGAATCATTACAAGAAACTGCAAGGTCAGCGACCACGCGTTTTGGGCCTTACAGCTTCAATTCTGAATAAAAAAGTGCCATCCTCTCGTATTGAGTGCACCGCTCAGCTGCTAGAACAAATAATGGATTCACAGATAGAAACTTCCAGCAATTATACACAGGAGCGCCTTCGTTCATTCATTGAAAAGAATGAAGATTTTCATTCAGAACTTGAAGTTGATCCGCGTCGCAAAATTTTTGAGACAATTTCCCGTTCATTTTCAACTCTTCAACAAGTTGGTAGCTGGGCAGCTCTAAAAGGTTTTATCCTTTGGCAAAAGAATTTACTTAAACATGTTGATGACCCAATAATTGGCAATAAACAAAAATGTATTTTAAGAATGGCTGAAACTGCTTTTAGAACTTGTTCAAAAGTTTTAAGTCACAAAATAAATCCGTTAAACAGCTATGACAAATTGAGTAACGTAAATAAATCATTTATCAGTGACAGAGTGCGCAAATTGTTGGAAATATTAAAAAGTTATTCGCCCTCGAAACGTGAATTGTCTGGAATTAAAGATACACTTTTTGGATTAGTTTTTGTGAAAGAACGATTTATTGCCTTTATGATTAATAATTTGCTTCGGTTTCTTGTAAAACAAAATCCTGAAGAGTTTGGACATTTAAAAGTGGACTTTATTGTTGGACATACTGGAAATTCTGAGACAGGCGATGAAGATAGGCGTTTGGTGAATCGAAAACAAGAACAAACTCTTACAAAATTCCGAAATGGCCAATTAAATTTATTAATTACGACAAATGTTTTGGAAGAAGGAATTGATTTAAGAAATTGTAATTTAGTCGTTCGATTTGATCCGCCGATGGACTTTCGTTCATTTATTCAATCTAGCGGTCGTGCTCGAAGAGAAAATAGCGCTTTTTATATACTTATTGAAGAGAAAAATTATTTGGATTTTATGATGGACTTAACAGGGTATGCACAAGCAGAAGAGTTTGTTCTGAGACGTTATCGTTCTGGCAATGATTTTACTTTGGAAGGAAATGATGAAACAAAAATATTACATCCACATCTGGATGATGCTGTTGCTCCTTATGTTGTTACAACTGAAAAAGGGACAGCTAAAGTCTCGTTAAGTAATGCTATACAGTACTGTGCAAAATTACCATCTGATATTTTTACTCGATTAGTGCCACGTTATACAATACAAACTTTATCAGAAAATGGACAAACTTTGTATATTGCCGAATTATATCTCCCAATCAATTCACCAATAAAGGAGCCAATAAAATCTAAACCGATGAATTCTAAAAGATTATCTTTAATGGCAGTCGCATTGGAGGCTTGCAAACGATTACATCAAAGAAAAGAATTAAATGACAATCTTTTGCCTGCAGGAAAGGATATCTTGGATGATCTTTTAGGTGAAGTTGATGATGATGAATATCTGCCTCATCTACCAAGCAGAATGGGATCCTCAAAGAAAAAACGTCTCTATGATAGAAAAATGTCGAAAACTCTTAATTCTACATTACCTTCGCAAGACAGTGAATGCATTCTTTACGTTATGGAAATGAAACTGGTTAAGCCCGTTACTGATGAAGGGAATCCGAAAAGGAGAAAAATTATTGATCCGTTCGAATCAAATTCGGCATTTGGATTTTTGTCTTCGAAGGAGCTCCCGAAGATTCCGGGATTTCCAGTTTTTCAACGGAATGGTGAAATGAATGTGCAAATTCGAAAAGTAAAGAATCAACAATTTCGTCCCACTTTTGAACTTTTACAACTTATTTACCTCTTTCATCAACACATCTTTGAAGATATTTTGCGTGTTGCTCGTGGTGGTGTTGTTTTTGCACCTGGTCATTCTCCAATACCACTTTTAATTGTTCCTTTGAAAAAATTAAGCAATGCCGATTTGGACTATGAAATTGACAAAGATTACCTAAATTGGGATATTCGTGAATCTCCAACTACACCTTCAGATGAAATTAGAAAACAATTCTCTTTTGAAGAATCAAACTACTTAAATGCTGTTGTTTCACCTTGGTATCGTTCTGAAGATCAGTCTGCTTTTTATTACGTAGCTGAGATAATGACTGATCAATTTCCGTCTTCTTCATTTCCTGATGAAAAATTTACTTGTTTTAATCAATATTTTATGAGTAAATATCAATTGGAGATCTACAATCAAAAACAAAATTTACTTGATGTTGACCATACATCTGCTAGAATGAATCTTTTATTACCACGAGCAATTACTGGAAAATCAGCTACTCGTTCATTAGACCCATCTCAACGTCAAATTCTTGTTCCAGAATTGGTACATATACATCCTCTTTCTGCAACTCTTTGGTCAATTATTGTTACTCTTCCTACAATTCTTTATAGACTTAATTCTTTACTGCTTGCTGATGAATTTCGTAGCAAAGTTCTTGAAGATGCTTTAAAAATTAATTCACAAACACCCGACGATTTTGAATGGAGTCCTCTTCAATATGTCACTCTAAATGATGATCTGACACAAAAAAGTATAAGAAATTTAGATCAGTTGAGAAAAATGAACAAACAAGAGAAGGAAAATGAAGTACCAATGGAGTGTGAGAATATTGAGGAGAATGTGAATGAGACAGCTGCAAGTGGAATAGTGGGTGAATTAAAATATTTGAAATCAAATGATTTTGAAATCGGCGTTTGGGACCCTTCAGATGGCGCACAAATGTCTAGCAATGATAATACTCTGAATGAACCTTCCCCAGTCATTCATAATGCACCTGTAAATGGATTAATCCCTGGGCGGAGGAACGGTCTTCGAGGAGTTATAGCTGCTAGAGACGAGGAACTCTCTGAAATTATTGCTGTCGGGAATGATACAACACTTCATAATTATGGAGATATTTCTGATGATGATGATGTTGCAGCTGAATATGATAAATTTAAATTTTTGATGGACAATAAAATGACTACAAGTGATATTGGTGATCTAGGTGAAATGGATGTTCGACCTGCCGGGTGGAATGACGACAGCAATGCCAATGTTATCCAAATTGAAAACGAAAGTCTTCCATTGACAATATCGACAAACAATCCTCATATTAATATTGCAAGTTTAATGAATGATTTGGAGAAAAATTATGCTGCTTTTGGAACTTCAAACCATATTTCAAATAATAAAACGAATACACCGATCATTTCGAGTGATACTGCTGTTGACAAAACGGTCTTACAAAGAAAGGAGGTCCTCTTAACATATCTTATTAACAATTCGGTAAAAAGGCAAAATGAAGATATTTTACCCGAGGAAGTTTTTTGGGGAATGGATGAAGCTGAGATAGGAGAGAATATTAGCAGTGAAGCATTGACAACTTCAAGTGCTGCGGATGGAATAAATTTGGAAAGGTTAGAAACAATAGGAGATTCCTTTCTCAAAATGGCTGTCACCAACTATTTCTACTATAAACACACTGAACAACATGAAGGAAAGTTAAGCTATGCTCGTTCAAAAGAAGTTTCGAACTCCCATCTTTTTTATCTTGGACGGCAACGAGGCATCCCTCTTCTAATAGAAACACTGAAATTCGATCCACATGTTAACTGGCTTCCTCCTTGTTATGCTTCTACTTCAGAATTCCATGCTGTCAATCCTTTTGATTATACAGATTTAGATGAAGATCAATGTCAAGTGCCGATGGAGGGTGTTGATACTACTGAAACTTTAGATCAACAGCAAAAGATTAATAAAGAAACAATAGCAACTGGATGGGGAACTCTTGACGATGATAAACAGAATTATAAACGTGAAAATGGAGTAGAAACTTTAACTTTTCCACAACCAACAAAATCAGAAATTCCGGACTTACCTCCAATGCCATACAATATGCTTACTCAACAATGGATAAGTGACAAATCAATTGCTGATGCAGTAGAGGCGTTAATTGGAGCACATTTAATTCAACTTGGACAATCAGCTACTCTCAAATTTATGAATTGGTTGGGAATTAAGGTTCTAACTGATATTTCTTCATTGCCCTCACCACTTCTTCGTTTCATTGACACACCTGAAGATCCTAATCTTTCACTTAAACATCTTGCCCTTCTTTATGAGAAATTTGATTTTGCTACTGTTGAGAACAATATTGGTTATAAATTTGCAAATAAGGCATACTTAGTCCAAGCATTTACTCACGCTTCATATTATAATAATCGAGTAACCGGTTGTTACCAGCGTTTGGAATTCCTTGGCGATGCTGTTTTAGATTATATGATAACTCGTTTTCTTTATGAACACAAGAAGCAATATAGTCCGGGTGTTCTTACTGATCTTCGCTCCGCTCTTGTCAATAATACAATTTTTGCTTCGCTTGCAGTTAAATATTCTTTTCACAAACATTTTGTTATGATTTGTCCTCCACTTTATCAAATGGTCGAAAAGTTTGTTAATTTCTGTAAGCAAAAAGATTTTCTTCATTGCGCGAATTTTGACGATGAAATATTTATGTTAACTGAAGATGAAATTGATGAGGAAGACCTTTTAAGTGAAGAAGATGTCGAAGTACCTAAAGCAATGGGTGATATTTTTGAATCAATTGCTGGCGCTGTTTATTTAGATTGTGGAATGGATTTAGATATTGTTTGGCGTGTATTTTATAATTTAATGAGAGATGTTATACAAAAATGTTGTGAAAATCCACCACAATCGCCAGTTAGAGAATTATTTGAGAGAAAGAATTGTCGAGCAAAATTCTCAAAATTGGAACGAAAATTAGAAACAGGCAAAGTTAGAGTTACTGTAACGGTTAATGATAATCTCCAATTTACTGGTATGGGAAGGAGTTATCGAATTGCTAAATGTACAGCTGCTAAACGAGCACTTCAACATTTAAGAAAATTGGATGCCACAAAAACAGCAAAGAATAAATAA

**Supplementary Data S2:** Predicted protein sequences of DCRs of eight *Meloidogyne* species (*M. arenaria, M. luci, M. incognita, M. enterolobii, M. javanica, M. hapla, M.* *floridensis* and *M. graminicola)*. Accession numbers of the contig are followed by the species name and length of the protein sequence in parenthesis.

>CACSLI010000069-*M. luci* (1810aa)

MSPPKDFSGKCIPPRDYQVELLDRAKIQNTIISLGTGSGKTFVAVLLIKEYSQRLLHQNEKAVFLVNTVELVAQQAEHIEFHSSLSVARISGSTIKRKYERKEVEKITSSNQASHVLLKSSLFFVLKVIVITAQVFLDLINHGLFDFSSLALLIVDECHHCLGELHPYRLIMNHYKKLQGQRPRVLGLTASILNKKVPSSRIECTAQLLEQIMDSRIETSSNYTQERLRSFIEKNEDFHSELEVDPRRKIFETISRSFSTLQQVGSWAALKGFILWQKNLLKHVDDPIIGNKQKCILRMAETAFRTCSKVLSHKINPLNSYDKLVNVNKSFISDRVRKLLEILKSYSPSKRELSGIKDTLFGLVFVKERFIAFMINNLLRFLVKQNPEEFGHLKVDFIVGHTGNSETGDEDRRLVNRKQEQTLTKFRNGQLNLLITTNVLEEGIDLRNCNLVVRFDPPMDFRSFIQSSGRARRENSAFYILIEEKNYLEFMMDLTGYAQAEEFVLRRYRSGNDFTLEGNDETKLLHPHLDDAVAPYVVTTEKGAAKVSLSNAIQYCAKLPSDIFTRLVPRYTIQTLSENGQTLYIAELYLPINSPIKEPIKSKPMTSKRLSLMAVALEACKRLHQRKELNDNLLPAGKDILDDLLGEVDDDEYLPHLPSRMGSSKKKRLYDRKMSKTLNSTLPSQDSECVLYVMEMKLIKPVTDEGNPKRRKIIDPFESNSAFGFLSSKELPKVPGFPVFQRNGEMNVQIRKAKNQQFRPTFELLQLIYLFHQHIFEDILRVARGGVVFAPGHSPIPLLIVPLKKLSTADLDYEIDRDYLNWNIRESPTTPSDEIRKQFSFEESNYLNAVVSPWYRSEDQSAFYYVAEIMTDQFPSSSFPDEKFACFNQYFMSKYQLEIYNQKQNLLDVDHTSARMNLLLPRAISGKSATRSLDPSQRQILVPELVHIHPLSATLWSIIVTLPTILYRLNSLLLADEFRSKVLEDALKINSQTPDDFEWSPLQYVTLNDDLTQKSIRNLDQLRKMNKQEKENEVPMECDIIEENVNETAASGIVGRLKFLKSNDFEIGVWDPSDGAQMSNNDNTPNEPSPVIHNAPVNGLIPGRRNGLRGVIAARDEELSEIIAVGNDTTLHNYGDISDDDDVAAEYDKFKFLMDNKMTTSDIGDLGEMDVRPAGWNDDSNANVIQIENESLPLTISTNNPHINIASLMNDLEKNYAAFGTSNHISNNNKTNTPNIPNATADKTVLQKKEVLLTYLINNSVKRQNEDILPEEVFWGMDEAEIGENISSEALTTSSAADGINLERLETIGDSFLKMAVTNYFYYKHTEQHEGKLSYARSKEVSNSHLFYLGRQRGIPLLIETLKFDPHVNWLPPCYASTSEFHAVNPFDYTDLDEDQCAVPMEGVDTTETVDQQQKINKETIATGWGTIDDDKQNYKRENGVETLTFPQPTKSEIPDLPPMPYNMLTQQWISDKSIADAVEALIGAHLIQLGQSATLKFMNWLGIKVLTDISSLPSPLLRFIDTPEDPNLSLKHLALLYEKFDFATVENNIGYKFANKAYLVQAFTHASYYNNRVTGCYQRLEFLGDAVLDYMITRFLYEHKKQYSPGVLTDLRSALVNNTIFASLAVKYSFHKHFVMICPPLYQMVEKFVNFCKQKDFLHCANFDDEIFMLTEDEIDEEDLLSEEDVEVPKAMGDIFESIAGAVYLDCGMDLDIVWRVFYNLMRDVIQKCCENLPQSPVRELFERKNCRAKFSKLERKLETGKVRVTVTVNDNLQFTGMGRSYRIAKCTAAKRALQHLRKLDATKTAKNK

>CACSLI010000224-*M. luci* (1804aa)

MSPPKDFSGKCIPPRDYQFKFKVELLDRAKIQNTIISLGTGSGKTFVAVLLIKEYSQRLLHQNEKAVFLVNTVELVAQQAEHIEFHSSLSVARISGSTIKRKYEKKEVEKITSSNQASHVIVITAQVFLDLINHGLFDFSSLALLIVDECHHCLGELHPYRLVMNHYKKLQGQRPRVLGLTASILNKKVPSSRIECTAQLLEQIMDSQIETSSNYTQERLRSFIEKNEDFHSELEVDPRRKIFETISRSFSTLQQVGSWAALKGFILWQKNLLKHVDDPIIGNKQKCILRMAETAFRTCSKVLSHKINPLNSYDKLSNVNKSFISDRVRKLLEILKSYSPSKRELSGIKDTLFGLVFVKERFIAFMINNLLRFLVKQNPEEFGHLKVDFIVGHTGNSETGDEDRRLVNRKQEQTLTKFRNGQLNLLITTNVLEEGIDLRNCNLVVRFDPPMDFRSFIQSSGRARRENSAFYILIEEKNYLDFMMDLTGYAQAEEFVLRRYRSGNDFTLEGNDETKILHPHLDDAVAPYVVTTEKGTAKVSLSNAIQYCAKLPSDIFTRLVPRYTIQTLSENGQTLYIAELYLPINSPIKEPIKSKPMNSKRLSLMAVALEACKRLHQRKELNDNLLPAGKDILDDLLGEVDDDEYLPHLPSRMGSSKKKRLYDRKMSKTLNSTLPSQDSECILYVMEMKLIKPVTDEGNPKRRKIIDPFESNSAFGFLSSKELPKIPGFPVFQRNGEMNVQIRKVKNQQFRPNFELLQLIYLFHQHIFEDILRVARGGVVFAPGHSPIPLLIVPLKKLSTADLDYEIDKDYLNWNIRESPTTPSDEIRKQFSFEESNYLNAVVSPWYRSEDQSAFYYVAEIMTDQFPSSSFPDEKFTCFNQYFMSKYQLEIYNQKQNLLDVDHTSARMNLLLPRAITGKSATRSLDPSQRQILVPELVHIHPLSATLWSIIVTLPTILYRLNSLLLADEFRSKVLEDALKINSRTPDDFEWSPLQYVTLNDDLTQKSIRNLDQLRKMNKQEKENEVPMECENIEENVNETAANGIVGNFKILRSIINDFEIGVWDPSDGAQMSNNDNTPNEPSPVIHNAPVNGLIPGRRNGLRGVIAARDEELSEIIAVGNDTTLHNYGDISDDDDVAAEYDKFKFLMDNKMTTSDIGDLGEMDVRPAGWNDDSNANVIQIENESLPLTISTNNPHINIASLMNDLEKNYAAFGTSNHIINNKTNTPIISSDTAVDKTVLQRKEVLLTYLINNSVKRQNEDILPEEVFWGMDEAEIGENISSEALTTSSAADGINLERLETIGDSFLKMAVTNYFYYKHTEQHEGKLSYARSKEVSNSHLFYLGRQRGIPLLIETLKFDPHVNWLPPCYASTSEFHAVNPFDYTDLDEDQCAVPMEGVDTTETVDQQQKINKETIATGWGTLDDDKQNYKRENGVETLTFPQPTKSEIPDLPPMPYNMLTQQWISDKSIADAVEALIGAHLIQLGQSATLKFMNWLGIKVLTDISSLPSPLLRFIDTPEDPNLSLKHLALLYEKFDFATVENNIGYKFANKAYLVQAFTHASYYNNRVTGCYQRLEFLGDAVLDYMITRFLYEHKKQYSPGVLTDLRSALVNNTIFASLAVKYSFHKHFVMICPPLYQMVEKFVNFCKQKDFLHCANFDDEIFMLTEDEIDEEDLLSEEDVEVPKAMGDIFESIAGAVYLDCGMDLDIVWRVFYNLMRDVIQKCCENPPQSPVRELFERKNCRAKFSKLERKLETGKVRVTVTVNDNLQFTGMGRSYRIAKCTAAKRALQHLRKLDATKTAKNK

>CACSLI010000297-*M. luci* (1806aa)

MSPPKDFSGKCIPPRDYQFKFKVELLDRAKIQNTIISLGTGSGKTFVAVLLIKEYSQRLLHQNEKAVFLVNTVELVAQQAEHIEFHSSLSVARISGSTIKRKYERKEVEKITNTNQASHVIVITAQVFLDLINHGLFDFSSLALLIVDECHHCLGELHPYRLIMNHYKKLQGQRPRVLGLTASILNKKVPSSRIECTAQLLEQIMDSQIETSSNYTQERLRSFIEKNEDFHSELEVDPRRKIFETISRSFSTLQQVGSWAALKGFILWQKNLLKHVDDPIIGNKQKCILRMAETAFRTCSKVLSHKINPLNSYDKLGNVNKSYISDRVRKLLEILKSYSPSKRELSGIKDTLFGLVFVKERFIAFMINNLLRFLVKQNPEEFGHLKVDFIVGHTGNSETGDEDRRLVNRKQEQTLTRFRNGQLNLLITTNVLEEGIDLRNCNLVIRFDPPMDFRSFIQSSGRARRENSAFYILIEEKNYLDFMMDLTGYAQAEEFVLRRYRSGNDFTLEGNDETKILHPHLDDAVAPYVVTTEKGTAKVSLSNAIQLVNRYCAKLPSDIFTRLVPRYTIQTLSENGQTLYIAELYLPINSPIKEPIKSKPMTSKRLSLMAVALEACKRLHQRKELNDNLLPAGKDILDDLLGEVDDDEYLPHLPSRMGSSKKKRLYDRKMSKTLNSTLPSQDSECILYVMEMKLIKPVTDEGNPKRRKIIDPFESNSAFGFLSSKELPKIPGFPVFQRNGEMNVQIRKVKNQQFRPTFELLQLIYLFHQHIFEDILRVARGGVVFAPGHSPIPLLIVPLKKLSTADLDYEIDRDYLNWDIRESPTTPSDEIRKQFSFEESNYLNAVVSPWYRSEDQSAFYYVAEIMTDQFPSSSFPDEKFTCFNQYFMSKYQLEIYNQKQNLLDVDHTSARMNLLLPRAITGKSATRSLDPSQRQILVPELVHIHPLSATLWSIIVTLPTILYRLNSLLLADEFRSKVLEDALKINSQTPDDFEWSPLQYVTLNDDLTQKSIRNLDQLRKMNKQEKENEVPMECDIIEENVNETAASGIVGKLKYLKSNDFEIGVWDPSDGAQMSSNDNTLNEPSPVIHNAPVNGIIPGRRNGLRGVIAARDEELSEIIAVGNDTTLHNYGDISDDDDVAAEYDKFKFLMDNKMTTSDIGDLGEMDVRPAGWNDDSNANVIQIENESLPLTISTNNPHINIASLMNDLEKNYAAFGTSNHISNNKTNTPIISSDTAVDKTVLQRKEVLLTYLINNSVKRQNEDILPEEVFWGMDEAEIGENISSEALTTSSAADGINLERLETIGDSFLKMAVTNYFYYKHTEQHEGKLSYARSKEVSNSHLFYLGRQRGIPLLIETLKFDPHVNWLPPCYASTSEFHAVNPFDYTDLDEDQCQVPMEGVDTTETVDQQQKINKETIATGWGTLDDDKQNYKRENGVETLTFPQPTKSEIPDLPPMPYNMLTQQWISDKSIADAVEALIGAHLIQLGQSATLKFMNWLGIKVLTDISSLPSPLLRFIDTPEDPNLSLKHLALLYEKFDFATVENNIGYKFANKAYLVQAFTHASYYNNRVTGCYQRLEFLGDAVLDYMITRFLYEHKKQYSPGVLTDLRSALVNNTIFASLAVKYSFHKHFVMICPPLYQMVEKFVNFCKQKDFLHCANFDDEIFMLTEDEIDEEDLLSEEDVEVPKAMGDIFESIAGAVYLDCGMDLDIVWRVFYNLMRDVIQKCCENPPQSPVRELFERKNCRAKFSKLERKLETGKVRVTVTVNDNLQFTGMGRSYRIAKCTAAKRALQHLRKLDATKTAKNK

>CEWM01000731-*M. arenaria* (1810aa)

MSPPKDFSGKCIPPRDYQVELLDRAKIQNTIISLGTGSGKTFVAVLLIKEYSQRLLHQNEKAVFLVNTVELVAQQAEHIEFHSSLSVARISGSTIKRKYERKEVEKITSSNQASHVLLKSSLFFVLKVIVITAQVFLDLINHGLFDFSSLALLIVDECHHCLGELHPYRLIMNHYKKLQGQRPRVLGLTASILNKKVPSSRIECTAQLLEQIMDSQIETSSNYTQERLRSFIERNEDFHSELEVDPRRKIFETISRSFSTLQQVGSWAALKGFILWQKNLLKHVDDPIIGNKQKCILRMAETAFRTCSKVLSHKINPLNSYDKLVNVNKSFISDRVRKLLEILKSYSPSKRELSGIKDTLFGLVFVKERFIAFMINNLLRFLVKQNPEEFGHLKVDFIVGHTGNSETGDEDRRLVNRKQEQTLTKFRNGQLNLLITTNVLEEGIDLRNCNLVVRFDPPMDFRSFIQSSGRARRENSAFYILIEEKNYLEFMMDLTGYAQAEEFVLRRYRSGNDFTLEGNDETKLLHPHLDDAVAPYVVTTEKGTAKVSLSNAIQYCAKLPSDIFTRLVPRYTVQTLSENGQTLYIAELYLPINSPIKEPIKSKPMTSKRLSLMAVALEACKRLHQRKELNDNLLPAGKDILDDLLGEVDDDEYLPHLPSRMGSSKKKRLYDRKMSKTLNSTLPSQDSECVLYVMEMKLIKPVTDEGNPKRRKIIDPFESNSAFGFLSSKELPKVPGFPVFQRNGEMNVQIRKVKNQQFRPTFELLQLIYLFHQHIFEDILRVARGGVVFAPGHSPIPLLIVPLKKLSTADLDYEIDRDYLNWNIRESPTTPSDEIRKQFSFEESNYLNAVVSPWYRSEDQSAFYYVAEIMTDQFPSSSFPDEKFACFNQYFMSKYQLEIYNQKQNLLDVDHTSARMNLLLPRAISGKSATRSLDPSQRQILVPELVHIHPLSATLWSIIVTLPTILYRLNSLLLADEFRSKVLEDALKINSQTPDDFEWSPLQYVTLNDDLTQKSIRNLDQLRKMNKQEKENEVPMECDIIEENVNETAASGIVGRLKFLKSNDFEIGVWDPSDGAQMSNNDNTPNEPSPVIHNAPVNGLIPGRRNGLRGVIAARDEELSEIIAVGNDTTLHNYGDISDDDDVAAEYDKFKFLMDNKMTTSDIGDLGEMDVRPAGWNDDSNANVIQIENESLPLTISTNNPHINIASLMNDLEKNYAAFGTSNHISNNNKTNTPNIPNATADKTDLQKKEVLLTYLINNSVKRQNEDILPEEVFWGMDEAEIGENISSEALTTSSAADGINLERLETIGDSFLKMAVTNYFYYKHTEQHEGKLSYARSKEVSNSHLFYLGRQRGIPLLIETLKFDPHVNWLPPCYASTSEFHAVNPFDYTDLDEDQCQVPMEGVDTTETLDQQQKTNKETIATGWGTLDDDKQNYKRENGVETLTFPQPTKSEIPDLPPMPYNMLTQQWISDKSIADAVEALIGAHLIQLGQSATLKFMNWLGIKVLTDISSLPSPLLRFIDTPEDPNLSLKHLALLYEKFDFATVENNIGYKFANKAYLVQAFTHASYYNNRVTGCYQRLEFLGDAVLDYMITRFLYEHKKQYSPGVLTDLRSALVNNTIFASLAVKYSFHKHFVMICPPLYQMVEKFVNFCKQKDFLHCANFDDEIFMLTEDEIDEEDLLSEEDVEVPKAMGDIFESIAGAVYLDCGMDLDIVWRVFYNLMRDVIQKCCENPPQSPVRELFERKNCRAKFSKLERKLETGKVRVTVTVNDNLQFTGMGRSYRIAKCTAAKRALQHLRKLDATKTARNK

>CEWM01002306*- M. arenaria* (1818aa)

MSPPKDFSGKCIPPRDYQFKFKVELLDRAKIQNTIISLGTGSGKTFVAVLLIKEYSQRLLHQNEKAVFLVNTVELVAQQAEHIEFHSSLSVARISGSTIKRKYERKEVEKITSSNQASHVLLKFSLFFVLKVIVITAQVFLDLINHGLFDFSSLALLIVDECHHCLGELHPYRLIMNHYKKLQGQRPRVLGLTASILNKKVPSSRIECTAQLLEQIMDSQIETSSNYTQERLRSFIEKNEDFHSELEVDPRRKIFETISRSFSTLQQVGSWAALKGFILWQKNLLKHVDDPIIGNKQKCILRMAETAFRTCSKVLSHKINPLNSYDKLGNVNKSYISDRVRKLLEILKSYSPSKREFSGIKDTLFGLVFVKERFIAFMINNLLRFLVKQNPEEFGHLKVDFIVGHTGNSETGDEDRRLVNRKQEQTLTRFRNGQLNLLITTNVLEEGIDLRNCNLVIRFDPPMDFRSFIQSSGRARRENSAFYILIEEKNYLDFMMDLTGYAQAEEFVLRRYRSGNDFTLEGNDETKILHPHLDDAVAPYVVTTEKGTAKVSLSNAIQLVNRYCAKLPSDIFTRLVPRYTIQTLSENGQTLYIAELYLPINSPIKEPIKSKPMTSKRLSLMAVALEACKRLHQRKELNDNLLPAGKDILDDLLGEVDDDEYLPHLPSRMGSSKKKRLYDRKMSKTLNSTLPSQDSECILYVMEMKLIKPVTDEGNPKRRKIIDPFESNSAFGFLSSKELPKIPGFPVFQRNGEMNVQIRKVKNQQFRPTFELLQLIYLFHQHIFEDILRVARGGVVFAPGHSPIPLLIVPLKKLSTADLDYEIDRDYLNWDIRESPTTPSDEIRKQFSFEESNYLNAVVSPWYRSEDQSAFYYVAEIMTDQFPSSSFPDEKFTCFNQYFMSKYQLEIYNQKQNLLDVDHTSARMNLLLPRAITGKSATRSLDPSQRQILVPELVHIHPLSATLWSIIVTLPTILYRLNSLLLADEFRSKVLEDALKINSQTPDDFEWSPLQYVTLNDDLTQKSIRNLDQLRKMNKQEKENEVPMECDIIEENVNETAASGIVGKLKYLKSNDFEIGVWDPSDGAQMSNNDNTLNEPSPVIHNAPVNGIIPGRRNGLRGVIAARDEELSEIIAVGNDTTLHNYGDISDDDDVAAEYDKFKFLMDNKMTTSDIGDLGEMDVRPAGWNDDSNANVIQIENESLPLTISTNNPHINIASLMNDLEKNYAAFGTSNHISNNKTNTPIISSDTAVDKTVLQRKEVLLTYLINNSVKRQNEDILPEEVFWGMDEAEIGENISSEALTTSSAADGINLERLETIGDSFLKMAVTNYFYYKHTEQHEGKLSYARSKEVSNSHLFYLGRQRGIPLLIETLKFDPHVNWLPPCYASTSEFHAVNPFDYTDLDEDQCQVPMEGVDTTETVDQQQKINKETIATGWGTLDDDKQNYKRENGVETLTFPQPTKSEIPDLPPMPYNMLTQQWISDKSIADAVEALIGAHLIQLGQSATLKFMNWLGIKVLTDISSLPSPLLRFIDTPEDPNLSLKHLALLYEKFDFATVENNIGYKFANKAYLVQAFTHASYYNNRVTGCYQRLEFLGDAVLDYMITRFLYEHKKQYSPGVLTDLRSALVNNTIFASLAVKYSFHKHFVMICPPLYQMVEKFVNFCKQKDFLHCANFDDEIFMLTEDEIDEEDLLSEEDVEVPKAMGDIFESIAGAVYLDCGMDLDIVWRVFYNLMRDVIQKCCENPPQSPVRELFERKNCRAKFSKLERKLETGKVRVTVTVNDNLQFTGMGRSYRIAKCTAAKRALQHLRKLDATKTAKNK

>QEUI01000193- *M. arenaria* (1798aa)

MSPPKDFSGKCIPPRDYQVELLDRAKIQNTIISLGTGSGKTFVAVLLIKEYSQRLLHQNEKAVFLVNTVELVAQQAEHIEFHSSLSVARISGSTIKRKYERKEVEKITSSNQASHVLLKSSLFFVLKVIVITAQLFDFSSLALLIVDECHHCLGELHPYRLIMNHYKKLQGQRPRVLGLTASILNKKVPSSRIECTAQLLEQIMDSQIETSSNYTQERLRSFIEKNEDFHSELEVDPRRKIFETISRSFSTLQQVGSWAALKGFILWQKNLLKHVDDPIIGNKQKCILRMAETAFRTCSKVLSHKINPLNSYDKLVNVNKSFISDRVRKLLEILKSYSPSKRELSGIKDTLFGLVFVKERFIAFMINNLLRFLVKQNPEEFGHLKVDFIVGHTGNSETGDEDRRLVNRKQEQTLTKFRNGQLNLLITTNVLEEGIDLRNCNLVVRFDPPMDFRSFIQSSGRARRENSAFYILIEEKNYLEFMMDLTGYAQAEEFVLRRYRSGNDFTLEGNDETKLLHPHLDDAVAPYVVTTEKGTAKVSLSNAIQYCAKLPSDIFTRLVPRYTIQTLSENGQTLYIAELYLPINSPIKEPIKSKPMTSKRLSLMAVALEACKRLHQRKELNDNLLPAGKDILDDLLGEVDDDEYLPHLPSRMGSSKKKRLYDRKMSKTLNSTLPSQDSECVLYVMEMKLIKPVTDEGNPKRRKIIDPFESNSAFGFLSSKELPKVPGFPVFQRNGEMNVQIRKVKNQQFRPTFELLQLIYLFHQHIFEDILRVARGGVVFAPGHSPIPLLIVPLKKLSTADLDYEIDRDYLNWNIRESPTTPSDEIRKQFSFEESNYLNAVVSPWYRSEDQSAFYYIMTDQFPSSSFPDEKFACFNQYFMSKYQLEIYNQKQNLLDVDHTSARMNLLLPRAISGKSATRSLDPSQRQILVPELVHIHPLSATLWSIIVTLPTILYRLNSLLLADEFRSKVLEDALKINSQTPDDFEWSPLQYVTLNDDLTQKSIRNLDQLRKMNKQEKENEVPMECDIIEENVNETAASGIVGRLKFLKSNDFEIGVWDPSDGAQMSNNDNTPNEPSPVIHNAPVNGLIPGRRNGLRGVIAARDEELSEIIAVGNDTTLHNYGDISDDDDVAAEYDKFKFLMDNKMTTSDIGDLGEMDVRPAGWNDDSNANVIQIENESLPLTISTNNPHINIASLMNDLEKNYAAFGTSNHISNNNKTNTPNIPNATADKTDLQKKEVLLTYLINNSVKRQNEDILPEEVFWGMDEAEIGENISSEALTTSSAADGINLERLETIGDSFLKMAVTNYFYYKHTEQHEGKLSYARSKEVSNSHLFYLGRQRGIPLLIETLKFDPHVNWLPPCYASTSEFHAVNPFDYTDLDEDQCAVPMEGVDTTETVDQQQKINKETIATGWGTIDDDKQNYKRENGVETLTFPQPTKSEIPDLPPMPYNMLTQQWISDKSIADAVEALIGAHLIQLGQSATLKFMNWLGIKVLTDISSLPSPLLRFIDTPEDPNLSLKHLALLYEKFDFATVENNIGYKFANKAYLVQAFTHASYYNNRVTGCYQRLEFLGDAVLDYMITRFLYEHKKQYSPGVLTDLRSALVNNTIFASLAVKYSFHKHFVMICPPLYQMVEKFVNFCKQKDFLHCANFDDEIFMLTEDEIDEEDLLSEEDVEVPKAMGDIFESIAGAVYLDCGMDLDIVWRVFYNLMRDVIQKCCENPPQSPVRELFERKNCRAKFSKLERKLETGKVRVTVTVNDNLQFTGMGRSYRIAKCTAAKRALQHLRKLDATKTAKNK

>QEUI01000369- *M. arenaria* (1815aa)

MSPPKDFSGKCIPPRDYQFKFKVELLDRAKIQNTIISLGTGSGKTFVAVLLIKEYSQRLLHQNEKAVFLVNTVELVAQQAEHIEFHSSLSVARISGSTIKRKYERKEVEKITSSNQASHVLLKFSLFFVLKVIVITAQVFLDLINHGLFDFSSLALLIVDECHHCLGELHPYRLIMNHYKKLQGQRPRVLGLTASILNKKVPSSRIECTAQLLEQIMDSQIETSSNYTQERLRSFIEKNEDFHSELEVDPRRKIFETISRSFSTLQQVGSWAALKGFILWQKNLLKHVDDPIIGNKQKCILRMAETAFRTCSKVLSHKINPLNSYDKLSNVNKSFISDRVRKLLEILKSYSPSKRELSGIKDTLFGLVFVKERFIAFMINNLLRFLVKQNPEEFGHLKVDFIVGHTGNSETGDEDRRLVNRKQEQTLTKFRNGQLNLLITTNVLEEGIDLRNCNLVVRFDPPMDFRSFIQSSGRARRENSAFYILIEEKNYLDFMMDLTGYAQAEEFVLRRYRSGNDFTLEGTDETKIIHPHLDDAVAPYVVTTEKGTAKVSLSNAIQYCAKLPSDIFTRLVPRYTIQTLSENGQTLYIAELYLPINSPIKEPIKSKPMNSKRLSLMAVALEACKRLHQRKELNDNLLPAGKDILDDLLGEVDDDEYLPHLPSRMGSSKKKRLYDRKMSKTLNSTLPSQDSACFLYVMEMKLIKPVTDEGNPKRRKIIDPFESNSAFGFLSSKELPKVQGFPVFQRNGEMNVQIRKVKNQQFRPNFELLQLIYLFHQHIFEDILRVARGGVVFAPGHSPIPLLIVPLKKLSTADLDYEIDKDYLNWDIRESPTTPSDEIRKQFSFEESNYLNAVVSPWYRSEDQSAFYYVAEIMTDQFPSSSFPDEKFTCFNQYFMSKYQLEIYNQKQNLLDVDHTSARMNLLLPRAITGKSATRSLDPSQRQILVPELVHIHPLSATLWSIIVTLPTILYRLNSLLLADEFRSKVLEDALKINSQTPDDFEWSPLRYVTLNDDLTQKSIRNLDQLRKMNKQEKENEVPMECENIEENVNETAASGIVGELKNFRSNDFEIGVWDPSDGAQMSNNDNTLNEPSPVIHNAPVNGLIPGRRNGLRGVIAARDEELSEIIAVGNDTTLHNYGDISDDDDVAAEYDKFKFLMDNKMTTSDIGDLGEMDVRPAGWNDDSNANVIQIENESLPLTISTNNPHINIASLMNDLEKNYAAFGTSSHISNNTRTNTPIISSDTAVDKTALQRKEVLLTYLTNNSVKRQNEDILPEEVFWGMDEAEIGENISSEALTTSSAADGINLERLETIGDSFLKMAVTNYFYYKHTEQHEGKLSYARSKEVSNSHLFYLGRQRGIPLLIETLKFDPHVNWLPPCYASTSEFHAVNPFDYTDLDEDQCQVPMEGVDTTETLDQQQKTNKETIATGWGTLDDDKQNYKRENGVETLTFPQPTKSEIPDLPPMPYNMLTQQWISDKSIADAVEALIGAHLIQLGQSATLKFMNWLGIKVLTDISSLPSPLLRFIDTPEDPNLSLKHLALLYEKFDFATVENNIGYKFANKAYLVQAFTHASYYNNRVTGCYQRLEFLGDAVLDYMITRFLYEHKKQYSPGVLTDLRSALVNNTIFASLAVKYSFHKHFVMICPPLYQMVEKFVNFCKQKDFLHCANFDDEIFMLTEDEIDEEDLLSEEDVEVPKAMGDIFESIAGAVYLDCGMDLDIVWRVFYNLMRDVIQKCCENPPQSPVRELFERKNCRAKFSKLERKLETGKVRVTVTVNDNLQFTGMGRSYRIAKCTAAKRALQHLRKLDATKTARNK

>RCFJ01030829- *M. arenaria* (1791aa)

MSPPKDFSGKCIPPRDYQFKFKVELLDRAKIQNTIISLGTGSGKTFVAVLLIKEYSQRLLHQNEKAVFLVNTVELVAQQAEHIEFHSSLSVARISGSTIKRKYERKEVEKITNTNQASHLFDFSSLALLIVDECHHCLGELHPYRLIMNHYKKLQGQRPRVLGLTASILNKKVPSSRIECTAQLLEQIMDSQIETSSNYTQERLRSFIEKNEDFHSELEVDPRRKIFETISRSFSTLQQVGSWAALKGFILWQKNLLKHVDDPIIGNKQKCILRMAETAFRTCSKVLSHKINPLNSYDKLGNVNKSYISDRVRKLLEILKSYSPSKRELSGIKDTLFGLVFVKERFIAFMINNLLRFLVKQNPEEFGHLKVDFIVGHTGNSETGDEDRRLVNRKQEQTLTRFRNGQLNLLITTNVLEEGIDLRNCNLVIRFDPPMDFRSFIQSSGRARRENSAFYILIEEKNYLDFMMDLTGYAQAEEFVLRRYRSGNDFTLEGNDETKILHPHLDDAVAPYVVTTEKGTAKVSLSNAIQLVNRYCAKLPSDIFTRLVPRYTIQTLSENGQTLYIAELYLPINSPIKEPIKSKPMTSKRLSLMAVALEACKRLHQRKELNDNLLPAGKDILDDLLGEVDDDEYLPHLPSRMGSSKKKRLYDRKMSKTLNSTLPSQDSECILYVMEMKLIKPVTDEGNPKRRKIIDPFESNSAFGFLSSKELPKIPGFPVFQRNGEMNVQIRKVKNQQFRPTFELLQLIYLFHQHIFEDILRVARGGVVFAPGHSPIPLLIVPLKKLSTADLDYEIDRDYLNWDIRESPTTPSDEIRKQFSFEESNYLNAVVSPWYRSEDQSAFYYVAEIMTDQFPSSSFPDEKFTCFNQYFMSKYQLEIYNQKQNLLDVDHTSARMNLLLPRAITGKSATRSLDPSQRQILVPELVHIHPLSATLWSIIVTLPTILYRLNSLLLADEFRSKVLEDALKINSQTPDDFEWSPLQYVTLNDDLTQKSIRNLDQLRKMNKQEKENEVPMECENIEENVNETAASGIVGELKNFRSNDFEIGVWDPSDGAQMSNNDNTLNEPSPVIHNAPVNGLIPGRRNGLRGVIAARDEELSEIIAVGNDTTLHNYGDISDDDDVAAEYDKFKFLMDNKMTTSDIGDLGEMDVRPAGWNDDSNANVIQIENESLPLTISTNNPHINIASLMNDLEKNYAAFGTSSHISNNTRTNTPIISSDTAVDKTALQRKEVLLTYLTNNSVKRQNEDILPEEVFWGMDEAEIGENISSEALTTSSAADGINLERLETIGDSFLKMAVTNYFYYKHTEQHEGKLSYARSKEVSNSHLFYLGRQRGIPLLIETLKFDPHVNWLPPCYASTSEFHAVNPFDYTDLDEDQCQVPMEGVDTTETLDQQQKTNKETIATGWGTLDDDKQNYKRENGVETLTFPQPTKSEIPDLPPMPYNMLTQQWISDKSIADAVEALIGAHLIQLGQSATLKFMNWLGIKVLTDISSLPSPLLRFIDTPEDPNLSLKHLALLYEKFDFATVENNIGYKFANKAYLVQAFTHASYYNNRVTGCYQRLEFLGDAVLDYMITRFLYEHKKQYSPGVLTDLRSALVNNTIFASLAVKYSFHKHFVMICPPLYQMVEKFVNFCKQKDFLHCANFDDEIFMLTEDEIDEEDLLSEEDVEVPKAMGDIFESIAGAVYLDCGMDLDIVWRVFYNLMRDVIQKCCENPPQSPVRELFERKNCRAKFSKLERKLETGKVRVTVTVNDNLQFTGMGRSYRIAKCTAAKRALQHLRKLDATKTAKNK

>CABB01000157-*M. incognita* (1803aa)

MSPPRDFSGKCIPPRDYQFKFKVELLDRAKIQNTIISLGTGSGKTFVAVLLIKEYSQRLLHQNEKAVFLVNTVELVAQQAEHIEFHSSLSVARISGSTIKRKYERKEVEKITNTNQASHVIVITAQVFLDLINHGLFDFSSLALLIVDECHHCLGELHPYRLIMNHYKKLQGQRPRVLGLTASILNKKVPSSRIECTAQLLEQIMDSQIETSSNYTQERLRSFIEKNEDFHSELEVDPRRKIFETISRSFSTLQQVGSWAALKGFILWQKNLLKHVDDPIIGNKQKCILRMAETAFRTCSKVLSHKINPLNSYDKLSNVNKSFISDRVRKLLEILKSYSPSKRELSGIKDTLFGLVFVKERFIAFMINNLLRFLVKQNPEEFGHLKVDFIVGHTGNSETGDEDRRLVNRKQEQTLTKFRNGQLNLLITTNVLEEGIDLRNCNLVVRFDPPMDFRSFIQSSGRARRENSAFYILIEEKNYLDFMMDLTGYAQAEEFVLRRYRSGNDFTLEGNDETKILHPHLDDAVAPYVVTTEKGTAKVSLSNAIQYCAKLPSDIFTRLVPRYTIQTLSENGQTLYIAELYLPINSPIKEPIKSKPMNSKRLSLMAVALEACKRLHQRKELNDNLLPAGKDILDDLLGEVDDDEYLPHLPSRMGSSKKKRLYDRKMSKTLNSTLPSQDSECILYVMEMKLVKPVTDEGNPKRRKIIDPFESNSAFGFLSSKELPKIPGFPVFQRNGEMNVQIRKVKNQQFRPTFELLQLIYLFHQHIFEDILRVARGGVVFAPGHSPIPLLIVPLKKLSNADLDYEIDKDYLNWDIRESPTTPSDEIRKQFSFEESNYLNAVVSPWYRSEDQSAFYYVAEIMTDQFPSSSFPDEKFTCFNQYFMSKYQLEIYNQKQNLLDVDHTSARMNLLLPRAITGKSATRSLDPSQRQILVPELVHIHPLSATLWSIIVTLPTILYRLNSLLLADEFRSKVLEDALKINSQTPDDFEWSPLQYVTLNDDLTQKSIRNLDQLRKMNKQEKENEVPMECENIEENVNETAASGIVGELKNFRSNDFEIGVWDPSDGAQMSNNDNTLNEPSPVIHNAPVNGLIPGRRNGLRGVIAARDEELSEIIAVGNDTTLHNYGDISDDDDVAAEYDKFKFLMDNKMTTSDIGDLGEMDVRPAGWNDDSNANVIQIENESLPLTISTNNPHINIASLMNDLEKNYAAFGTSSHISNNTRTNTPIISSDTAVDKTALQRKEVLLTYLTNNSVKRQNEDILPEEVFWGMDEAEIGENISSEALTTSSAADGINLERLETIGDSFLKMAVTNYFYYKHTEQHEGKLSYARSKEVSNSHLFYLGRQRGIPLLIETLKFDPHVNWLPPCYASTSEFHAVNPFDYTDLDEDQCQVPMEGVDTTETLDQQQKTNKETIATGWGTLDDDKQNYKRENGVETLTFPQPTKSEIPDLPPMPYNMLTQQWISDKSIADAVEALIGAHLIQLGQSATLKFMNWLGIKVLTDISSLPSPLLRFIDTPEDPNLSLKHLALLYEKFDFATVENNIGYKFANKAYLVQAFTHASYYNNRVTGCYQRLEFLGDAVLDYMITRFLYEHKKQYSPGVLTDLRSALVNNTIFASLAVKYSFHKHFVMICPPLYQMVEKFVNFCKQKDFLHCANFDDEIFMLTEDEIDEEDLLSEEDVEVPKAMGDIFESIAGAVYLDCGMDLDIVWRVFYNLMRDVIQKCCENPPQSPVRELFERKNCRAKFSKLERKLETGKVRVTVTVNDNLQFTGMGRSYRIAKCTAAKRALQHLRKLDATKTAKNK

>FXSY01000007- *M. incognita* (1803aa)

MSPPRDFSGKCIPPRDYQFKFKVELLDRAKIQNTIISLGTGSGKTFVAVLLIKEYSQRLLHQNEKAVFLVNTVELVAQQAEHIEFHSSLSVARISGSTIKRKYERKEVEKITNTNQASHVIVITAQVFLDLINHGLFDFSSLALLIVDECHHCLGELHPYRLIMNHYKKLQGQRPRVLGLTASILNKKVPSSRIECTAQLLEQIMDSQIETSSNYTQERLRSFIEKNEDFHSELEVDPRRKIFETISRSFSTLQQVGSWAALKGFILWQKNLLKHVDDPIIGNKQKCILRMAETAFRTCSKVLSHKINPLNSYDKLSNVNKSFISDRVRKLLEILKSYSPSKRELSGIKDTLFGLVFVKERFIAFMINNLLRFLVKQNPEEFGHLKVDFIVGHTGNSETGDEDRRLVNRKQEQTLTKFRNGQLNLLITTNVLEEGIDLRNCNLVVRFDPPMDFRSFIQSSGRARRENSAFYILIEEKNYLDFMMDLTGYAQAEEFVLRRYRSGNDFTLEGNDETKILHPHLDDAVAPYVVTTEKGTAKVSLSNAIQYCAKLPSDIFTRLVPRYTIQTLSENGQTLYIAELYLPINSPIKEPIKSKPMNSKRLSLMAVALEACKRLHQRKELNDNLLPAGKDILDDLLGEVDDDEYLPHLPSRMGSSKKKRLYDRKMSKTLNSTLPSQDSECILYVMEMKLVKPVTDEGNPKRRKIIDPFESNSAFGFLSSKELPKIPGFPVFQRNGEMNVQIRKVKNQQFRPTFELLQLIYLFHQHIFEDILRVARGGVVFAPGHSPIPLLIVPLKKLSNADLDYEIDKDYLNWDIRESPTTPSDEIRKQFSFEESNYLNAVVSPWYRSEDQSAFYYVAEIMTDQFPSSSFPDEKFTCFNQYFMSKYQLEIYNQKQNLLDVDHTSARMNLLLPRAITGKSATRSLDPSQRQILVPELVHIHPLSATLWSIIVTLPTILYRLNSLLLADEFRSKVLEDALKINSQTPDDFEWSPLQYVTLNDDLTQKSIRNLDQLRKMNKQEKENEVPMECENIEENVNETAASGIVGELKNFRSNDFEIGVWDPSDGAQMSNNDNTLNEPSPVIHNAPVNGLIPGRRNGLRGVIAARDEELSEIIAVGNDTTLHNYGDISDDDDVAAEYDKFKFLMDNKMTTSDIGDLGEMDVRPAGWNDDSNANVIQIENESLPLTISTNNPHINIASLMNDLEKNYAAFGTSSHISNNTRTNTPIISSDTAVDKTALQRKEVLLTYLTNNSVKRQNEDILPEEVFWGMDEAEIGENISSEALTTSSAADGINLERLETIGDSFLKMAVTNYFYYKHTEQHEGKLSYARSKEVSNSHLFYLGRQRGIPLLIETLKFDPHVNWLPPCYASTSEFHAVNPFDYTDLDEDQCQVPMEGVDTTETLDQQQKTNKETIATGWGTLDDDKQNYKRENGVETLTFPQPTKSEIPDLPPMPYNMLTQQWISDKSIADAVEALIGAHLIQLGQSATLKFMNWLGIKVLTDISSLPSPLLRFIDTPEDPNLSLKHLALLYEKFDFATVENNIGYKFANKAYLVQAFTHASYYNNRVTGCYQRLEFLGDAVLDYMITRFLYEHKKQYSPGVLTDLRSALVNNTIFASLAVKYSFHKHFVMICPPLYQMVEKFVNFCKQKDFLHCANFDDEIFMLTEDEIDEEDLLSEEDVEVPKAMGDIFESIAGAVYLDCGMDLDIVWRVFYNLMRDVIQKCCENPPQSPVRELFERKNCRAKFSKLERKLETGKVRVTVTVNDNLQFTGMGRSYRIAKCTAAKRALQHLRKLDATKTAKNK

>FXSY01000995- *M. incognita* (1810aa)

MSPPKDFSGKCISPRDYQVELLDRAKIQNTIISLGTGSGKTFVAVLLIKEYSQRLLHQNEKAVFLVNTVELVAQQAEHIEFHSSLSVARISGSTIKRKYERKEVEKITSSNQASHVLLKSSLFFVLKVIVITAQVFLDLINHGLFDFSSLALLIVDECHHCLGELHPYRLIMNHYKKLQGQRPRVLGLTASILNKKVPSSRIECTAQLLEQIMDSQIETSSNYTQERLRSFIEKNEDFHSELEVDPRRKIFETISRSFSTLQQVGSWAALKGFILWQKNLLKHVDDPIIGNKQKCILRMAETAFRTCSKVLSHKINPLNSYDKLVNVNKSFISDRVRKLLEILKSYSPSKRELSGIKDTLFGLVFVKERFIAFMINNLLRFLVKQNPEEFGHLKVDFIVGHTGNSETGDEDRRLVNRKQEQTLTKFRNGQLNLLITTNVLEEGIDLRNCNLVVRFDPPMDFRSFIQSSGRARRENSAFYILIEEKNYLEFMMDLTGYAQAEEFVLRRYRSGNDFTLEGNDETKLLHPHLDDAVAPYVVTTEKGTAKVSLSNAIQYCAKLPSDIFTRLVPRYTVQTLSENGQTLYIAELYLPINSPIKEPIKSKPMTSKRLSLMAVALEACKRLHQRKELNDNLLPAGKDILDDLLGEVDDDEYLPHLPSRMGSSKKKRLYDRKMSKTLNSTLPSQDSECVLYVMEMKLIKPVTDEGNPKRRKIIDPFESNSAFGFLSSKELPKVPGFPVFQRNGEMNVQIRKVKNQQFRPTFELLQLIYLFHQHIFEDILRVARGGVVFAPGHSPIPLLIVPLKKLSTADLDYEIDRDYLNWNIRESPTTPSDEIRKQFSFEESNYLNAVVSPWYRSEDQSAFYYVAEIMTDQFPSSSFPDEKFACFNQYFMSKYQLEIYNQKQNLLDVDHTSARMNLLLPRAISGKSATRSLDPSQRQILVPELVHIHPLSATLWSIIVTLPTILYRLNSLLLADEFRSKVLEDALKINSQTPDDFEWSPLQYVTLNDDLTQKSIRNLDQLRKMNKQEKENEVPMECDIIEENVNETAASGIVGRLKFLKSNDFEIGVWDPSDGAQMSNNDNTPNEPSPVIHNAPVNGLIPGRRNGLRGVIAARDEELSEIIAVGNDTTLHNYGDISDDDDVAAEYDKFKFLMDNKMTTSDIGDLGEMDVRPAGWNDDSNANVIQIENESLPLTISTNNPHINIASLMNDLEKNYAAFGTSNHISNNNKTNTPNIPNATADKTDLQKKEVLLTYLINNSVKRQNEDILPEEVFWGMDEAEIGENISSEALTTSSAADGINLERLETIGDSFLKMAVTNYFYYKHTEQHEGKLSYARSKEVSNSHLFYLGRQRGIPLLIETLKFDPHVNWLPPCYASTSEFHAVNPFDYTDLDEDQCAVPMEGVDTTETVDQQQKINKETIATGWGTIDDDKQNYKRENGVETLTFPQPTKSEIPDLPPMPYNMLTQQWISDKSIADAVEALIGAHLIQLGQSATLKFMNWLGIKVLTDISSLPSPLLRFIDTPEDPNLSLKHLALLYEKFDFATVENNIGYKFANKAYLVQAFTHASYYNNRVTGCYQRLEFLGDAVLDYMITRFLYEHKKQYSPGVLTDLRSALVNNTIFASLAVKYSFHKHFVMICPPLYQMVEKFVNFCKQKDFLHCANFDDEIFMLTEDEIDEEDLLSEEDVEVPKAMGDIFESIAGAVYLDCGMDLDIVWRVFYNLMRDVIQKCCENPPQSPVRELFERKNCRAKFSKLERKLETGKVRVTVTVNDNLQFTGMGRSYRIAKCTAAKRALQHLRKLDATKTAKNK

>RCFL01003232- *M. incognita* (1810aa)

MSPPKDFSGKCISPRDYQVELLDRAKIQNTIISLGTGSGKTFVAVLLIKEYSQRLLHQNEKAVFLVNTVELVAQQAEHIEFHSSLSVARISGSTIKRKYERKEVEKITSSNQASHVLLKSSLFFVLKVIVITAQVFLDLINHGLFDFSSLALLIVDECHHCLGELHPYRLIMNHYKKLQGQRPRVLGLTASILNKKVPSSRIECTAQLLEQIMDSQIETSSNYTQERLRSFIEKNEDFHSELEVDPRRKIFETISRSFSTLQQVGSWAALKGFILWQKNLLKHVDDPIIGNKQKCILRMAETAFRTCSKVLSHKINPLNSYDKLVNVNKSFISDRVRKLLEILKSYSPSKRELSGIKDTLFGLVFVKERFIAFMINNLLRFLVKQNPEEFGHLKVDFIVGHTGNSETGDEDRRLVNRKQEQTLTKFRNGQLNLLITTNVLEEGIDLRNCNLVVRFDPPMDFRSFIQSSGRARRENSAFYILIEEKNYLEFMMDLTGYAQAEEFVLRRYRSGNDFTLEGNDETKLLHPHLDDAVAPYVVTTEKGTAKVSLSNAIQYCAKLPSDIFTRLVPRYTVQTLSENGQTLYIAELYLPINSPIKEPIKSKPMTSKRLSLMAVALEACKRLHQRKELNDNLLPAGKDILDDLLGEVDDDEYLPHLPSRMGSSKKKRLYDRKMSKTLNSTLPSQDSECVLYVMEMKLIKPVTDEGNPKRRKIIDPFESNSAFGFLSSKELPKVPGFPVFQRNGEMNVQIRKVKNQQFRPTFELLQLIYLFHQHIFEDILRVARGGVVFAPGHSPIPLLIVPLKKLSTADLDYEIDRDYLNWNIRESPTTPSDEIRKQFSFEESNYLNAVVSPWYRSEDQSAFYYVAEIMTDQFPSSSFPDEKFACFNQYFMSKYQLEIYNQKQNLLDVDHTSARMNLLLPRAISGKSATRSLDPSQRQILVPELVHIHPLSATLWSIIVTLPTILYRLNSLLLADEFRSKVLEDALKINSQTPDDFEWSPLQYVTLNDDLTQKSIRNLDQLRKMNKQEKENEVPMECDIIEENVNETAASGIVGRLKFLKSNDFEIGVWDPSDGAQMSNNDNTPNEPSPVIHNAPVNGLIPGRRNGLRGVIAARDEELSEIIAVGNDTTLHNYGDISDDDDVAAEYDKFKFLMDNKMTTSDIGDLGEMDVRPAGWNDDSNANVIQIENESLPLTISTNNPHINIASLMNDLEKNYAAFGTSNHISNNNKTNTPNIPNATADKTDLQKKEVLLTYLINNSVKRQNEDILPEEVFWGMDEAEIGENISSEALTTSSAADGINLERLETIGDSFLKMAVTNYFYYKHTEQHEGKLSYARSKEVSNSHLFYLGRQRGIPLLIETLKFDPHVNWLPPCYASTSEFHAVNPFDYTDLDEDQCAVPMEGVDTTETVDQQQKINKETIATGWGTIDDDKQNYKRENGVETLTFPQPTKSEIPDLPPMPYNMLTQQWISDKSIADAVEALIGAHLIQLGQSATLKFMNWLGIKVLTDISSLPSPLLRFIDTPEDPNLSLKHLALLYEKFDFATVENNIGYKFANKAYLVQAFTHASYYNNRVTGCYQRLEFLGDAVLDYMITRFLYEHKKQYSPGVLTDLRSALVNNTIFASLAVKYSFHKHFVMICPPLYQMVEKFVNFCKQKDFLHCANFDDEIFMLTEDEIDEEDLLSEEDVEVPKAMGDIFESIAGAVYLDCGMDLDIVWRVFYNLMRDVIQKCCENPPQSPVRELFERKNCRAKFSKLERKLETGKVRVTVTVNDNLQFTGMGRSYRIAKCTAAKRALQHLRKLDATKTAKNK

>RCFL01003234- *M. incognita* (1787aa)

MSPPRDFSGKCIPPRDYQFKFKVELLDRAKIQNTIISLGTGSGKTFVAVLLIKEYSQRLLHQNEKAVFLVNTVELVAQQAEHIEFHSSLSVARISGSTIKRKYERKEVEKITNTNQASHLFDFSSLALLIVDECHHCLGELHPYRLIMNHYKKLQGQRPRVLGLTASILNKKVPSSRIECTAQLLEQIMDSQIETSSNYTQERLRSFIEKNEDFHSELEVDPRRKIFETISRSFSTLQQVGSWAALKGFILWQKNLLKHVDDPIIGNKQKCILRMAETAFRTCSKVLSHKINPLNSYDKLSNVNKSFISDRVRKLLEILKSYSPSKRELSGIKDTLFGLVFVKERFIAFMINNLLRFLVKQNPEEFGHLKVDFIVGHTGNSETGDEDRRLVNRKQEQTLTKFRNGQLNLLITTNVLEEGIDLRNCNLVVRFDPPMDFRSFIQSSGRARRENSAFYILIEEKNYLDFMMDLTGYAQAEEFVLRRYRSGNDFTLEGNDETKILHPHLDDAVAPYVVTTEKGTAKVSLSNAIQYCAKLPSDIFTRLVPRYTIQTLSENGQTLYIAELYLPINSPIKEPIKSKPMNSKRLSLMAVALEACKRLHQRKELNDNLLPAGKDILDDLLGEVDDDEYLPHLPSRMGSSKKKRLYDRKMSKTLNSTLPSQDSECILYVMEMKLVKPVTDEGNPKRRKIIDPFESNSAFGFLSSKELPKIPGFPVFQRNGEMNVQIRKVKNQQFRPTFELLQLIYLFHQHIFEDILRVARGGVVFAPGHSPIPLLIVPLKKLSNADLDYEIDKDYLNWDIRESPTTPSDEIRKQFSFEESNYLNAVVSPWYRSEDQSAFYYVAEIMTDQFPSSSFPDEKFTCFNQYFMSKYQLEIYNQKQNLLDVDHTSARMNLLLPRAITGKSATRSLDPSQRQILVPELVHIHPLSATLWSIIVTLPTILYRLNSLLLADEFRSKVLEDALKINSQTPDDFEWSPLQYVTLNDDLTQKSIRNLDQLRKMNKQEKENEVPMECENIEENVNETAASGIVGELKNFRSNDFEIGVWDPSDGAQMSNNDNTLNEPSPVIHNAPVNGLIPGRRNGLRGVIAARDEELSEIIAVGNDTTLHNYGDISDDDDVAAEYDKFKFLMDNKMTTSDIGDLGEMDVRPAGWNDDSNANVIQIENESLPLTISTNNPHINIASLMNDLEKNYAAFGTSSHISNNTRTNTPIISSDTAVDKTALQRKEVLLTYLTNNSVKRQNEDILPEEVFWGMDEAEIGENISSEALTTSSAADGINLERLETIGDSFLKMAVTNYFYYKHTEQHEGKLSYARSKEVSNSHLFYLGRQRGIPLLIETLKFDPHVNWLPPCYASTSEFHAVNPFDYTDLDEDQCQVPMEGVDTTETLDQQQKTNKETIATGWGTLDDDKQNYKRENGVETLTFPQPTKSEIPDLPPMPYNMLTQQWISDKSIADAVEALIGAHLIQLGQSATLKFMNWLGIKVLTDISSLPSPLLRFIDTPEDPNLSLKHLALLYEKFDFATVENNIGYKFANKAYLVQAFTHASYYNNRVTGCYQRLEFLGDAVLDYMITRFLYEHKKQYSPGVLTDLRSALVNNTIFASLAVKYSFHKHFVMICPPLYQMVEKFVNFCKQKDFLHCANFDDEIFMLTEDEIDEEDLLSEEDVEVPKAMGDIFESIAGAVYLDCGMDLDIVWRVFYNLMRDVIQKCCENPPQSPVRELFERKNCRAKFSKLERKLETGKVRVTVTVNDNLQFTGMGRSYRIAKCTAAKRALQHLRKLDATKTAKNK

>RCFM01005457-*M. enterolobii* (1787aa)

MSPPKDFSAKCIPPRDYQVELLDRAKIQNTIISLGTGSGKTFVAVLLIKEYSQRLLHQNEKAVFLVNTVELVAQQAEHIEFHSSLSVARISGSTIKRKYERREVEKITSNNQASHLFDFSSLALLIVDECHHCLGELHPYRLIMNHYKKLQGQRPRVLGLTASILNKKVPSSRIECTAQLLEQIMDSQIETSSNYTQERLRSFIEKNEDFHSEFEVDPRRKIFETISRSFSTLQQVGSWAALKGFILWQKNLLKHVDDPIIGNKQKCILRMAETAFRTCSKVLSHKINPLNSYDKLGNVNKSYISDRVRKLLEILKSYSPSKRELSGIKDTLFGLVFVKERFIAFMINNLLRFLVKQNPEEFGHLKVDFIVGHTGNSETGDEDRRLVNRKQERTLTQFRNGQLNLLITTNVLEEGIDLRNCNLVIRFDPPMDFRSFIQSSGRARRENSAFYILIEEKNYLDFMMDLTGYAQAEEFVLRRYRSGNDFTLEGNDETKMIQPHLDDAVAPYVVTTEKGTAKVSLSNAIQLVNRYCAKLPSDIFTRLVPRYTIQTLSENGQTLYIAELYLPINSPIKEPIKSKPMNSKRLSLMAVALEACKRLHQRKELNDNLLPAGKDILDDLLGEVDDDEYLPHLPSRMGSSKKKRLYDRKMSKTLNSTLPSQDSQCILYVMEMKLVKPVTDEGNPKRRKIIDPFESNSAFGFLSSKELPKVPGFPVFQRNGEMLVQIRKVKNQQFRPTFELLQLIYLFHQHIFEDILRVARGGVVFAPGHSPIPLLIVPLKKLSTADLDYEIDKDYLNWNIRESPTTPSDEIRRQFSFEESNYLNAVVSPWYRSEDQSAFYYVAEIMTDQFPSSSFPDEKFTCFNQYFMSKYQLEIYNQKQNLLDVDHTSARMNLLLPRAITGKSATRSLDPSQRQILVPELVHIHPLSATLWSIIVTLPTILYRLNSLLLADEFRSKVLEDALKVNSQTPDDFEWSPLQYVTLNDDLTQKSIKNLDQLRKMNKQEKENEVPMELDIIDENVNETAATGMVGELKILRSNDFEIGVWDPSDGTQMSNNDNTLNEPSPVIHNAPVNGLIPGRRNGLRGVIAARDEELSEIIAVGNDTTLHNYGDISDDDDVAAEYDKFKFLMDNKMTTSDIGDLGEMDVRPAGWNDDSNANVIQIENESLPLTISTNNPHINIASLMNDLEKNYAAFGTSSHISNNNKTNTSIIPSATAVDKTVLQRKEVLLTYLINNSVKRQNEDILPEEVFWGMDEAEIGENISSEALTTSSAADGINLERLETIGDSFLKMAVTNYFYYKHTEQHEGKLSYARSKEVSNSHLFYLGRQRGIPLLIETLKFDPHVNWLPPCYASTSEFHAVNPFDYTDLDEDQCQVPMEGVGTTETVDQQQKINKETIATGWGTLDDDKQNYKRENGVETLTFPQPTKSEIPDLPPMPYNMLTQQWISDKSIADAVEALIGAHLIQLGQSATLKFMNWLGIKVLTDISSLPSPLLRFIDTPEDPNLSLKHLALLYEKFDFATVENNIGYKFANKAYLVQAFTHASYYNNRVTGCYQRLEFLGDAVLDYMITRFLYEHKKQYSPGVLTDLRSALVNNTIFASLAVKYSFHKHFVMICPPLYQMVEKFVNFCKQKDFLHCANFDDEIFMLTEDEIDEEDLLSEEDVEVPKAMGDIFESIAGAVYLDCGMNLDIVWRVFYNLMRDVIQKCCENPPQSPVRELFERKNCRAKFSKLERKLETGKVRVTVTVNDNLQFTGMGRSYRIAKCTAAKRALQHLRKLDAAKTAKNK

>RCFM01006500- *M. enterolobii* (1800aa)

MSPPKDFSAKCIPPRDYQVELLDRAKIQNTIISLGTGSGKTFVAVLLIKEYSQRLLHQNEKAVFLVNTVELVAQQAEHIEFHSSLSVARISGSTIKRKYERKEVEKITSSSQVIVITAQVFLDLINHGLFDFSSLALLIVDECHHCLGELHPYRLIMNHYKKLQGQRPRVLGLTASILNKKVPSSRIECTAQLLEQIMDSQIETSSNYTQERLRSFIEKNEDFHSELEVDPRRKIYETISRSFSTLQQVGSWAALKGFILWQKNLLKHVDDPIIGNKQKCILRMAETAFRTCSKVLSHKINPLNSYDKLGNVNKSYISDRVRKLLEILKSYSPSKRELSGIKDTLFGLVFVKERFIAFMINNLLRFLVKQNPEEFGHLKVDFIVGHTGNSETGDEDRRLVNRKQERTLTQFRNGQLNLLITTNVLEEGIDLRNCNLVIRFDPPMDFRSFIQSSGRARRENSAFYILIEEKNYLDFMMDLTGYAQAEEFVLRRYRSGNDFTLEGNDETKMIHPHLDDAVAPYVVTTEKGTAKVSLSNAIQLVNRYCAKLPSDIFTRLVPRYTIQTLSENGQTLYIAELYLPINSPIKEPIKSKPMTSKRLALMAVALEACKRLHQRKELNDNLLPAGKDILDDLLGEVDDDEYLPHLPSRMGSSKKKRLYDRKMSKTLNSTLPSQNSECILYVMEMKLVKPVTDEGNPKRRKIIDPFESNSAFGFLSSKELPKVPGFPVFQRNGEMLVQIRKVKNQQFRPTFELLQLIYLFHQHIFEDILRVARGGVVFAPGHSPIPLLIVPLKKLSTADLDYEIDKDYLNWNIRESPTTPSDEIRKQFSFEESNYLNSVVSPWYRSEDQSAFYYVAEIMTDQFPSSSFPDEKFTCFNQYFMSKYQLEIYNQKQNLLDVDHTSARMNLLLPRAITGKSATRSLDPSQRQILVPELVHIHPLSATLWSIIVTLPTILYRLNSLLLADEFRSKVLEDALKFNSQTPDDFEWSPLQYVTLNDDLTQKSIKNLDQLRKMNKQEKENEVPMELDIIGENVNETAASGIVGELKILKSNDFEIGVWDPSDGAQMSNNDNTSNEPSPVIHNAPVNGLIPGRRNGLRGVIAARDEELSEIIAVGNDTTLHNYGDISDDDDVAAEYDKFKFLMDNKMTTSDIGDLGEMDVRPAGWNDDSNANVIQIENESLPLTISTNNPHINIASLMNDLEKNYAAFGTSSHISNNNKANTPIIPSATAVDKTVLQRKEVLLTYLINNSVKRQNEDILPEEVFWGMDEAQIGENISSEALTTSSAADGINLERLETIGDSFLKMAVTNYFYYKHTEQHEGKLSYARSKEVSNSHLFYLGRQRGIPLLIETLKFDPHVNWLPPCYASTSEFHAVNPFDYTDLDEDQREVPMEGVDTNETVDQQQKINKEAITTGWGTLDDDKQNYKRENGVETLTFPQPTKSEIPDLPPMPYNMLTQQWISDKSIADAVEALIGAHLIQLGQSATLKFMNWLGIKVLTDISSLPSPLLRFIDTPEDPNLSLKHLALLYEKFDFATVENNIGYKFANKAYLVQAFTHASYYNNRVTGCYQRLEFLGDAVLDYMITRFLYEHKKQYSPGVLTDLRSALVNNTIFASLAVKYSFHKHFVMICPPLYQMVEKFVNFCKQKDFLHCANFDDEIFMLTEDEIDEEDLLSEEDVEVPKAMGDIFESIAGAVYLDCGMDLDIVWRVFYNLMRDVIQKCCENPPQSPVRELFERKNCRAKFSKLERKLETGKVRVTVTVNDNLQFTGMGRSYRIAKCTAAKRALQHLRKLDATKSAKNK

>CEWN01000587-*M. javanica* (1814aa)

MSPPKDFSGKCIPPRDYQFKFKVELLDRAKIQNTIISLGTGSGKTFVAVLLIKEYSQRLLHQNEKAVFLVNTVELVAQQAEHIEFHSSLSVARISGSTIKRKYERKEVEKITSSNQASHVLLKFSLFFVLKVIVITAQVFLDLINHGLFDFSSLALLIVDECHHCLGELHPYRLIMNHYKKLQGQRPRVLGLTASILNKKVPSSRIECTAQLLEQIMDSQIETSSNYTQERLRSFIEKNEDFHSELEVDPRRKIFETISRSFSTLQQVGSWAALKGFILWQKNLLKHVDDPIIGNKQKCILRMAETAFRTCSKVLSHKINPLNSYDKLVNVNKSFISDRVRKLLEILKSYSPSKRELSGIKDTLFGLVFVKERFIAFMINNLLRFLVKQNPEEFGHLKVDFIVGHTGNSETGDEDRRLVNRKQEQTLTKFRNGQLNLLITTNVLEEGIDLRNCNLVVRFDPPMDFRSFIQSSGRARRENSAFYILIEEKNYLEFMMDLTGYAQAEEFVLRRYRSGNDFTLEGNDETKLLHPHLDDAVAPYVVTTEKGTAKVSLSNAIQYCAKLPSDIFTRLVPRYTIQTLSENGQTLYIAELYLPINSPIKEPIKSKPMTSKRLSLMAVALEACKRLHQRKELNDNLLPAGKDILDDLLGEVDDDEYLPHLPSRMGSSKKKRLYDRKMSKTLNSTLPSQDSECVLYVMEMKLIKPVTDEGNPKRRKIIDPFESNSAFGFLSSKELPKVPGFPVFQRNGEMNVQIRKVKNQQFRPTFELLQLIYLFHQHIFEDILRVARGGVVFAPGHSPIPLLIVPLKKLSTADLDYEIDRDYLNWNIRESPTTPSDEIRKQFSFEESNYLNAVVSPWYRSEDQSAFYYVAEIMTDQFPSSSFPDEKFACFNQYFMSKYQLEIYNQKQNLLDVDHTSARMNLLLPRAISGKSATRSLDPSQRQILVPELVHIHPLSATLWSIIVTLPTILYRLNSLLLADEFRSKVLEDALKINSQTPDDFEWSPLQYVTLNDDLTQKSIRNLDQLRKMNKQEKENEVPMECDIIEENVNETAASGIVGRLKFLKSNDFEIGVWDPSDGAQMSNNDNTPNEPSPVIHNAPVNGLIPGRRNGLRGVIAARDEELSEIIAVGNDTTLHNYGDISDDDDVAAEYDKFKFLMDNKMTTSDIGDLGEMDVRPAGWNDDSNANVIQIENESLPLTISTNNPHINIASLMNDLEKNYAAFGTSNHISNNNKTNTPNIPNATADKTVLQKKEVLLTYLINNSVKRQNEDILPEEVFWGMDEAEIGENISSEALTTSSAADGINLERLETIGDSFLKMAVTNYFYYKHTEQHEGKLSYARSKEVSNSHLFYLGRQRGIPLLIETLKFDPHVNWLPPCYASTSEFHAVNPFDYTDLDEDQCAVPMEGVDTTETVDQQQKINKETIATGWGTIDDDKQNYKRENGVETLTFPQPTKSEIPDLPPMPYNMLTQQWISDKSIADAVEALIGAHLIQLGQSATLKFMNWLGIKVLTDISSLPSPLLRFIDTPEDPNLSLKHLALLYEKFDFATVENNIGYKFANKAYLVQAFTHASYYNNRVTGCYQRLEFLGDAVLDYMITRFLYEHKKQYSPGVLTDLRSALVNNTIFASLAVKYSFHKHFVMICPPLYQMVEKFVNFCKQKDFLHCANFDDEIFMLTEDEIDEEDLLSEEDVEVPKAMGDIFESIAGAVYLDCGMDLDIVWRVFYNLMRDVIQKCCENPPQSPVRELFERKNCRAKFSKLERKLETGKVRVTVTVNDNLQFTGMGRSYRIAKCTAAKRALQHLRKLDATKTAKNK

>RCFK01001654- *M. javanica* (1815aa)

MSPPKDFSGKCIPPRDYQFKFKVELLDRAKIQNTIISLGTGSGKTFVAVLLIKEYSQRLLHQNEKAVFLVNTVELVAQQAEHIEFHSSLSVARISGSTIKRKYERKEVEKITSSNQASHVLLKFSLFFVLKVIVITAQVFLDLINHGLFDFSSLALLIVDECHHCLGELHPYRLIMNHYKKLQGQRPRVLGLTASILNKKVPSSRIECTAQLLEQIMDSQIETSSNYTQERLRSFIEKNEDFHSELEVDPRRKIFETISRSFSTLQQVGSWAALKGFILWQKNLLKHVDDPIIGNKQKCILRMAETAFRTCSKVLSHKINPLNSYDKLSNVNKSFISDRVRKLLEILKSYSPSKRELSGIKDTLFGLVFVKERFIAFMINNLLRFLVKQNPEEFGHLKVDFIVGHTGNSETGDEDRRLVNRKQEQTLTKFRNGQLNLLITTNVLEEGIDLRNCNLVVRFDPPMDFRSFIQSSGRARRENSAFYILIEEKNYLDFMMDLTGYAQAEEFVLRRYRSGNDFTLEGTDETKIIHPHLDDAVAPYVVTTEKGTAKVSLSNAIQYCAKLPSDIFTRLVPRYTIQTLSENGQTLYIAELYLPINSPIKEPIKSKPMNSKRLSLMAVALEACKRLHQRKELNDNLLPAGKDILDDLLGEVDDDEYLPHLPSRMGSSKKKRLYDRKMSKTLNSTLPSQDSACFLYVMEMKLIKPVTDEGNPKRRKIIDPFESNSAFGFLSSKELPKVPGFPVFQRNGEMNVQIRKVKNQQFRPNFELLQLIYLFHQHIFEDILRVARGGVVFAPGHSPIPLLIVPLKKLSTADLDYEIDKDYLNWDIRESPTTPSDEIRKQFSFEESNYLNAVVSPWYRSEDQSAFYYVAEIMTDQFPSSSFPDEKFTCFNQYFMSKYQLEIYNQKQNLLDVDHTSARMNLLLPRAITGKSATRSLDPSQRQILVPELVHIHPLSATLWSIIVTLPTILYRLNSLLLADEFRSKVLEDALKINSQTPDDFEWSPLQYVTLNDDLTQKSIRNLDQLRKMNKQEKENEVPMECDIIEENVNETAASGIVGKLKYLKSNDFEIGVWDPSDGAQMSNNDNTLNEPSPVIHNAPVNGIIPGRRNGLRGVIAARDEELSEIIAVGNDTTLHNYGDISDDDDVAAEYDKFKFLMDNKMTTSDIGDLGEMDVRPAGWNDDSNANVIQIENESLPLTISTNNPHINIASLMNDLEKNYAAFGTSSHISNNTRTNTPIISSDTAVDKTALQRKEVLLTYLTNNSVKRQNEDILPEEVFWGMDEAEIGENISSEALTTSSAADGINLERLETIGDSFLKMAVTNYFYYKHTEQHEGKLSYARSKEVSNSHLFYLGRQRGIPLLIETLKFDPHVNWLPPCYASTSEFHAVNPFDYTDLDEDQCQVPMEGVDTTETLDQQQKTNKETIATGWGTLDDDKQNYKRENGVETLTFPQPTKSEIPDLPPMPYNMLTQQWISDKSIADAVEALIGAHLIQLGQSATLKFMNWLGIKVLTDISSLPSPLLRFIDTPEDPNLSLKHLALLYEKFDFATVENNIGYKFANKAYLVQAFTHASYYNNRVTGCYQRLEFLGDAVLDYMITRFLYEHKKQYSPGVLTDLRSALVNNTIFASLAVKYSFHKHFVMICPPLYQMVEKFVNFCKQKDFLHCANFDDEIFMLTEDEIDEEDLLSEEDVEVPKAMGDIFESIAGAVYLDCGMDLDIVWRVFYNLMRDVIQKCCENPPQSPVRELFERKNCRAKFSKLERKLETGKVRVTVTVNDNLQFTGMGRSYRIAKCTAAKRALQHLRKLDATKTARNK

>RCFK01001432- *M. javanica* (1810aa)

MSPPKDFSGKCIPPRDYQVELLDRAKIQNTIISLGTGSGKTFVAVLLIKEYSQRLLHQNEKAVFLVNTVELVAQQAEHIEFHSSLSVARISGSTIKRKYERKEVEKITSSNQASHVLLKSSLFFVLKVIVITAQVFLDLINHGLFDFSSLALLIVDECHHCLGELHPYRLIMNHYKKLQGQRPRVLGLTASILNKKVPSSRIECTAQLLEQIMDSQIETSSNYTQERLRSFIEKNEDFHSELEVDPRRKIFETISRSFSTLQQVGSWAALKGFILWQKNLLKHVDDPIIGNKQKCILRMAETAFRTCSKVLSHKINPLNSYDKLVNVNKSFISDRVRKLLEILKSYSPSKRELSGIKDTLFGLVFVKERFIAFMINNLLRFLVKQNPEEFGHLKVDFIVGHTGNSETGDEDRRLVNRKQEQTLTKFRNGQLNLLITTNVLEEGIDLRNCNLVVRFDPPMDFRSFIQSSGRARRENSAFYILIEEKNYLEFMMDLTGYAQAEEFVLRRYRSGNDFTLEGNDETKLLHPHLDDAVAPYVVTTEKGTAKVSLSNAIQYCAKLPSDIFTRLVPRYTIQTLSENGQTLYIAELYLPINSPIKEPIKSKPMTSKRLSLMAVALEACKRLHQRKELNDNLLPAGKDILDDLLGEVDDDEYLPHLPSRMGSSKKKRLYDRKMSKTLNSTLPSQDSECVLYVMEMKLIKPVTDEGNPKRRKIIDPFESNSAFGFLSSKELPKVPGFPVFQRNGEMNVQIRKVKNQQFRPTFELLQLIYLFHQHIFEDILRVARGGVVFAPGHSPIPLLIVPLKKLSTADLDYEIDRDYLNWNIRESPTTPSDEIRKQFSFEESNYLNAVVSPWYRSEDQSAFYYVAEIMTDQFPSSSFPDEKFACFNQYFMSKYQLEIYNQKQNLLDVDHTSARMNLLLPRAISGKSATRSLDPSQRQILVPELVHIHPLSATLWSIIVTLPTILYRLNSLLLADEFRSKVLEDALKINSQTPDDFEWSPLQYVTLNDDLTQKSIRNLDQLRKMNKQEKENEVPMECDIIEENVNETAASGIVGRLKFLKSNDFEIGVWDPSDGAQMSNNDNTPNEPSPVIHNAPVNGLIPGRRNGLRGVIAARDEELSEIIAVGNDTTLHNYGDISDDDDVAAEYDKFKFLMDNKMTTSDIGDLGEMDVRPAGWNDDSNANVIQIENESLPLTISTNNPHINIASLMNDLEKNYAAFGTSNHISNNNKTNTPNIPNATADKTDLQKKEVLLTYLINNSVKRQNEDILPEEVFWGMDEAEIGENISSEALTTSSAADGINLERLETIGDSFLKMAVTNYFYYKHTEQHEGKLSYARSKEVSNSHLFYLGRQRGIPLLIETLKFDPHVNWLPPCYASTSEFHAVNPFDYTDLDEDQCAVPMEGVDTTETVDQQQKINKETIATGWGTIDDDKQNYKRENGVETLTFPQPTKSEIPDLPPMPYNMLTQQWISDKSIADAVEALIGAHLIQLGQSATLKFMNWLGIKVLTDISSLPSPLLRFIDTPEDPNLSLKHLALLYEKFDFATVENNIGYKFANKAYLVQAFTHASYYNNRVTGCYQRLEFLGDAVLDYMITRFLYEHKKQYSPGVLTDLRSALVNNTIFASLAVKYSFHKHFVMICPPLYQMVEKFVNFCKQKDFLHCANFDDEIFMLTEDEIDEEDLLSEEDVEVPKAMGDIFESIAGAVYLDCGMDLDIVWRVFYNLMRDVIQKCCENPPQSPVRELFERKNCRAKFSKLERKLETGKVRVTVTVNDNLQFTGMGRSYRIAKCTAAKRALQHLRKLDATKTAKNK

>RCFN01006666-*M. floridensis* (1802aa)

MSPPKDFSGKCIPPRDYQFKFKVELLDRAKIQNTIISLGTGSGKTFVAVLLIKEYSQRLLHQNEKAVFLVNTVELVAQQAEHIEFHSSLSVARISGSTIKRKYERKEVEKITNTNQASHVIVITAQVFLDLINHGLFDFSSLALLIVDECHHCLGELHPYRLIMNHYKKLQGQRPRVLGLTASILNKKVPSSRIECTAQLLEQIMDSQIETSSNYTQERLRSFIEKNEDFHSELEVDPRRKIFETISRSFSTLQQVGSWAALKGFILWQKNLLKHVDDPIIGNKQKCILRMAETAFRTCSKVLSHKINPLNSYDKLSNVNKSFISDRVRKLLEILKSYSPSKRELSGIKDTLFGLVFVKERFIAFMINNLLRFLVKQNPEEFGHLKVDFIVGHTGNSETGDEDRRLVNRKQEQTLTKFRNGQLNLLITTNVLEEGIDLRNCNLVVRFDPPMDFRSFIQSSGRARRENSAFYILIEEKNYLDFMMDLTGYAQAEEFVLRRYRSGNDFTLEGNDETKILHPHLDDAVAPYVVTTEKGTAKVSLSNAIQYCAKLPSDIFTRLVPRYTIQTLSENGQTLYIAELYLPINSPIKEPIKSKPMNSKRLSLMAVALEACKRLHQRKELNDNLLPAGKDILDDLLGEVDDDEYLPHLPSRMGSSKKKRLYDRKMSKTLNSTLPSQDSECILYVMEMKLVKPVTDEGNPKRRKIIDPFESNSAFGFLSSKELPKIPGFPVFQRNGEMNVQIRKVKNQQFRPTFELLQLIYLFHQHIFEDILRVARGGVVFAPGHSPIPLLIVPLKKLSNADLDYEIDKDYLNWDIRESPTTPSDEIRKQFSFEESNYLNAVVSPWYRSEDQSAFYYVAEIMTDQFPSSSFPDEKFTCFNQYFMSKYQLEIYNQKQNLLDVDHTSARMNLLLPRAITGKSATRSLDPSQRQILVPELVHIHPLSATLWSIIVTLPTILYRLNSLLLADEFRSKVLEDALKINSQTPDDFEWSPLQYVTLNDDLTQKSIRNLDQLRKMNKQEKENEVPMECENIEENVNETAASGIVGELKYLKSNDFEIGVWDPSDGAQMSSNDNTLNEPSPVIHNAPVNGLIPGRRNGLRGVIAARDEELSEIIAVGNDTTLHNYGDISDDDDVAAEYDKFKFLMDNKMTTSDIGDLGEMDVRPAGWNDDSNANVIQIENESLPLTISTNNPHINIASLMNDLEKNYAAFGTSNHISNNKTNTPIISSDTAVDKTVLQRKEVLLTYLINNSVKRQNEDILPEEVFWGMDEAEIGENISSEALTTSSAADGINLERLETIGDSFLKMAVTNYFYYKHTEQHEGKLSYARSKEVSNSHLFYLGRQRGIPLLIETLKFDPHVNWLPPCYASTSEFHAVNPFDYTDLDEDQCQVPMEGVDTTETLDQQQKINKETIATGWGTLDDDKQNYKRENGVETLTFPQPTKSEIPDLPPMPYNMLTQQWISDKSIADAVEALIGAHLIQLGQSATLKFMNWLGIKVLTDISSLPSPLLRFIDTPEDPNLSLKHLALLYEKFDFATVENNIGYKFANKAYLVQAFTHASYYNNRVTGCYQRLEFLGDAVLDYMITRFLYEHKKQYSPGVLTDLRSALVNNTIFASLAVKYSFHKHFVMICPPLYQMVEKFVNFCKQKDFLHCANFDDEIFMLTEDEIDEEDLLSEEDVEVPKAMGDIFESIAGAVYLDCGMDLDIVWRVFYNLMRDVIQKCCENPPQSPVRELFERKNCRAKFSKLERKLETGKVRVTVTVNDNLQFTGMGRSYRIAKCTAAKRALQHLRKLDATKTAKNK

>ABLG01001138-*M. hapla* (1770aa)

MSPPKDFSGKCIPPRDYQVELLDRAKIQNTIISLGTGAGKTFVAVLLIKEYAQRLLHRNEKAAFLVNTVELVAQQAEHIEFHSSLSVARISGSTIKRKHERGEVEKITRNNQASIVIVITAQVFLDFINHGLFDFSSLAVLIVDECHHCLGELHPYRLIMNHYRKLPGQRPRVLGLTASILNKKVPFSRIECTAQLLEQIMDSHIETTSNYTQERLRSFIEKNEDFHSELEVDPRRPIFEKKLLKLVDDPILGTKQKCILRMAETAFRTCSKVLSHKINPLNTYDKLANPKKSYLSDRMRKLVEILKSYAPSKREKSGIKDTLFGLIFVKERFIAFMINNLLRFLVKQNPEDFGHLKVDFLVGQTGNSETGDEDRRLAARKQEQTLCRFRNGQLNLLVTTNVLEEGIDLRNCNLVIRFDPPMDFRSFVQSSGRARKENSAFYMLIEEKNYLEFMMDLTGYAQAEELVLRRYRSGNDFTLNGKEEGNNETKIIQPHLDDVVAPYVVITPNGTAKVSLSGAIHLVNRYCSKLPSDIFTRLVPRYTIKTISENGQTLYIAELYLPINSPIKEPITSKPMASKRLSLMTAALEACKRLHQRKELNDQLLPAGKEIVLDLLGEVDDDEYLPYLPSKMGSSKKKRLYDRKMSKTLSSTLPAQESECILYVMEMKLIKPVSEERNPKRRKIIDPFESNSAFGFLSSKELPKVPGFPVFQRNGEMIVQIRKAKNQPVRLTFELFQLICLFHQHIFEDILRIARGGVVFAPGHSPIPLLIVPLKKLGTVDLDYEIDRDYLNWDIREPPTTPSDEIRKQYVFEESSYLNAVVSPWYRSEDQSAFYYVAEIMTDQFPSSSFPDEKFTCFNQYFMSKYQLEIYNQKQNLLDVDHTSARMNLLLPRAITGKSALRSLDPSQRQILVPELLHIHPLSATLWSIIVTLPTILYRLNSLLLADEFRSKVLEDALKLGSQTPSDFEWTPLQYVTPNDDQNQKSIRNLDQLRKINQQEKENEVAMECDTVEEKGNETAASGINDFEIGVWDPTPNIDNILDEPPPVIHNAPVNGLIPGRRNGLRGVIAARDEELSEIIAVGNDTTIHNYGDISDDDDVAAEYDKFKFLMHNKMTTSDIGELGEMDVRPAGWNDDSNVNVIQIENESLPLTISTNNPHINIASLMNDLEKNYAAFGTSTSTANNNKVNTPVTPSATTTDKTVIQRKELNLDSLNVIDQNDPSIKRQNEDILPEEVFWGMDEMEENINNEALTTSSAADGINLERLETIGDSFLKMAVTNYFYHKHTEQHEGKLSFARSKEVSNSHLFYLGRQRGIPLLIETLKFDPHVNWLPPCYASTSEFHAVNPFDYTDLDEEQDQREVPMEGVETNKTVDQQQKNNKETIATGWGTLDDDRQNYKCENGVETLTFPQQTKSEIPDLPPMPYNMLTQQWISDKSIADAVEALIGAHLIQLGQSSTLKFMNWLGIKVLTDISSLPSPLLRFIDTPEDPNLSLKHLALFYEKFDFATVENNIGYKFANKAYLVQAFTHASYYNNRVTGCYQRLEFLGDAVLDYMITRFLYEHKRQYSPGVLTDLRSALVNNTIFASLAVKYSFHKHFVMICPPLYQMVEKFVNFCKQKDFLHCANFDDEIFMLTEEEIDEEDLVSEEDVEVPKAMGDIFESIAGAVYLDCGMDLDIVWRVFYNLMRDVIQKCCENPPQSPVRELFERKNCRAKFSKLERKLETGKVRVTVTVNDNLQFTGMGRSYRIAKCTAAKRALQHLRKLDAAKNK

>NXFT01000985-*M. graminicola* (1775aa)

MSPPKDFIGKCIPPRDYQVELLDRAKIQNTIISLGTGAGKTFVAVLLIKEYSQRLLHRNEKAVFLVNTALLTCQQADHIELHSSLSVARVSGSTIKGKYDREKVEKITKENQVIVITAQVFLDFINHGLFDLTSLALLIVDECHHCLGETHPYRLIMNHYMKLQGFQQRPRVLGLTASILNKKVPFSRIEFTAQLLEKIMDSRLETASSYTQDRLRSFVEKNEDFHSELEADPRRPILQAISRSFSTLQQVGGWAALEGFVIWQKNLLKLADDSNIGTKQKCILRMAETTFRTCSKVLSHKIKSLDSYDKLADSKKSFISDRIRKLVEILKMYSPLKREKHGIKDALAGLVFVKERFIASMINKLLRYLAKKYHEDFGYLKVDFIVGNTGSLETGDEDRRLANRKQEQTLRDFKNGQLNLLITTSVLEEGIDLRNCNLVVRFDPPMDFCSYVQSSGRARKENGAFYMLIEEKNYVDFMMDLTKYAQAEELVLRRYRSGNDFILNDKEEMNDKMILQPHIDDVVSPYVVTSANGTAKVSLSGAIQLVNRYCSKLPSDIFTRLVPRYSIQTISEAGQQMYIAELILPINSPIKETITTKPMSSKKLSLMAAALEACKRLHERKELNDQLLPTGKEIVLDLLGEVDDDEYLPYLPSKMGSSKKKRLYDRKMSKTLDSTLPSQETDCFLYIMEMKLVKPVSEERNPKRRKIIDPFESNSAFGFLSSKELPKIPGFPVFQRHGEMLVQFRKAKKPVNLTFELFQLICLFHQHIFEDILRVARGGVVFSPGHSPMPLLIVPLKKVGTVDMDYEIDRDYLNWDIRESSTIPSDEIRKQFVFDESRYLNAVVSPWYRSEDQSAFYYVAEIMSDQFPSSSFPDEKFTCFNQYFMSKYQLEIYNQKQNLLDVDHTSARMNLLLPRSLTGKSTTRSLDPSQRQILIPELVHIHPLSATLWSFIVTLPTILYRLNFLLLADEFRSKVLEDALKLSSQTPDDFEWNPLQYVTSIDGQAEKSIRNLDQLRKMNQLEKENELIMECDNNESKTSEAIQDENEMNDFEIGVWDPSDAAQFSNDANTQNEPTQVIHNAPINGLINGRRNGLRGILAARDEELSEIIAVGNDTTIYNYGDISDDDDVAAEYDKFKFSMHNKMTTSDIGEIGEISHVRPAGWDDNNANVIQIDNENLPLTISTNNPHINIASLMNDLERNYAAFGTSTTNVVNNKDVAMQSTLKKKEYNFDSFDVMDQNDPSIKKQNEEILPEEVFWGIDENEIDENINNEALTTSSAAGGINLERLETIGDSFLKMAVTNYFYHKHTEQHEGKLSFARSKEVSNSHLFYLGRQRGIPLLMETLKFDPHVNWLPPCYASTSEFHAVNPFDYTDLDEEQNQRVLPMEGIETIKNVDQKQKNKETVATGWGTLDDEKQNYKYENGIETLTFPQPTKSEIPDLPPMPYNMLTQQWISDKSIADAVEALIGAHLIQLGQSATLKFMNWLGIKPNRSLRRLALLYEKFDFATVENNIGYKFANKAYLVQAFTHASYYDNRVTGCYQRLEFLGDAVLDYMITRFLYEHKKQYSPGVLTDLRSALVNNTIFASLAVKYSFHKHFIMICPPLYQMIEKFVNFCKQKDFMHCANFDDEIFMLTEEEIDEEELLSEEDVEVPKAMGDVFESMAGAVYLDCGMNLDIVWRVMYNLMRDVIQKCCENPPQSPVRELFERKNCRAKFSKLERKLETGKVRVTVTINENLQFTGMGRSYRIAKCTAAKRALQHLRKLDSAKNVSSTNK

**Supplementary Figure S1**: Multiple alignments of translated amino acid sequences of DCRs of eight *Meloidogyne spp.* **(a)** Sequences of the various DCRs of eight *Meloidogyne* spp. **(b)** Sequences of the Helicase ATP binding domain **(c)** Sequences of the Helicase C-terminal domain **(d)** Sequences of the Dicer Dimerisation domain **(e)** Sequences of the PAZ domain **(f)** Sequences of the Ribonuclease IIIa domain **(g)** Sequences of the Ribonuclease IIIb domain **(h)** Sequences of the double stranded RNA binding motif.


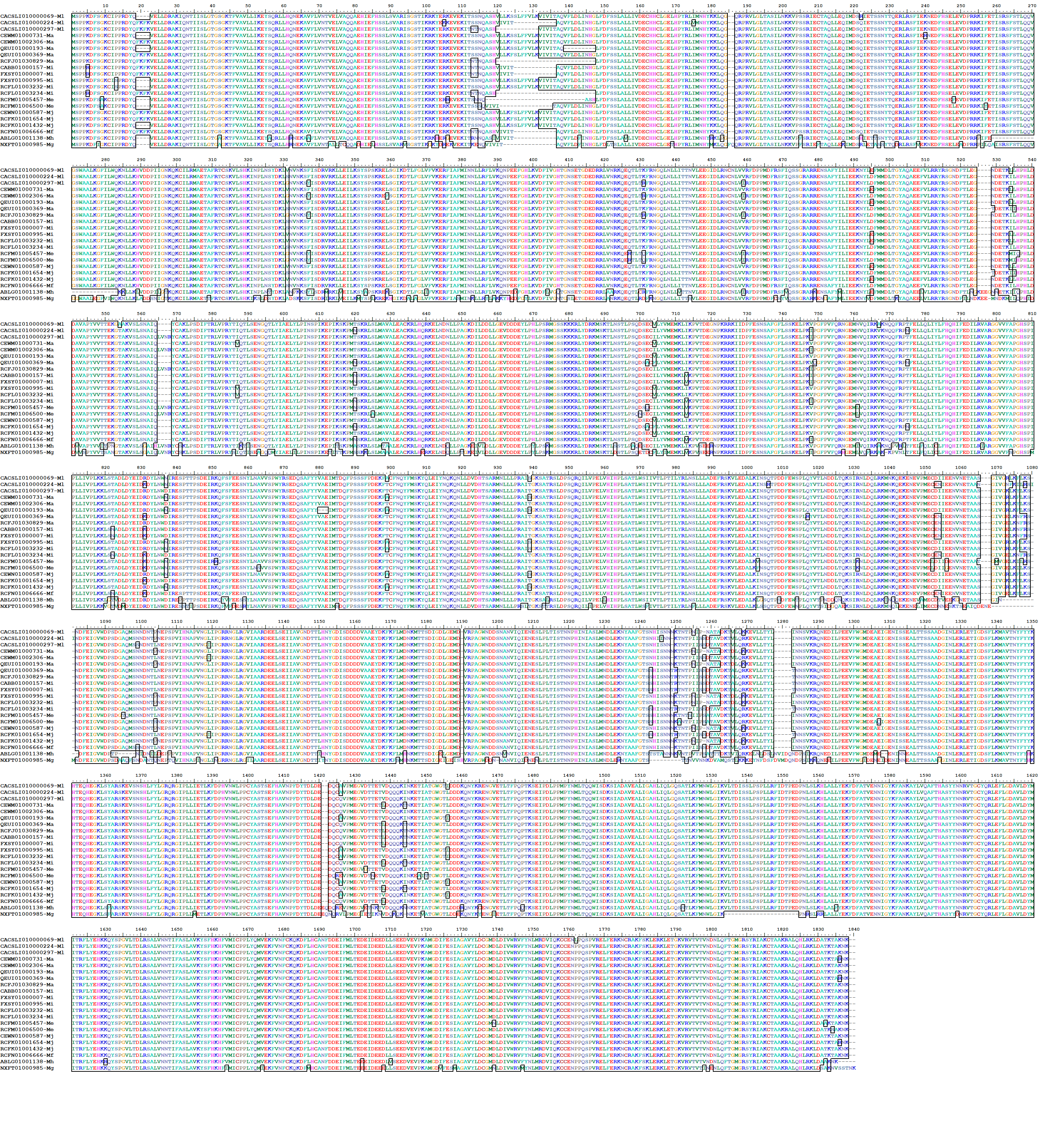
**(a)**

**(b)**

**
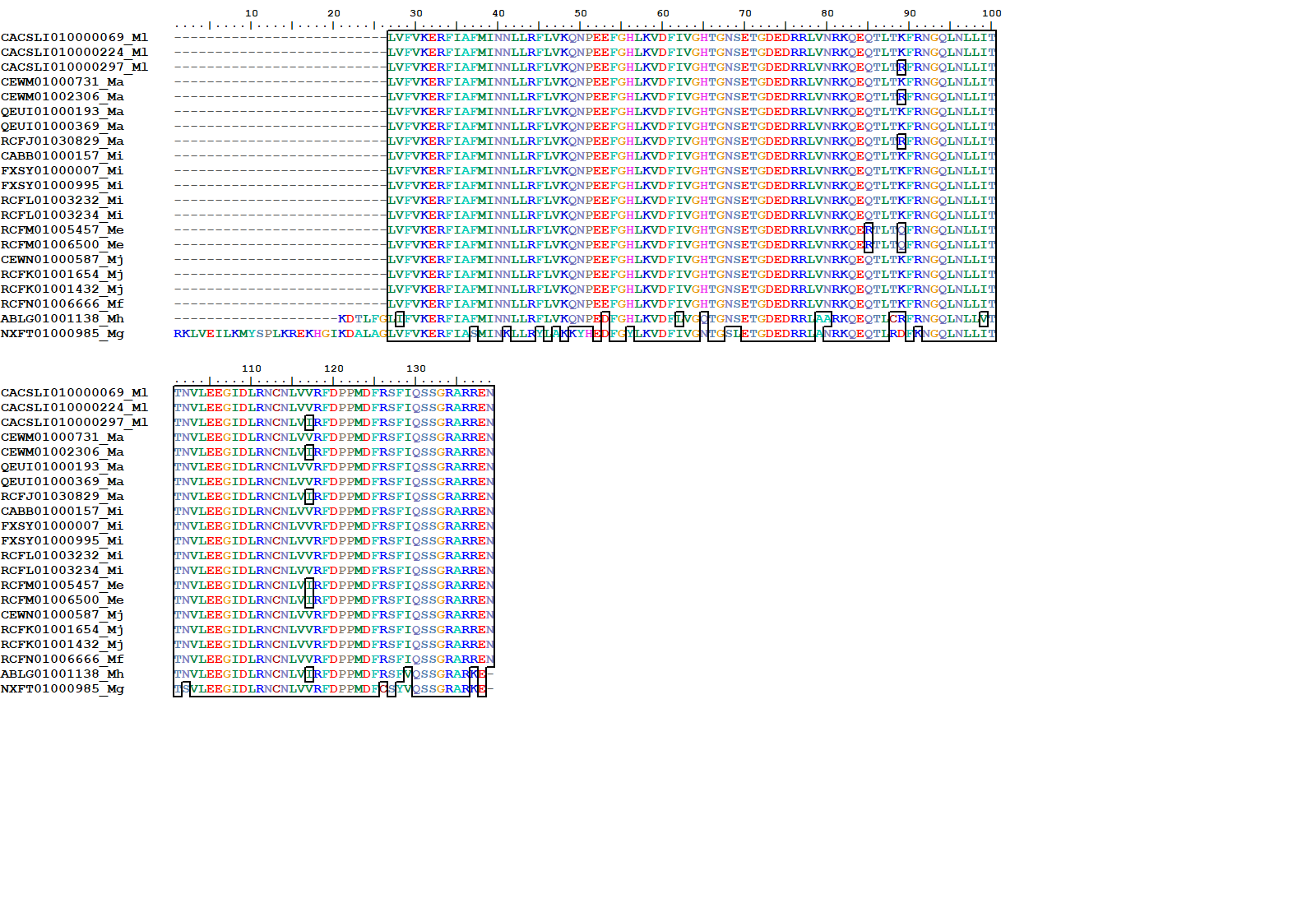
**

**(c)**

**
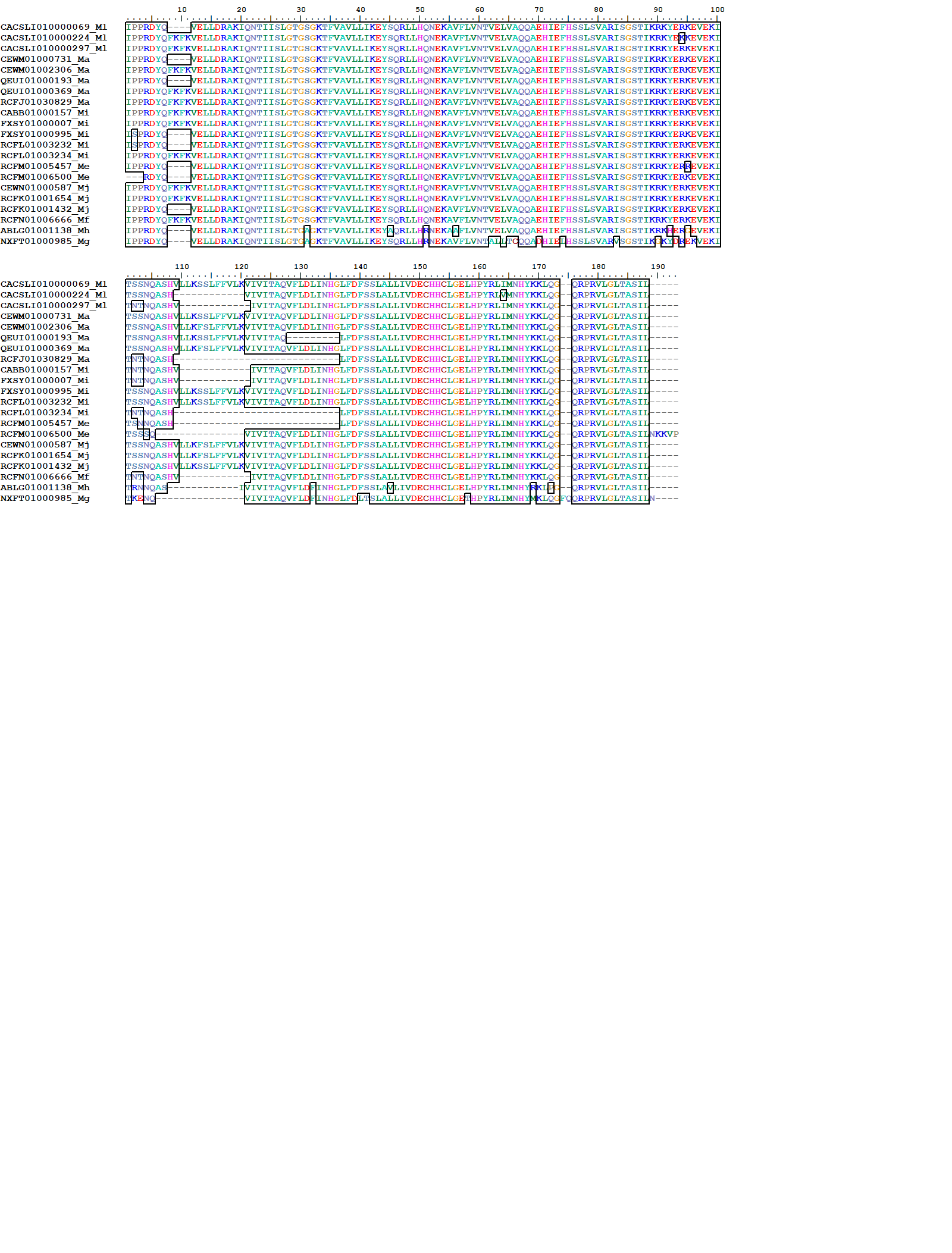
**


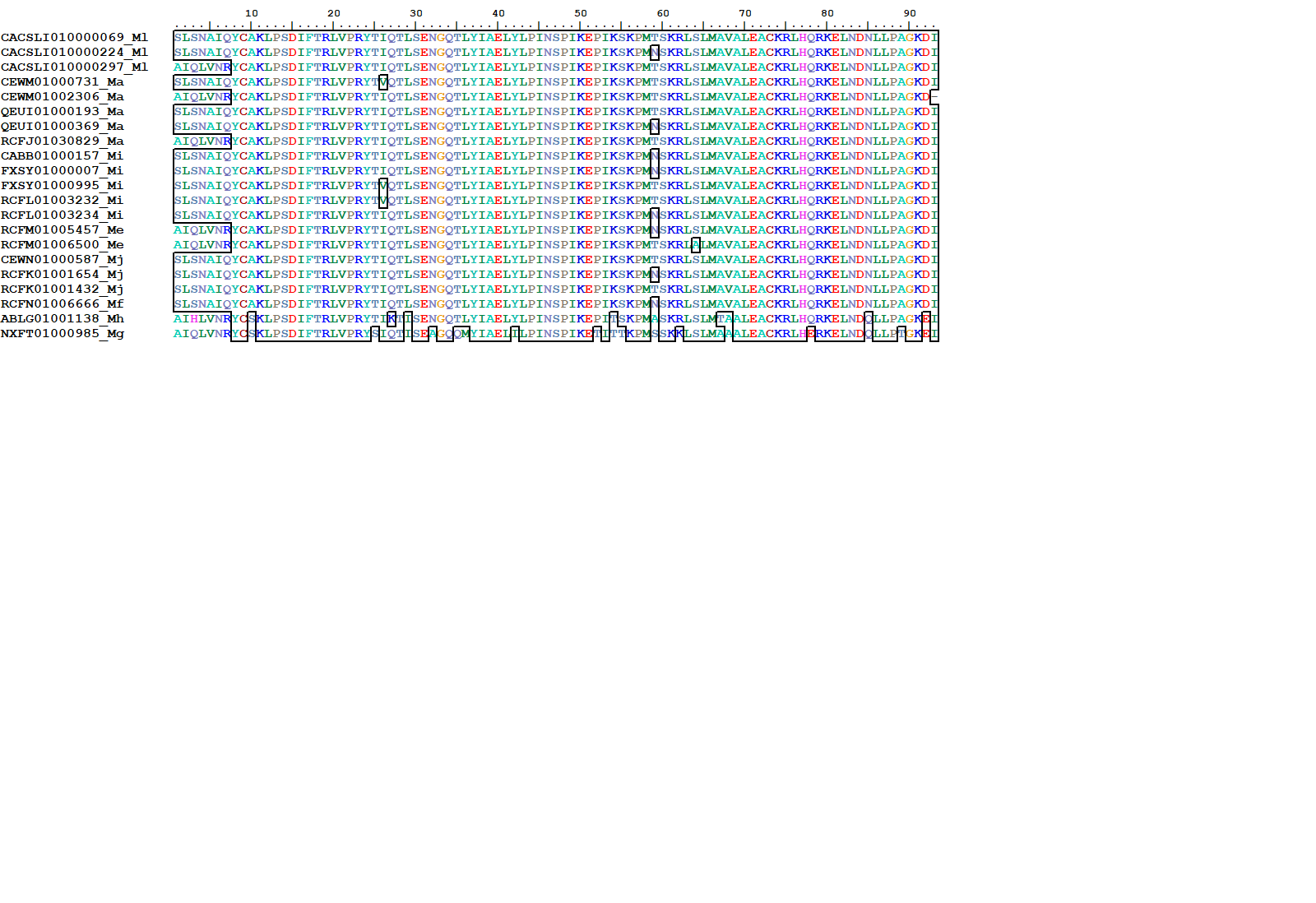
**(d)**


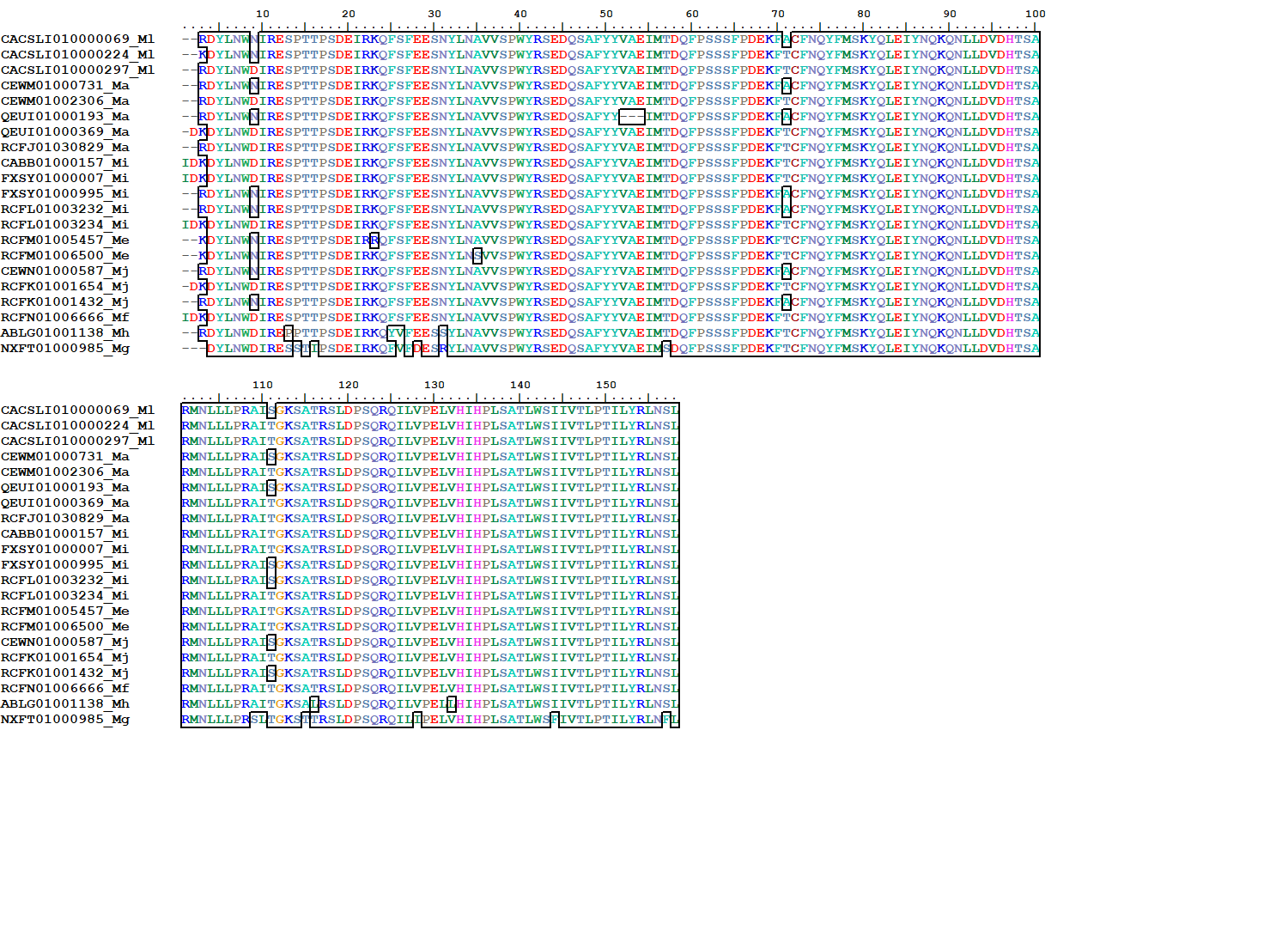
**(e)**


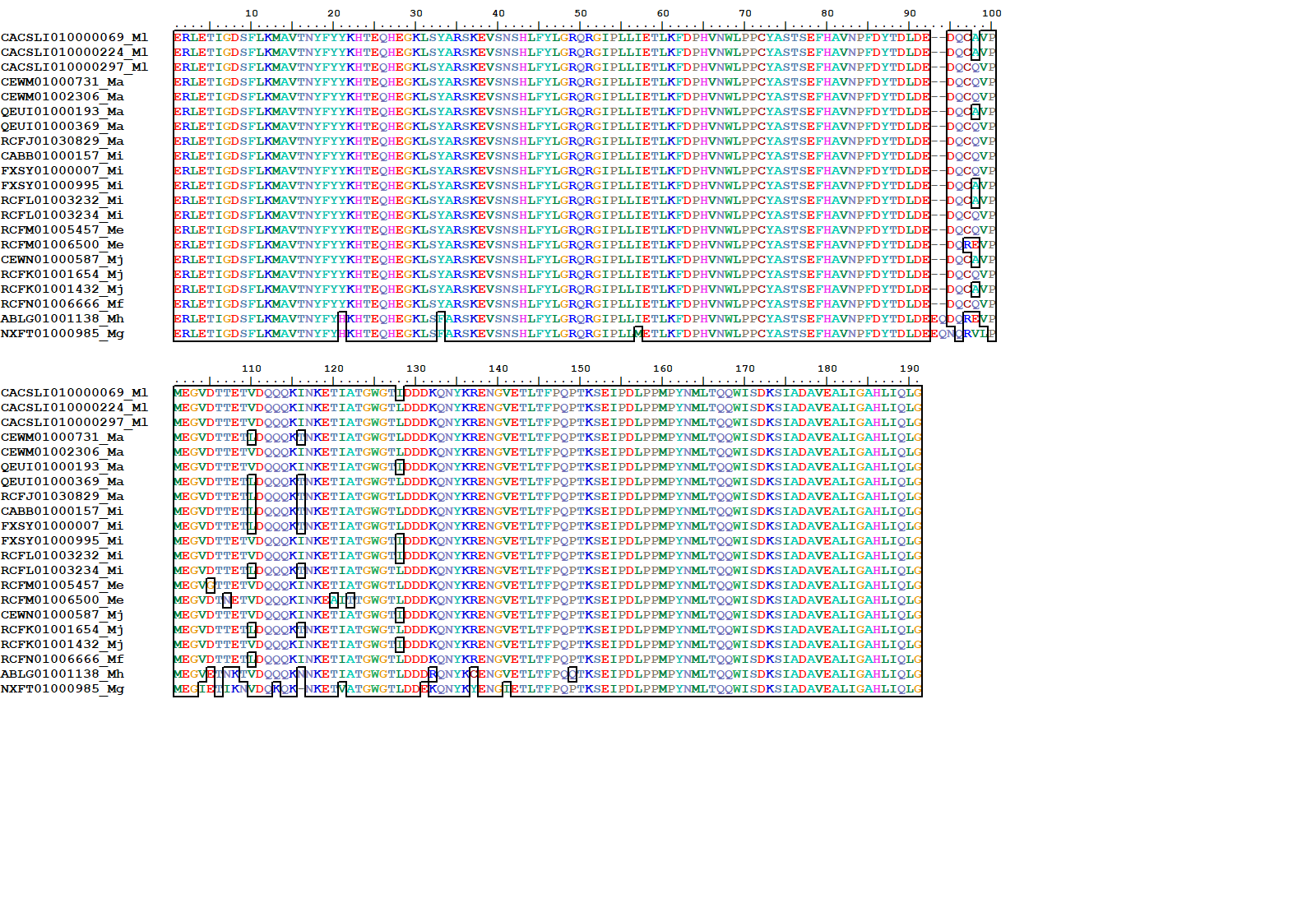
**(f)**


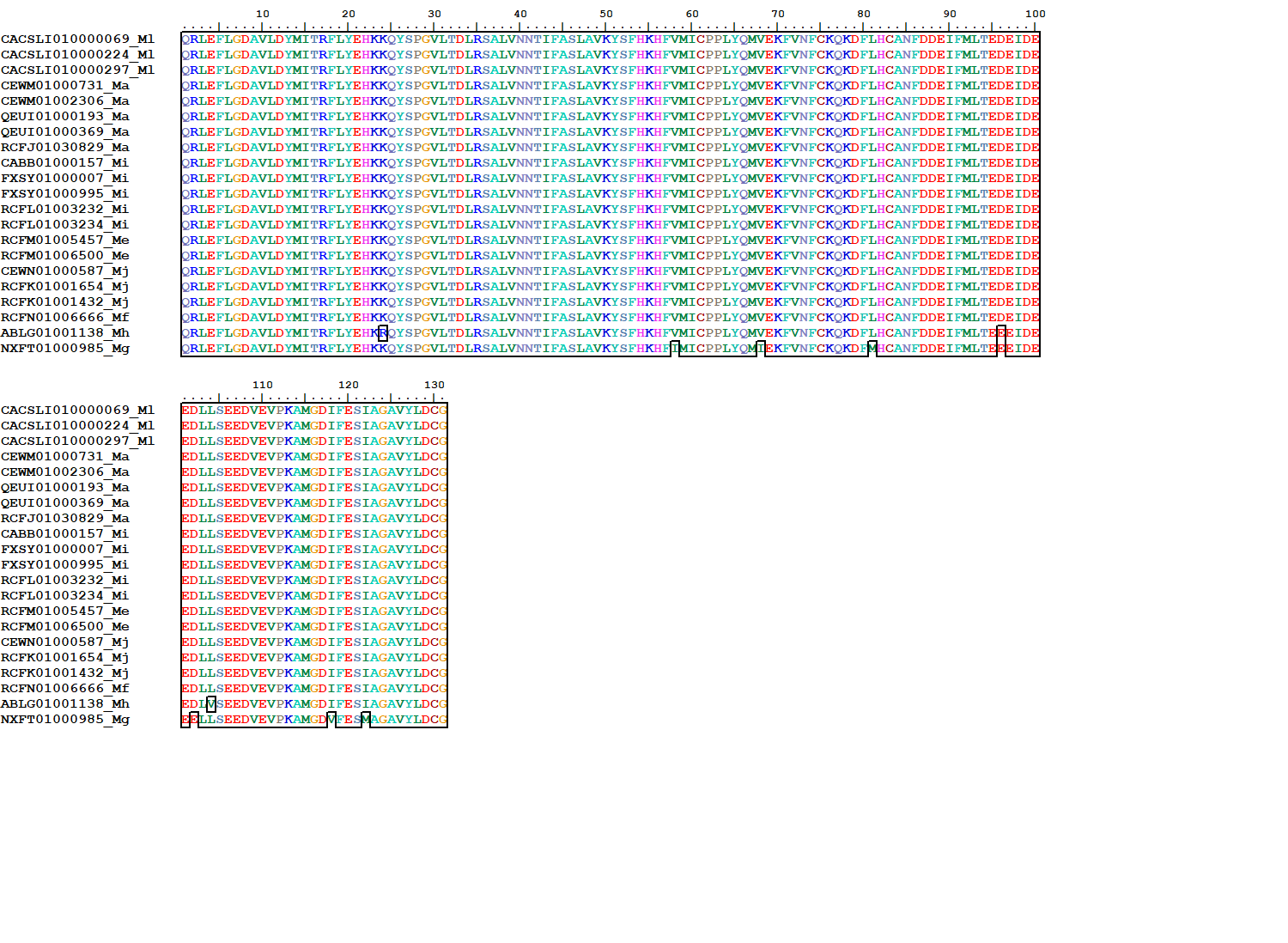
**(g)**


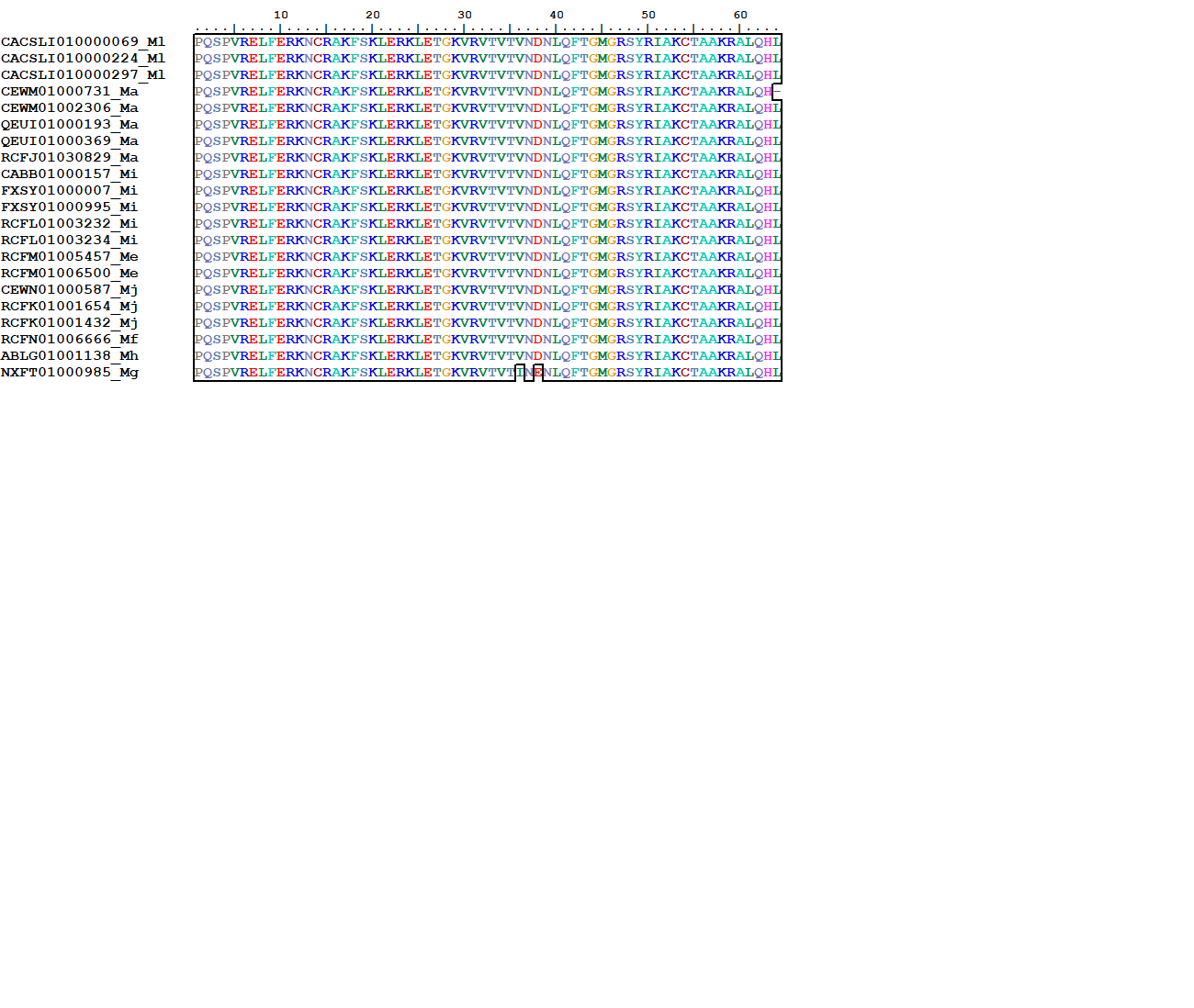
**(h)**

**Supplementary Table S4:** Primers used in standard and quantitative PCRs to the amplify parts of the coding regions of the seven domains of MiDCR-1.1 and for characterisation of transgenic plants.

| **Primer ID** | **Primer sequence 5′− 3′** | **Flanked Domain** | **Primer position on genomic contig (CABB01000157)** | ***M. incognita* cDNA Amplicon (bp)** |
| --- | --- | --- | --- | --- |
| MiDcr1HelATPB-F  MiDcr1HelATPB-R | TTGATGTAACAACCTCTGGGA  AATTCAAGGTTGAATTACTTGATCG | Helicase ATP binding domain | 19818-19945 | 122 |
| MiDcr1HelCT-F  MiDcr1HelCT-R | CTTGAATAAATGAACGAAAGTCCATC  AGACAGGCGATGAAGATAGG | Helicase C terminal | 17995-18177 | 182 |
| MiDcr1DSRBF-F  MiDcr1DSRBF-R | TTCCTGCAGGCAAAAGATTGTC  GGTACTGTGCAAAATTACCATCTG | Dicer Dimer | 17223-17520 | 251 |
| MiDcr1PAZ-F  MiDcr1PAZ-R | CATTCTAGCAGATGTATGGTCAAC  GAATCTCCAACTACACCTTCAGATG | PAZ | 16037-16398 | 273 |
| MiDcr1RiboC1-F  MiDcr1RiboC1-R | CATGTGGATCGAATTTAAGTGTTTC  CATTGACAACTTCGAGTGCTG | Ribonuclease III | 13597-13881 | 234 |
| MiDcr1RiboC2-F  MiDcr1RiboC2-R | ACAGAAATTAACAAACTTTTCGACC  TTAGCGTTTGGAATTCCTTGG | Ribonuclease III | 12434-12738 | 224 |
| MiDcr1DSRM-F  MiDcr1DSRM-R | TCCAATTTTCTTAAATGTTGAAGTGC  TTGGAACGAAAATTAGAAACAGGC | Double Stranded RNA binding motif | 11897-12039 | 143 |
| **Primer ID** | **Primer sequence 5′− 3′** | **Accession No.** | **Position on the sequence** | **Amplicon (bp)** |
| qMiActin-F  qMiActin-R | GTGCCATCCAAGCCGTTCTT  ATTGCGTGTGGCGAAGCATA | BE225475.1 | 29-149 | 121 |
| qMiDcr1-F  qMiDcr1-R | TCGTCGGGTGTTTGTGAATTA  ACATCCTCTTTCTGCAACTCTT | CABB01000157 | 15681 - 15870 | 143 |
| qMiDrsh1-F  qMiDrsh1-R | CAAGTGAATATCTTTACAAACAATTTCC  CCTGTGGAATAACCAAATATTTAACC | CABB01000477 | 15338 - 15453 | 116 |
| qMiAlg1-F  qMiAlg1-R | GGAATGCCAATTCAAGGTCAA  GCCAGGAAGTACAACACAAAC | CABB01000336 | 27756 - 27884 | 129 |
| qMiMut2-F  qMiMut2-R | CGACGATTGGCTTGCATTATT  GTTGATTGGCGTGTTCGTTTA | CABB01003815 | 1280 - 1363 | 84 |
| hpgfp-F  hpgfp-R | TTCACTGGAGTTGTCCCAATT  TAATGATCAGCGAGTTGCACG | M62653 | 47-570 | 524 |
| NptII-F  NptII-R | TGCTCCTGCCGAGAAAGTAT  AATATCACGGGTAGCCAACG | - | - | 364 |

**Supplementary Figure 2:** Original gel images from Figure 7 for characterisation of T2 transgenic *A. thaliana* lines. **(a)** PCR bands of the *nptII* gene (364 bp) from genomic DNA of transgenic lines. Lane M = 100 bp DNA marker, Lane 1 = No DNA control, Lane 2 – 10 = Bands from nine lines of dsgfp T2 plants, Lane 11-19 = Bands from nine lines of dsD3 T2 plants **(b)** RT-PCR of dsgfp hairpin from RNA of the nine T2 dsgfp lines. Lane M = 100 bp DNA marker, Lane 1 = No cDNA control, Lane 2-10 = Bands (524 bp) from cDNA of dsgfp from nine dsgfp lines. **(c)** RT-PCR of dsD3 hairpin from RNA of the nine T2 dsD3 lines. Lane M= 100 bp DNA marker, Lane 1 = no cDNA control, Lane 2-10 = Bands (251 bp) from cDNA of dsD3 from nine dsD3 lines.

**(a)**


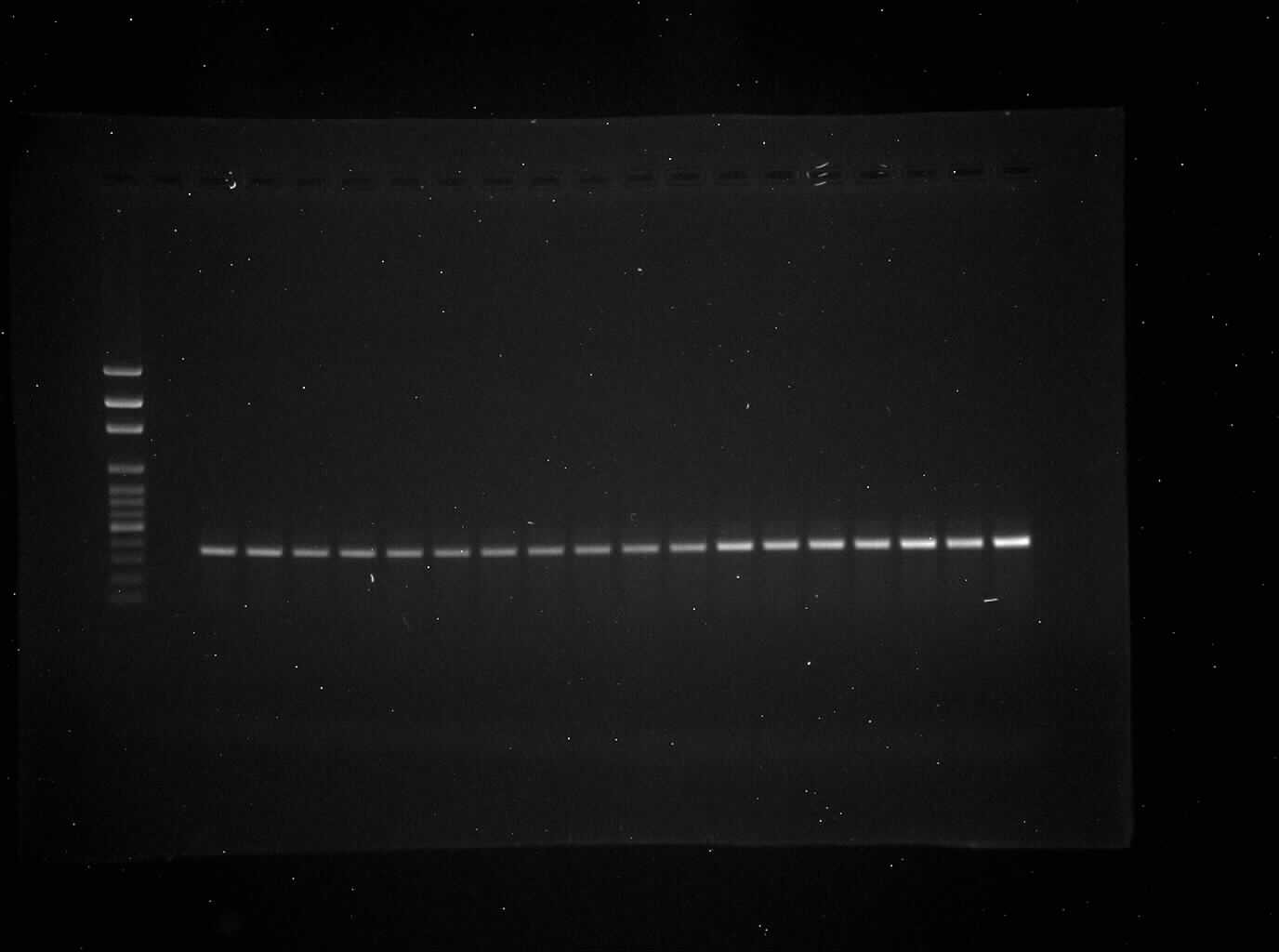


**(b)**


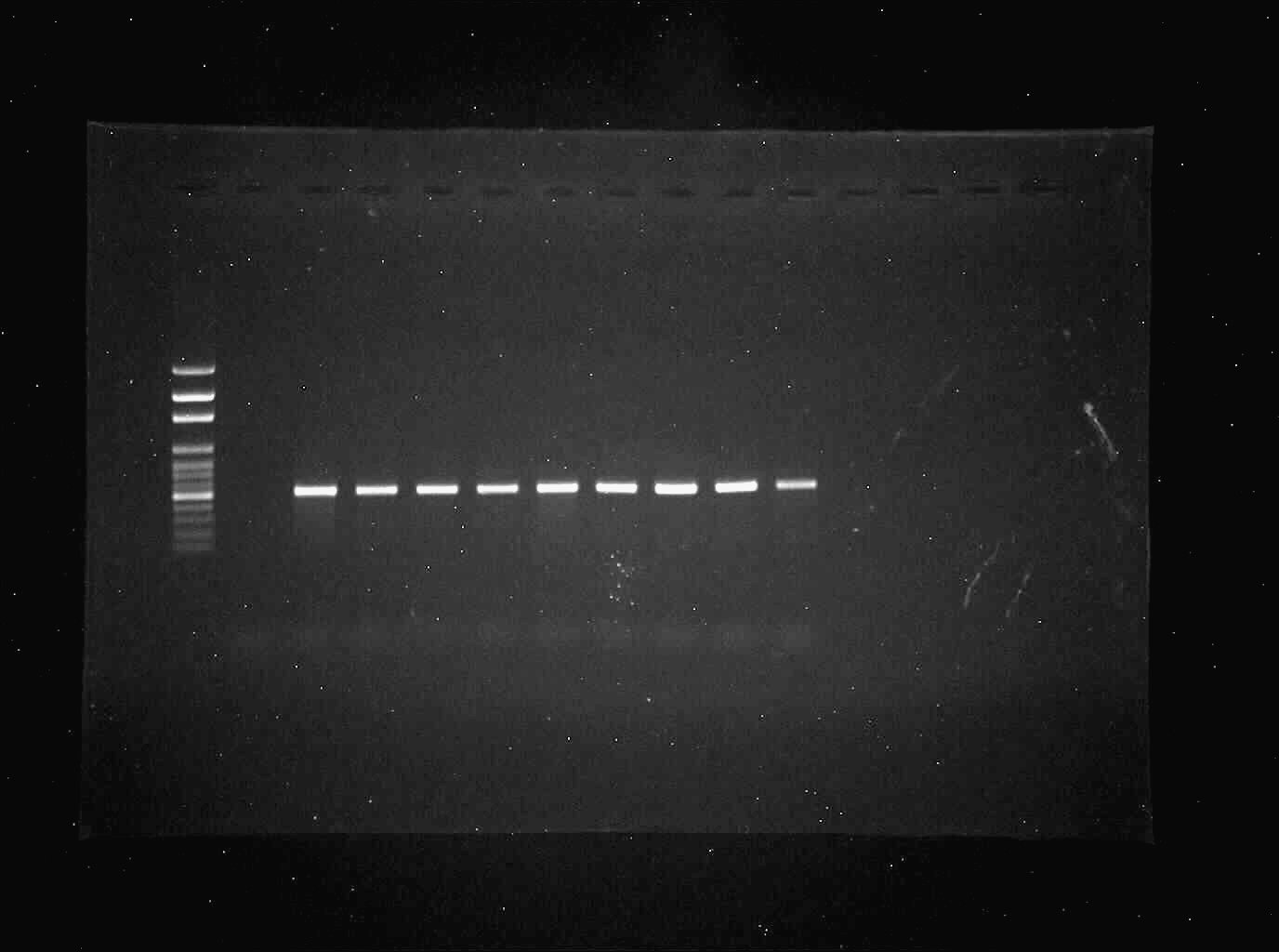


**(c)**


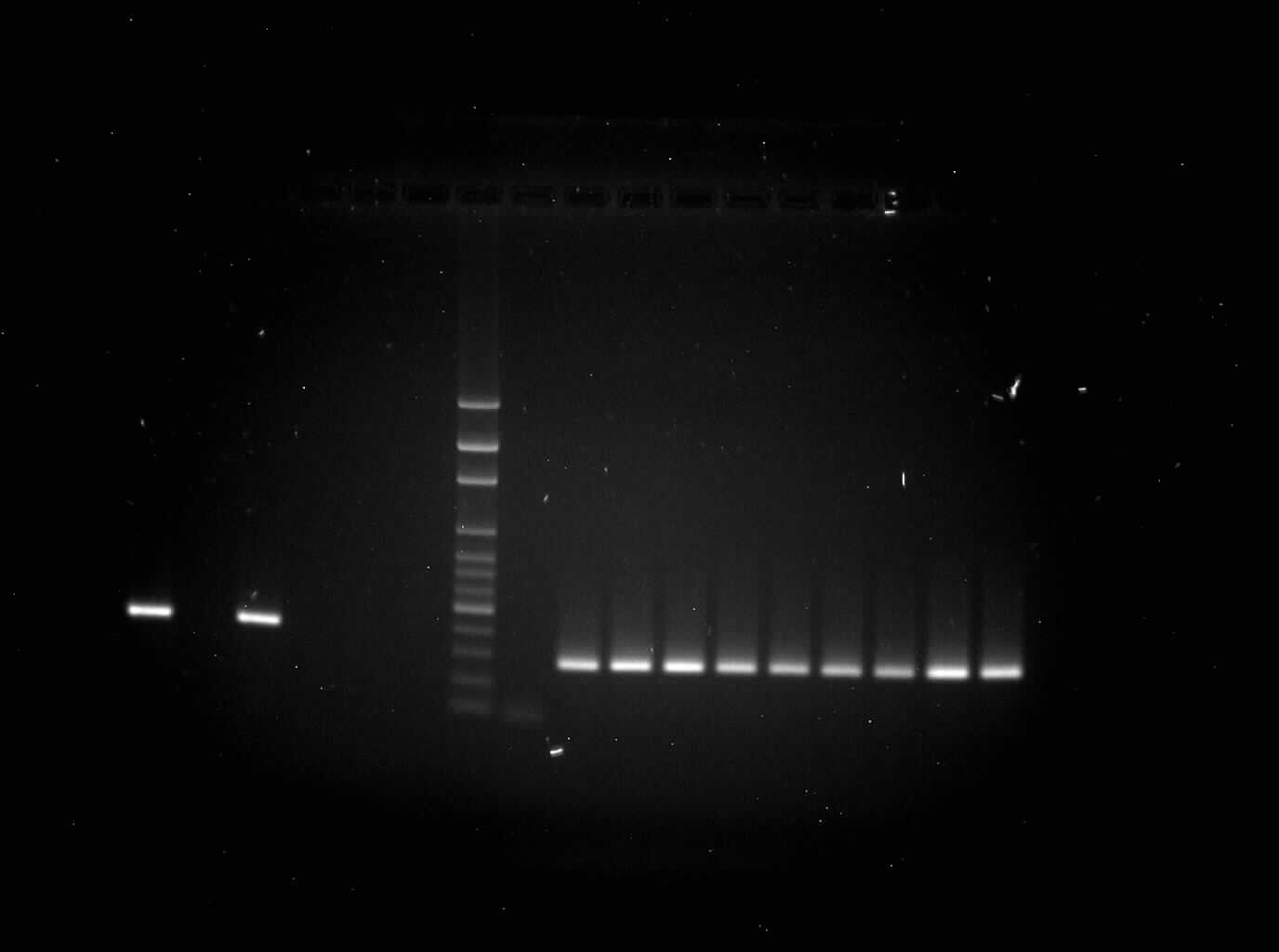

Supplement: Supplementary file 1 — Supplementary Information. [file 41598_2021_90363_MOESM1_ESM.doc]
